# Brønsted acid catalyzed mechanochemical domino multicomponent reactions by employing liquid assisted grindstone chemistry

**DOI:** 10.1038/s41598-023-27948-y

**Published:** 2023-01-25

**Authors:** Biplob Borah, Sidhartha Swain, Mihir Patat, Bhupender Kumar, Ketan Kumar Prajapat, Rathindranath Biswas, R. Vasantha, L. Raju Chowhan

**Affiliations:** 1 School of Applied Material Sciences, Centre for Applied Chemistry, Sector-30, Central University of Gujarat, Gandhinagar, 382030 India; 2 Department of Chemistry, Central University of Punjab, Bathinda, 151401 India

**Keywords:** Sustainability, Organocatalysis

## Abstract

Here, we have demonstrated a metal-free energy-efficient mechanochemical approach for expedient access to a diverse set of 2-amino-3-cyano-aryl/heteroaryl-4*H*-chromenes, tetrahydrospiro[chromene-3,4′-indoline], 2,2′-aryl/heteroarylmethylene-bis(3-hydroxy-5,5-dimethylcyclohex-2-enone) as well as tetrahydro-1*H*-xanthen-1-one by employing the reactivity of 5,5-dimethylcyclohexane-1,3-dione/cyclohexane-1,3-dione with TsOH⋅H_2_O as Brønsted acid catalyst under water-assisted grinding conditions at ambient temperature. The ability to accomplish multiple C–C, C=C, C–O, and C–N bonds from readily available starting materials via a domino multicomponent strategy in the absence of metal-catalyst as well as volatile organic solvents with an immediate reduction in the cost of the transformation without necessitates complex operational procedures, features the significant highlights of this approach. The excellent yield of the products, broad functional group tolerances, easy set-up, column-free, scalable synthesis with ultralow catalyst loading, short reaction time, waste-free, ligand-free, and toxic-free, are other notable advantages of this approach. The greenness and sustainability of the protocol were also established by demonstrating several green metrics parameters.

## Introduction

The current global challenges associated with environmental safety concerns due to chemical pollution caused by volatile organic solvents, toxic reagents, or hazardous chemicals during a chemical process both at laboratory and industrial scales; demand novel synthetic routes that provide expedient access to complex structural scaffolds by introducing environmentally benign reaction conditions with the main focus to reduces the cost-effectiveness of the transformation to make a pollution-free and sustainable environment^1–7^. With this perspective, mechanochemistry appears to be a highly attractive and promising environmentally benign activation method and has recently received a considerable and steadily increasing interest from synthetic potentiality as well as green and more sustainable chemistry point of view^8–11^. Despite, the tremendous growth accomplished in the mechanochemical synthesis by ball milling or grinding via a mortar and pestle (also termed as “grindstone chemistry”) in the past decades, which are mainly demonstrating the potentiality of the solvent-free green synthesis^12–14^; the use of a small amount of liquid in the reaction mixture at the time of grinding, termed as liquid assisted grinding (LAG)^15–19^ offers an outstanding environment to execute organic synthesis with better reactivity and selectivity as compared to traditional solution phase, alternative solvent-less synthesis as well as solid-state mechanosynthesis. In addition, the exploitation of water as solvent or co-solvent in organic synthesis poses significant challenges to the synthetic community due to the poor solubility of most of the organic compounds in water^20–23^. However, the attractive benefits associated with water include wide abundance, safe and eco-friendly, non-flammable, toxic-free, hazard-free nature, and sometimes provides better reactivity and selectivity as compared to other solvents; making water as the recent choice of reaction medium for organic synthesis with respect to synthetic efficiency and from the viewpoint of sustainability^24–26^.

On the other hand, multicomponent reactions (MCRs) which allowed the formation of multiple bonds in a single operation, have been demonstrated as a promising tool for the creation of diverse molecular structures with enhanced efficiency, reduced waste, and high atom economy from easily accessible simple and inexpensive starting materials by effortless mixing of the reactant. The ability to accomplish the requisite products in “one-pot” by operationally simple workup procedure without using complex purification techniques and avoiding the isolation of the reaction intermediate, features multi-component reaction a powerful strategy for green or sustainable synthesis^27–29^.

**Figure 1:**
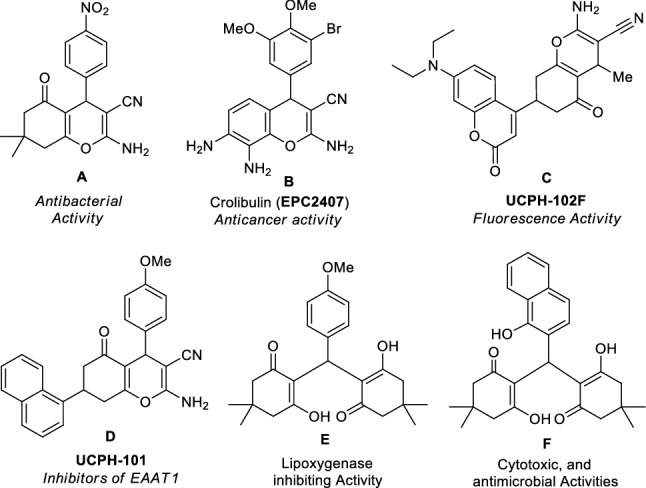
Examples of 2-amino-4*H*-chromene (**A–D**) and tetraketones (**E–F**) with potential therapeutic and optoelectronic application.

The construction of oxygen and nitrogen-containing heterocycles have always been attracted as a key fascinating area for organic synthesis owing to their widespread prevalence in the domain of drug design and developments, medicinal and pharmaceutical chemistry, as well as material sciences^30–32^. Chromene and their derivatives, particularly 2*H*-chromene-2-one also known as coumarin^6^, and 2-amino-4*H*-chromene^33^, possessing cyano functionality at the C-3 position, constitute such an imperative class of oxygen-containing heterocycles, which have potential therapeutic application in the treatment of diverse range disease manifestations such as Alzheimer’s disease, psoriatic arthritis, rheumatoid arthritis, amyotrophic lateral sclerosis, Parkinson’s disease, cancer therapy, as well as in Huntington’s disease.^34–37^ They frequently exist in the basic skeleton of numerous natural products, and synthetic drug candidates and hold a pivotal position in medicinal chemistry owing to their notable pharmacological activities including anti-allergic, anticoagulant, antitumor, antimicrobial, antiproliferative, antioxidant, and antifungal (Fig. 1, Entry A–D)^38–42^. Some material science application such as fluorescence markers, optical brighteners, and laser dyes, has been established in the last decades^43–45^. They are also used in cosmetics, biodegradable agrochemicals, pigments, etc.^46–48^.

Recognizing such prominent features and therapeutic potential of 2-amino-3-cyano-4*H*-chromene, the synthesis of heterocycles possessing chromene moiety is of increasing scientific and academic interest. Accordingly, several synthetic methods to realize this heterocycle by using different catalytic systems such as heterogeneous or homogeneous catalysts^49–51^, magnetic nanoparticles^52–54^, ionic liquids^55–57^, metal complex^58–60^, organocatalysts^61–64^, polymers^65^, carbon quantum dots^66^, organic–inorganic hybrids^67^, metal–organic frameworks^68^, deep eutectic solvents^69^, as well as catalyst-free conditions^70,71^ were developed in the last decades.

At the same time, 2,2′-methylene-bis(3-hydroxy-5,5-dimethylcyclohex-2-enone) also known as tetraketones, belonging to one of the most promising classes of oxygen-containing heterocycles, have received substantial attention of chemists and pharmacologist both at academic and industrial level for their remarkable therapeutic activities such as tyrosinase inhibitors, antibacterial, antioxidant, antiviral, and lipoxygenase inhibitors, etc. (Fig. 1, Entry E–F)^72–76^. Besides these, they were considered as versatile building blocks for the construction of value-added compounds such as xanthendione, acridindione, and thiaxanthenes that offer significant applications in the pharmaceutical industry and laser technology^77–79^. A vast array of synthetic procedures has been developed by employing different catalytic systems such as In(OTf)_3_^80^, urea^81^, SmCl_3_^82^, Nano Fe/NaY zeolite^83^, Pd NPs^84^, Fe_3_O_4_@SiO_2_–SO_3_H^85^, Na_2_CaP_2_O_7_^86^, and PPA–SiO_2_^87^.

Notwithstanding, the reported works offer several advantages, most of them suffer serious drawbacks such as the utilization of toxic materials, expensive catalysts, as well as metal catalysts, volatile organic solvents, prolonged reaction time, requirements of high energy conditions, excess amounts of solvents, tedious work-up procedures, and high loading of catalysts, etc.

**Figure 2:**
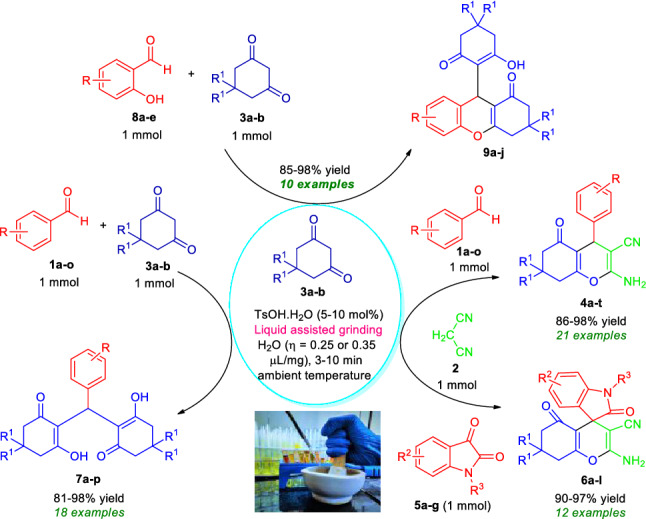
Liquid assisted grinding accelerated one-pot Brønsted acid-catalyzed domino multicomponent reactions for the synthesis of diverse complex-fused and spiro-heterocycles.

Considering all these aspects and the synergic combination of the features of liquid assisted grinding as an eco-friendly activation method with the synthetic efficiency associated to the metal-free multicomponent reactions to create a pollution-free environment, the present research demonstrated an Brønsted acid-catalyzed mechanochemically scalable one-pot approach for the rapid access to a diverse set of highly functionalized 2-amino-3-cyano-aryl/heteroaryl-4*H*-chromenes **4a–t**, 2-amino-3-cyano-tetrahydrospiro-[chromene-3,4′-indoline] **6a–l**, 2,2′-aryl/heteroarylmethylene-bis(3- hydroxy-5,5-dimethylcyclohex-2-enone) derivatives **7a–p** as well as tetrahydro-1*H*-xanthen-1-one **9a–j** by employing the reactivity of 5,5-dimethylcyclohexane-1,3-dione/cyclohexane-1,3-dione **3a–b** based on a domino multicomponent strategy under energy-efficient grindstone chemistry with water as grinding additives at ambient conditions (Fig. 2).

## Result and discussion

**Figure 3:**
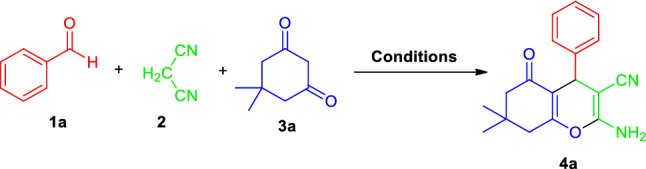
Optimization of reaction conditions for the synthesis of 2-amino-3-cyano-4*H*-chromenes.

Recently, the use of liquid-assisted grinding techniques for controlling or accelerating organic transformation has become one of the current interests of synthetic organic chemistry in the move towards green or sustainable synthesis. In line with this, along with establishing the standard reaction condition for the synthesis of 2-amino-3-cyano-4*H*-chromene derivatives **4a–t**, we choose the initial multicomponent reaction of benzaldehyde **1a**, malononitrile **2**, and 5,5-dimethylcyclohexane-1,3-dione **3a** as the representative example (Fig. 3) that was being executed in different catalytic as well as non-catalytic systems under different reaction conditions (Table 1). An immediate analysis of the data presented in Table 1 for the preparation of our desired product **4a** revealed that the reaction performed without using any catalyst failed to yield any product even after 24 h under grinding conditions (Table 1, Entry 1). Similarly, with K_2_CO_3_, and Na_2_CO_3_; no product formation was noticed after a long reaction time (Table 1, Entry 2–3). Alternatively, switching the catalytic system from inorganic bases to organic bases including Et_3_N, DABCO, DBU, and DMAP in grinding conditions leads to the corresponding products in poor to moderate yields with slightly reduced reaction times (Table 1, Entry 3–6). The excellent yield of the product was achieved when the reaction was executed by grinding the reactants, benzaldehyde **1a**, malononitrile **2**, and 5,5-dimethylcyclohexane-1,3-dione **3a** in a mortar and pestle in presence of 10 mol% of Brønsted acid TsOH⋅H_2_O by using water (*η* = 0.35 μL/mg) as the liquid assisted grinding additives (LAGs) at ambient temperature within 5 min. The effects of different energy inputs on the model reaction in presence of TsOH⋅H_2_O were also investigated among which grinding proven to be efficient compared to others (stirring or reflux) as they required prolonged times and the yield of the product was not satisfactory (Table 1, Entry 7). With the aid of TFA, the corresponding product **4a** was achieved in 78% yield within 36 min under grinding conditions (Table 1, Entry 8). In the meantime, the model reaction under ultrasound irradiation was found to slightly proceed in presence of NH_2_SO_3_H as the organocatalyst to deliver the desired product **4a** in 70% yield where using grinding only 65% of the product was isolated after 2 h (Table 1, Entry 9). The applicability of taurine as a green bio-based catalyst under grinding as well as reflux conditions were also demonstrated. But no improvements in the rate of the reaction and complete conversion were observed (Table 1, Entry 10). The synergic combination of the features of TsOH⋅H_2_O as operationally simple, inexpensive, and readily available metal-free catalyst along with the efficiency associated to the use of water as a safe, eco-friendly green liquid assisted grinding solvent prompted us to consider water-assisted grinding of benzaldehyde, malononitrile, and 5,5-dimethylcyclohexane-1,3-dione with the aid of a simple mortar and pestle by employing 10 mol% of TsOH⋅H_2_O as the optimum reaction condition for the synthesis of 2-amino-3-cyano-4*H*-chromene at ambient temperature.

**Table 1:** Screening of catalytic system for the synthesis of 2-amino-3-cyano-4*H*-chromenes.

Entry	Catalyst (loading)	Condition	Time^a^	Yield (%)^b^
1	Catalyst-free	Grinding	24 h	NR
2	K_2_CO_3_ (10 mol%)	Grinding	1 h	NR
3	Na_2_CO_3_	Grinding	2 h	NR
3	Et_3_N (10 mol%)	Grinding	1.5 h	58
4	DABCO (10 mol%)	Grinding	0.5 h	45
5	DBU (10 mol%)	Grinding	40 min	50
6	DMAP (10 mol%)	Grinding	1 h	38
**7**	**TsOH**⋅**H**_**2**_**O** **(10 mol%)**	**Grinding**	**5 min**	**96**
		Stirring	2 h	80^c^
		Reflux	3 h	76^c^
8	TFA (10 mol%)	Grinding	25 min	78
9	NH_2_SO_3_H (10 mol%)	Grinding	2 h	65
		US^d^	36 min	70^e,f^
10	Taurine (10 mol%)	Reflux	78 min	42^c^
		Grinding	55 min	65^f^

Reaction condition: Benzaldehyde **1** (1.0 mmol), malononitrile **2** (1.0 mmol), and 5,5-dimethylcyclohexane-1,3-dione **3a** (1.0 mmol) in the presence or absence of catalyst(s) in H_2_O (*η* = 0.35 μL/mg) as LAGs [*η* = V (liquid; μL)/m (reagents; mg)] at ambient temperature.

*NR* no reaction.

Significant values are in [bold].

^a^Progress of the reaction was scrutinized by TLC.

^b^Yields of the isolated products.

^c^Reaction was carried out with 3 mL of water as the solvent.

^d^Ultrasound irradiation.

^e^Sonication was performed using an ultrasonic bath (model: SB-3200DT) with an operating frequency of 40 kHz and nominal power of 180 W.

^f^Using 20 mol% of taurine.

**Figure 4:**
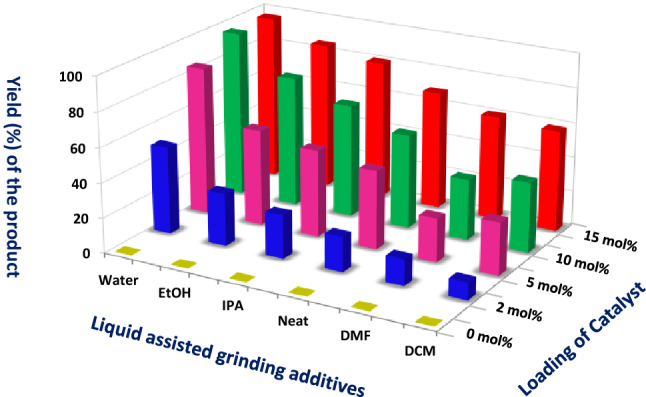
Screening of LAGs for the synthesis of **4a** with different loading of the catalyst.

The effect of different LAGs and the amount of catalyst on the model reaction was further examined (Fig. 4). The preliminary used EtOH, IPA, DMF, DCM as LAGs (*η* = 0.35 μL/mg) and neat grinding under the influences of TsOH⋅H_2_O reduces the yield of the products even after increasing the amount of the catalyst from 2 to 15 mol%. To our delight, the best results were accomplished while performing the reaction with water by employing 10 mol% of catalyst at ambient conditions. Further, increases or decreases in the catalyst loading had no suspicious significance in the rate of the reaction. The temperature of the reaction mixture (monitored by using IR thermometer of accuracy range ± 0.03 °C) was found to be slightly raised from 35.4 to 36.5 °C after 1 min of grinding and after 1.5 min a slight change was observed and then remained constant during the course of the reaction.

**Figure 5:**
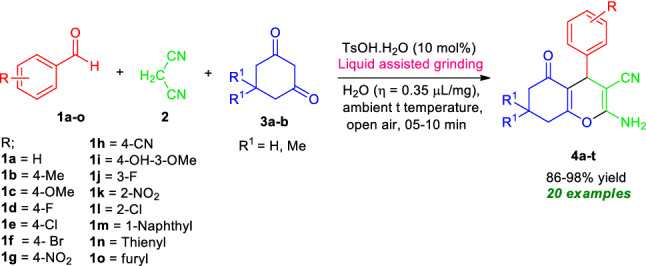
Synthesis of 2-amino-3-cyano-4*H*-chromenes **4** under the standard conditions.

With the optimum conditions in hand, the general feasibility of the present protocol was demonstrated by treating a diverse set of aryl aldehydes **1b–o** with malononitrile **2**, and 5,5-dimethylcyclohexane-1,3-dione **3a** in presence of 10 mol% of TsOH⋅H_2_O in water (*η* = 0.35 μL/mg) using liquid assisted mechanical grinding conditions (Fig. 5, Table 2). Surprisingly, the corresponding 2-amino-3-cyano-4H-chromene products **4a–t** were obtained in good to excellent yield in all the cases within 5–10 min. Aldehydes possessing electron-donating groups including methyl and –OMe afforded the desired products **4b** and **4c** in 88% and 86% yield respectively at 8 min (Table 2). While substitution by different halogen groups like F, Cl, Br on C-4 and C-3 positions of the phenyl ring of the aldehyde was found to be efficiently delivered the products **4d**, **4e**, **4f**, **4j**, 4**l** in excellent yields. Strong electron-withdrawing groups like NO_2_ and CN attached to the benzaldehyde ring proceeded smoothly under the standard condition. The reaction was also found to be well worked for heteroaryl aldehydes, getting the respective products **4m**, **4n**, and **4o** in 90%, 92%, and 93% yields correspondingly. Similarly, using cyclohexane-1,3-dione **3b** instead of 5,5-dimethylcyclohexane-1,3-dione **3a**, the three-component reaction with various aryl as well as heteroaryl aldehydes and malononitrile under the standard reaction conditions afforded the desired products **4p–t** in good to excellent yield (86–97% yield) within 6 to 10 min (Table 2).

**Table 2:**
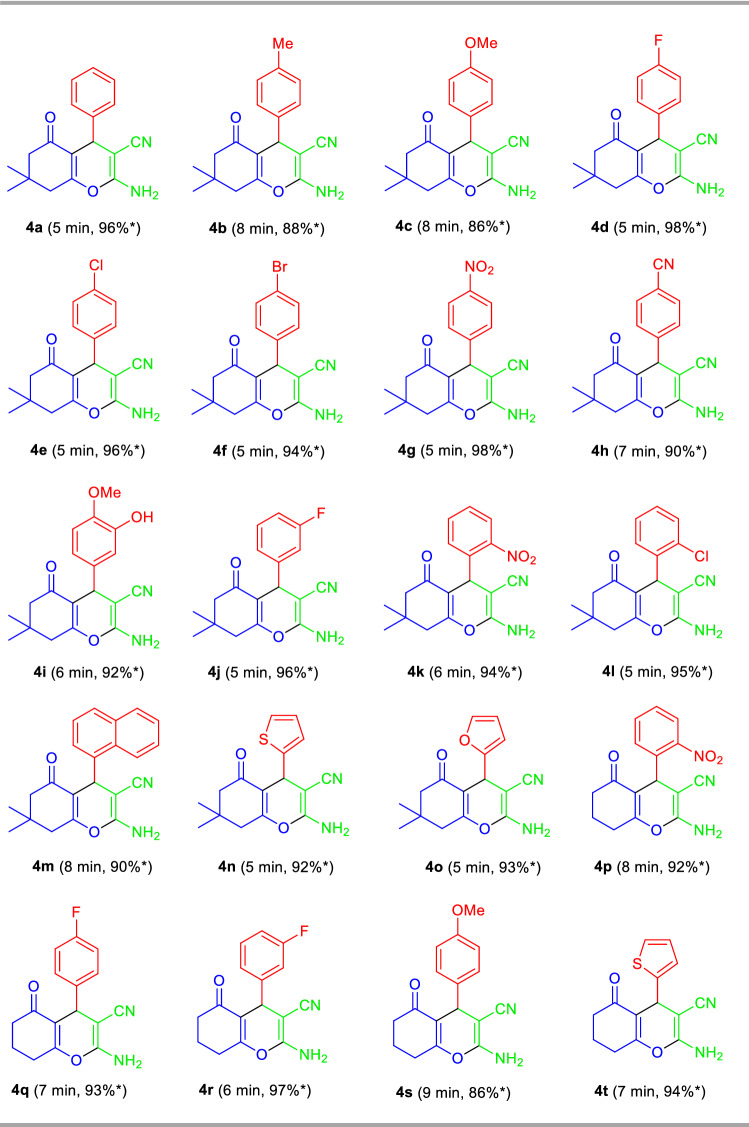
Substrate scopes for the synthesis of 2-amino-3-cyano-4*H*-chromenes derivatives **4a–t**.

Reaction condition: Aldehyde **1a-o** (1.0 mmol), malononitrile **2a** (1.0 mmol), and 5,5-dimethylcyclohexane-1,3-dione **3a**/cyclohexane-1,3-dione **3b** (1.0 mmol) in the presence of 10 mol% of TsOH⋅H_2_O in H_2_O (*η* = 0.35 μL/mg) as LAGs [*η* = V (liquid; μL)/m (reagents; mg)] at ambient temperature. *Isolated yield.

**Figure 6:**
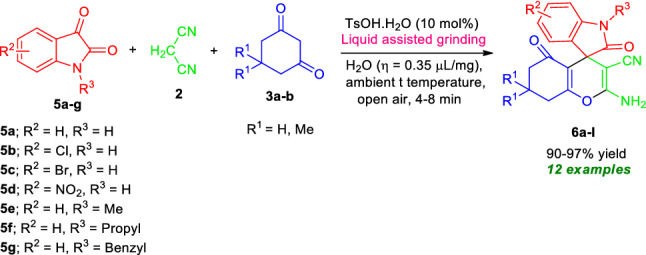
Synthesis of spirooxindoles **6** under the standard conditions.

Enlightened by these results, we extended this domino three-component reaction of malononitrile **2**, 5,5-dimethylcyclohexane-1,3-dione **3a** with isatins **5a** by replacing aryl/heteroaryl aldehydes **1a–o** in presence of 10 mol% of TsOH⋅H_2_O with water as LAGs under mechanochemical grinding at ambient temperature for 5 min (Fig. 6, Table 3). To our delight, the corresponding 2-amino-7,7-dimethyl-2′,5-dioxo-5,6,7,8- tetrahydrospiro[chromene-4,3′-indoline]-3-carbonitrile **6a** was obtained in 96% yield only at 5 min. Then we carried out a total of eleven reactions with different types of isatin derivatives such as 5-chloro isatin **5b**/5-bromo isatin **5c**/5-nitro isatin **5d**/*N*-methyl isatin **5e**/*N*-propyl isatin **5f**/*N*-benzyl isatin **5g** with malononitrile **2** and 5,5-dimethylcyclohexane-1,3-dione **3a**/cyclohexane-1,3-dione **3b** under the standard conditions to furnished the respective products 2-amino-3-cyano-tetrahydro-spiro[chromene-4,3′-indoline] **6b–l** in 90–97% yields at 4 to 8 min (Table 3). Isatins having substitutions like chloro, bromo, nitro are well suited for this reaction, delivering the products **6b**, **6c**, **6d**, and **6i** in good to excellent yield. Similarly, *N*-unsubstituted isatins (R^3^ = H) (**5a–d**) as well as *N*-substituted isatins (R^3^ = Me, propyl, benzyl) (**5e–g**), were efficiently tolerated by this reaction.

**Table 3:**
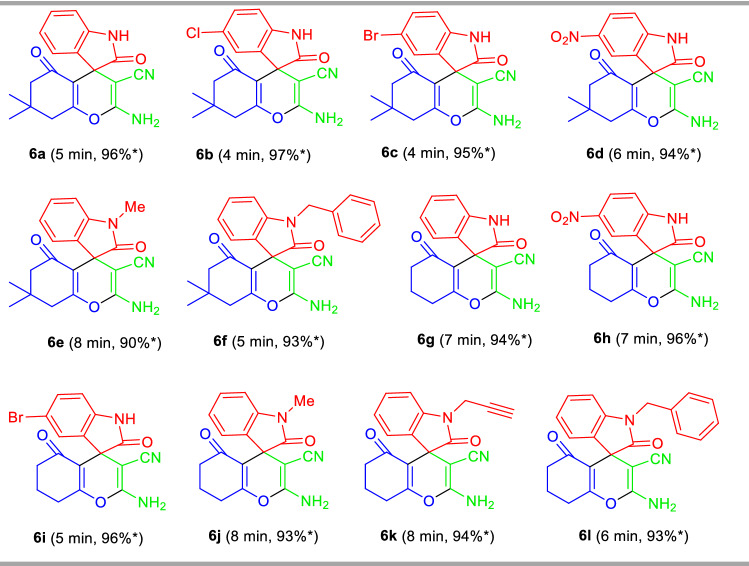
Substrate scopes for the synthesis of 2-amino-tetrahydrospiro[chromenes-3,4′-indoline]-3-carbonitriles **6a-l**.

Reaction condition: Isatins **5a–g** (1.0 mmol), malononitrile **2** (1.0 mmol), and 5,5-dimethylcyclohexane-1,3-dione **3a**/cyclohexane-1,3-dione **3b** (1.0 mmol) in the presence of 10 mol% of TsOH⋅H_2_O in H_2_O (*η* = 0.35 μL/mg) as LAGs [*η* = V (liquid; μL)/m (reagents; mg)] at ambient temperature. *Isolated yield.

**Figure 7:**
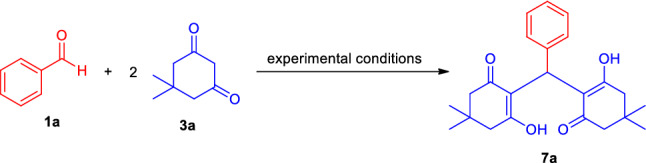
Model reaction for optimization studies.

To further explore the applicability of the present approach, we investigated a series of trial reactions between 1 equivalent of benzaldehyde **1a** with 2 equivalent of 5,5-dimethylcyclohexane-1,3-dione **3a** under different reaction conditions (Fig. 7). Initial execution of the reaction in the absence of any catalyst as well as any solvents or in presence of EtOH either in stirring, grinding, or reflux conditions, formation of the desired product 2,2′-(phenylmethylene)bis(3-hydroxy-5,5-dimethylcyclohex-2-enone) **7a** was not detected (Table 4, Entry 1–3). Grinding the reactants under catalyst-free conditions in water furnished the desired product in a 40% yield (Table 4, Entry 2). Then we employed 2 mol% of TsOH⋅H_2_O as the easily accessible catalyst in the model reaction in solvent-free stirring or grinding conditions, but no improvements in the rate of the reaction were observed (Table 4, Entry 5–6). Surprisingly, switching the neat grinding to liquid assisted grinding by employing ethanol, and water as the liquid assisted grinding additive (LAGs), an increase in the yield of the product was observed (Table 4, Entry 7–8). With water as the LAGs, the desired product **7a** was achieved in 87% yield in only 8 min under the influences of 2 mol% of the catalyst (Table 4, Entry 8). By increasing the loading of the catalyst from 2 to 5 mol%, the yield of the product was found to be increased up to 95% in only 3 min (Table 4, Entry 9). However, there was no change in the product yield with increased loading of the catalyst from 5 to 10 mol% (Table 4, Entry 10). In the meantime, the reaction rate was found to be slow when we changed the reaction condition with 5 mol% of TsOH⋅H_2_O in EtOH, ACN as LAGs under mechanical grinding (Table 4, Entry 11–12). On the other hand, the unusual requirements of long reaction time and low yield of the product for the reaction conducted in reflux or ultrasonication with 5 mol% of TsOH⋅H_2_O using water as the solvent system suggest the mechanochemical grinding as the best method of choice for this three-component reaction (Table 4, Entry 13–14). Furthermore, the yield of the product was also decreased when employing 5 mol% of secondary amine catalyst L-proline in water under grinding or reflux conditions (Table 4, Entry 15–16).

**Table 4:** Optimization of reaction conditions for the synthesis of 2,2′-aryl/heteroaryl-methylene-bis(3-hydroxy-cyclohex-2-enone) derivatives.

Entry	Catalyst	Loading	Solvent	Condition	Time (min)^a^	Yield (%)^b^
1	–	–	Neat	Stirring	180	NR
2	–	–	Neat	Grinding	70	NR
3^c^	–	–	EtOH	Reflux	120	NR
4^d^	–	–	H_2_O	Grinding	30	40
5	TsOH⋅H_2_O	2 mol%	Neat	Stirring	90	NR
6	TsOH⋅H_2_O	2 mol%	Neat	Grinding	40	38
7^d^	TsOH⋅H_2_O	2 mol%	EtOH	Grinding	25	53
8^d^	TsOH⋅H_2_O	2 mol%	H_2_O	Grinding	8	87
**9**^d^	**TsOH**⋅**H**_**2**_**O**	**5 mol%**	**H**_**2**_**O**	**Grinding**	**3**	**95**
10^d^	TsOH⋅H_2_O	10 mol%	H_2_O	Grinding	3	96
11^d^	TsOH⋅H_2_O	5 mol%	EtOH	Grinding	15	69
12^d^	TsOH⋅H_2_O	5 mol%	ACN	Grinding	60	20
13^e^	TsOH⋅H_2_O	5 mol%	H_2_O	Ultrasound	90	55
14^c^	TsOH⋅H_2_O	5 mol%	H_2_O	Reflux	150	68
15^d^	L-proline	5 mol%	H_2_O	Grinding	20	45
16^c^	L-proline	5 mol%	H_2_O	Reflux	90	28

Reaction condition: Benzaldehyde **1a** (1.0 mmol), 5,5-dimethylcyclohexane-1,3-dione **3a** (2.0 mmol) in the presence or absence of catalyst(s) in different solvent system as well as neat conditions.

*NR* no reaction.

Significant values are in [bold].

^a^The progress of the reaction was scrutinized by TLC.

^b^Yields of the isolated products.

^c^Reaction was carried out in stirring or reflux conditions by using 3 mL of solvent.

^d^All the starting materials were ground in a mortar and pestle using different LAGs (*η* = 0.25 μL/mg) where [*η* = V (liquid; μL)/m (reagents; mg)].

^e^Sonication was performed using an ultrasonic bath (model: SB-3200DT) with an operating frequency of 40 kHz and nominal power of 180 W.

**Figure 8:**
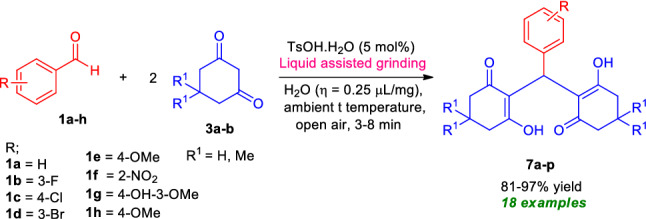
Synthesis of 2,2′-aryl/heteroaryl-methylene-bis(3-hydroxy-cyclohex-2-enone) under the standard conditions.

After ascertaining the optimal reaction conditions and to broadening the scopes of the reaction, a series of different types of aldehydes possessing electron-rich as well as electron-deficient substituents were treated with 5,5-dimethyl cyclohexane-1,3-dione **3a/**cyclohexane-1,3-dione **3b** (Fig. 8). The reaction condition was proven to be very efficient for all the substrates and as represented in Table 5, the respective 2,2′-aryl/heteroaryl-methylene-bis(3-hydroxy-cyclohex-2-enone) products **7a–p** were formed in good to excellent yields within 3–8 min without resulting in any side products. This operationally simple and highly beneficial approach efficiently tolerates different halogenated groups such as fluoro, chloro, bromo as well as other functional groups like nitro, hydroxyl present in the different positions of the aryl ring of aldehydes with both 5,5-dimethyl cyclohexane-1,3-dione **3a** or cyclohexane-1,3-dione **3b** and furnished the product **7b**, **7c**, **7d**, **7f**, **7g**, **7j**, **7k**, **7l**, **7m**, and **7n** in 98%, 96%, 93%, 95%, 94%, 95%, 93%, 87%, 96% and 95% yield respectively. Similarly, methoxy substituted aldehydes are well documented for this reaction. The reaction condition was found to be limited to aliphatic aldehydes.

**Table 5:**
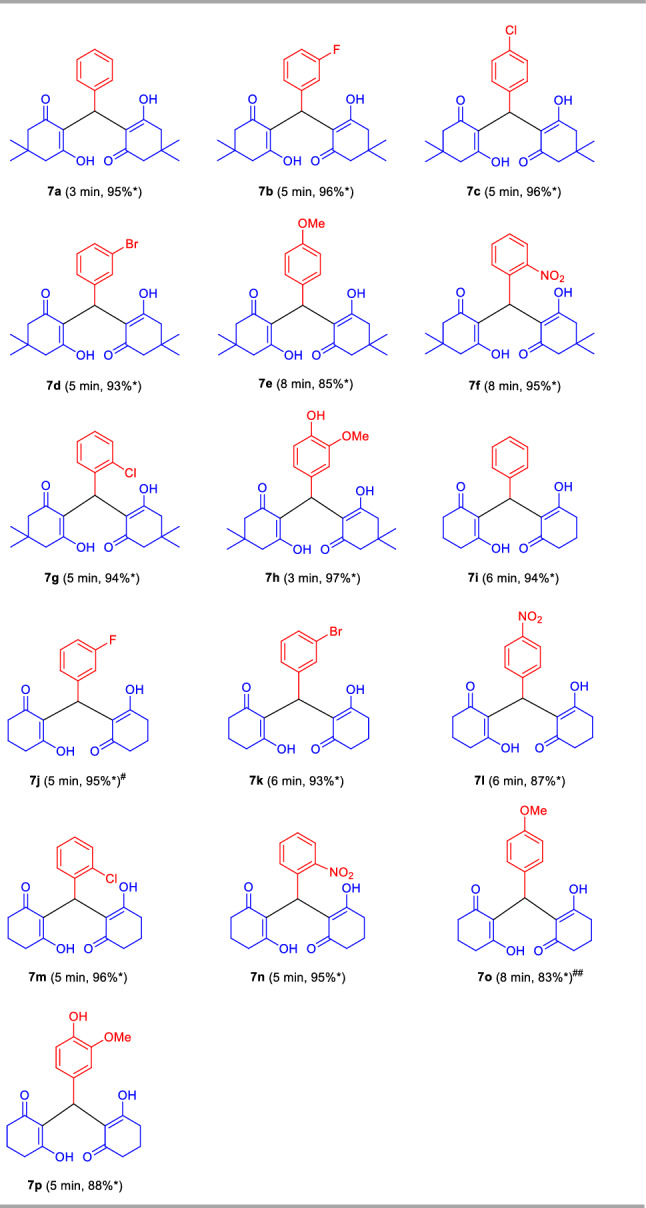
Substrate scopes for the synthesis of 2,2′-aryl/heteroaryl-methylene-bis(3-hydroxy-cyclohex-2-enone) derivatives **7a–p**.

Reaction condition: Aldehydes **1a–h** (1.0 mmol), and 5,5-dimethylcyclohexane-1,3-dione **3a**/cyclohexane-1,3-dione **3b** (2.0 mmol) in the presence of 5 mol% of TsOH⋅H_2_O in H_2_O (*η* = 0.25 μL/mg) as LAGs [*η* = V (liquid; μL)/m (reagents; mg)] at ambient temperature. *Isolated yield. ^#^7 mol% of TsOH⋅H_2_O was used. ^##^4 mol% of TsOH⋅H_2_O was used.

Xanthenes and their derivatives on the other hand recently gained tremendous interest in medicinal chemistry and material sciences due to their remarkable therapeutic potential and optoelectronic properties allowed by their attractive structural features^88,89^. They are commonly encountered in agricultural^90^, laser technology^91^ and found in diverse pharmacological applications^92–94^ such as antiestrogenic, antibacterial, antimicrobial as well as hypoglycaemic activities, neuropeptide YY5 receptor antagonist, etc.

Consequently, a vast array of catalytic systems such as sulfamic acid^95^, diethylamine^96^, 2,4,6-trichloro-1,3,5-triazine^97^, *p*-TSA^98^, and catalyst-free^99^ conditions have been discovered. Although the reported methodology offers several advantages, some of them suffer disadvantages like harsh reaction conditions, prolonged reaction time, high energy inputs, utilization of volatile organic solvents, non-recyclable catalytic system, low yield, and narrow substrate scopes.

**Figure 9:**
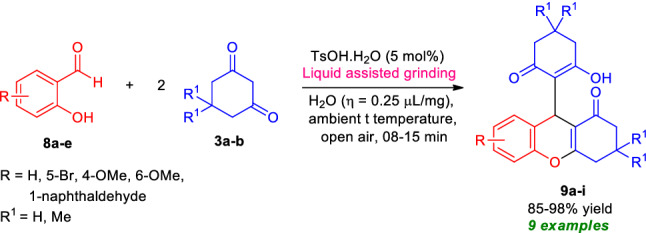
Synthesis of tetrahydro-1*H*-xanthen-1-one under the standard conditions.

Recognizing all these limitations and encouraged by our aforementioned results, as well as our ongoing interest in the multicomponent synthesis of medicinally privileged heterocycles^100–103^, we performed a domino three-component reaction between 1 equivalent of salicylaldehyde **8a** and 2 equivalent of 5,5-dimethyl cyclohexane-1,3-dione **3a** by employing water (*η* = 0.25 μL/mg) as the liquid assisted additives in presence of 5 mol% of TsOH⋅H_2_O as the Brønsted acid catalyst under mechanochemical grinding conditions. To our delight, the reaction afforded the corresponding 9-(2-hydroxy-4,4-dimethyl-6-oxocyclohex-1-en-1-yl)-3,3-dimethyl-2,3,4,9-tetrahydro-1*H*-xanthen-1-one, **9a** in 96% yield within 8 min. Enlightened by this successful result, we attempted a series of reactions between different derivatives of salicylaldehydes **8** and 5,5-dimethyl cyclohexane-1,3-dione **3a**/cyclohexane-1,3-dione **3b** under the standard conditions (Fig. 9, Table 6). Salicylaldehydes with halogenated substrates (R = Br) and electron releasing substrates (R = 4-OMe, 6-OMe) all were found to be very suitable for this reaction and furnished the desired products in excellent yield. On the other hand, heteroaryl substituted salicylaldehyde like 2-hydroxy naphthaldehyde also worked well in these reaction conditions (Table 6).

**Table 6:**
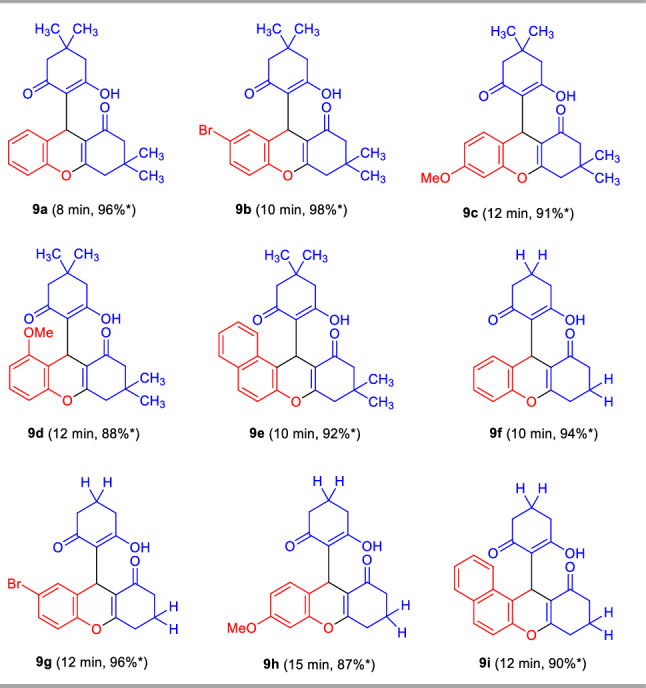
Substrate scopes for the synthesis of tetrahydro-1*H*-xanthen-1-one **9a–i**.

Reaction condition: Salicylaldehydes **8a–e** (1.0 mmol), and 5,5-dimethylcyclohexane-1,3-dione **3a**/cyclohexane-1,3-dione **3b** (2.0 mmol) in the presence of 5 mol% of TsOH⋅H_2_O in H_2_O (*η* = 0.25 μL/mg) as LAGs [*η* = V (liquid; μL)/m (reagents; mg)] at ambient temperature. *Isolated yield.

All of the synthesized compounds were isolated pure just by filtering off the precipitate along with continuous washing by water followed by recrystallization from ethanol. Notably, the approach does not necessitate the use of time-consuming column chromatographic techniques. The structure of the synthesized compounds has been determined based on spectroscopic analysis such as ^1^H NMR, ^13^C NMR, and HRMS (see supporting information).

**Figure 10:**
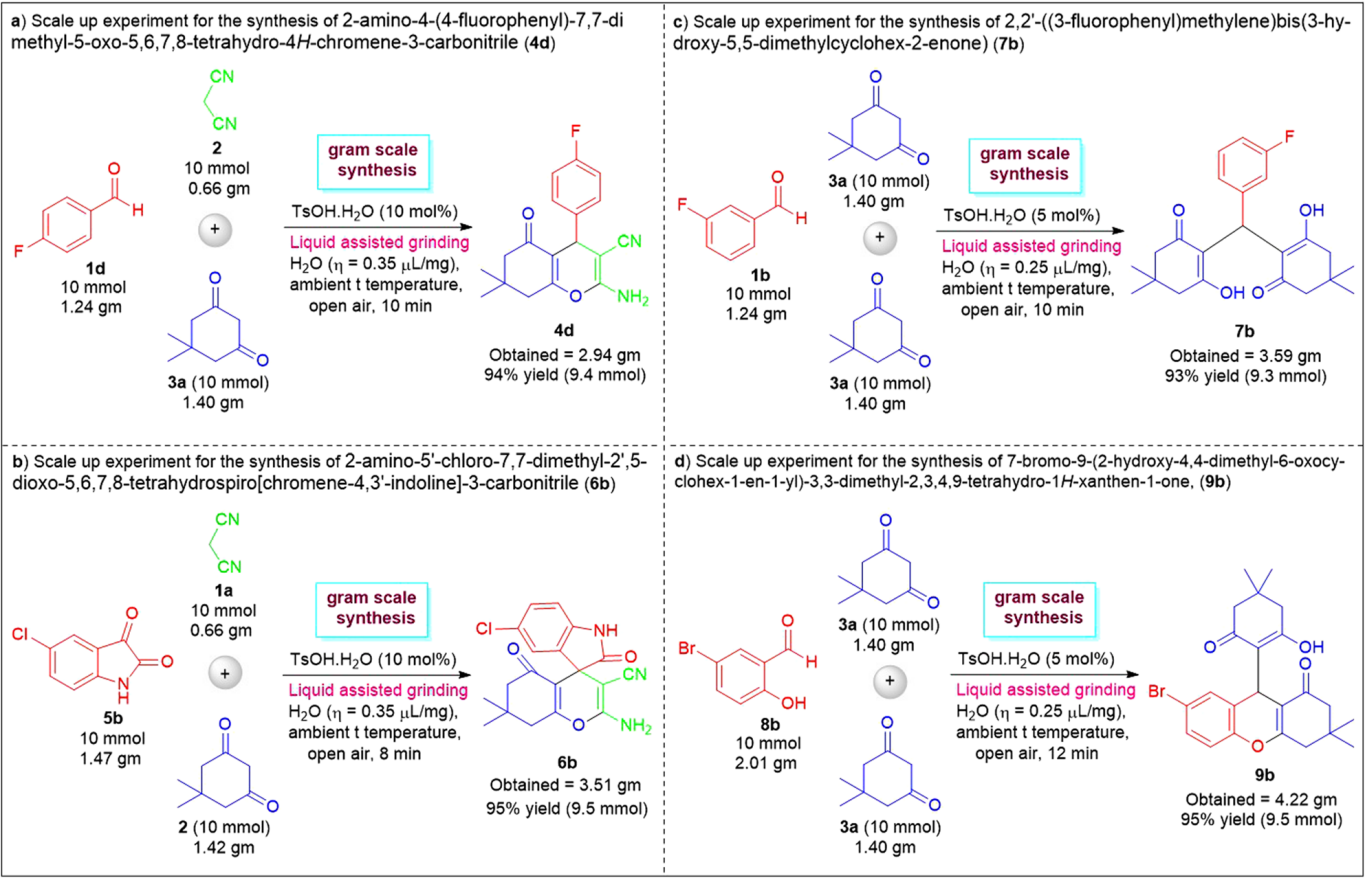
Preparative gram scale experiments for the synthesis of **4d**, **6b**, **7b**, and **9b**.

To further validate the potentiality of the present one-pot three-component domino work, three preparative scales up reaction for the synthesis of **4d**, **6b**, **7b**, and **9b** on a 10 mmol scale (Fig. 10). With the help of 10 mol% of TsOH⋅H_2_O, the mechanochemical water-assisted grinding of 4-flurobenzaldehyde **1d**, malononitrile **2**, and 5,5-dimethyl cyclohexane-1,3-dione **3a** smoothly afforded 2.94 g (94% yield) of the desired product **4d** after 10 min at ambient conditions (Fig. 10a). Similarly, the three-component reaction between 1.47 g of 5-chloro isatin, 0.66 g of malononitrile, and 1.42 g of 5,5-dimethylcyclohexane-1,3-dione **3a** efficiently proceeded at 8 min of grinding using 10 mol% of the TsOH⋅H_2_O in presence of water as LAGs to deliver 3.51 g of the product **6b** (95% yield) (Fig. 10b). Alternatively, we were also successful in the construction of 3.59 g of the product **7b** (93% yield) from1.24 g of 3-flurobenzaldehyde **1b**, and 2.8 g of **3a** (Fig. 10c). Similarly, by employing 5 mol% of TsOH⋅H_2_O with the aid of water-assisted mechanochemical grinding via a mortar and pestle, we synthesized the products **9b** in 4.22 g (95% yield) (Fig. 10d). Although all the four reactions took place in a slightly longer reaction time when using 10 mmol scales as compared to 1 mmol scales, the yield of the products was accomplished in the almost same quantity.

## Plausible mechanism

**Figure 11:**
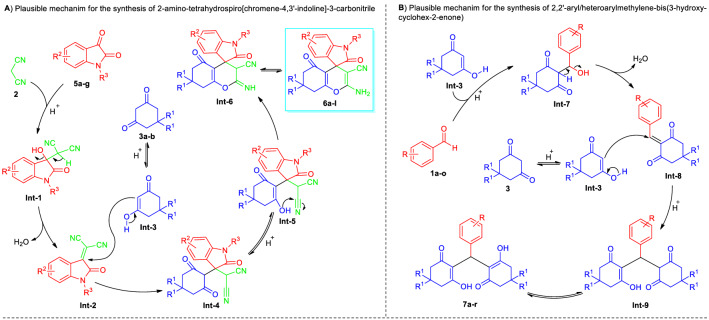
Plausible mechanism for the synthesis of 2-amino-tetrahydro-spiro[[chromene-4,3′-indoline]-3-carbonitrile **6** and 2,2′-aryl/heteroaryl-methylene-bis(3-hydroxy-cyclohex-2-enone) **7**.

The suggested mechanism for the formation of 2-amino-tetrahydro-spiro[chromene-3,4-indoline]-3-carbonitrile derivatives **6a–l** and 2,2′-aryl/heteroaryl-methylene-bis(3-hydroxy-cyclohex-2-enone) products **7a–p** were represented in Fig. 11A and B respectively. For products **6a–l**, an initial acid-catalyzed Knoevenagel condensation between substituted isatin **5a**-**g** with active methylene compound **2** took place smoothly to deliver the intermediate **Int-2** after the successful removal of water from **Int-1**. Besides, the keto-enol tautomerization of either 5,5-dimethyl cyclohexane-1,3-dione **3a** or cyclohexane-1,3-dione **3b** under the influences of the catalyst efficiently furnished the intermediate **Int-3** which eventually underwent Michael addition with intermediate **Int-2** to form the **Int-4**. The subsequent *6-exo-dig* cyclization of the –OH group onto the cyano moiety of **Int-5** delivers the imine intermediate **Int-6** that yields the final products **6a-l** after an imine-enamine tautomerization (Fig. 11A).

On the other hand, the mechanism for products **7a–r** proceeded through the formation of intermediate **Int-8** via an acid-catalyzed Knoevenagel condensation of substituted aldehydes **1a–o** with enol form **Int-3** of one molecule of 5,5-dimethyl cyclohexane-1,3-dione **3a** or cyclohexane-1,3-dione **3b**. This intermediate **Int-8** further experiences nucleophilic attack from the enol form **Int-3** of the second molecule of 5,5-dimethyl cyclohexane-1,3-dione **3a** or cyclohexane-1,3-dione **3b**, thereby delivering the intermediate **Int-9**. Consequently, the intermediate **Int-9** produced the respective product **7a–r** through the tautomerization reaction (Fig. 11B).

## Green chemistry metrics calculation

**Figure 12:**
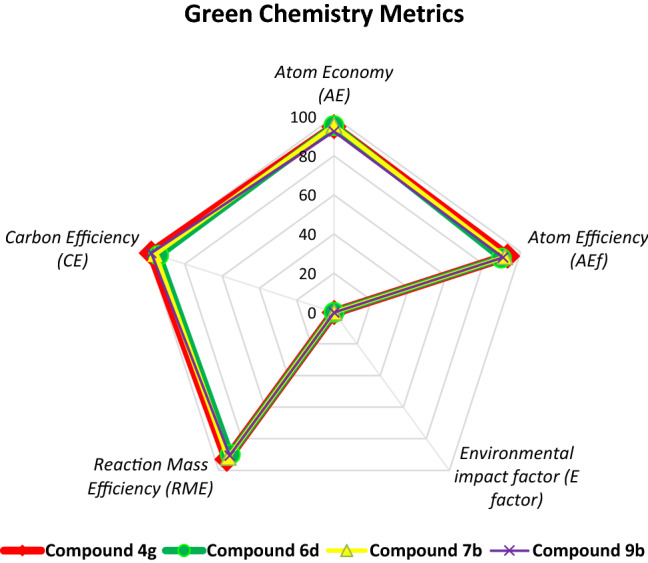
Radial pentagon diagram of green chemistry metrics calculation for the synthesis of **4g**, **6d, 7b**, and **9b** by Brønsted acid catalyzed water-assisted grinding via a mortar and pestle at ambient conditions.

The assessment of the greenness of different chemical processes is crucial in order to formulate the practical advancement of chemical synthesis and chemical processes in both industry and academics in the move toward more environmental sustainability^104^. The quantification of sustainable practices has to lead to the development of a series of metrics to support and reinforce the behavior change of chemical technology with the aim to address green or sustainable chemistry^105–109^. Most importantly used green metrics including Atom Economy (AE), atom efficiency (AEf), the environmental impact factor (*E*-factor), Reaction Mass Efficiency (RME), Carbon Efficiency (CE) are calculated for our newly developed methodology among which we examined the value of the metrics for compound **4g**, **6d 7b** and **9b** (see supporting information). The overall results are depicted in Table 7. From Table 7, it was observed that the calculated value of the atom economy and atom efficiency for compounds **4g**, **6d 7b**, and **9b** were much closer to the ideal value of AE and AEf, which indicates the presence of all starting material in the final product. The ideal value of the E-factor is considered zero for a chemical process and the calculated E-factor for compounds **4g**, **6d 7b**, and **9b** was found to be almost similar to the ideal value that points out the avoidance of waste products from the reaction. Similarly, the value of RME ranges from 0–100%, and the greater amount of RME provides the “cleanness” of a chemical reaction. The calculated value of RME for compounds **4g**, **6d 7b**, and **9b** resemble the ideal value which confirms the cleanness of the presented protocol. Also, the value of CE is matched with the calculated value for compounds **4g**, **6d**, **7b**, and **9b**. The graphical representation of the calculated data for various parameters is depicted in Fig. 12 in the form of a radial pentagon diagram which clearly showcases/supports the greenness or sustainability of the present approach (for further information see supporting information).

**Table 7:** Green metrics calculation for compounds **4g**, **6d**, **7b**, and **9b**.

Entry	Green Metrics	Ideal Value	Compound **4g**	Compound **6d**	Compound **7b**	Compound **9b**
1	Atom economy (AE) (%)	100	94.96	95.47	95.54	92.51
2	Atom efficiency (%)	100	93.06	89.74	91.71	90.66
3	E-factor	0	0.07	0.11	0.08	0.10
4	Reaction mass efficiency (RME) (%)	100	93	89.94	91.83	90.65
5	Carbon efficiency (CE) (%)	100	98	94	96	98

## Conclusions

In this study, we have disclosed an energy-efficient and environmentally sustainable route for the construction of a diverse set of 2-amino-3-cyano-4*H*-chromenes, tetrahydro- spiro[chromene-3,4′-indoline]-3-carbonitriles as well as 2,2′-aryl/heteroaryl-methylene-bis(3-hydroxy-cyclohex-2-enones) via water-assisted grinding induced Brønsted acid-catalyzed one-pot domino multicomponent reactions by employing the reactivity of 5,5-dimethyl-cyclohexane-1,3-dione/cyclohexane-1,3-dione at ambient conditions. With the help of low loading (only 5–10 mol%) of TsOH⋅H_2_O as the catalyst, all the synthesized products were achieved in good to excellent yields. The ability to accomplish the multiple C–C, C=C, C–O, C–N bond in a mechanochemical single pot operation from readily accessible materials without using any hazardous chemicals with water as green LAGs under metal-free conditions with great success in reducing the complex purification steps and cost of the overall process features the significant advantages of this approach. The practical effectiveness of the present method was established by demonstrating scale-up synthesis in almost quantitative yield with ultralow catalyst loading. The calculated green metrics for the present protocol were found to ideally resemble in all the cases, which supports the sustainability credentials of the current transformation. Other notable features of this environmentally benign, and highly atom economic procedure include short reaction time, broad functional group tolerances, operationally simple, mild setup procedure, cost-effective, energy-efficient, column chromatography free, metal-free, ligand-free, waste-free, toxic-free, high atom economy, atom efficiency, low E-factor, high reaction mass efficiency, open-air work, and easy isolation of products, which ensures the present approach an alternative sustainable route to the existing method.

## Experimental section

### General experimental detail

All commercially available chemicals were used without further purification. Thin Layer Chromatography (TLC) was executed utilizing silica gel 60 F254 (Merck) plates. Proton nuclear magnetic resonance spectra (^1^H NMR spectra) were obtained on Bruker 500 MHz, JEOL 400, and 600 MHz NMR spectrometers in CDCl_3_ and DMSO-*d*_*6*_ solvents. ^13^C NMR spectra were recorded on Bruker at 125 MHz, 100, and 150 MHz. Chemical shifts are reported in parts per million (ppm) relative to the TMS signal. Multiplicity is indicated as follows: s (singlet); bs (broad singlet); d (doublet); t (triplet); q (quartet); m (multiplet); dd (doublet of doublets), etc. TOF and quadrupole mass analyzer types are used for the HRMS measurements.

#### General Procedure for the synthesis of 2-amino-3-cyano-4***H***-chromene 4 and 2-amino-tetrahydrospiro[chromenes-3,4′-indoline]-3-carbonitriles 6

In a typical grinding method, carbonyl compounds such as aryl/heteroaryl aldehydes **1a–o** (1 mmol), or substituted isatin **5a–g** (1 mmol), malononitrile (1 mmol), and 5,5-dimethyl cyclohexane-1,3-dione/cyclohexane-1,3-dione **3a–b** (1 mmol) were mixed in a mortar and ground properly by a pestle in presence of water (*η* = 0.35 μL/mg) as LAGs with 10 mol% of TsOH⋅H_2_O as the catalyst at ambient temperature for the indicated time. The progress of the reaction was determined by TLC (thin layer chromatography). After complete consumption of starting material, as indicated by the TLC, the reaction mixture was transferred to a beaker and filtered off as well as washed with water, and then the crude product was washed with cold ethanol to give analytically pure products **4**, and **6**. All the compounds were fully characterized based on analytical data and detailed spectral studies including ^1^H NMR, ^13^C NMR, and HRMS (Supplementary information).

#### Spectral Data for the selected compounds 4a, 4b, 6a, and 6b

##### 2-amino-7,7-dimethyl-5-oxo-4-phenyl-5,6,7,8-tetrahydro-4*H*-chromene-3-carbonitrile, 4a

96% yield, white solid. R_*f*_ = 0.5 (60% EtOAc/Hexane). ^1^H NMR (500 MHz, CDCl_3_) δ 7.29 (t, *J* = 7.5 Hz, 2H), 7.24–7.19 (m, 3H), 4.52 (s, 2H), 4.41 (s, 1H), 2.46 (s, 2H), 2.23 (d, *J* = 8.0 Hz, 2H), 1.11 (s, 3H), 1.04 (s, 3H). ^13^C NMR (126 MHz, CDCl_3_ + DMSO-*d*_*6*_) δ 195.72, 162.31, 158.54, 144.54, 128.23, 127.28, 127.18, 126.59, 119.80, 113.06, 58.76, 50.25, 35.64, 35.61, 31.87, 28.66, 27.15, 27.10. HRMS (ESI^+^): m/z calculated for [C_18_H_18_N_2_O_2_ + H^+^]: 295.1447; found 295.1439.

##### 2-amino-7,7-dimethyl-5-oxo-4-(p-tolyl)-5,6,7,8-tetrahydro-4*H*-chromene-3-carbonitrile, 4b

88% yield, white solid. R_*f*_ = 0.6 (60% EtOAc/Hexane). ^1^H NMR (500 MHz, CDCl_3_) δ 7.10 (q, *J* = 7.9 Hz, 4H), 4.51 (s, 2H), 4.37 (s, 1H), 2.45 (s, 2H), 2.29 (s, 3H), 2.22 (d, *J* = 7.9 Hz, 2H), 1.11 (s, 3H), 1.04 (s, 3H). ^13^C NMR (126 MHz, CDCl_3_ + DMSO-*d*_*6*_) δ 195.59, 161.35, 157.77, 140.48, 135.78, 128.57, 126.81, 119.15, 113.38, 60.71, 50.16, 34.83, 34.66, 31.64, 28.39, 27.07, 20.54. HRMS (ESI^+^): m/z calculated for [C_19_H_20_N_2_O_2_ + H^+^]: 309.1603; found 309.1625.

##### 2-amino-7,7-dimethyl-2′,5-dioxo-5,6,7,8-tetrahydrospiro[chromene-4,3′-indoline]-3-carbonitrile, 6a

96% yield, white solid. R_*f*_ = 0.5 (80% EtOAc/Hexane). ^1^H NMR (600 MHz, CDCl_3_ + DMSO-*d*_*6*_) δ 10.20 (s, 1H), 7.15 (qd, *J* = 7.7, 1.4 Hz, 1H), 6.97–6.94 (m, 1H), 6.92 (dd, *J* = 13.4, 6.9 Hz, 1H), 6.86 (dd, *J* = 6.7, 6.2 Hz, 1H), 6.43 (t, *J* = 11.6 Hz, 2H), 2.53 (dd, *J* = 9.9, 5.1 Hz, 2H), 2.17 (ddd, *J* = 42.7, 16.2, 5.5 Hz, 2H), 1.12 (s, 3H), 1.07 (s, 3H). ^13^C NMR (126 MHz, DMSO-*d*_*6*_) δ 194.93, 178.08, 164.19, 158.81, 142.09, 134.45, 128.21, 123.05, 121.73, 117.39, 110.83, 109.28, 57.53, 50.04, 46.85, 40.43, 31.98, 27.65, 27.05. HRMS (ESI^+^): m/z calculated for [C_19_H_17_N_3_O_3_ + H^+^]: 336.1348; found 336.1318.

##### 2-amino-5′-chloro-7,7-dimethyl-2′,5-dioxo-5,6,7,8-tetrahydrospiro[chromene-4,3′-indoline]-3-carbonitrile, 6b

97% yield, white solid. R_*f*_ = 0.45 (80% EtOAc/Hexane). ^1^H NMR (600 MHz, CDCl_3_ + DMSO-*d*_*6*_) δ 7.78 (s, 1H), 7.12 (ddd, *J* = 8.2, 2.0, 0.9 Hz, 1H), 6.93 (d, *J* = 1.4 Hz, 1H), 6.83–6.80 (m, 1H), 6.69 (s, 2H), 2.54 (d, *J* = 9.1 Hz, 2H), 2.19 (s, 2H), 1.12 (s, 3H), 1.09 (s, 3H). ^13^C NMR (151 MHz, CDCl_3_ + DMSO-*d*_*6*_) δ 208.39, 194.27, 177.62, 163.74, 158.57, 140.42, 135.48, 127.68, 126.12, 122.81, 116.61, 110.29, 49.96, 46.88, 40.26, 31.63, 27.53, 27.32. HRMS (ESI^+^): m/z calculated for [C_19_H_16_ClN_3_O_3_ + H^+^]: 370.0958; found 370.0958.

## Supplementary Information

Supplementary Information.

## Data Availability

All data generated or analysed during this study are included in this published article [and its supplementary information files].
