# Supplementary material for: Brønsted acid catalyzed mechanochemical domino multicomponent reactions by employing liquid assisted grindstone chemistry
Source: Sci Rep. 2023 Jan 25;13:1386. doi: 10.1038/s41598-023-27948-y (PMC9876939; doi:10.1038/s41598-023-27948-y)
Supplement: Supplementary file 1 — Supplementary Information. [file 41598_2023_27948_MOESM1_ESM.pdf]

*Supporting Information*

# Brønsted acid catalyzed mechanochemical domino multicomponent reactions by employing liquid assisted grindstone chemistry

Biplob Borah,[a] Sidhartha Swain,[a] Mihir Patat,[a] Bhupender Kumar,[a] Ketan Kumar Prajapat,[a] Rathindranath Biswas,[b] Vasantha R,[a] and L. Raju Chowhan*[a]

[a] *School of Applied Material Sciences, Centre for Applied Chemistry, Sector-30, Central University of Gujarat, Gandhinagar-382030, India*
[b] *Department of Chemistry, Central University of Punjab, Bathinda 151401, India*

*** Correspondence:**
Corresponding Author
E-mail: rchowhan@cug.ac.in

***Table of content***


| Sl. No | Section | Page no |
|---|---|---|
| 1 | General | S1 |
| 2 | General Procedure for the synthesis of 2-amino-3-cyano-4*H*-chromene **4** and 2-amino-tetrahydrospiro[chromenes-3,4´-indoline]-3-carbonitriles **6** | S1-S2 |
| 3 | General procedure for the synthesis of 2,2'-aryl/heteroaryl-methylene-bis(3-hydroxy-cyclohex-2-enones) **7** | S2-S3 |
| 4 | General procedure for the synthesis of substituted 9-(2-hydroxy-6-oxocyclohex-1-en-1-yl)-2,3,4,9-tetrahydro-1*H*-xanthen-1-one **9** | S3 |
| 5 | Green Chemistry Metrics calculation | S4-S8 |
| 6 | Characterization data for the products (**4a-4t**), (**6a-6j**), (**7a-7p**) and (**9a-9i**) | S8-S35 |
| 7 | Copies of $^{1}$H and $^{13}$C NMR spectra for the products (**4a-4t**), (**6a-6j**), (**7a-9p**), and (**9a-9i**) (**Figure S5-S97**) | S36-S83 |
| 8 | Single point energy calculation of **Int-3-5** and **6a** in various solvents (**Figure S98**). | S84 |

## 1. General Experimental Detail

All commercially available chemicals were used without further purification. Thin Layer Chromatography (TLC) was executed utilizing silica gel 60 F254 (Merck) plates. Proton nuclear magnetic resonance spectra ($^{1}$H NMR spectra) were obtained on Bruker 500 MHz, JEOL 400 and 600 MHz NMR spectrometers in $CDCl_3$ and DMSO-$d_6$ solvents. $^{13}$C NMR spectra were recorded at 125 MHz, 100 and 150 MHz. Chemical shifts are reported in parts per million (ppm) relative to the TMS signal. Multiplicity is indicated as follows: s (singlet); bs (broad singlet); d (doublet); t (triplet); q (quartet); m (multiplet); dd (doublet of doublets), etc. TOF and quadrupole mass analyzer types are used for the HRMS measurements.

## 2. General Procedure for the synthesis of 2-amino-3-cyano-4*H*-chromene, (4a-t) and 2-amino-tetrahydrospiro[chromenes-3,4´-indoline]-3-carbonitriles, (6a-l):

In a typical grinding method, carbonyl compounds such as aryl/heteroaryl aldehydes **1a-o** (1 mmol), or substituted isatin **5a-g** (1 mmol), malononitrile (1 mmol), and 5,5-dimethylcyclohexane-1,3-dione/cyclohexane-1,3-dione **3a-b** (1 mmol) were mixed in a mortar and ground properly by a pestle in presence of water ($\eta$ = 0.35 μL/mg) as LAGs with 10 mol% of TsOH.$H_2O$ as the catalyst at ambient temperature for the indicated time. The progress of the reaction was determined by TLC (thin layer chromatography). After, complete consumption of starting material, as indicated by the TLC, the reaction mixture was transferred to a beaker and filtered off as well as washed with water and then the crude product was recrystallized from ethanol to give analytically pure products **4**, and **6** (**Figure S1**).

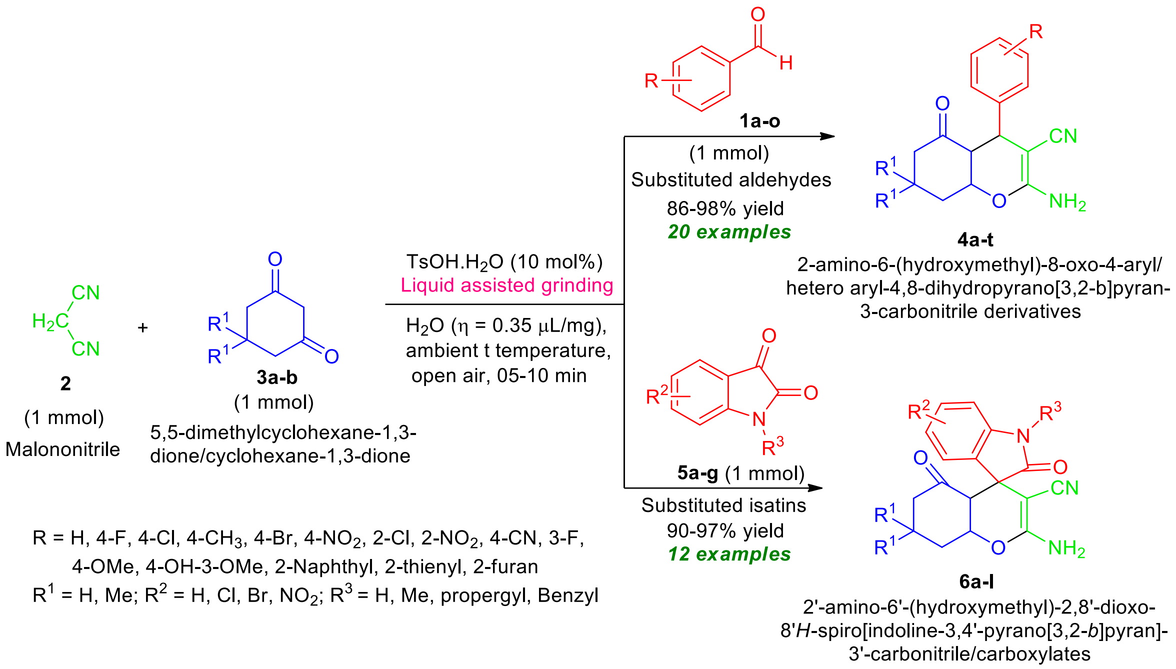


**Figure S1** Liquid-assisted grinding induced domino three-component reaction of aldehydes **1a-o**, or isatin **5a-g**, malononitrile **2**, and 5,5-dimethylcyclohexane-1,3-dione/cyclohexane-1,3-dione **3a-b** by using TsOH.$H_2O$ as the Brønsted acid catalyst

## 3. General procedure for the synthesis of 2,2'-aryl/heteroaryl-methylene-bis(3-hydroxy-cyclohex-2-enones), (7a-p):

A mixture of aryl/heteroaryl aldehydes **1a-o** (1 mmol), and 5,5-dimethylcyclohexane-1,3-dione **3a** (2 mmol)/cyclohexane-1,3-dione **3b** (2 mmol), and 5 mol% of TsOH.$H_2O$ in presence of liquid additive, ($H_2O$, $\eta$ = 0.20 μL/mg) was ground with the help of a mortar and pestle at ambient temperature for the required time. After the complete consumption of starting material as indicated by TLC, the residue was transferred to an oven-dried beaker and the solid was filtered off. The crude product was further washed with cold ethanol to afford analytically pure products **7** (**Figure S2**).

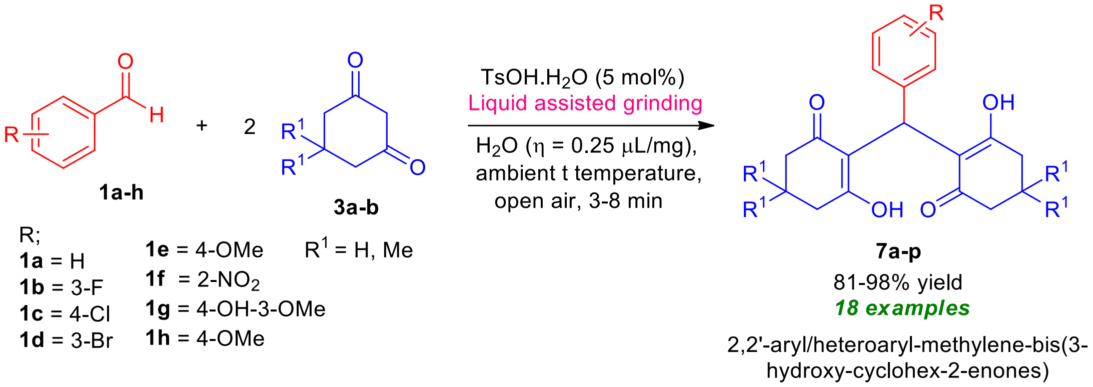


**Figure S2:** General procedure for the domino synthesis of 2,2'-aryl/heteroaryl-methylene-bis(3-hydroxy-cyclohex-2-enone) derivatives **7a-p** by using TsOH.$H_2O$ as the catalyst under water-assisted grinding conditions

## 4. General procedure for the synthesis of substituted 9-(2-hydroxy-6-oxocyclohex-1-en-1-yl)-2,3,4,9-tetrahydro-1*H*-xanthen-1-one, (9a-i):

In a typical grinding method, salicylaldehydes **8a-e** (1 mmol), and 5,5-dimethylcyclohexane-1,3-dione/cyclohexane-1,3-dione **3a-b** (2 mmol) were mixed in a mortar and ground properly by a pestle in presence of water ($\eta$ = 0.25 μL/mg) as liquid assisted grinding additives with 5 mol% of TsOH.$H_2O$ as the catalyst at ambient temperature for the indicated time. The progress of the reaction was determined by TLC (thin layer chromatography). After, complete consumption of starting material, as indicated by the TLC, the reaction mixture was transferred to a beaker and filtered off as well as washed with water, and then the crude product was recrystallized from ethanol to give analytically pure products **9a-i** (**Figure S3**).

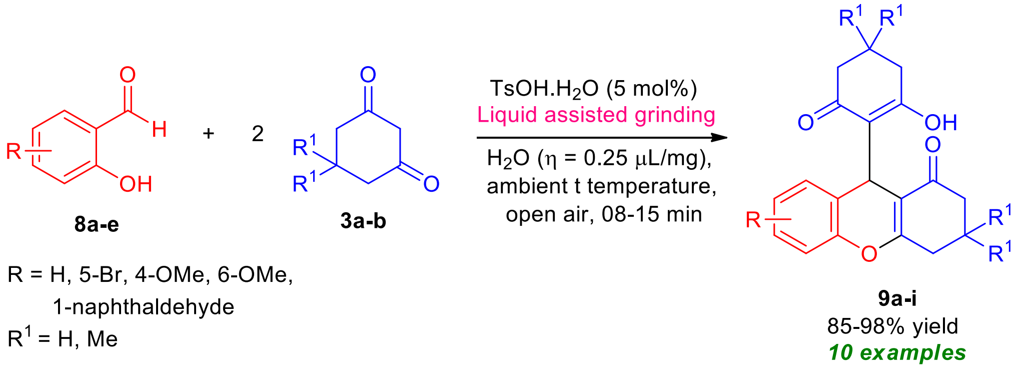


**Figure S3** General procedure for the synthesis of substituted 9-(2-hydroxy-4,4-dimethyl-6-oxocyclohex-1-en-1-yl)-3,3-dimethyl-2,3,4,9-tetrahydro-1*H*-xanthen-1-one (**9a-i**) by liquid assisted grinding induced organocatalytic domino reactions.

## 5. Green Chemistry Metrics calculation

Several green chemistry metrics such as Atom Economy (AE), atom efficiency, E-factor, Reaction Mass Efficiency (RME), Process Mass Intensity (PMI), Carbon Efficiency (CE) are calculated for the present study (**Table S1**). The schematic representation of the obtained value for the synthesis of compounds **4g** (**Figure S4a**), **6d** (**Figure S4b**), **7b** (**Figure S4c**), and **9b** (**Figure S4d**) to calculate the green chemistry metrics are presented below-

**a**) Reaction between 4-nitrobenzaldehyde, malononitrile, and 5,5-dimethylcyclohexane-1,3-dione under standard condition

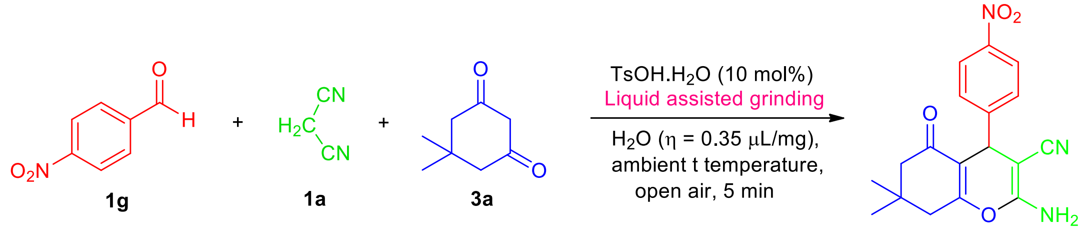


| | 1g | 1a | 3a | 4g |
|---|---|---|---|---|
| **FW** | 151.12 g/mol | 66.061 g/mol | 140.18 g/mol | 339.35 g/mol |
| **mmol** | 1 | 1 | 1 | 0.98 |
| **weight** | 0.151 gm | 0.066 gm | 0.140 gm | 0.332 gm |

**b**) Reaction between 5-nitroisatin, malononitrile, and 5,5-dimethylcyclohexane-1,3-dione under standard condition

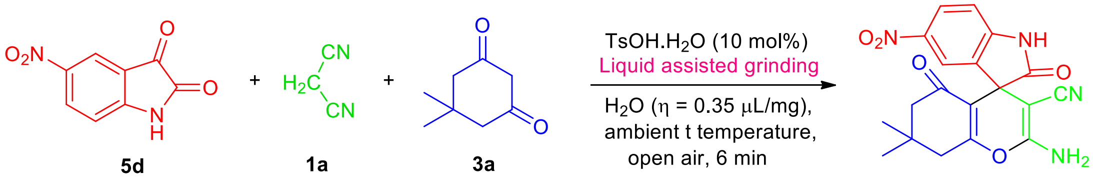


| | 5d | 1a | 3a | 6d |
|---|---|---|---|---|
| **FW** | 192.13 g/mol | 66.061 g/mol | 140.18 g/mol | 380.35 g/mol |
| **mmol** | 1 | 1 | 1 | 0.94 |
| **weight** | 0.192 gm | 0.066 gm | 0.140 gm | 0.358 gm |

**c**) Reaction between 1 equivalent of 4-fluorobenzaldehyde and 2 equivalent of 5,5-dimethylcyclohexane-1,3-dione

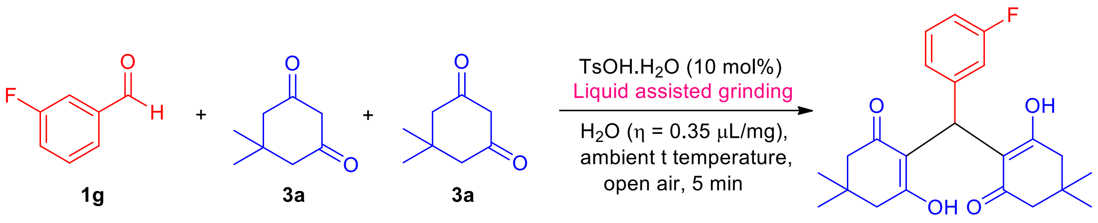


| | 1g | 3a | 3a | 7b |
|---|---|---|---|---|
| **FW** | 124.11 g/mol | 140.18 g/mol | 140.18 g/mol | 386.46 g/mol |
| **mmol** | 1 | 1 | 1 | 0.96 |
| **weight** | 0.124 gm | 0.140 gm | 0.140 gm | 0.371 gm |

**d)** Reaction between 1 equivalent of 5-bromosalicylaldehyde and 2 equivalent of 5,5-dimethylcyclohexane-1,3-dione

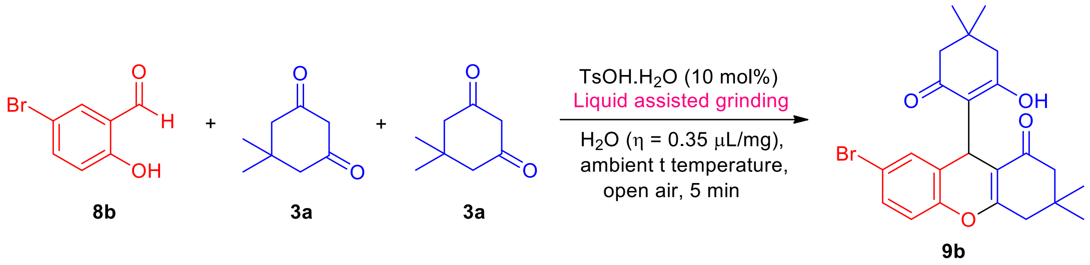


| | 8b | 3a | 3a | 9b |
|---|---|---|---|---|
| **FW** | 201.02 g/mol | 140.18 g/mol | 140.18 g/mol | 445.35 g/mol |
| **mmol** | 1 | 1 | 1 | 0.96 |
| **weight** | 0.201 gm | 0.140 gm | 0.140 gm | 0.436 gm |

**Figure S4:** Materials used in the synthetic purpose and the obtained value of their representative products

## <u>Calculated value:</u>

***Atom economy (AE):***

AE = Molecular Weight of product ÷ Σ (Molecular Weight of stoichiometric reactants) × 100%

For compound **4g**

AE = 339.35 g/mol ÷ (151.12 g/mol + 66.06 g/mol + 140.18 g/mol) × 100%

= 94.96%

For compound **6d**

AE = 380.35 g/mol ÷ (192.13 g/mol + 66.06 g/mol + 140.18 g/mol) × 100%

= 95.47%

For compound **7b**

AE = 386.46 g/mol ÷ (124.11 g/mol + 140.18 g/mol + 140.18 g/mol) × 100%

= 95.54%

For compound **9b**

AE = 445.35 g/mol ÷ (201.02 g/mol + 140.18 g/mol + 140.18 g/mol) × 100%

= 92.51%

***Atom efficiency (AEf):***

Atom efficiency = (Yield × AE)/100

For compound **4g**

AEf = (98% × 94.96%)/100 = 93.06%

For compound **6d**

AEf = (94% × 95.47%)/100 = 89.74%

For compound **7b**

AEf = (96% × 95.54%)/100 = 91.71%

For compound **9b**

AEf = (98% × 92.51%)/100 = 90.66%

***E-factor or environmental (E) factor:***

E-factor = [Total mass of wastes]/ Mass of product

Total mass of waste = Total mass of raw materials - Total mass of product

For compound **4g**

E-factor = [(0.151 g + 0.066 g + 0.140 g) - 0.332 g] / 0.332 g

= 0.07

For compound **6d**

E-factor = [(0.192 g + 0.066 g + 0.140 g) - 0.358 g] / 0.358 g

= 0.11

For compound **7b**

E-factor = [(0.124 g + 0.140 g + 0.140 g) - 0.371 g] / 0.371 g

= 0.08

For compound **9b**

E-factor = [(0.201 g + 0.140 g + 0.140 g) - 0.436 g] / 0.436 g

= 0.10

***Reaction mass efficiency (RME)***:

RME = Mass of isolated product / Σ (Mass of stoichiometric reactants) × 100%

For compound **4g**

RME = 0.332 g ÷ (0.151 g + 0.066 g + 0.140 g) × 100% = 93%

For compound **6d**

RME = 0.358 g ÷ (0.192 g + 0.066 g + 0.140 g) × 100% = 89.94%

For compound **7b**

= 0.371 g ÷ (0.124 g + 0.140 g + 0.140 g) × 100% = 91.83%

For compound **9b**

= 0.436 g ÷ (0.201 g + 0.140 g + 0.140 g) × 100% = 90.65%

***Process mass intensity (PMI):***

PMI = Σ (Mass of stoichiometric reactants + solvent) / Mass of product

For compound **4g**

PMI = (0.151 g + 0.066 g + 0.140 g + 0.018 g) ÷ 0.332 g

= 1.12

For compound **6d**

PMI = (0.161 g + 0.066 g + 0.140 g + 0.018 g) ÷ 0.358 g

= 1.11

For compound **7b**

PMI = (0.124 g + 0.140 g + 0.140 g + 0.018 g) ÷ 0.371 g

= 1.13

For compound **9b**

PMI = (0.201 g + 0.140 g + 0.140 g + 0.018 g) ÷ 0.436 g

= 1.14

***Carbon efficiency (CE):***

CE = (Amount of carbon in the product/Total carbon present in reactant) × 100%

For compound **4g**

CE = [0.98 × 18 / (1 × 7 + 1 × 3 + 1 × 8)] × 100% = 98%

For compound **6d**

CE = [0.94 × 19 / (1 × 8 + 1 × 3 + 1 × 8)] × 100% = 94%

For compound **7b**

CE = [0.96 × 23 / (1 × 7 + 1 × 8 + 1 × 8)] × 100% = 96%

For compound **9b**

CE = [0.98 × 23 / (1 × 7 + 1 × 8 + 1 × 8)] × 100% = 98%

**Table S1**: Green metrics calculation for compounds **4g**, **6d**, **7b**, and **9b**

| Entry | Green Metrics | Ideal Value | Compound **4g** | Compound **6d** | Compound **7b** | Compound **9b** |
|---|---|---|---|---|---|---|
| 1 | Atom economy (AE) (%) | 100 | 94.96 | 95.47 | 95.54 | 92.51% |
| 2 | Atom efficiency (%) | 100 | 93.06 | 89.74 | 91.71 | 90.66% |
| 3 | E-factor | 0 | 0.07 | 0.11 | 0.08 | 0.10 |
| 4 | Reaction mass efficiency (RME) (%) | 100 | 93 | 89.94 | 91.83 | 90.65% |
| 5 | Process Mass Intensity | | 1.12 | 1.11 | 1.13 | 1.14 |
| 5 | Carbon efficiency (CE) (%) | 100 | 98 | 94 | 96 | 98% |

## 6. Characterization data for the product (4a-4t), (6a-6l), (7a-7p), and (9a-9i):

**2-amino-7,7-dimethyl-5-oxo-4-phenyl-5,6,7,8-tetrahydro-4*H*-chromene-3-carbonitrile, 4a:**

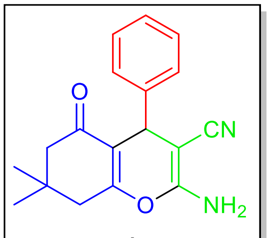


4a

96% yield, white solid. $R_f$ = 0.5 (60% EtOAc/Hexane). $^1$H NMR (500 MHz, $CDCl_3$) δ 7.29 (t, *J* = 7.5 Hz, 2H), 7.24 – 7.19 (m, 3H), 4.52 (s, 2H), 4.41 (s, 1H), 2.46 (s, 2H), 2.23 (d, *J* = 8.0 Hz, 2H), 1.11 (s, 3H), 1.04 (s, 3H). $^{13}$C NMR (126 MHz, $CDCl_3$ + DMSO-$d_6$) δ 195.72, 162.31, 158.54, 144.54, 128.23, 127.28, 127.18, 126.59, 119.80, 113.06, 58.76, 50.25, 35.64, 31.87, 28.66, 27.15, 27.10. HRMS (ESI$^+$): m/z calculated for [$C_{18}H_{18}N_2O_2$ + $H^+$]: 295.1447; found 295.1439

**2-amino-7,7-dimethyl-5-oxo-4-(p-tolyl)-5,6,7,8-tetrahydro-4*H*-chromene-3-carbonitrile, 4b:**

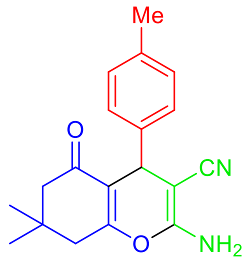


**4b**

88% yield, white solid. $R_f$ = 0.6 (60% EtOAc/Hexane). $^1$H NMR (500 MHz, $CDCl_3$) δ 7.10 (q, *J* = 7.9 Hz, 4H), 4.51 (s, 2H), 4.37 (s, 1H), 2.45 (s, 2H), 2.29 (s, 3H), 2.22 (d, *J* = 7.9 Hz, 2H), 1.11 (s, 3H), 1.04 (s, 3H). $^{13}$C NMR (126 MHz, $CDCl_3$+ DMSO-$d_6$) δ 195.59, 161.35, 157.77, 140.48, 135.78, 128.57, 126.81, 119.15, 113.38, 60.71, 50.16, 34.83, 34.66, 31.64, 28.39, 27.07, 20.54. HRMS (ESI$^+$): m/z calculated for [$C_{19}H_{20}N_2O_2$ + $H^+$]: 309.1603; found 309.1625

**2-amino-4-(4-methoxyphenyl)-7,7-dimethyl-5-oxo-5,6,7,8-tetrahydro-4*H*-chromene-3-carbonitrile, 4c:**

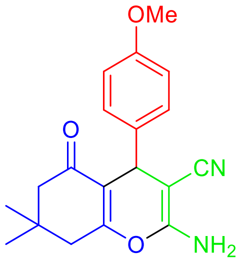


**4c**

86% yield, white solid. $R_f$ = 0.6 (60% EtOAc/Hexane). $^1$H NMR (500 MHz, $CDCl_3$ + DMSO-$d_6$) δ 7.15 (d, *J* = 8.3 Hz, 2H), 6.82 (d, *J* = 8.3 Hz, 2H), 4.51 (s, 2H), 4.36 (s, 1H), 3.77 (s, 3H), 2.44 (s, 2H), 2.22 (q, *J* = 16.3 Hz, 2H), 1.11 (s, 3H), 1.03 (s, 3H). $^{13}$C NMR (126 MHz, $CDCl_3$ + DMSO-$d_6$) δ 195.87, 161.50, 158.04, 157.91, 135.95, 128.23, 119.50, 113.50, 113.23, 60.35, 54.78, 50.27, 40.19, 34.46, 31.75, 28.54, 27.13. HRMS (ESI$^+$): m/z calculated for [$C_{19}H_{20}N_2O_3$ + $H^+$]: 325.1552; found 325.1532

**2-amino-4-(4-fluorophenyl)-7,7-dimethyl-5-oxo-5,6,7,8-tetrahydro-4*H*-chromene-3-carbonitrile, 4d:**

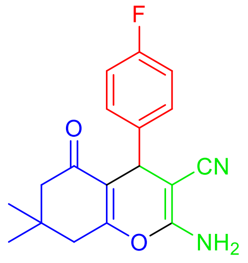


**4d**

98% yield, white solid. $R_f$ = 0.45 (60% EtOAc/Hexane). $^1$H NMR (500 MHz, $CDCl_3$+ DMSO-$d_6$) δ 7.24 – 7.17 (m, 2H), 6.97 (t, *J* = 8.5 Hz, 2H), 4.56 (s, 2H), 4.40 (s, 1H), 2.45 (s, 2H), 2.22 (q, *J* = 16.5 Hz, 2H), 1.11 (s, 3H), 1.03 (s, 3H). $^{13}$C NMR (126 MHz, $CDCl_3$+ DMSO-*d6*) δ 195.64, 162.13, 161.46, 160.18, 157.86, 139.23, 128.77, 128.71, 128.65, 118.95, 114.88, 114.75, 114.58, 113.27, 60.52, 50.15, 34.65, 34.48, 31.68, 28.36, 27.06. HRMS (ESI$^+$): m/z calculated for [$C_{18}H_{17}FN_2O_2$ + $H^+$]: 313.1352; found 313.1342

**2-amino-4-(4-chlorophenyl)-7,7-dimethyl-5-oxo-5,6,7,8-tetrahydro-4*H*-chromene-3-carbonitrile, 4e:**

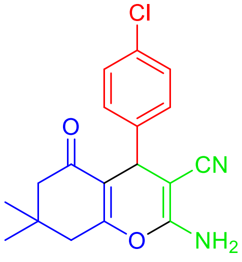


**4e**

96% yield, white solid. $R_f$ = 0.4 (60% EtOAc/Hexane). $^1$H NMR (600 MHz, DMSO-$d_6$) δ 7.36 – 7.32 (m), 7.17 (d, *J* = 8.3 Hz), 7.06 (s), 4.19 (s), 2.50 (s), 2.25 (d, *J* = 16.1 Hz), 2.11 (d, *J* = 16.1 Hz), 1.04 (s), 0.95 (s);$^{13}$C NMR (150 MHz, $CDCl_3$+ DMSO-$d_6$) δ 195.32, 161.52, 157.93, 142.08, 131.77, 128.51, 127.89, 118.82, 112.86, 59.67, 50.03, 40.08, 34.73, 31.54, 28.28, 26.96. HRMS (ESI$^+$): m/z calculated for [$C_{18}H_{17}ClN_2O_2$ + $H^+$]: 329.1057; found 329.1045

**2-amino-4-(4-bromophenyl)-7,7-dimethyl-5-oxo-5,6,7,8-tetrahydro-4*H*-chromene-3-carbonitrile, 4f:**

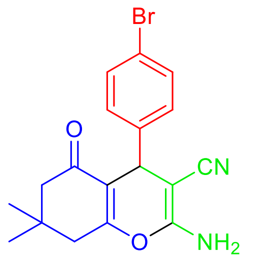


**4f**

94% yield, white solid. $R_f$ = 0.45 (60% EtOAc/Hexane). $^1$H NMR (600 MHz, DMSO-$d_6$) δ 7.48 (d, *J* = 8.3 Hz), 7.11 (d, *J* = 8.3 Hz), 7.05 (s), 4.18 (s), 2.51 (t, *J* = 6.1 Hz), 2.25 (d, *J* = 16.1 Hz), 2.10 (d, *J* = 16.1 Hz), 1.04 (s), 0.95 (s). $^{13}$NMR (151 MHz, $CDCl_3$ + DMSO-$d_6$) δ 195.73, 162.30, 158.54, 143.48, 131.05, 129.26, 119.94, 119.46, 112.76, 58.54, 50.25, 40.18, 35.19, 31.80, 28.54, 27.18. HRMS (ESI$^+$): m/z calculated for [$C_{18}H_{17}BrN_2O_2$ + $H^+$]: 373.0552; found 373.0536

**2-amino-7,7-dimethyl-4-(4-nitrophenyl)-5-oxo-5,6,7,8-tetrahydro-4*H*-chromene-3-carbonitrile, 4g:**

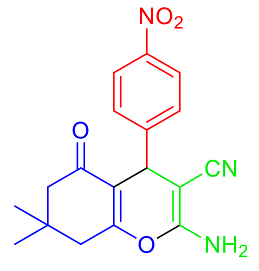


**4g**

98% yield, white solid. $R_f$ = 0.5 (60% EtOAc/Hexane). $^1$H NMR (500 MHz, $CDCl_3$ + DMSO-$d_6$) δ 8.14 (d, *J* = 8.5 Hz, 2H), 7.44 (d, *J* = 8.5 Hz, 2H), 6.39 (s, 2H), 4.46 (s, 1H), 2.52 (s, 2H), 2.34 – 2.03 (m, 2H), 1.12 (s, 3H), 1.03 (s, 3H). $^{13}$C NMR (126 MHz, $CDCl_3$+ DMSO-$d_6$) δ 95.72, 162.53, 158.48, 151.23, 146.28, 128.35, 123.39, 119.03, 112.27, 58.12, 50.09, 35.52, 31.80, 28.48, 27.13. HRMS (ESI+): m/z calculated for [$C_{18}H_{17}N_3O_4$ + $H^+$]: 340.1297; found 340.1279

**2-amino-4-(4-cyanophenyl)-7,7-dimethyl-5-oxo-5,6,7,8-tetrahydro-4*H*-chromene-3-carbonitrile, 4h:**

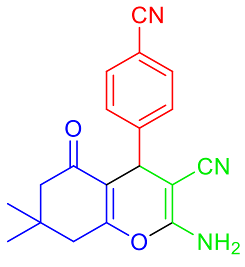

**4h**

90% yield, white solid. $R_f$ = 0.6 (60% EtOAc/Hexane). $^1$H NMR (600 MHz, DMSO-$d_6$) δ 7.76 – 7.66 (m), 7.35 – 7.25 (m), 7.10 (s), 4.25 (s), 2.49 (s), 2.21 (d, *J* = 16.0 Hz), 2.08 (d, *J* = 16.0 Hz), 1.00 (s), 0.91 (s). $^{13}$C NMR (150 MHz, $CDCl_3$ + DMSO-$d_6$) δ 195.26, 162.03, 158.15, 148.81, 131.69, 127.97, 118.61, 118.24, 112.24, 109.91, 58.60, 49.94, 40.06, 35.54, 31.56, 28.25, 26.98. HRMS (ESI$^+$): m/z calculated for [$C_{19}H_{17}N_3O_2$ + $H^+$]: 320.1399; found 320.1373

**2-amino-4-(3-hydroxy-4-methoxyphenyl)-7,7-dimethyl-5-oxo-5,6,7,8-tetrahydro-4*H*-chromene-3-carbonitrile, 4i:**

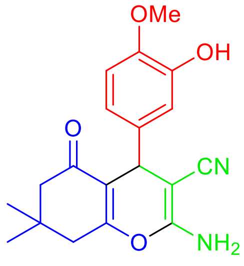

**4i**

92% yield, white solid. $R_f$ = 0.4 (60% EtOAc/Hexane). $^1$H NMR (500 MHz, $CDCl_3$+ DMSO-$d_6$) δ 7.84 (s, 1H), 6.79 – 6.70 (m, 2H), 6.58 (d, *J* = 7.9 Hz, 1H), 6.33 (s, 2H), 4.18 (s, 1H), 3.82 (s, 3H), 2.48 (d, *J* = 4.8 Hz, 2H), 2.20 (dd, *J* = 40.7, 16.2 Hz, 2H), 1.18 – 1.07 (m, 3H), 1.04 (s, 3H). $^{13}$C NMR (126 MHz, $CDCl_3$+ DMSO-$d_6$) δ 195.77, 161.78, 158.27, 147.08, 144.99, 135.58, 119.83, 119.36, 115.04, 113.39, 110.95, 59.49, 55.52, 50.30, 34.97, 31.76, 28.74, 26.98. HRMS (ESI$^+$): m/z calculated for [$C_{19}H_{20}N_2O_4$ + $H^+$]: 341.1501; found 341.1525

**2-amino-4-(3-fluorophenyl)-7,7-dimethyl-5-oxo-5,6,7,8-tetrahydro-4*H*-chromene-3-carbonitrile, 4j:**

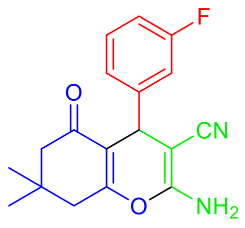

**4j**

96% yield, white solid. $R_f$ = 0.5 (60% EtOAc/Hexane). $^1$H NMR (500 MHz, $CDCl_3$+ DMSO-$d_6$) δ 7.28 – 7.22 (m, 1H), 7.04 (d, *J* = 7.5 Hz, 1H), 6.89 (dd, *J* = 20.0, 9.7 Hz, 2H), 5.58 (s, 2H), 4.37 (s, 1H), 2.47 (s, 2H), 2.22 (q, *J* = 16.4 Hz, 2H), 1.11 (s, 3H), 1.04 (s, 3H). $^{13}$C NMR (126 MHz, $CDCl_3$ + DMSO-$d_6$) δ 195.41, 163.14, 162.08, 161.19, 158.39, 146.80, 146.75, 129.59, 129.53, 122.76, 119.28, 114.11, 113.95, 113.83, 113.66, 113.38, 113.21, 113.09, 112.92, 112.62, 58.42, 50.08, 35.19, 31.67, 28.40, 27.07. HRMS ($ESI^+$): m/z calculated for [$C_{18}H_{17}FN_2O_2$ + $H^+$]: 313.1352; found 329.1339

**2-amino-7,7-dimethyl-4-(2-nitrophenyl)-5-oxo-5,6,7,8-tetrahydro-4*H*-chromene-3-carbonitrile, 4k:**

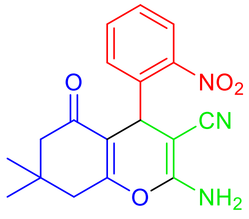

**4k**

94% yield, white solid. $R_f$ = 0.45 (60% EtOAc/Hexane). $^1$H NMR (500 MHz, $CDCl_3$+ DMSO-$d_6$) δ 7.80 (d, *J* = 7.9 Hz, 1H), 7.52 (t, *J* = 7.4 Hz, 1H), 7.33 (dd, *J* = 12.3, 7.9 Hz, 2H), 5.19 (s, 1H), 4.71 (s, 2H), 2.45 (s, 2H), 2.16 (q, *J* = 16.4 Hz, 2H), 1.09 (s, 3H), 0.99 (s, 3H). $^{13}$C NMR (126 MHz, $CDCl_3$+ DMSO-*d6*) δ 195.32, 161.57, 158.21, 149.13, 137.66, 132.21, 129.90, 127.23, 123.52, 118.13, 112.70, 59.42, 49.65, 31.66, 30.02, 28.27, 27.00. HRMS ($ESI^+$): m/z calculated for [$C_{18}H_{17}N_3O_4$ + $H^+$]: 340.1297; found 340.1278

**2-amino-4-(2-chlorophenyl)-7,7-dimethyl-5-oxo-5,6,7,8-tetrahydro-4*H*-chromene-3-carbonitrile, 4l:**

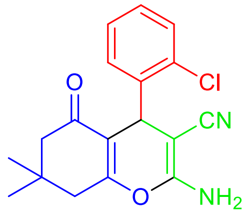


**4l**

95% yield, pale yellow solid. $R_f$ = 0.5 (60% EtOAc/Hexane). $^1$H NMR (500 MHz, $CDCl_3$) δ 7.33 (d, *J* = 7.8 Hz, 1H), 7.20 (d, *J* = 12.9 Hz, 2H), 7.15 (d, *J* = 6.3 Hz, 1H), 4.85 (s, 1H), 4.58 (s, 2H), 2.45 (s, 2H), 2.22 (d, *J* = 10.2 Hz, 2H), 1.12 (s, 3H), 1.07 (s, 3H). $^{13}$C NMR (126 MHz, $CDCl_3$+ DMSO-$d_6$) δ 195.55, 162.22, 158.02, 139.95, 132.66, 129.87, 129.58, 127.81, 126.61, 118.74, 112.19, 59.43, 50.11, 33.04, 31.66, 28.43, 27.18. HRMS ($ESI^+$): m/z calculated for [$C_{18}H_{17}ClN_2O_2$ + $H^+$]: 329.1057; found 329.1039

**2-amino-7,7-dimethyl-4-(naphthalen-2-yl)-5-oxo-5,6,7,8-tetrahydro-4*H*-chromene-3-carbonitrile, 4m:**

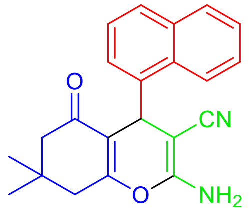


**4m**

90% yield, brown solid. $R_f$ = 0.45 (60% EtOAc/Hexane). $^1$H NMR (600 MHz, $CDCl_3$+ DMSO-$d_6$) δ 7.43 (dd, J = 8.0, 6.2 Hz), 7.15 – 7.09 (m), 6.98 – 6.93 (m), 6.91 – 6.83 (m), 5.66 (s), 4.72 (s), 2.44 (s, J = 17.6 Hz), 2.26 (d, J = 2.8 Hz), 1.11 (s, J = 10.9 Hz), 1.06 (s, J = 14.6 Hz). $^{13}$C NMR (150 MHz, $CDCl_3$+ DMSO-$d_6$) δ 195.28, 161.44, 161.41, 158.18, 158.15, 147.90, 147.87, 126.29, 126.26, 123.97, 123.94, 123.55, 123.52, 118.76, 113.31, 60.42, 50.02, 40.05, 31.52, 29.94, 28.46, 26.85. HRMS ($ESI^+$): m/z calculated for [$C_{22}H_{20}N_2O_2$ + $H^+$]: 345.1603; found 345.1589

**2-amino-7,7-dimethyl-5-oxo-4-(thiophen-2-yl)-5,6,7,8-tetrahydro-4*H*-chromene-3-carbonitrile, 4n:**

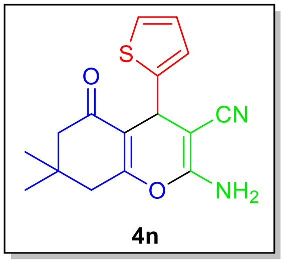


92% yield, white solid. $R_f$ = 0.45 (60% EtOAc/Hexane). $^1$H NMR (500 MHz, $CDCl_3$) δ 8.38 (d, *J* = 8.0 Hz, 1H), 7.27-7.25 (m, 1H), 7.83 (d, *J* = 8.2 Hz, 1H), 7.72 (d, *J* = 8.1 Hz, 1H), 7.57 (t, *J* = 7.5 Hz, 1H), 7.47 (t, *J* = 7.4 Hz, 1H), 7.39 (t, *J* = 7.7 Hz, 1H), 7.24 (d, *J* = 7.0 Hz, 1H), 5.27 (s, 1H), 4.52 (s, 2H), 2.57 – 2.48 (m, 2H), 2.20 (q, *J* = 16.4 Hz, 2H), 1.13 (s, 3H), 1.07 (s, 3H). $^{13}$C NMR (126 MHz, $CDCl_3$+ DMSO-$d_6$) δ 195.51, 161.76, 157.78, 133.26, 130.65, 127.07, 125.59, 125.15, 118.98, 113.89, 61.17, 50.08, 31.66, 28.42, 28.36, 27.25. HRMS (ESI$^+$): m/z calculated for [$C_{16}H_{16}N_2O_2S$ + $H^+$]: 301.1011; found 301.0991

**2-amino-4-(furan-2-yl)-7,7-dimethyl-5-oxo-5,6,7,8-tetrahydro-4*H*-chromene-3-carbonitrile, 4o:**

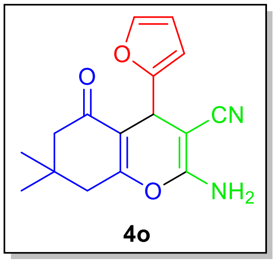


93% yield, white solid. $R_f$ = 0.5 (60% EtOAc/Hexane). $^1$H NMR (600 MHz, $CDCl_3$ + DMSO-$d_6$) δ 7.74 – 7.68 (m), 6.28 – 6.21 (m), 6.07 (t, J = 3.4 Hz), 4.45 (s, J = 3.4 Hz), 2.46 (s), 2.25 (d, J = 8.1 Hz), 1.11 (s, J = 8.6 Hz), 1.06 (s). $^{13}$C NMR (150 MHz, $CDCl_3$ + DMSO-$d_6$) δ 195.09, 162.42, 158.82, 154.83, 140.78, 119.00, 110.61, 109.85, 104.88, 56.76, 49.98, 40.08, 31.52, 28.62, 28.40, 26.68. HRMS (ESI$^+$): m/z calculated for [$C_{16}H_{16}N_2O_3$ + $H^+$]: 285.1239; found 285.1215

**2-amino-4-(2-nitrophenyl)-5-oxo-5,6,7,8-tetrahydro-4*H*-chromene-3-carbonitrile, 4p:**

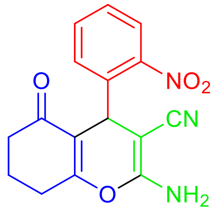


**4p**

92% yield, white solid. $R_f$ = 0.4 (60% EtOAc/Hexane). $^1H$ NMR (500 MHz, $CDCl_3$) δ 7.75 (dd, *J* = 8.4, 1.6 Hz, 1H), 7.52 (t, *J* = 7.3 Hz, 1H), 7.36 (d, *J* = 5.4 Hz, 1H), 7.34 – 7.31 (m, 1H), 5.58 (d, *J* = 15.9 Hz, 2H), 5.17 (s, 1H), 2.59 (d, *J* = 5.6 Hz, 2H), 2.29 (td, *J* = 5.8, 2.5 Hz, 2H), 2.07 – 1.91 (m, 2H). $^{13}C$ NMR (126 MHz, $CDCl_3$) δ 195.40, 189.01, 163.66, 158.65, 155.88, 148.91, 139.31, 138.45, 130.02, 113.68, 106.18, 56.48, 35.82, 29.83, 26.35, 19.51. HRMS ($ESI^+$): m/z calculated for [$C_{16}H_{13}N_3O_4 + H^+$]: 312.0984; found 312.0949

**2-amino-4-(4-fluorophenyl)-5-oxo-5,6,7,8-tetrahydro-4*H*-chromene-3-carbonitrile, 4q:**

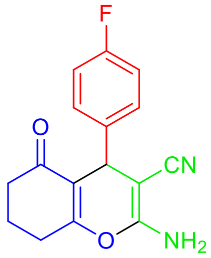


**4q**

93% yield, white solid. $R_f$ = 0.42 (60% EtOAc/Hexane). $^1H$ NMR (500 MHz, $CDCl_3$ + DMSO-$d_6$) δ 7.40 – 7.38 (m, 1H), 7.24 – 7.20 (m, 1H), 6.96 (ddd, *J* = 10.0, 6.0, 2.6 Hz, 2H), 5.51 (s, 2H), 4.39 (s, 1H), 2.60 – 2.58 (m, 2H), 2.42 – 2.29 (m, 2H), 2.10 – 1.94 (m, 2H). $^{13}C$ NMR (126 MHz, $CDCl_3$) δ 195.51, 163.38, 158.08, 155.90, 139.74, 139.72, 139.34, 128.68, 128.62, 114.59, 114.42, 114.17, 106.20, 59.13, 56.50, 36.22, 34.51, 26.50, 19.56. HRMS ($ESI^+$): m/z calculated for [$C_{16}H_{13}N_2FO_2 + H^+$]: 285.1039; found 285.1020

**2-amino-4-(3-fluorophenyl)-5-oxo-5,6,7,8-tetrahydro-4*H*-chromene-3-carbonitrile, 4r:**

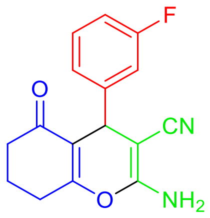


**4r**

97% yield, white solid. $R_f$ = 0.45 (60% EtOAc/Hexane). $^1$H NMR (600 MHz, $CDCl_3$ + DMSO-$d_6$) δ 7.23 (dd, *J* = 14.6, 7.1 Hz, 1H), 7.02 (d, *J* = 7.7 Hz, 1H), 6.91 – 6.84 (m, 2H), 6.15 (s, 2H), 4.33 (s, 1H), 2.66 – 2.54 (m, 2H), 2.38 – 2.29 (m, 2H), 2.09 – 1.93 (m, 2H). NMR (151 MHz, $CDCl_3$ + DMSO-$d_6$) δ 195.36, 163.54, 162.95, 161.32, 158.11, 146.33, 129.29, 122.73, 118.95, 113.82, 113.67, 113.11, 112.97, 58.98, 36.15, 34.89, 26.46, 19.50. HRMS (ESI$^+$): m/z calculated for [$C_{16}H_{13}N_2FO_2$ + $H^+$]: 285.1039; found 285.1029

**2-amino-4-(4-methoxyphenyl)-5-oxo-5,6,7,8-tetrahydro-4*H*-chromene-3-carbonitrile, 4s:**

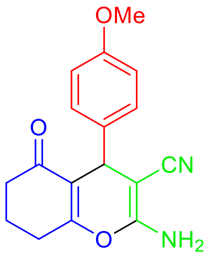


**4s**

86% yield, creamy solid. $R_f$ = 0.55 (60% EtOAc/Hexane). $^1$H NMR (600 MHz, $CDCl_3$ +) δ 7.15 – 7.11 (m, 2H), 6.81 – 6.77 (m, 2H), 6.18 (s, 2H), 4.28 (s, 1H), 3.76 (s, 3H), 2.58 (dt, *J* = 3.7, 1.7 Hz, 2H), 2.32 (dd, *J* = 7.9, 4.8 Hz, 2H), 2.10 – 1.93 (m, 2H). $^{13}$C NMR (126 MHz, $CDCl_3$ + DMSO-$d_6$) δ 195.50, 163.32, 158.08, 141.00, 139.34, 135.54, 128.53, 126.86, 119.41, 114.38, 106.21, 59.43, 36.29, 34.74, 26.52, 20.51, 19.63.. HRMS (ESI$^+$): m/z calculated for [$C_{17}H_{16}N_2O_3$ + $H^+$]: 297.1239; found 297.1222

**2-amino-5-oxo-4-(thiophen-2-yl)-5,6,7,8-tetrahydro-4*H*-chromene-3-carbonitrile, 4t:**

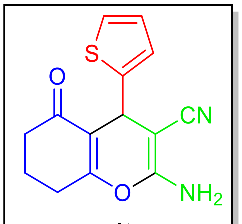

**4t**

94% yield, pale yellow solid. $R_f$ = 0.5 (60% EtOAc/Hexane). $^1$H NMR (500 MHz, $CDCl_3$) δ 7.39 – 7.36 (m, 1H), 7.13 – 7.08 (m, 2H), 5.37 (d, *J* = 9.0 Hz, 2H), 4.36 (s, 1H), 2.40 – 2.30 (m, 2H), 2.29 (s, 2H), 2.10 – 1.92 (m, 2H). HRMS ($ESI^+$): m/z calculated for [$C_{14}H_{12}N_2O_2S$ + $H^+$]: 273.0698; found 273.0676

**2-amino-7,7-dimethyl-2',5-dioxo-5,6,7,8-tetrahydrospiro[chromene-4,3'-indoline]-3-carbonitrile, 6a:**

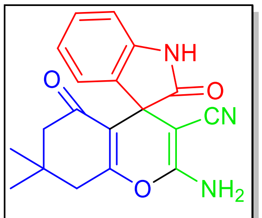

**6a**

96% yield, white solid. $R_f$ = 0.5 (80% EtOAc/Hexane). $^1$H NMR (600 MHz, $CDCl_3$ + DMSO-$d_6$) δ 10.20 (s, 1H), 7.15 (qd, *J* = 7.7, 1.4 Hz, 1H), 6.97 – 6.94 (m, 1H), 6.92 (dd, *J* = 13.4, 6.9 Hz, 1H), 6.86 (dd, *J* = 6.7, 6.2 Hz, 1H), 6.43 (t, *J* = 11.6 Hz, 2H), 2.53 (dd, *J* = 9.9, 5.1 Hz, 2H), 2.17 (ddd, *J* = 42.7, 16.2, 5.5 Hz, 2H), 1.12 (s, 3H), 1.07 (s, 3H). $^{13}$C NMR (126 MHz, DMSO-$d_6$) δ 194.93, 178.08, 164.19, 158.81, 142.09, 134.45, 128.21, 123.05, 121.73, 117.39, 110.83, 109.28, 57.53, 50.04, 46.85, 40.43, 31.98, 27.65, 27.05. HRMS ($ESI^+$): m/z calculated for [$C_{19}H_{17}N_3O_3$ + $H^+$]: 336.1348; found 336.1318

**2-amino-5'-chloro-7,7-dimethyl-2',5-dioxo-5,6,7,8-tetrahydrospiro[chromene-4,3'-indoline]-3-carbonitrile, 6b:**

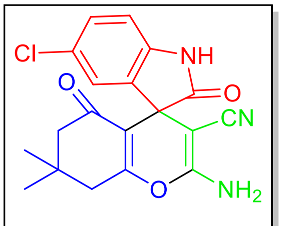


**6b**

97% yield, white solid. $R_f$ = 0.45 (80% EtOAc/Hexane). $^{1}$H NMR (600 MHz, $CDCl_3$ + DMSO-*d*$_6$) δ 7.78 (s, 1H), 7.12 (ddd, *J* = 8.2, 2.0, 0.9 Hz, 1H), 6.93 (d, *J* = 1.4 Hz, 1H), 6.83 – 6.80 (m, 1H), 6.69 (s, 2H), 2.54 (d, *J* = 9.1 Hz, 2H), 2.19 (s, 2H), 1.12 (s, 3H), 1.09 (s, 3H). $^{13}$C NMR (151 MHz, $CDCl_3$ + DMSO-*d*$_6$) δ 208.39, 194.27, 177.62, 163.74, 158.57, 140.42, 135.48, 127.68, 126.12, 122.81, 116.61, 110.29, 49.96, 46.88, 40.26, 31.63, 27.53, 27.32. HRMS (ESI$^+$): m/z calculated for [$C_{19}H_{16}ClN_3O_3$ + $H^+$]: 370.0958; found 370.0958

**2-amino-5'-bromo-7,7-dimethyl-2',5-dioxo-5,6,7,8-tetrahydrospiro[chromene-4,3'-indoline]-3-carbonitrile, 6c:**

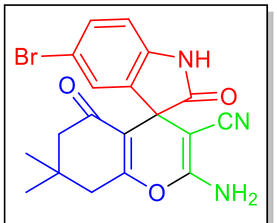


**6c**

95% yield, white solid. $R_f$ = 0.4 (80% EtOAc/Hexane). $^{1}$H NMR (600 MHz, $CDCl_3$ + DMSO-*d*$_6$) δ 10.54 (s, 1H), 7.33 (d, *J* = 2.1 Hz, 1H), 7.31 (d, *J* = 2.0 Hz, 2H), 7.21 (d, *J* = 2.0 Hz, 1H), 6.76 (d, *J* = 8.2 Hz, 1H), 2.54 (s, 2H), 2.16 (d, *J* = 7.7 Hz, 2H), 1.02 (s, 6H). $^{13}$C NMR (151 MHz, DMSO-*d*$_6$) δ 195.08, 177.65, 164.60, 158.87, 141.45, 136.79, 130.91, 125.94, 117.19, 113.30, 111.17, 110.18, 56.76, 49.94, 47.04, 31.97, 27.52, 27.16. HRMS (ESI$^+$): m/z calculated for [$C_{19}H_{16}BrN_3O_3$ + $H^+$]: 414.0453; found 414.0455

**2-amino-7,7-dimethyl-5'-nitro-2',5-dioxo-5,6,7,8-tetrahydrospiro[chromene-4,3'-indoline]-3-carbonitrile, 6d:**

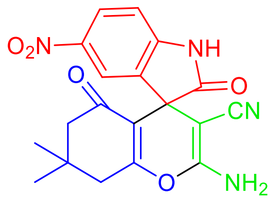

6d

94% yield, white solid. $R_f$ = 0.5 (80% EtOAc/Hexane). $^1$H NMR (600 MHz, $CDCl_3$ + DMSO-$d_6$) δ 10.99 (d, $J$ = 5.2 Hz, 1H), 8.13 (dd, $J$ = 8.6, 1.9 Hz, 1H), 7.85 (s, 1H), 7.00 (d, $J$ = 8.6 Hz, 1H), 6.70 (d, $J$ = 36.2 Hz, 2H), 3.08 (d, $J$ = 20.2 Hz, 2H), 2.20 (s, 2H), 1.13 (s, 3H), 1.10 (s, 3H). $^{13}$C NMR (151 MHz, $CDCl_3$ + DMSO-$d_6$) δ 194.54, 178.23, 164.29, 158.75, 158.73, 148.15, 142.36, 134.41, 125.16, 125.15, 118.42, 109.05, 49.85, 46.71, 40.29, 31.73, 27.48, 27.40. HRMS (ESI$^+$): m/z calculated for [$C_{19}H_{16}N_4O_5$ + $H^+$]: 381.1199; found 381.1192

**2-amino-1',7,7-trimethyl-2',5-dioxo-5,6,7,8-tetrahydrospiro[chromene-4,3'-indoline]-3-carbonitrile, 6e:**

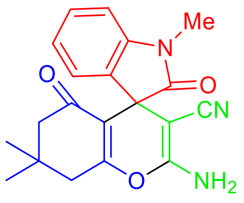

6e

90% yield, pale yellow solid. $R_f$ = 0.6 (80% EtOAc/Hexane). $^1$H NMR (600 MHz, $CDCl_3$ + DMSO-$d_6$) δ 7.31 – 7.24 (m, 1H), 7.07 – 6.98 (m, 2H), 6.88 (t, $J$ = 11.0 Hz, 1H), 6.50 (s, 2H), 3.25 (s, 3H), 2.58 (dt, $J$ = 3.7, 1.8 Hz, 1H), 2.54 (d, $J$ = 5.2 Hz, 1H), 2.15 (dd, $J$ = 38.2, 16.2 Hz, 2H), 1.12 (s, 3H), 1.07 (s, 3H). $^{13}$C NMR (151 MHz, $CDCl_3$ + DMSO-$d_6$) δ 194.31, 176.33, 163.47, 158.57, 143.10, 132.81, 122.35, 122.14, 110.92, 107.54, 49.95, 49.03, 46.25, 40.30, 31.61, 27.79, 27.04, 26.04. HRMS (ESI$^+$): m/z calculated for [$C_{20}H_{19}N_3O_3$ + $H^+$]: 350.1505; found 350.1492

**2-amino-1'-benzyl-7,7-dimethyl-2',5-dioxo-5,6,7,8-tetrahydrospiro[chromene-4,3'-indoline]-3-carbonitrile, 6f:**

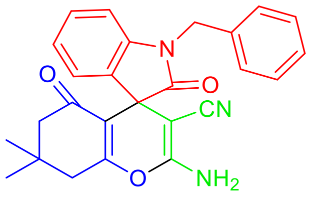


**6f**

93% yield, white solid. $R_f$ = 0.4 (80% EtOAc/Hexane). $^1$H NMR (600 MHz, $CDCl_3$ + DMSO-$d_6$) δ 7.48 (d, $J$ = 7.3 Hz, 2H), 7.32 (t, $J$ = 7.6 Hz, 2H), 7.25 (t, $J$ = 7.4 Hz, 1H), 7.12 – 7.09 (m, 1H), 7.03 (dd, $J$ = 7.4, 1.2 Hz, 1H), 6.97 (td, $J$ = 7.5, 0.7 Hz, 1H), 6.61 (d, $J$ = 7.8 Hz, 1H), 6.57 – 6.49 (m, 2H), 5.00 (d, $J$ = 11.4 Hz, 1H), 4.92 (d, $J$ = 16.1 Hz, 1H), 2.58 (dd, $J$ = 4.6, 1.3 Hz, 2H), 2.26 – 2.22 (m, 1H), 2.18 – 2.14 (m, 1H), 1.14 (s, 3H), 1.08 (s, 3H). $^{13}$C NMR (151 MHz, $CDCl_3$ + DMSO-$d_6$) δ 194.40, 176.54, 163.72, 158.62, 142.22, 135.20, 132.91, 128.08, 127.97, 126.77, 126.61, 122.48, 122.28, 110.89, 108.67, 49.99, 49.07, 46.40, 43.68, 40.41, 40.37, 31.66, 27.91, 27.02. HRMS (ESI$^+$): m/z calculated for [$C_{26}H_{23}N_3O_3$ + $H^+$]: 426.1818; found 426.1817

**2-amino-2',5-dioxo-5,6,7,8-tetrahydrospiro[chromene-4,3'-indoline]-3-carbonitrile, 6g:**

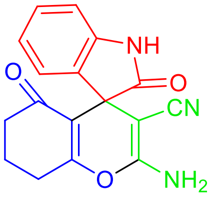


**6g**

94% yield, white solid. $R_f$ = 0.5 (80% EtOAc/Hexane). $^1$H NMR (600 MHz, $CDCl_3$ + DMSO-$d_6$) δ 10.25 (s, 1H), 7.13 (t, $J$ = 7.6 Hz, 1H), 6.97 (d, $J$ = 7.3 Hz, 1H), 6.91 (t, $J$ = 7.4 Hz, 1H), 6.83 (d, $J$ = 7.7 Hz, 1H), 6.60 (s, 2H), 2.70 – 2.63 (m, 2H), 2.30 – 2.24 (m, 2H), 2.10 – 1.96 (m, 2H). $^{13}$C NMR (151 MHz, $CDCl_3$ + DMSO-$d_6$) δ 194.21, 178.07, 165.16, 158.37, 141.56, 133.86, 127.75, 122.66, 121.35, 116.81, 112.06, 109.08, 58.18, 46.74, 36.20, 26.74, 19.49. HRMS (ESI$^+$): m/z calculated for [$C_{17}H_{13}N_3O_3$ + $H^+$]: 308.1035; found 308.1033

**2-amino-5'-nitro-2',5-dioxo-5,6,7,8-tetrahydrospiro[chromene-4,3'-indoline]-3-carbonitrile, 6h:**

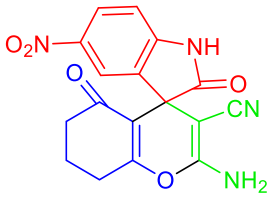


**6h**

96% yield, white solid. $R_f$ = 0.45 (80% EtOAc/Hexane). $^1$H NMR (600 MHz, $CDCl_3$ + DMSO-$d_6$) δ 11.01 (s, 1H), 8.15 – 8.10 (m, 1H), 7.87 (d, $J$ = 2.1 Hz, 1H), 7.71 – 7.66 (m, 1H), 6.99 (d, $J$ = 8.6 Hz, 1H), 6.71 (d, $J$ = 11.5 Hz, 2H), 2.76 – 2.68 (m, 2H), 2.33 (t, $J$ = 6.7 Hz, 2H), 2.12 – 2.04 (m, 2H). HRMS (ESI$^+$): m/z calculated for [$C_{17}H_{12}N_4O_5$ + $H^+$]: 353.0886; found 353.0886

**2-amino-5'-bromo-2',5-dioxo-5,6,7,8-tetrahydrospiro[chromene-4,3'-indoline]-3-carbonitrile, 6i:**

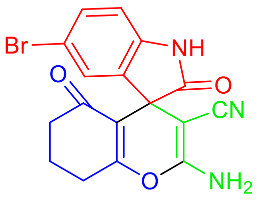


**6i**

96% yield, white solid. $R_f$ = 0.42 (80% EtOAc/Hexane). $^1$H NMR (600 MHz, $CDCl_3$ + DMSO-$d_6$) δ 10.49 (s, 1H), 7.28 – 7.25 (m, 1H), 7.10 (d, $J$ = 0.9 Hz, 1H), 6.97 (s, 2H), 6.77 (d, $J$ = 8.2 Hz, 1H), 2.68 (dd, $J$ = 13.3, 6.4 Hz, 2H), 2.30 (t, $J$ = 6.7 Hz, 2H), 2.04 (dd, $J$ = 12.7, 6.3 Hz, 2H). $^{13}$C NMR (151 MHz, $CDCl_3$ + DMSO-$d_6$) δ 194.44, 177.62, 165.76, 158.51, 140.99, 136.27, 130.49, 125.71, 116.76, 113.37, 111.47, 110.78, 57.10, 46.97, 36.14, 26.74, 19.48. HRMS (ESI$^+$): m/z calculated for [$C_{17}H_{12}BrN_3O_3$ + $H^+$]: 386.0140; found 386.0138

**2-amino-1'-methyl-2',5-dioxo-5,6,7,8-tetrahydrospiro[chromene-4,3'-indoline]-3-carbonitrile, 6j:**

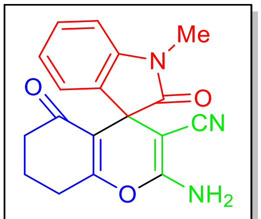

6j

93% yield, pale yellow solid. $R_f$ = 0.6 (80% EtOAc/Hexane). $^1$H NMR (600 MHz, $CDCl_3$ + DMSO-$d_6$) δ 7.29 – 7.21 (m, 1H), 7.05 – 6.98 (m, 2H), 6.87 (d, *J* = 8.4 Hz, 1H), 6.47 (d, *J* = 4.7 Hz, 2H), 2.67 (t, *J* = 6.7 Hz, 3), 2.62 – 2.61 (m, 2), 2.58 (dt, *J* = 3.9, 1.9 Hz, 2H), 2.31 – 2.26 (m, 2H). $^{13}$C NMR (151 MHz, $CDCl_3$ + DMSO-$d_6$) δ 194.41, 176.45, 165.25, 158.43, 143.06, 132.89, 128.11, 122.49, 122.13, 116.62, 112.09, 107.48, 46.34, 40.42, 36.12, 26.74, 26.04, 19.44. HRMS (ESI$^+$): m/z calculated for [$C_{18}H_{15}N_3O_3$ + $H^+$]: 322.1192; found 322.1204

**2-amino-2',5-dioxo-1'-(prop-2-yn-1-yl)-5,6,7,8-tetrahydrospiro[chromene-4,3'-indoline]-3-carbonitrile, 6k:**

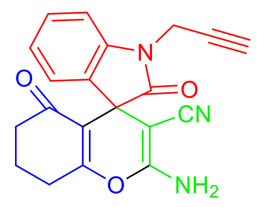

6k

94% yield, white solid. $R_f$ = 0.55 (80% EtOAc/Hexane). $^1$H NMR (600 MHz, $CDCl_3$ + DMSO-$d_6$) δ 7.69 (ddd, *J* = 14.9, 6.9, 4.8 Hz, 1H), 7.30 – 7.25 (m, 1H), 7.05 (ddd, *J* = 9.4, 8.4, 5.3 Hz, 2H), 6.51 (d, *J* = 37.6 Hz, 2H), 4.59 – 4.49 (m, 2H), 2.70 – 2.66 (m, 2H), 2.62 (d, *J* = 0.5 Hz, 1H), 2.32 – 2.27 (m, 2H), 2.10 – 1.96 (m, 2H). HRMS (ESI$^+$): m/z calculated for [$C_{20}H_{15}N_3O_3$ + $H^+$]: 346.1192; found 370.1202

**2-amino-1'-benzyl-2',5-dioxo-5,6,7,8-tetrahydrospiro[chromene-4,3'-indoline]-3-carbonitrile, 6l:**

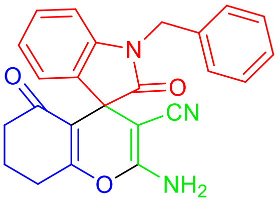

**6l**

93% yield, white solid. $R_f$ = 0.6 (80% EtOAc/Hexane). $^1H$ NMR (600 MHz, $CDCl_3$ + DMSO-$d_6$) δ 7.45 (d, *J* = 7.3 Hz, 2H), 7.29 – 7.25 (m, 4H), 7.24 – 7.19 (m, 1H), 7.10 – 7.06 (m, 2H), 6.93 – 6.90 (m, 1H), 6.64 (d, *J* = 7.8 Hz, 1H), 4.92 – 4.82 (m, 2H), 2.66 (dd, *J* = 10.7, 6.1 Hz, 2H), 2.28 – 2.17 (m, 2H), 1.94 – 1.88 (m, 2H). $^{13}C$ NMR (151 MHz, $CDCl_3$ + DMSO-$d_6$) δ 195.11, 176.77, 166.36, 158.77, 142.56, 136.17, 133.70, 128.36, 128.35, 128.34, 128.32, 128.29, 128.28, 128.18, 127.11, 127.09, 127.07, 127.06, 127.05, 123.08, 122.46, 117.37, 111.72, 108.74, 57.27, 46.63, 43.31, 36.29, 26.77, 19.74. HRMS ($ESI^+$): m/z calculated for $[C_{24}H_{19}N_3O_3 + H^+]$: 398.1505; found 398.1483

**2,2'-(phenylmethylene)bis(3-hydroxy-5,5-dimethylcyclohex-2-enone), 7a:**

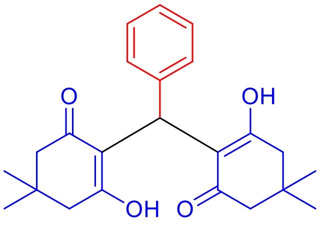

**7a**

95% yield, white solid. $R_f$ = 0.4 (40% EtOAc/Hexane). $^1H$ NMR (500 MHz, $CDCl_3$) δ 11.92 (s, 1H), 7.29 – 7.25 (m, 3H), 7.19 – 7.15 (m, 1H), 7.11 – 7.08 (m, 2H), 5.54 (s, 1H), 2.39 (dq, *J* = 33.1, 17.6 Hz, 9H), 1.24 (s, 7H), 1.10 (s, 6H). HRMS ($ESI^+$): m/z calculated for $[C_{23}H_{28}O_4 + H^+]$: 369.2065; found 369.2055

**2,2'-((3-fluorophenyl)methylene)bis(3-hydroxy-5,5-dimethylcyclohex-2-enone), 7b:**

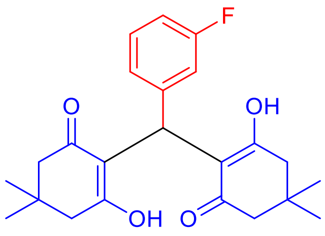


**7b**

96% yield, white solid. $R_f$ = 0.35 (40% EtOAc/Hexane). $^{1}$H NMR (500 MHz, $CDCl_3$) δ 11.92 (s, 1H), 7.22 (td, *J* = 8.0, 6.3 Hz, 1H), 6.87 (qd, *J* = 7.2, 3.8 Hz, 2H), 6.78 (d, *J* = 10.9 Hz, 1H), 5.50 (s, 1H), 2.49 – 2.29 (m, 8H), 1.23 (s, 6H), 1.10 (s, 6H). HRMS (ESI$^{+}$): m/z calculated for $[C_{23}H_{27}FO_4 + H^+]$: 387.1972; found 387.1959

**2,2'-((4-chlorophenyl)methylene)bis(3-hydroxy-5,5-dimethylcyclohex-2-enone), 7c:**

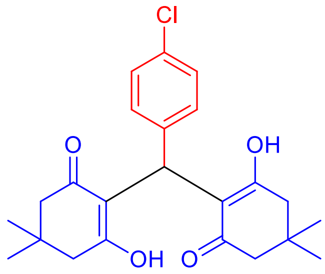


**7c**

96% yield, white solid. $R_f$ = 0.4 (40% EtOAc/Hexane). $^{1}$H NMR (500 MHz, $CDCl_3$) δ 11.88 (s, 1H), 7.25 – 7.21 (m, 2H), 7.01 (dd, *J* = 8.6, 1.1 Hz, 2H), 5.47 (s, 1H), 2.39 (ddd, *J* = 41.8, 32.1, 17.6 Hz, 8H), 1.22 (s, 6H), 1.10 (s, 6H). HRMS (ESI$^{+}$): m/z calculated for $[C_{23}H_{27}ClO_4 + H^+]$: 403.1676; found 403.1653

**2,2'-((3-bromophenyl)methylene)bis(3-hydroxy-5,5-dimethylcyclohex-2-enone), 7d:**

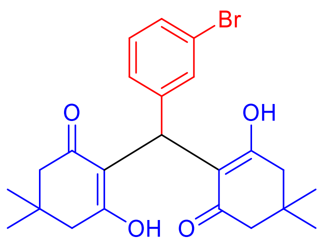


**7d**

93% yield, white solid. $R_f$ = 0.42 (40% EtOAc/Hexane). $^1$H NMR (500 MHz, $CDCl_3$) δ 11.91 (s, 1H), 7.32 – 7.29 (m, 1H), 7.23 (d, *J* = 1.3 Hz, 1H), 7.14 (t, *J* = 7.9 Hz, 1H), 7.02 (d, *J* = 7.9 Hz, 1H), 5.49 (s, 1H), 2.49 – 2.29 (m, 8H), 1.23 (s, 6H), 1.10 (s, 6H). HRMS (ESI$^+$): m/z calculated for [$C_{23}H_{27}BrO_4$ + $H^+$]: 447.1171; found 447.1159

**2,2'-((4-methoxyphenyl)methylene)bis(3-hydroxy-5,5-dimethylcyclohex-2-enone), 7e:**

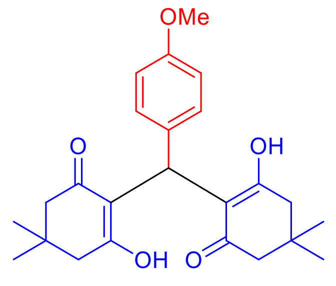

7e

85% yield, white solid. $R_f$ = 0.5 (40% EtOAc/Hexane). $^1$H NMR (500 MHz, $CDCl_3$) δ 11.92 (s, 1H), 7.00 (dd, *J* = 8.8, 0.9 Hz, 2H), 6.83 – 6.79 (m, 2H), 5.48 (s, 1H), 3.77 (s, 3H), 2.38 (dq, *J* = 31.7, 17.6 Hz, 8H), 1.23 (s, 6H), 1.10 (s, 6H). $^{13}$C NMR (126 MHz, CDCl3) δ 190.47, 189.43, 157.66, 129.90, 127.87, 115.85, 113.71, 55.26, 47.14, 46.50, 32.10, 31.46, 29.74, 27.45. HRMS (ESI$^+$): m/z calculated for [$C_{24}H_{30}O_5$ + $H^+$]: 399.2171; found 399.2149

**2,2'-((2-nitrophenyl)methylene)bis(3-hydroxy-5,5-dimethylcyclohex-2-enone), 7f:**

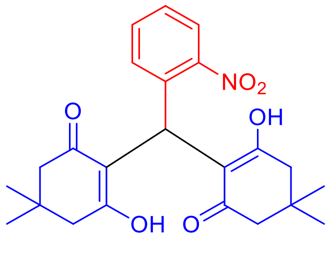

7f

95% yield, white solid. $R_f$ = 0.45 (40% EtOAc/Hexane). $^1$H NMR (500 MHz, $CDCl_3$) δ 11.60 (s, 1H), 7.55 (dd, *J* = 7.9, 1.3 Hz, 1H), 7.48 (td, *J* = 7.7, 1.4 Hz, 1H), 7.33 (t, *J* = 7.7 Hz, 1H), 7.25 (d, *J* = 8.0 Hz, 1H), 6.04 (s, 1H), 2.47 (dd, *J* = 33.4, 17.1 Hz, 4H), 2.32 (d, *J* = 15.2 Hz, 2H), 2.25 – 2.19 (m, 2H), 1.16 (s, 6H), 1.02 (s, 6H). HRMS (ESI$^+$): m/z calculated for [$C_{23}H_{27}NO_6$ + $H^+$]: 414.1917; found 414.1889

**2,2'-((2-chlorophenyl)methylene)bis(3-hydroxy-5,5-dimethylcyclohex-2-enone), 7g:**

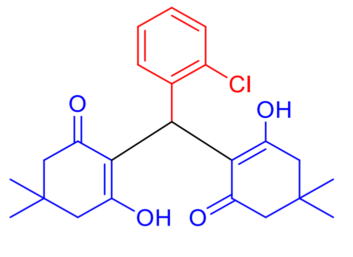


**7g**

94% yield, white solid. $R_f = 0.42$ (40% EtOAc/Hexane). $^1$H NMR (500 MHz, $CDCl_3$) δ 11.90 (s, 1H), 7.38 (d, *J* = 7.7 Hz, 1H), 7.30 (dd, *J* = 7.8, 1.4 Hz, 1H), 7.21 (td, *J* = 7.7, 1.4 Hz, 1H), 7.15 (td, *J* = 7.7, 1.5 Hz, 1H), 5.62 (s, 1H), 2.43 (t, *J* = 12.9 Hz, 5H), 2.37 – 2.31 (m, 3H), 2.27 (d, *J* = 16.8 Hz, 2H). HRMS ($ESI^+$): m/z calculated for $[C_{23}H_{27}ClO_4 + H^+]$: 403.1676; found 403.1649

**2,2'-((4-hydroxy-3-methoxyphenyl)methylene)bis(3-hydroxy-5,5-dimethylcyclohex-2-enone), 7h:**

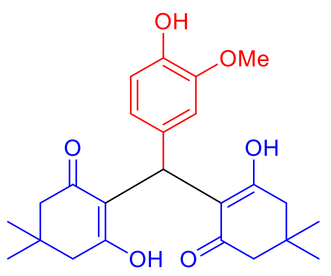


**7h**

97% yield, white solid. $R_f = 0.5$ (40% EtOAc/Hexane). $^1$H NMR (500 MHz, $CDCl_3$) δ 11.98 (s, 1H), 6.81 (d, *J* = 8.3 Hz, 1H), 6.63 – 6.56 (m, 2H), 5.49 (s, 1H), 5.47 (s, 1H), 3.78 (s, 3H), 2.47 – 2.30 (m, 8H), 1.24 (s, 6H), 1.11 (s, 6H). HRMS ($ESI^+$): m/z calculated for $[C_{24}H_{30}O_6 + H^+]$: 415.2121; found 415.2101

**2,2'-( phenylmethylene)bis(3-hydroxycyclohex-2-enone), 7i:**

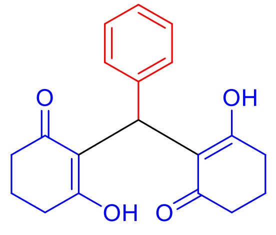

**7i**

94% yield, white solid. $R_f$ = 0.45 (40% EtOAc/Hexane). $^1$H NMR (500 MHz, $CDCl_3$) δ 12.37 (s, 1H), 7.28 (s, 1H), 7.25 (s, 1H), 7.17 (t, *J* = 7.3 Hz, 1H), 7.11 (d, *J* = 8.3 Hz, 2H), 5.47 (s, 1H), 2.69 – 2.54 (m, 4H), 2.50 – 2.35 (m, 4H), 2.07 – 2.00 (m, 4H). HRMS ($ESI^+$): m/z calculated for [$C_{19}H_{20}O_4$ + $H^+$]: 313.1440; found 313.1429

**2,2'-((3-fluorophenyl)methylene)bis(3-hydroxycyclohex-2-enone), 7j:**

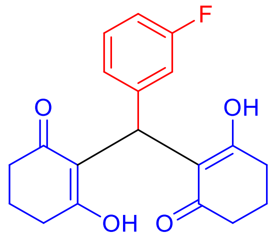

**7j**

95% yield, white solid. $R_f$ = 0.35 (40% EtOAc/Hexane). $^1$H NMR (500 MHz, $CDCl_3$) δ 12.38 (s, 1H), 7.22 (td, *J* = 8.0, 6.2 Hz, 1H), 6.90 – 6.84 (m, 2H), 6.80 (d, *J* = 10.8 Hz, 1H), 5.43 (s, 1H), 2.68 – 2.55 (m, 4H), 2.50 – 2.35 (m, 4H), 2.07 – 2.00 (m, 4H). HRMS ($ESI^+$): m/z calculated for [$C_{19}H_{19}FO_4$ + $H^+$]: 331.1346; found 331.1328

**2,2'-((3-bromophenyl)methylene)bis(3-hydroxycyclohex-2-enone), 7k:**

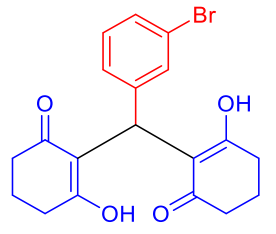

**7k**

93% yield, white solid. $R_f$ = 0.4 (40% EtOAc/Hexane). $^1$H NMR (500 MHz, $CDCl_3$) δ 12.36 (s, 1H), 7.32 – 7.28 (m, 1H), 7.20 (d, *J* = 1.4 Hz, 1H), 7.13 (dd, *J* = 10.1, 5.6 Hz, 1H), 7.03 (d, *J* = 7.9 Hz, 1H), 5.42 (s, 1H), 2.68 – 2.56 (m, 4H), 2.49 – 2.35 (m, 4H), 2.07 – 2.00 (m, 4H). HRMS ($ESI^+$): m/z calculated for [$C_{19}H_{19}BrO_4 + H^+$]: 391.0545; found 391.0529

**2,2'-((4-nitrophenyl)methylene)bis(3-hydroxycyclohex-2-enone), 7l:**

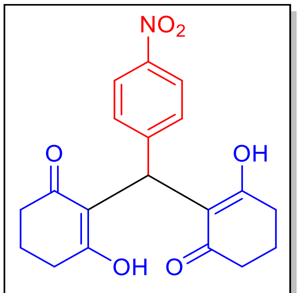


**7l**

87% yield, white solid. $R_f$ = 0.45 (40% EtOAc/Hexane). $^1$H NMR (500 MHz, $CDCl_3$) δ 12.27 (s, 1H), 8.15 – 8.13 (m, 1H), 8.13 – 8.11 (m, 1H), 7.28 – 7.27 (m, 1H), 7.26 (d, *J* = 1.1 Hz, 1H), 5.48 (s, 1H), 2.64 (ddt, *J* = 24.5, 18.0, 3.7 Hz, 4H), 2.45 (dddd, *J* = 32.5, 17.7, 11.5, 5.9 Hz, 4H), 2.10 – 2.02 (m, 4H). HRMS ($ESI^+$): m/z calculated for [$C_{19}H_{19}NO_6 + H^+$]: 358.1291; found 358.1269

**2,2'-((2-chlorophenyl)methylene)bis(3-hydroxycyclohex-2-enone), 7m:**

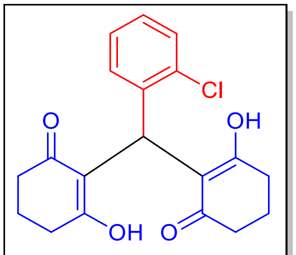


**7m**

96% yield, white solid. $R_f$ = 0.42 (40% EtOAc/Hexane). $^1$H NMR (500 MHz, $CDCl_3$) δ 12.37 (s, 1H), 7.33 – 7.28 (m, 1H), 7.21 (td, *J* = 7.7, 1.5 Hz, 1H), 7.17 – 7.07 (m, 2H), 5.54 (s, 1H), 2.61 – 2.50 (m, 2H), 2.48 – 2.33 (m, 2H), 1.58 (s, 4H), 1.25 (s, 4H). HRMS ($ESI^+$): m/z calculated for [$C_{19}H_{19}ClO_4 + H^+$]: 347.1050; found 347.1038

**2,2'-((2-nitrophenyl)methylene)bis(3-hydroxycyclohex-2-enone), 7n:**

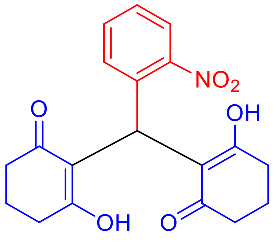


**7n**

95% yield, white solid. $R_f$ = 0.5 (40% EtOAc/Hexane). $^1$H NMR (500 MHz, $CDCl_3$) δ 12.21 (s, 1H), 7.51 (dd, *J* = 7.9, 1.3 Hz, 1H), 7.49 – 7.45 (m, 1H), 7.33 (t, *J* = 7.7 Hz, 1H), 7.24 (d, *J* = 8.0 Hz, 1H), 5.96 (s, 1H), 2.66 – 2.51 (m, 4H), 2.44 – 2.29 (m, 4H), 2.00 – 1.93 (m, 4H). HRMS ($ESI^+$): m/z calculated for [$C_{19}H_{19}NO_6 + H^+$]: 358.1291; found 358.1278

**2,2'-((4-methoxyphenyl)methylene)bis(3-hydroxycyclohex-2-enone), 7o**:

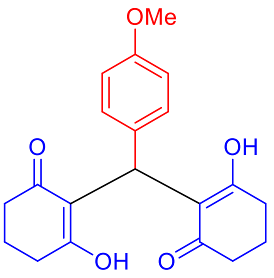


**7o**

83% yield, white solid. $R_f$ = 0.55 (40% EtOAc/Hexane). $^1$H NMR (500 MHz, $CDCl_3$) δ 12.38 (s, 1H), 7.01 (dd, *J* = 8.9, 1.1 Hz, 2H), 6.81 – 6.79 (m, 2H), 5.42 (s, 1H), 3.78 (s, 3H), 2.66 – 2.54 (m, 2H), 2.41 (ddd, *J* = 28.2, 17.5, 8.5 Hz, 2H), 1.58 (s, 4H), 1.26 (d, *J* = 12.3 Hz, 4H). HRMS ($ESI^+$): m/z calculated for [$C_{20}H_{22}O_5 + H^+$]: 343.1545; found 343.1524

**2,2'-((4-hydroxy-3-methoxyphenyl)methylene)bis(3-hydroxycyclohex-2-enone), 7p:**

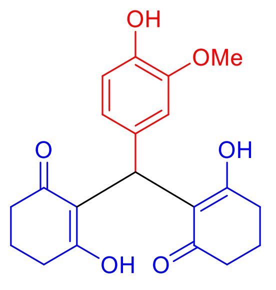


**7p**

88% yield, white solid. $R_f$ = 0.4 (40% EtOAc/Hexane). $^1$H NMR (500 MHz, $CDCl_3$) δ 12.44 (s, 1H), 6.81 (d, *J* = 8.4 Hz, 1H), 6.60 (dd, *J* = 7.7, 1.2 Hz, 2H), 5.47 (s, 1H), 5.42 (s, 1H), 3.80 (s, 3H), 2.60 (dd, *J* = 32.9, 17.8 Hz, 4H), 2.49 – 2.34 (m, 4H), 2.02 (ddd, *J* = 18.6, 10.6, 6.1 Hz, 4H). HRMS ($ESI^+$): m/z calculated for [$C_{20}H_{22}O_6 + H^+$]: 359.1495; found 359.1469

**9-(2-hydroxy-4,4-dimethyl-6-oxocyclohex-1-en-1-yl)-3,3-dimethyl-2,3,4,9-tetrahydro-1*H*-xanthen-1-one, 9a:**

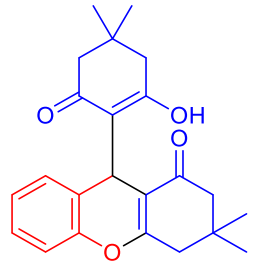


**9a**

96% yield, white solid. $R_f$ = 0.35 (50% EtOAc/Hexane). $^1$H NMR (500 MHz, $CDCl_3$) δ 10.51 (s, 1H), 7.18 – 7.14 (m, 1H), 7.03 (s, 1H), 7.00 (dd, *J* = 7.3, 5.1 Hz, 3H), 4.66 (s, 1H), 2.60 (d, *J* = 17.6 Hz, 1H), 2.48 (d, *J* = 17.5 Hz, 1H), 2.39 – 2.36 (m, 2H), 2.33 (d, *J* = 2.2 Hz, 2H), 1.99 (d, *J* = 16.6 Hz, 1H), 1.93 (d, *J* = 16.5 Hz, 1H), 1.13 (s, 3H), 1.03 (s, 3H), 0.99 (s, 6H). $^{13}$C NMR (126 MHz, CDCl3) δ 196.64, 170.76, 151.01, 128.02, 127.52, 124.58, 124.35, 118.32, 115.74, 111.05, 50.58, 49.95, 43.18, 41.54, 32.30, 30.98, 29.84, 29.19, 27.73, 27.20, 26.53. HRMS ($ESI^+$): m/z calculated for [$C_{23}H_{26}O_4 + H^+$]: 367.1909; found 367.1935

**7-bromo-9-(2-hydroxy-4,4-dimethyl-6-oxocyclohex-1-en-1-yl)-3,3-dimethyl-2,3,4,9-tetrahydro-1*H*-xanthen-1-one, 9b:**

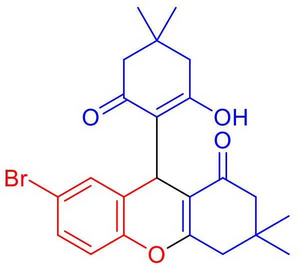


**9b**

98% yield, light pink solid. $R_f$ = 0.4 (50% EtOAc/Hexane). $R_f$ = 0.4 (40% EtOAc/Hexane). $^1$H NMR (500 MHz, $CDCl_3$) δ 10.42 (s, 1H), 7.24 (d, *J* = 2.3 Hz, 1H), 7.13 (d, *J* = 1.9 Hz, 1H), 6.91 (d, *J* = 8.6 Hz, 1H), 4.60 (s, 1H), 2.63 – 2.43 (m, 2H), 2.36 (dd, *J* = 28.3, 9.9 Hz, 4H), 2.18 (s, 2H), 1.13 (s, 3H), 1.02 (d, *J* = 1.0 Hz, 6H), 1.00 (s, 3H). 13C NMR (126 MHz, CDCl3) δ 196.02, 168.98, 167.33, 165.05, 160.70, 144.13, 111.00, 103.17, 72.83, 61.30, 44.73, 40.81, 31.58, 31.57, 28.66, 28.65, 26.53, 26.52, 26.43. HRMS (ESI$^+$): m/z calculated for [$C_{23}H_{25}BrO_4$ + $H^+$]: 445.1014; found 445.1030

**9-(2-hydroxy-4,4-dimethyl-6-oxocyclohex-1-en-1-yl)-6-methoxy-3,3-dimethyl-2,3,4,9-tetrahydro-1*H*-xanthen-1-one, 9c:**

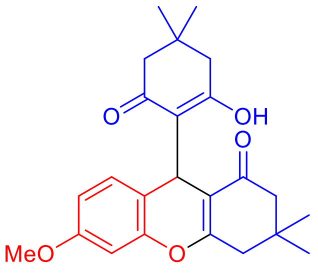


**9c**

91% yield, white solid. $R_f$ = 0.5 (50% EtOAc/Hexane). $^1$H NMR (500 MHz, $CDCl_3$) δ 10.45 (s, 1H), 6.89 (d, *J* = 9.3 Hz, 1H), 6.58 (dd, *J* = 7.5, 2.1 Hz, 2H), 4.61 (s, 1H), 3.77 (s, 3H), 2.59 (d, *J* = 17.6 Hz, 1H), 2.47 (d, *J* = 17.5 Hz, 1H), 2.37 (d, *J* = 3.3 Hz, 2H), 2.33 (d, *J* = 2.5 Hz, 2H), 1.99 (d, *J* = 16.6 Hz, 1H), 1.94 (d, *J* = 16.5 Hz, 1H), 1.12 (s, 3H), 1.02 (s, 3H), 0.99 (d, *J* = 1.6 Hz, 6H). 13C NMR (126 MHz, CDCl3) δ 196.71, 170.54, 161.23, 158.93, 151.59, 128.39, 118.30, 116.21, 111.53, 111.40, 110.99, 101.13, 61.90, 55.35, 50.69, 49.94, 43.16, 41.51, 32.34, 30.95,

29.92, 29.17, 27.24, 26.47. HRMS (ESI$^+$): m/z calculated for [$C_{24}H_{28}O_5$ + $H^+$]: 397.2015; found 397.2047

**9-(2-hydroxy-4,4-dimethyl-6-oxocyclohex-1-en-1-yl)-8-methoxy-3,3-dimethyl-2,3,4,9-tetrahydro-1*H*-xanthen-1-one, 9d:**

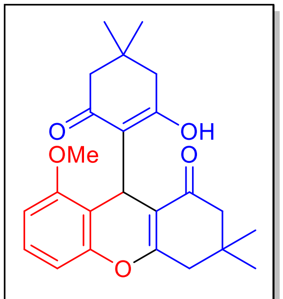

**9d**

88% yield, white solid. $R_f$ = 0.52 (50% EtOAc/Hexane). $^1$H NMR (500 MHz, $CDCl_3$) δ 10.46 (s, 1H), 6.95 (t, *J* = 7.9 Hz, 1H), 6.76 (dd, *J* = 8.2, 1.2 Hz, 1H), 6.61 – 6.58 (m, 1H), 4.66 (s, 1H), 3.89 (s, 3H), 2.68 (d, *J* = 17.6 Hz, 1H), 2.56 (d, *J* = 17.5 Hz, 1H), 2.37 (d, *J* = 2.0 Hz, 2H), 2.33 (s, 2H), 2.00 (d, *J* = 16.6 Hz, 1H), 1.94 (d, *J* = 16.5 Hz, 1H), 1.12 (s, 3H), 1.03 (s, 3H), 0.99 (s, 6H). $^{13}$C NMR (126 MHz, CDCl3) δ 194.35, 161.22, 147.08, 144.65, 140.63, 125.24, 124.24, 119.78, 118.16, 111.52, 110.91, 110.33, 61.82, 56.06, 49.95, 45.25, 41.55, 32.32, 30.93, 29.13, 27.75, 27.20. HRMS (ESI$^+$): m/z calculated for [$C_{24}H_{28}O_5$ + $H^+$]: 397.2015; found 397.2045

**7-(2-hydroxy-4,4-dimethyl-6-oxocyclohex-1-en-1-yl)-10,10-dimethyl-10,11-dihydro-7*H*-benzo[*c*]xanthen-8(9*H*)-one, 9e:**

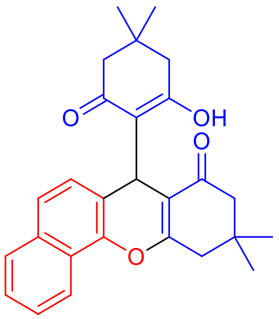

**9e**

92% yield, white solid. $R_f$ = 0.4 (50% EtOAc/Hexane). $^1$H NMR (500 MHz, $CDCl_3$) δ 10.69 (s, 1H), 7.77 (d, *J* = 7.8 Hz, 1H), 7.71 (dd, *J* = 8.6, 2.7 Hz, 2H), 7.46 (ddd, *J* = 8.4, 5.5, 1.3 Hz, 1H), 7.40 – 7.35 (m, 1H), 5.26 (s, 1H), 2.67 (d, *J* = 17.6 Hz, 1H), 2.56 (d, *J* = 17.6 Hz, 1H), 2.42 – 2.37 (m, 4H), 1.95 (d, *J* = 16.6 Hz, 1H), 1.81 (d, *J* = 16.6 Hz, 1H), 1.16 (s, 3H), 1.06 (s, 3H), 0.94 (s, 3H), 0.70 (s, 3H). 13C NMR (126 MHz, CDCl3) δ 201.56, 197.24, 172.75, 170.97, 161.23, 158.91, 151.43, 144.66, 128.48, 119.81, 116.52, 112.67, 111.53, 111.05, 100.92, 94.96, 61.83, 55.35, 45.27, 37.08, 36.04, 29.74, 27.98, 27.47, 19.96, 19.66. HRMS (ESI$^+$): m/z calculated for [$C_{27}H_{28}O_4$ + $H^+$]: 417.2066; found 417.2093

**9-(2-hydroxy-6-oxocyclohex-1-en-1-yl)-2,3,4,9-tetrahydro-1*H*-xanthen-1-one, 9f:**

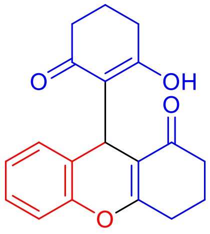

**9f**

94% yield, white solid. $R_f$ = 0.45 (50% EtOAc/Hexane). $^1$H NMR (500 MHz, $CDCl_3$) δ 10.85 (s, 1H), 7.16 (dt, *J* = 8.4, 4.3 Hz, 1H), 7.02 (t, *J* = 6.4 Hz, 3H), 4.64 (s, 1H), 2.77 (dt, *J* = 17.8, 4.6 Hz, 1H), 2.65 – 2.51 (m, 3H), 2.47 – 2.37 (m, 2H), 2.06 (dddd, *J* = 22.1, 17.1, 8.8, 4.1 Hz, 4H), 1.89 – 1.72 (m, 2H). 13C NMR (126 MHz, CDCl3) δ 193.16, 190.24, 155.89, 139.32, 127.81, 126.52, 123.81, 123.80, 120.69, 114.79, 111.75, 106.19, 56.49, 35.84, 27.32, 19.58, 19.43, 19.05. HRMS (ESI$^+$): m/z calculated for [$C_{19}H_{18}O_4$ + $H^+$]: 311.1283; found 311.1293

**7-bromo-9-(2-hydroxy-6-oxocyclohex-1-en-1-yl)-2,3,4,9-tetrahydro-1*H*-xanthen-1-one, 9g:**

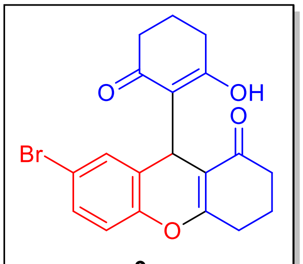


9g

96% yield, white solid. $R_f$ = 0.45 (50% EtOAc/Hexane). $^1$H NMR (500 MHz, $CDCl_3$) δ 10.77 (s, 1H), 7.25 (d, *J* = 2.3 Hz, 1H), 7.14 – 7.12 (m, 1H), 6.91 (d, *J* = 8.6 Hz, 1H), 4.57 (s, 1H), 2.75 (dt, *J* = 18.0, 4.7 Hz, 1H), 2.62 – 2.51 (m, 3H), 2.46 – 2.38 (m, 2H), 2.10 – 1.94 (m, 4H), 1.90 – 1.77 (m, 2H). HRMS (ESI$^+$): m/z calculated for [$C_{19}H_{17}BrO_4$ + $H^+$]: 389.0388; found 389.0410

**9-(2-hydroxy-6-oxocyclohex-1-en-1-yl)-6-methoxy-2,3,4,9-tetrahydro-1*H*-xanthen-1-one, 9h:**

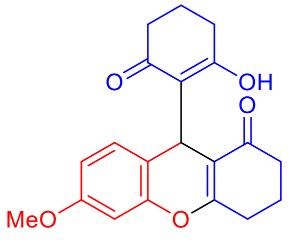


9h

87% yield, white solid. $R_f$ = 0.35 (50% EtOAc/Hexane). $^1$H NMR (500 MHz, $CDCl_3$) δ 10.80 (s, 1H), 6.90 (d, *J* = 7.9 Hz, 1H), 6.66 – 6.52 (m, 2H), 4.58 (s, 1H), 3.77 (s, 2H), 2.75 (dt, *J* = 17.9, 4.5 Hz, 1H), 2.63 – 2.50 (m, 3H), 2.41 (ddd, *J* = 18.3, 11.6, 5.3 Hz, 2H), 2.15 – 1.96 (m, 4H), 1.79 (dddd, *J* = 20.2, 17.8, 10.8, 6.7 Hz, 2H). HRMS (ESI$^+$): m/z calculated for [$C_{20}H_{20}O_5$ + $H^+$]: 341.1389; found 341.1412

**7-(2-hydroxy-6-oxocyclohex-1-en-1-yl)-10,11-dihydro-7*H*-benzo[c]xanthen-8(9*H*)-one, 9i:**

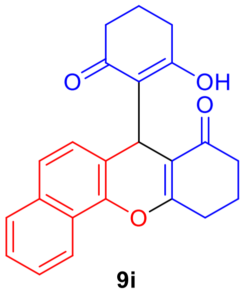


**9i**

$^{1}H$ NMR (500 MHz, $CDCl_3$) δ 11.02 (s, 1H), 7.78 (d, *J* = 8.1 Hz, 1H), 7.70 (dd, *J* = 14.3, 8.7 Hz, 2H), 7.46 (ddd, *J* = 8.4, 6.9, 1.3 Hz, 1H), 7.38 (ddd, *J* = 8.0, 6.9, 1.1 Hz, 1H), 5.25 (s, 1H), 2.84 (dt, *J* = 17.9, 4.5 Hz, 1H), 2.71 – 2.57 (m, 3H), 2.52 – 2.40 (m, 2H), 2.15 – 1.96 (m, 5H), 1.80 – 1.71 (m, 1H). HRMS ($ESI^+$): m/z calculated for [$C_{23}H_{20}O_4 + H^+$]: 361.1440; found 361.1467

## 7. Copies of $^{1}$H and $^{13}$C NMR spectra of products (4a-4t), (6a-6l), (7a-7p), and (9a-9i):

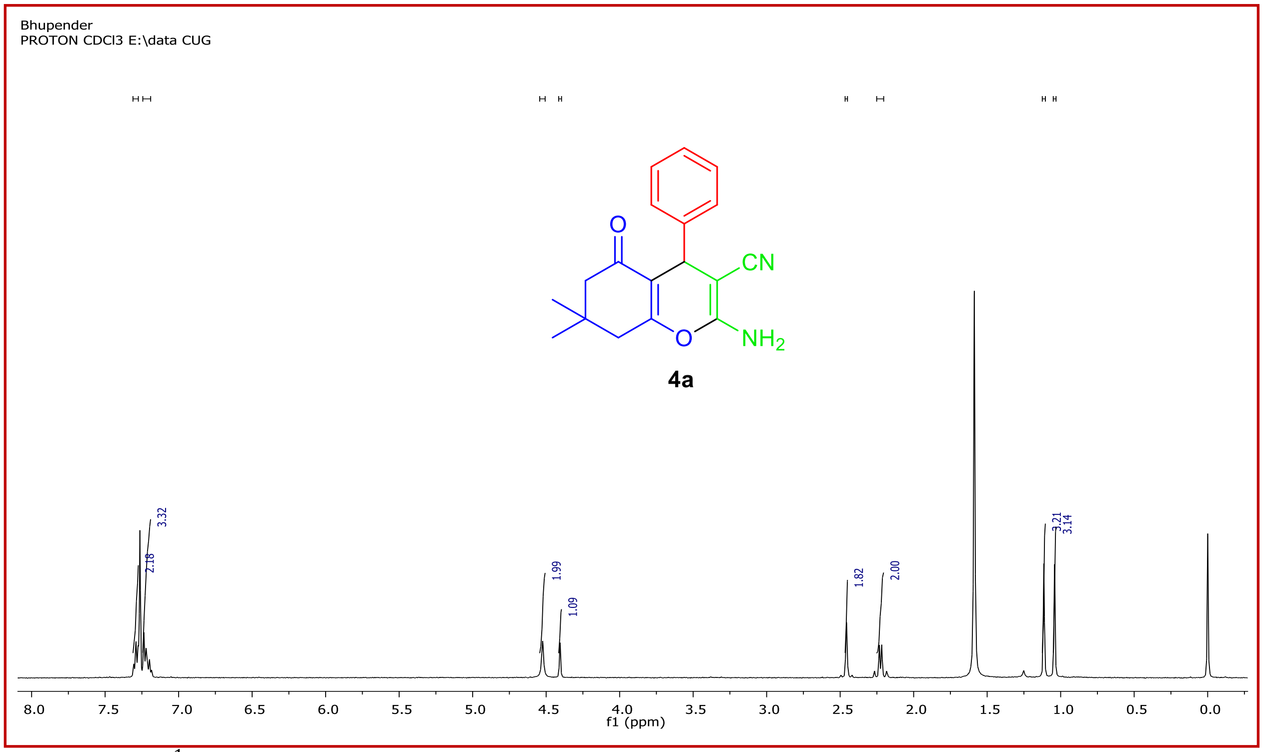


**Figure S5**: $^{1}$H NMR spectra of 2-amino-7,7-dimethyl-5-oxo-4-phenyl-5,6,7,8-tetrahydro-4*H*-chromene-3-carbonitrile **4a**

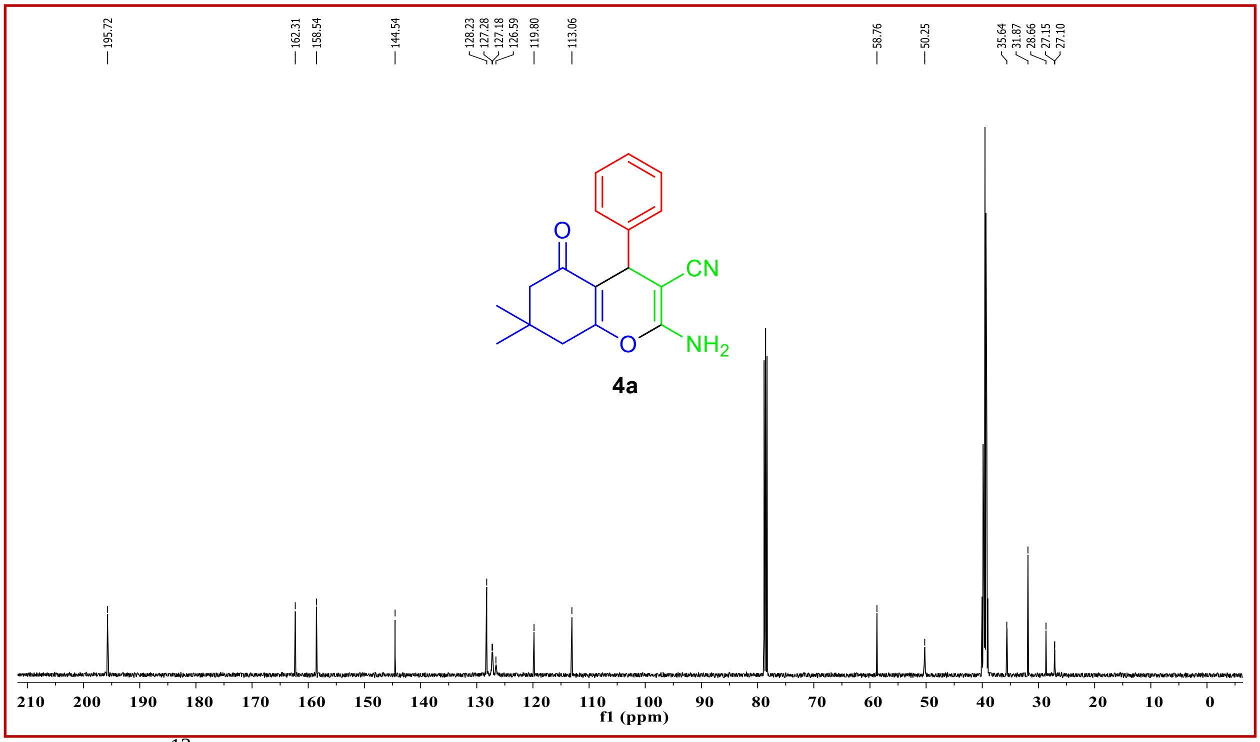


**Figure S6**: $^{13}$C NMR spectra of 2-amino-7,7-dimethyl-5-oxo-4-phenyl-5,6,7,8-tetrahydro-4*H*-chromene-3-carbonitrile **4a**

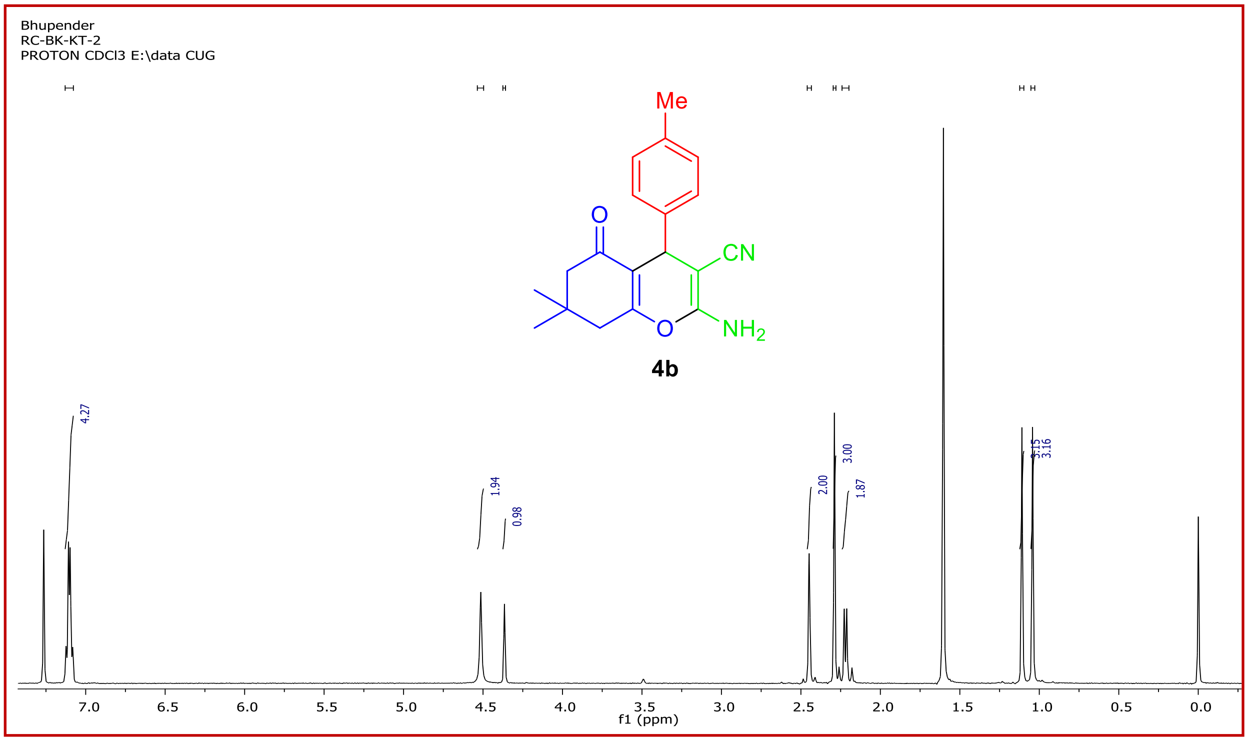


**Figure S7**: $^{1}$H NMR spectra of 2-amino-7,7-dimethyl-5-oxo-4-(p-tolyl)-5,6,7,8-tetrahydro-4*H*-chromene-3-carbonitrile **4b**

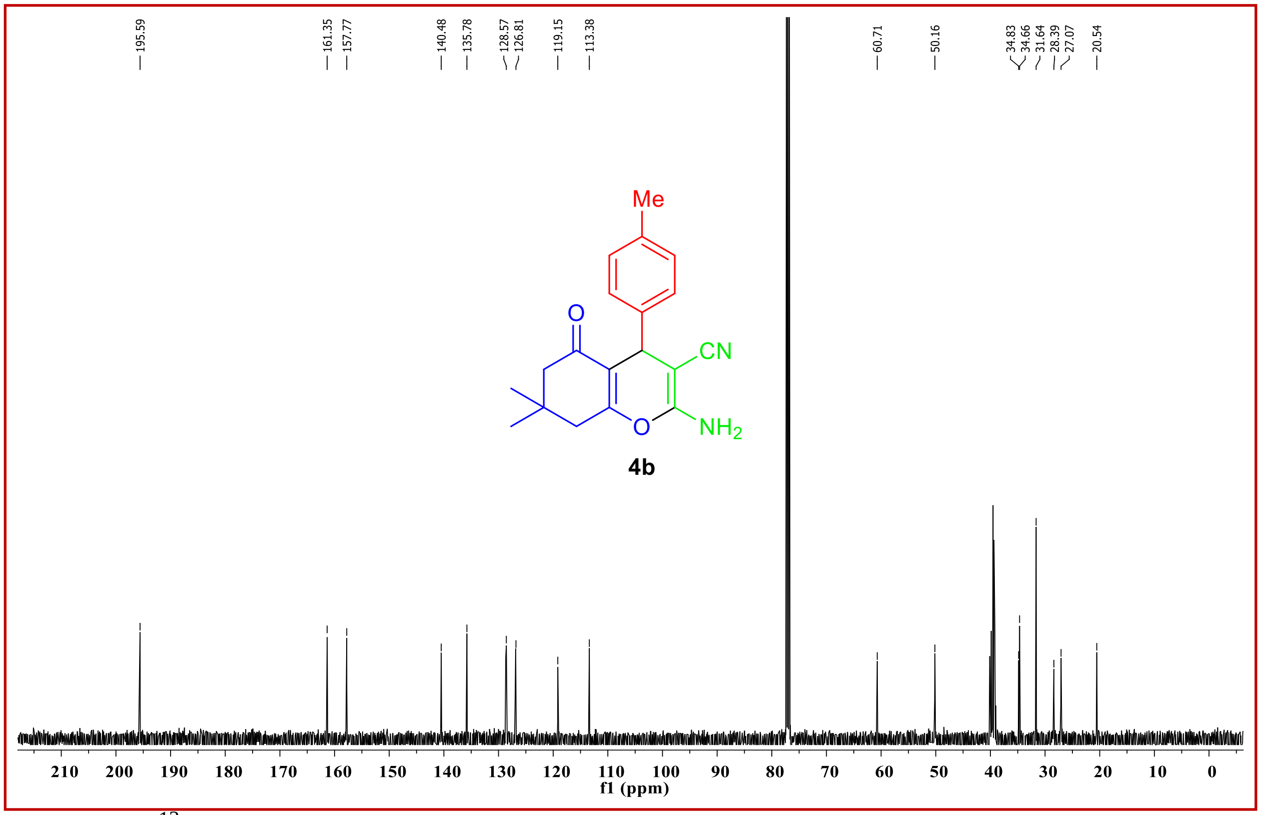


**Figure S8**: $^{13}$C NMR spectra of 2-amino-7,7-dimethyl-5-oxo-4-(p-tolyl)-5,6,7,8-tetrahydro-4*H*-chromene-3-carbonitrile **4b**

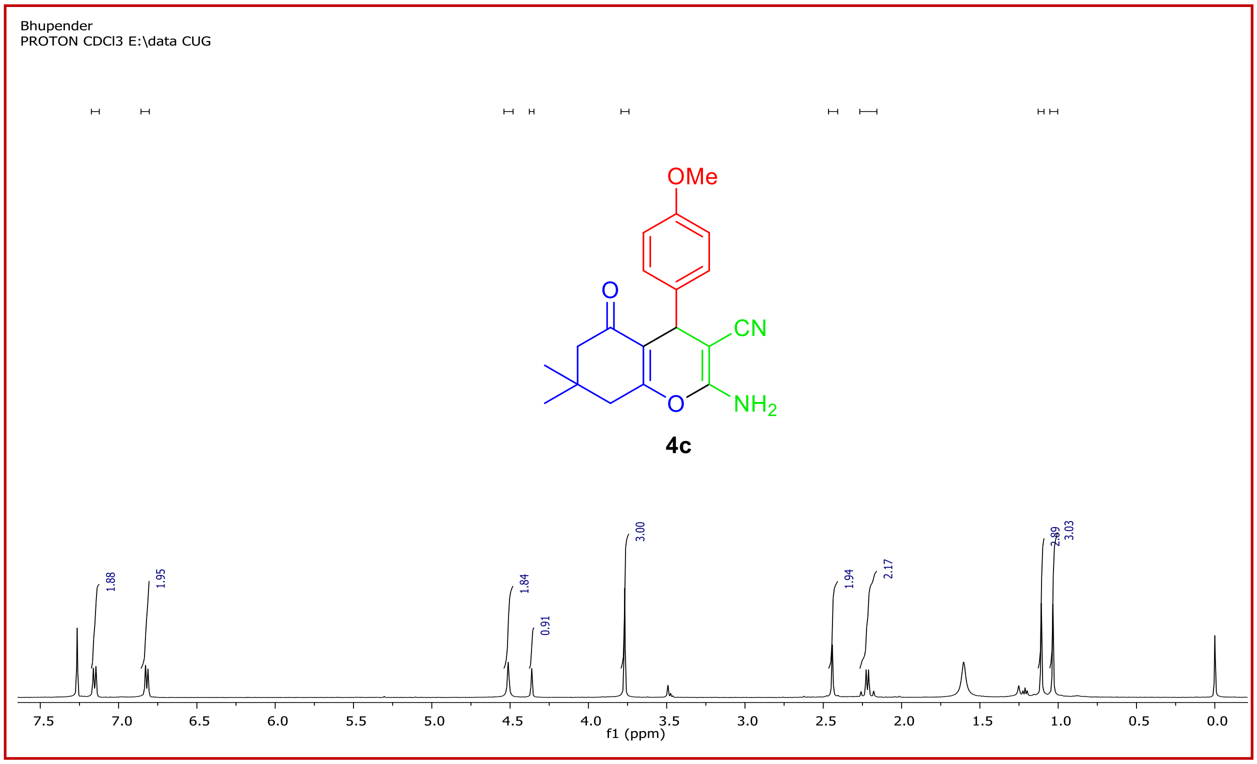


**Figure S9**: $^{1}$H NMR spectra of 2-amino-4-(4-methoxyphenyl)-7,7-dimethyl-5-oxo-5,6,7,8-tetrahydro-4*H*-chromene-3-carbonitrile **4c**

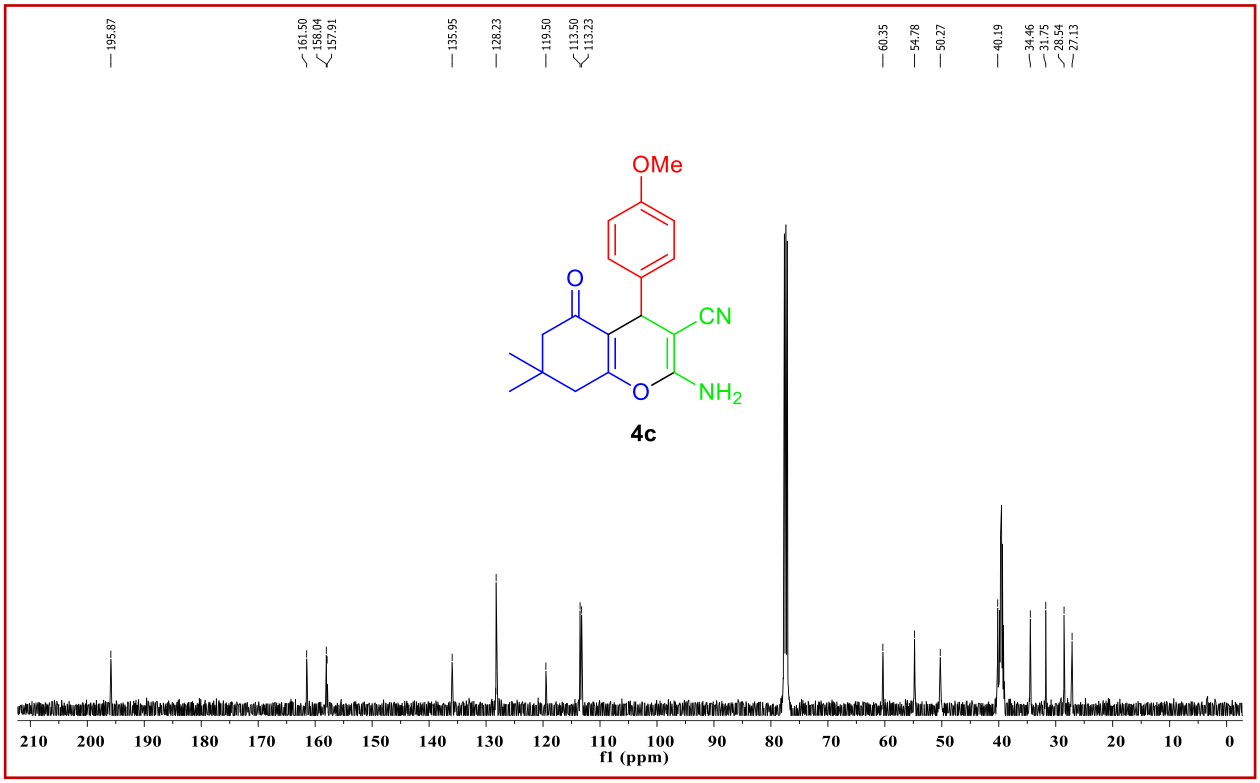


**Figure S10**: $^{13}$C NMR spectra of 2-amino-4-(4-methoxyphenyl)-7,7-dimethyl-5-oxo-5,6,7,8-tetrahydro-4*H*-chromene-3-carbonitrile **4c**

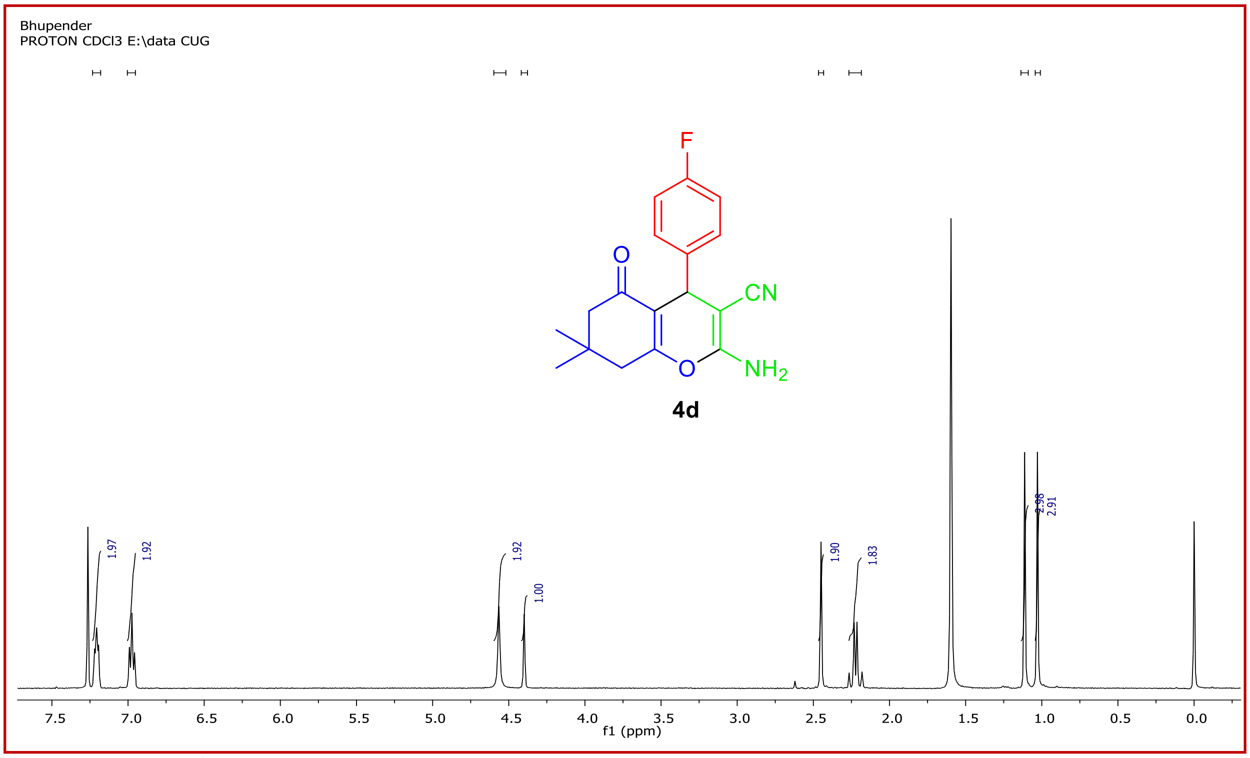


**Figure S11**: $^{1}$H NMR spectra of 2-amino-4-(4-fluorophenyl)-7,7-dimethyl-5-oxo-5,6,7,8-tetrahydro-4*H*-chromene-3-carbonitrile **4d**

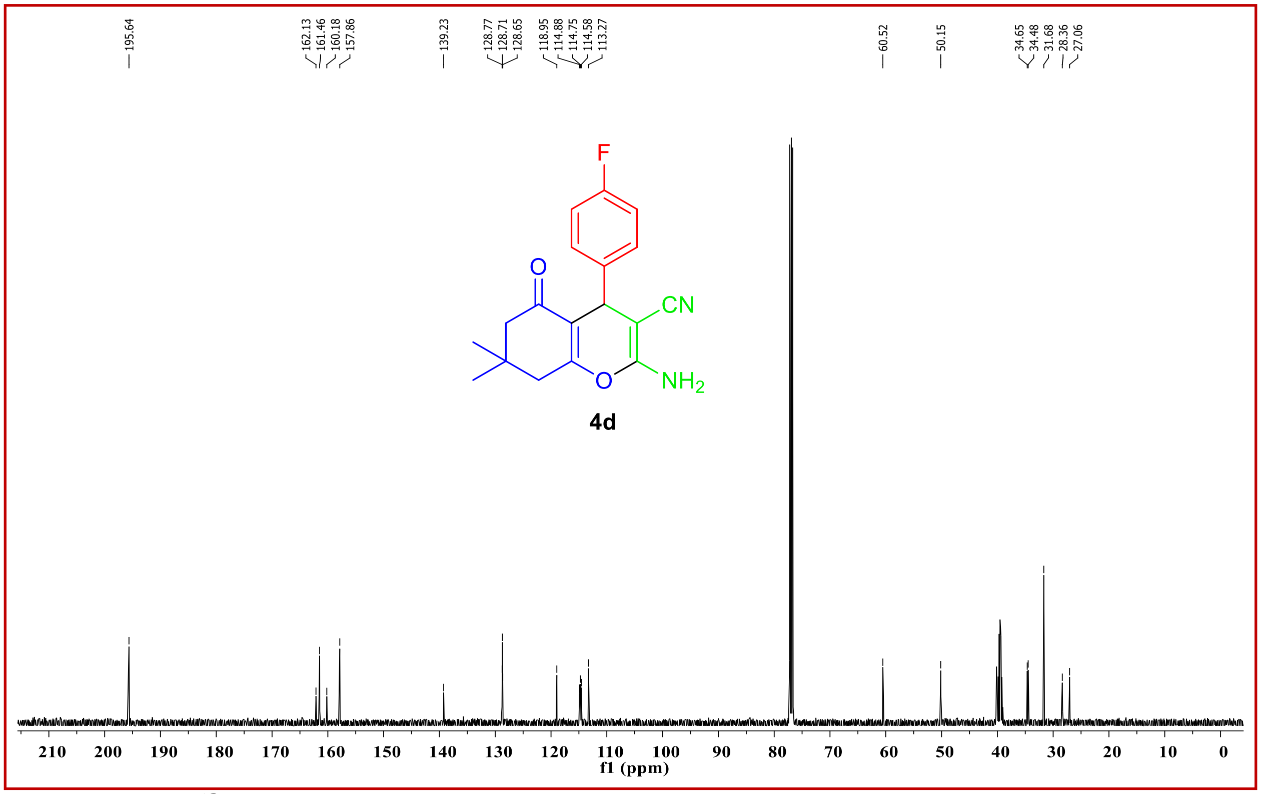


**Figure S12**: $^{13}$C NMR spectra of 2-amino-4-(4-fluorophenyl)-7,7-dimethyl-5-oxo-5,6,7,8-tetrahydro-4*H*-chromene-3-carbonitrile **4d**

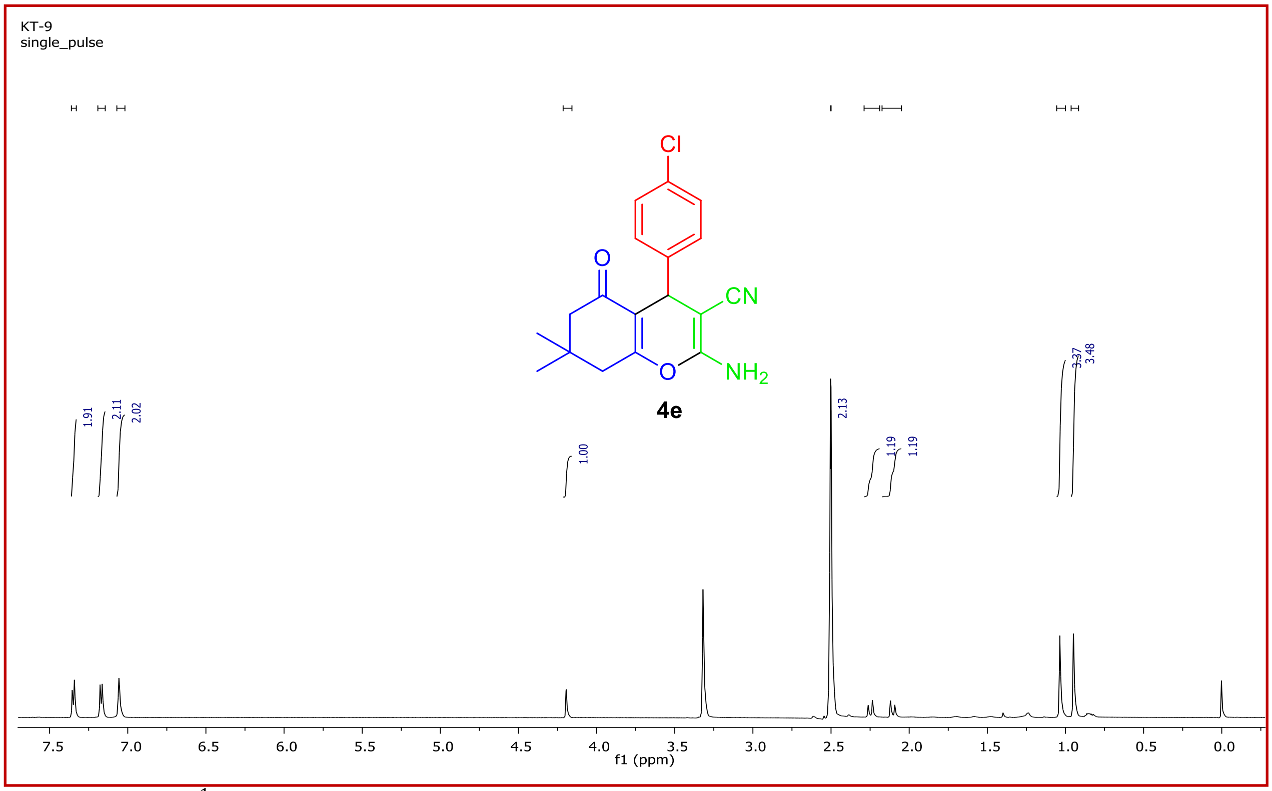


**Figure S13**: $^{1}$H NMR spectra of 2-amino-4-(4-chlorophenyl)-7,7-dimethyl-5-oxo-5,6,7,8-tetrahydro-4*H*-chromene-3-carbonitrile **4e**

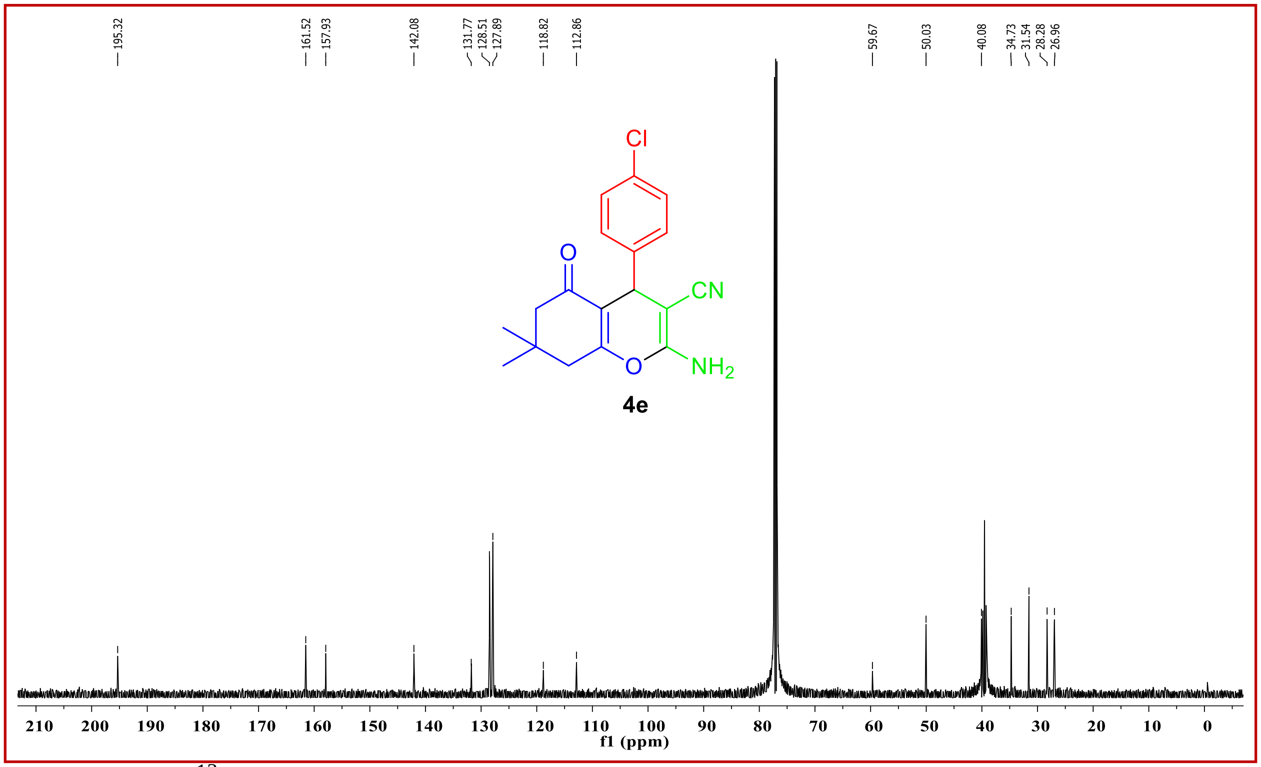


**Figure S14**: $^{13}$C NMR spectra of 2-amino-4-(4-chlorophenyl)-7,7-dimethyl-5-oxo-5,6,7,8-tetrahydro-4*H*-chromene-3-carbonitrile **4e**

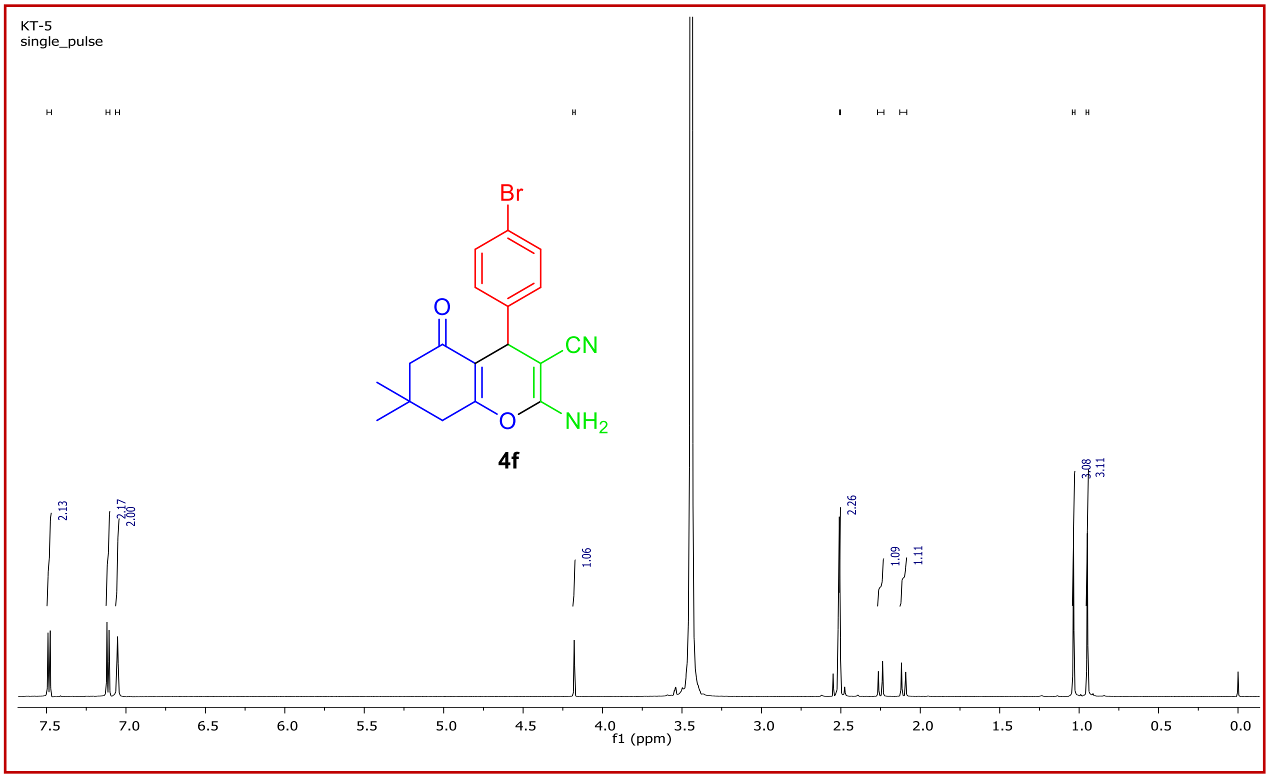


**Figure S15**: $^{1}$H NMR spectra of 2-amino-4-(4-bromophenyl)-7,7-dimethyl-5-oxo-5,6,7,8-tetrahydro-4*H*-chromene-3-carbonitrile **4f**

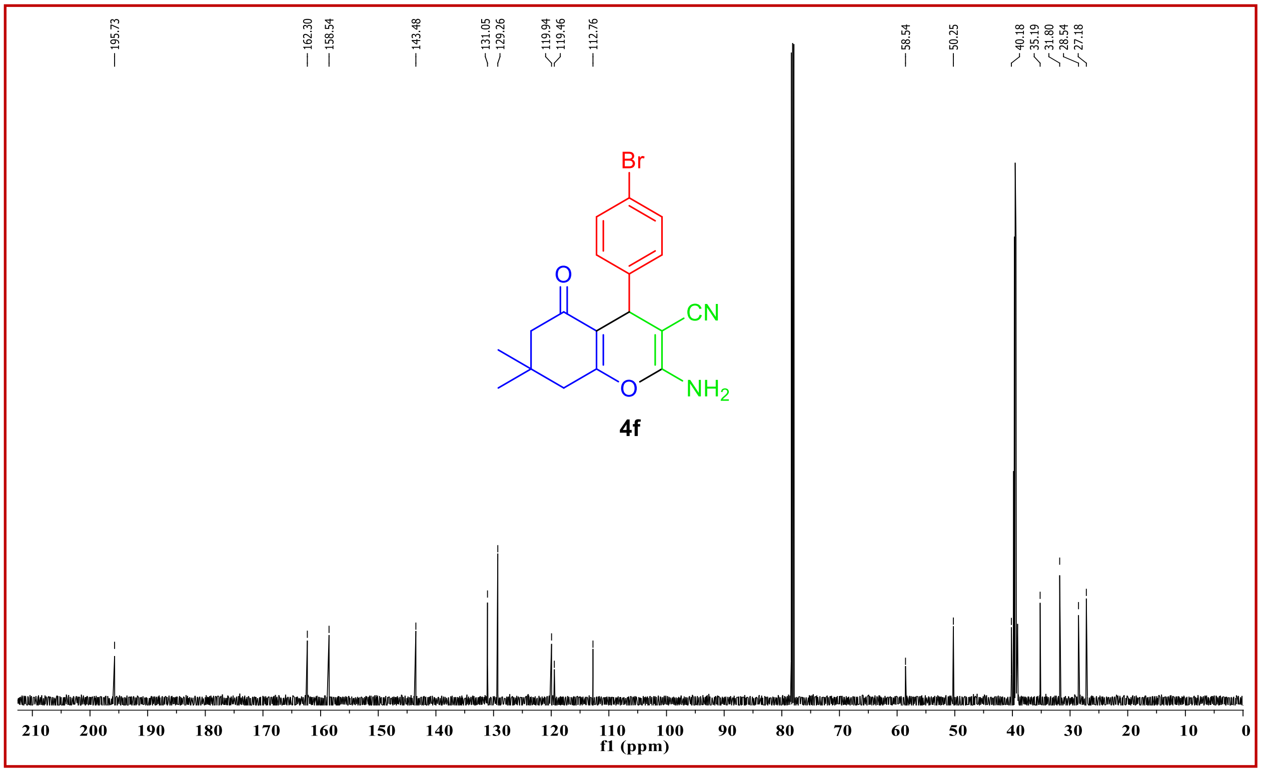


**Figure S16**: $^{13}$C NMR spectra of 2-amino-4-(4-bromophenyl)-7,7-dimethyl-5-oxo-5,6,7,8-tetrahydro-4*H*-chromene-3-carbonitrile **4f**

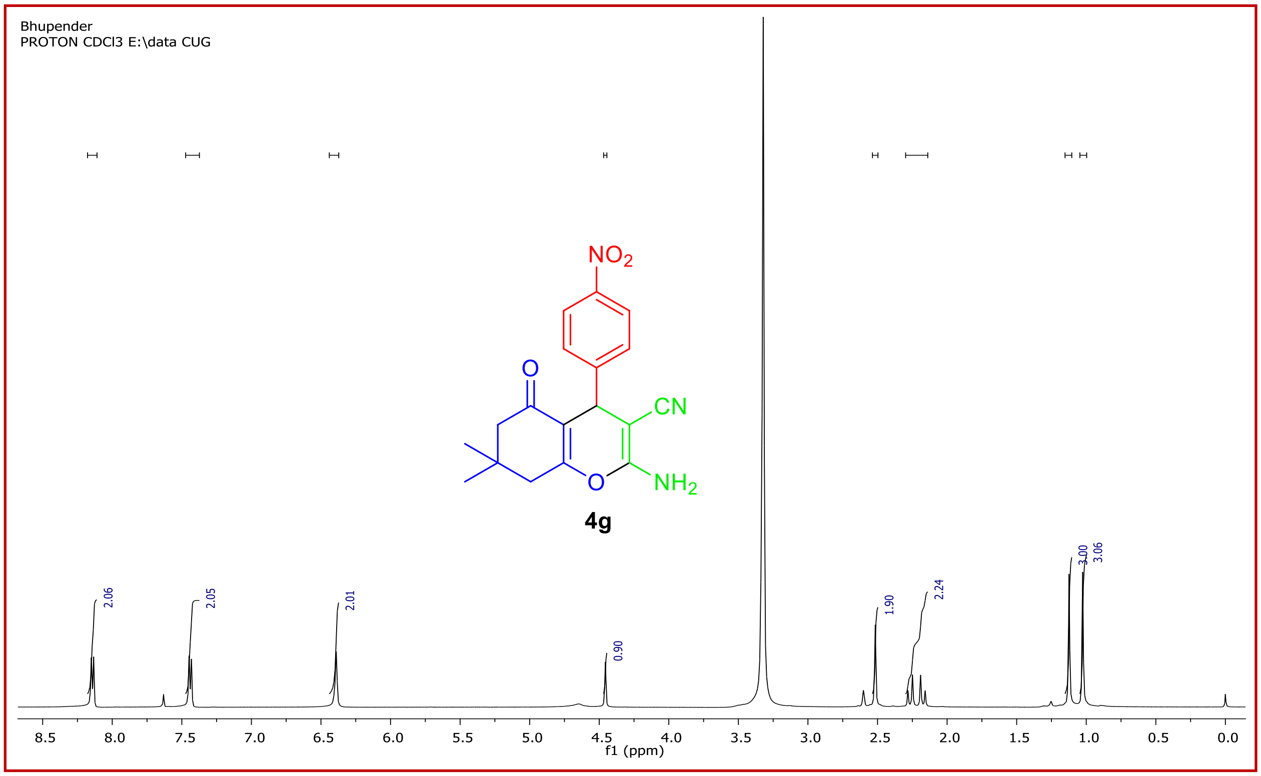


**Figure S17**: $^{1}$H NMR spectra of 2-amino-7,7-dimethyl-4-(4-nitrophenyl)-5-oxo-5,6,7,8-tetrahydro-4*H*-chromene-3-carbonitrile **4g**

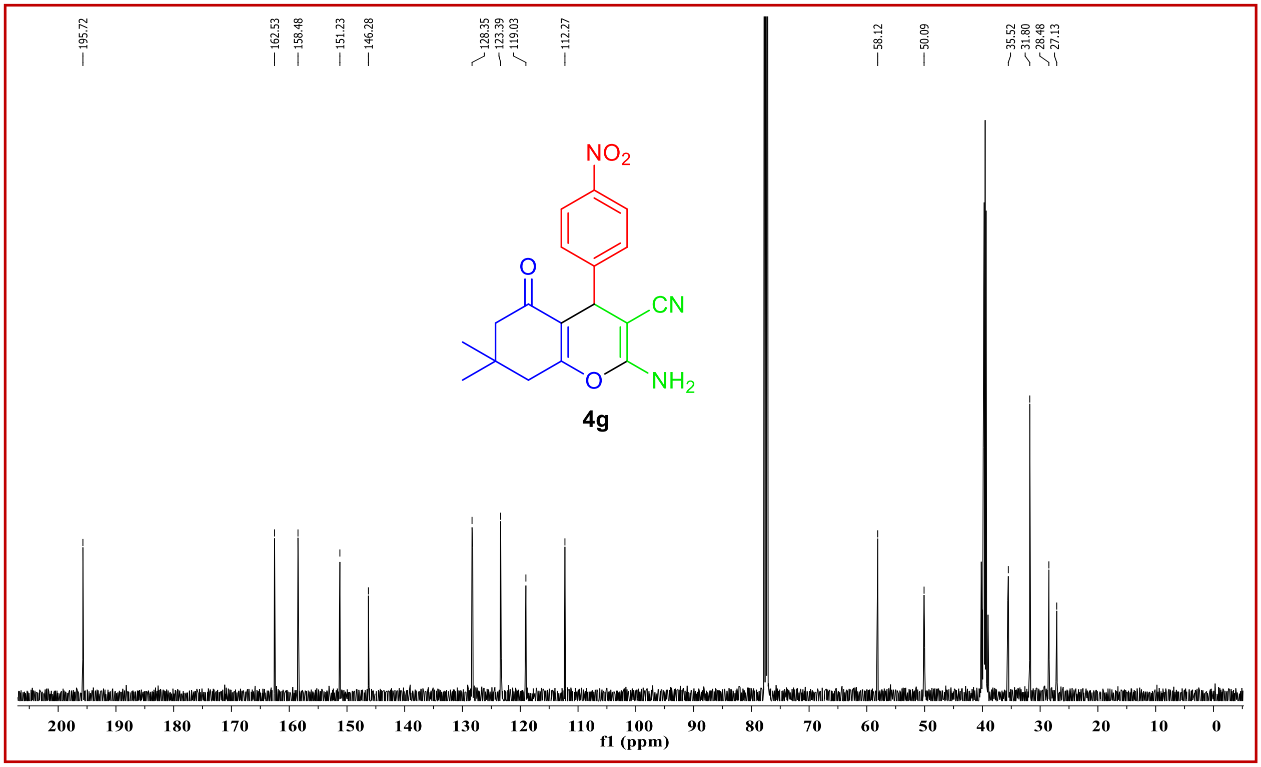


**Figure S18**: $^{13}$C NMR spectra of 2-amino-7,7-dimethyl-4-(4-nitrophenyl)-5-oxo-5,6,7,8-tetrahydro-4*H*-chromene-3-carbonitrile **4g**

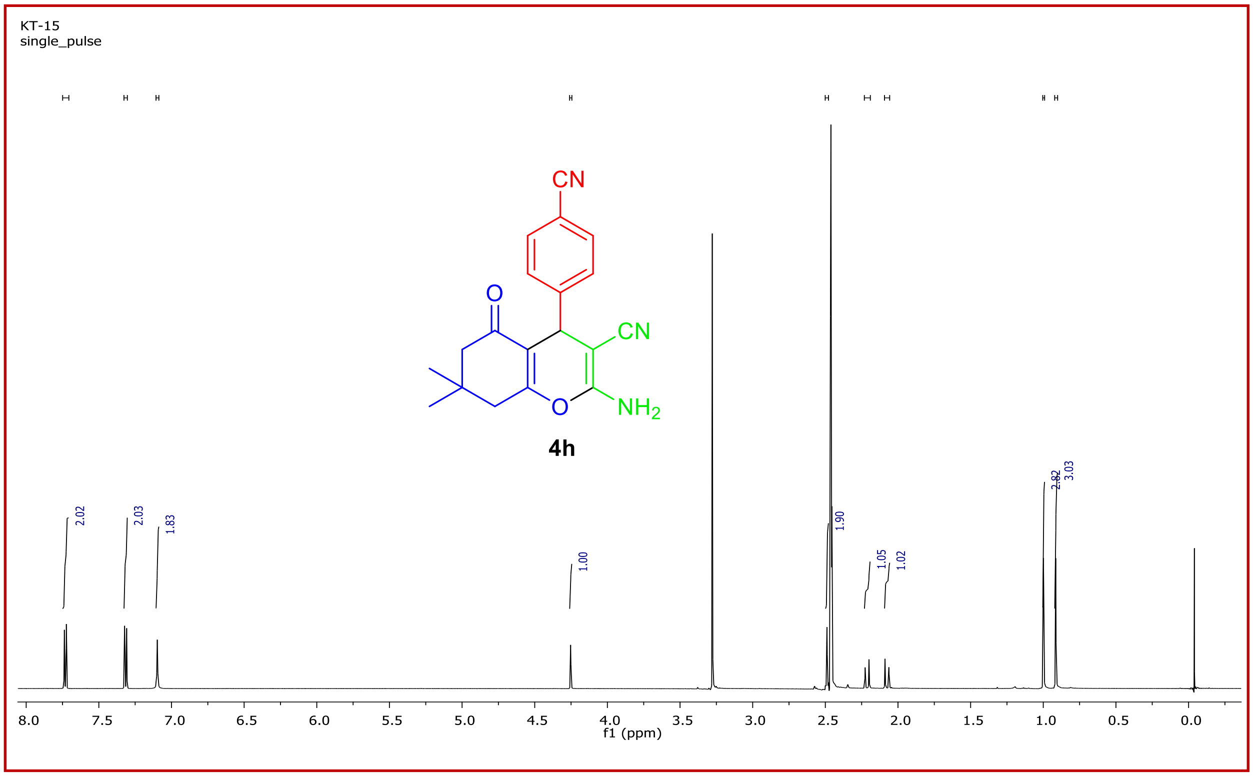


**Figure S19**: $^{1}$H NMR spectra of 2-amino-4-(4-cyanophenyl)-7,7-dimethyl-5-oxo-5,6,7,8-tetrahydro-4*H*-chromene-3-carbonitrile **4h**

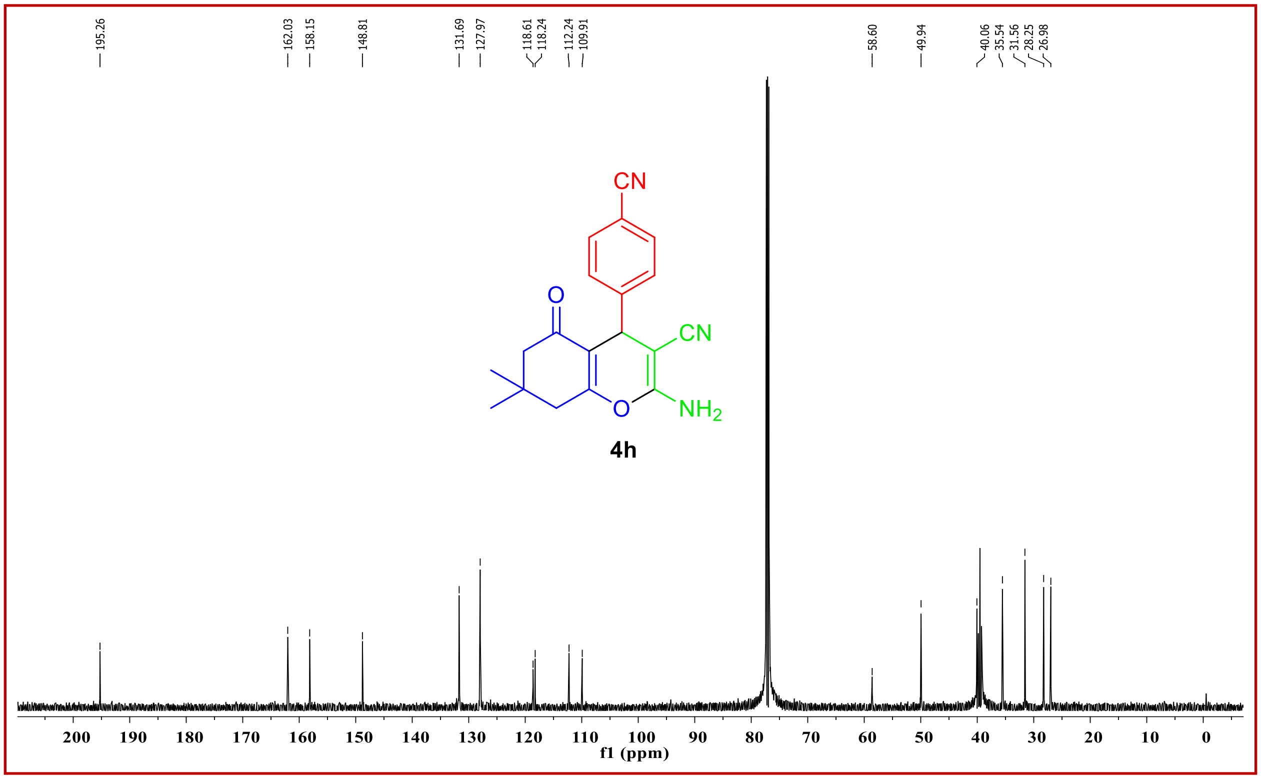


**Figure S20**: $^{13}$C NMR spectra of 2-amino-4-(4-cyanophenyl)-7,7-dimethyl-5-oxo-5,6,7,8-tetrahydro-4*H*-chromene-3-carbonitrile **4h**

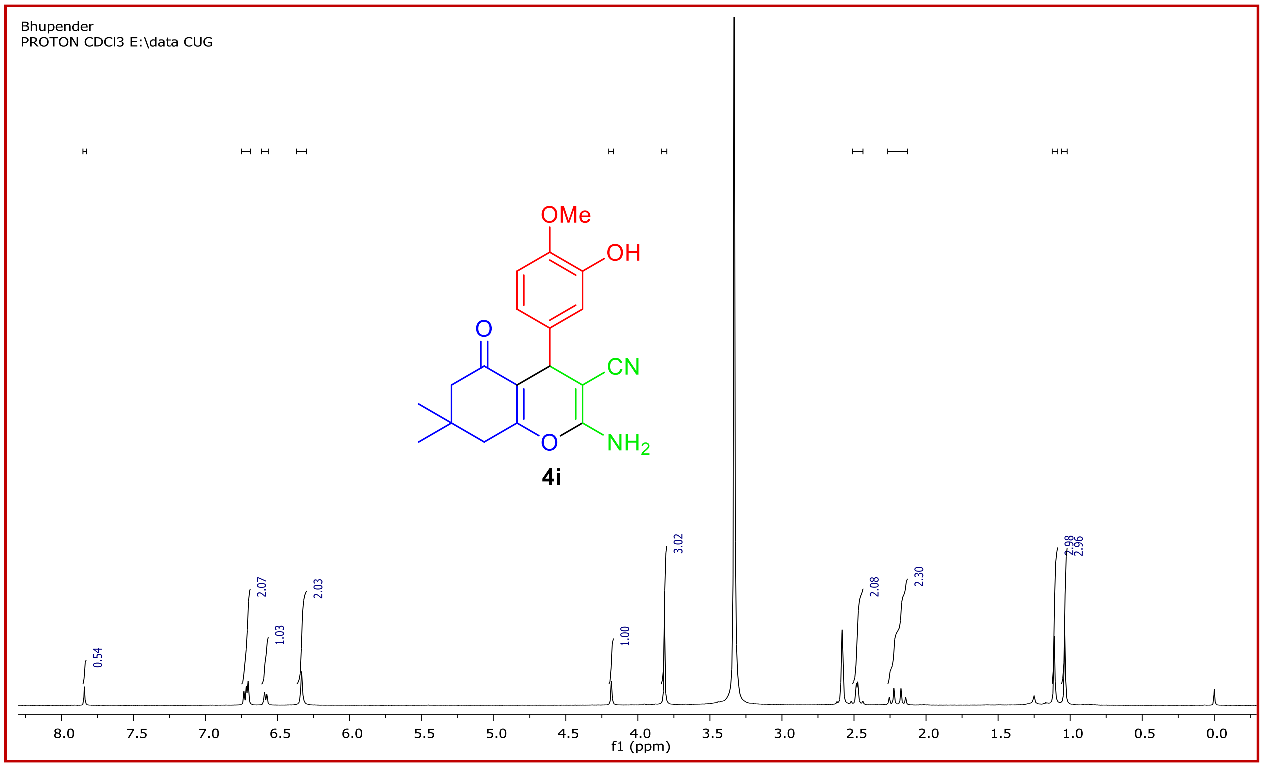


**Figure S21**: $^{1}$H NMR spectra of 2-amino-4-(3-hydroxy-4-methoxyphenyl)-7,7-dimethyl-5-oxo-5,6,7,8-tetrahydro-4*H*-chromene-3-carbonitrile **4i**

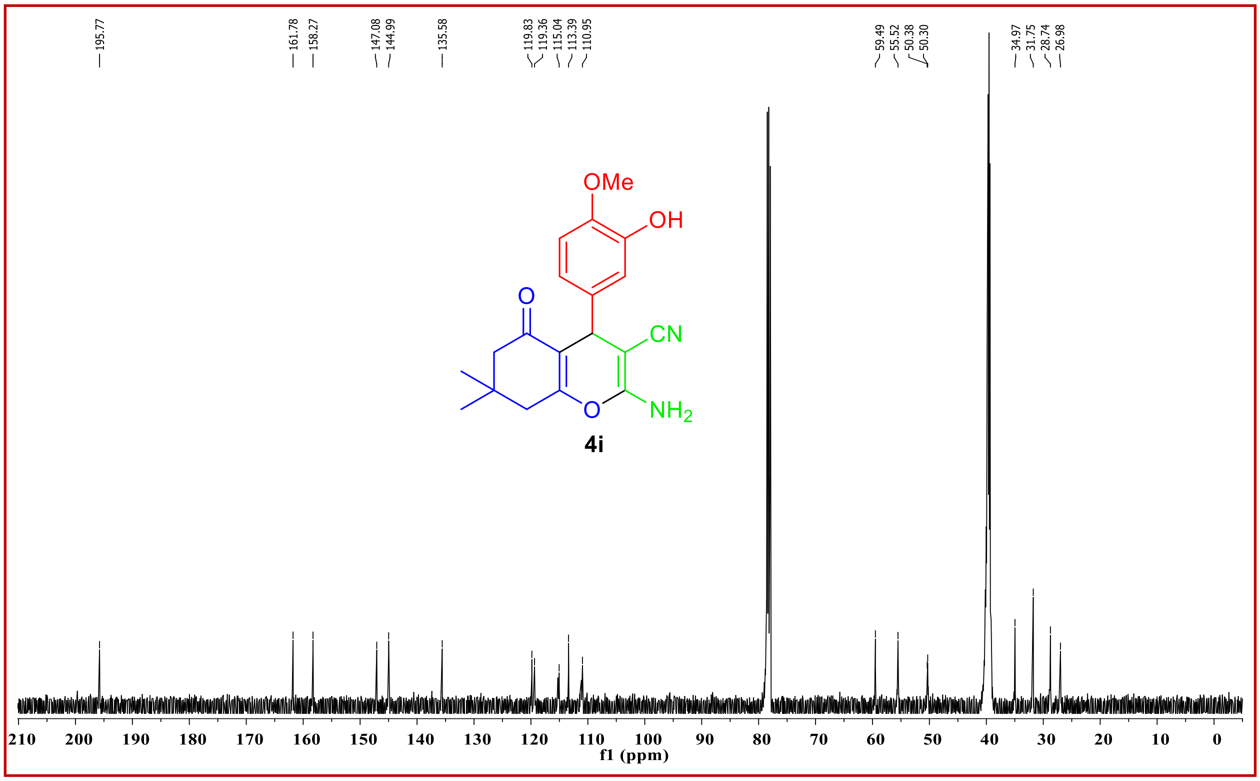


**Figure S22**: $^{13}$C NMR spectra of 2-amino-4-(3-hydroxy-4-methoxyphenyl)-7,7-dimethyl-5-oxo-5,6,7,8-tetrahydro-4*H*-chromene-3-carbonitrile **4i**

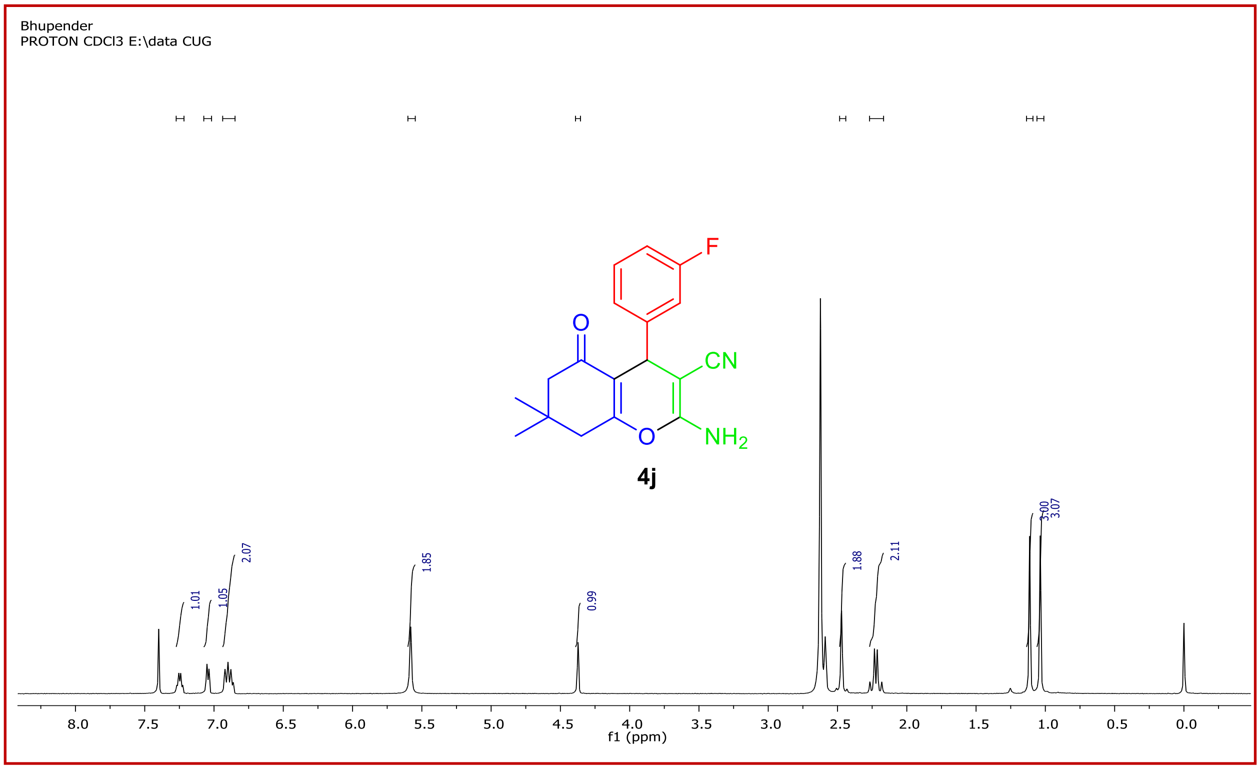


**Figure S23**: $^{1}$H NMR spectra of 2-amino-4-(3-fluorophenyl)-7,7-dimethyl-5-oxo-5,6,7,8-tetrahydro-4*H*-chromene-3-carbonitrile **4j**

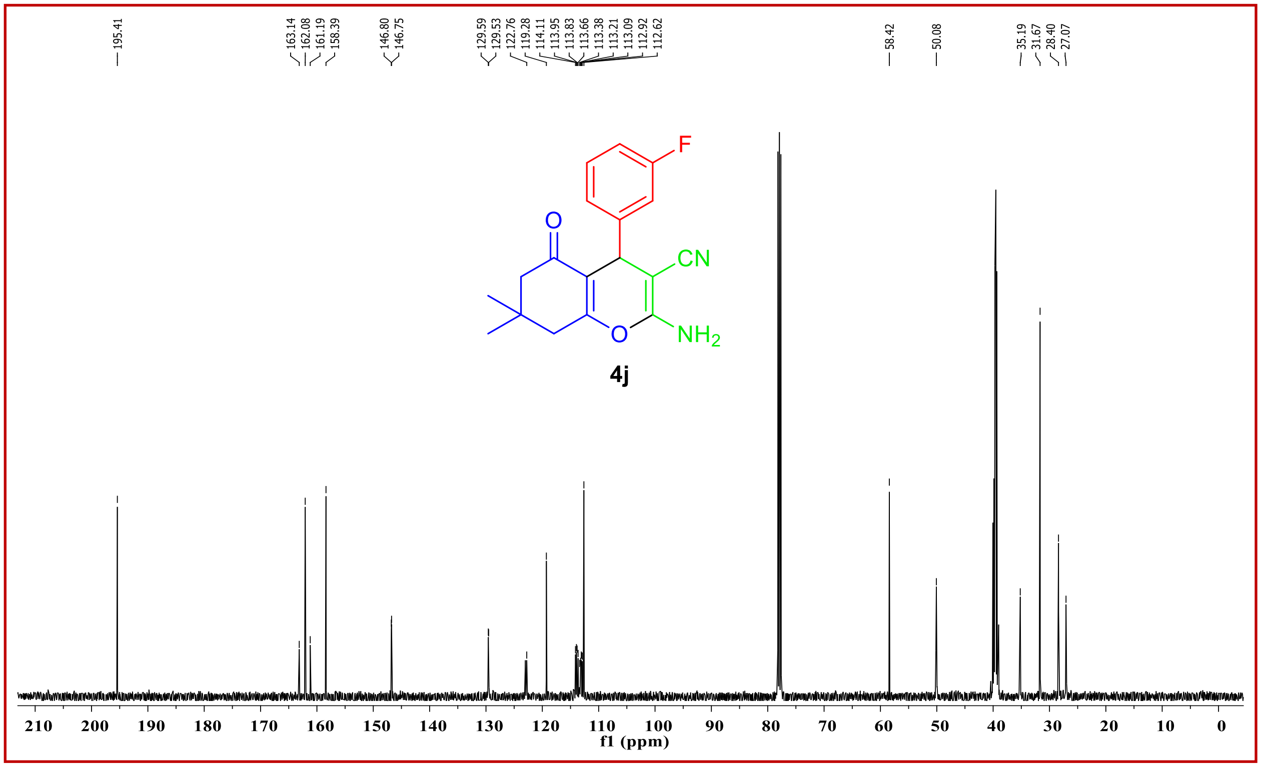


**Figure S24**: $^{13}$C NMR spectra of 2-amino-4-(3-fluorophenyl)-7,7-dimethyl-5-oxo-5,6,7,8-tetrahydro-4*H*-chromene-3-carbonitrile **4j**

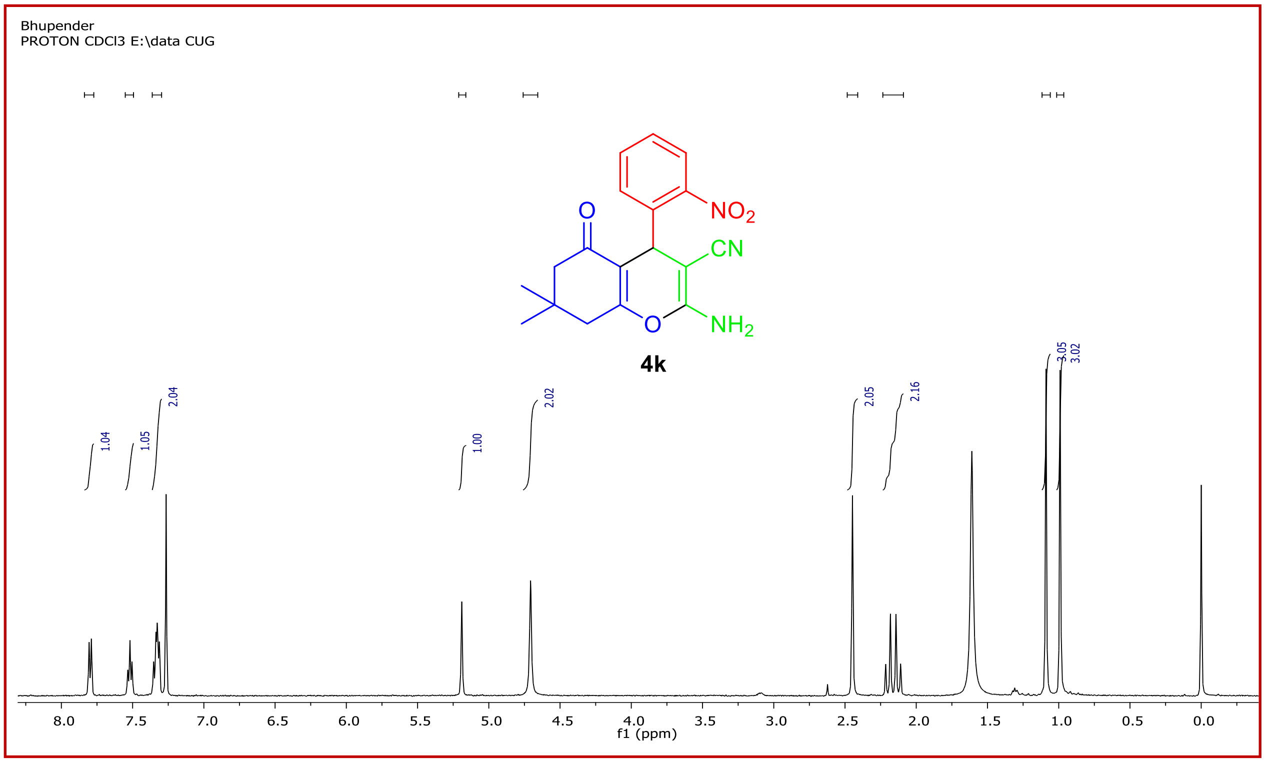


**Figure S25**: $^{1}$H NMR spectra of 2-amino-7,7-dimethyl-4-(2-nitrophenyl)-5-oxo-5,6,7,8-tetrahydro-4*H*-chromene-3-carbonitrile **4k**

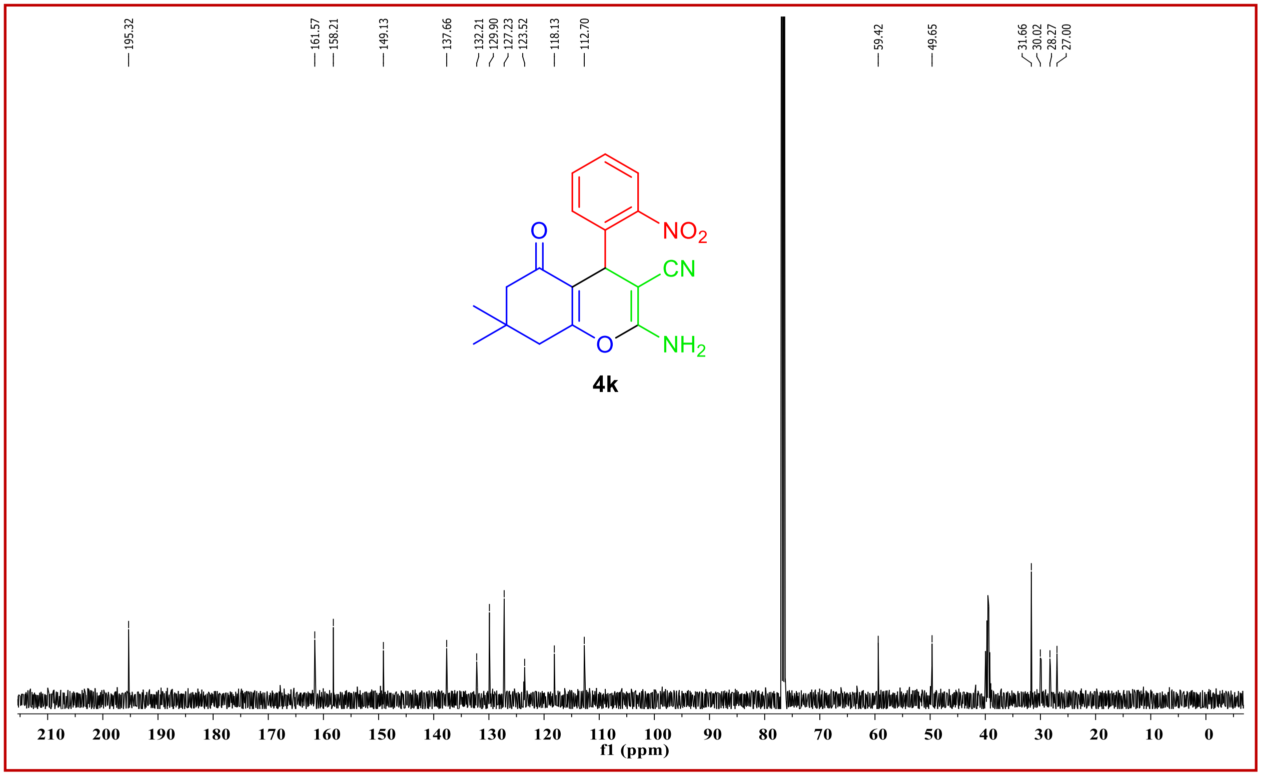


**Figure S26**: $^{13}$C NMR spectra of 2-amino-7,7-dimethyl-4-(2-nitrophenyl)-5-oxo-5,6,7,8-tetrahydro-4*H*-chromene-3-carbonitrile **4k**

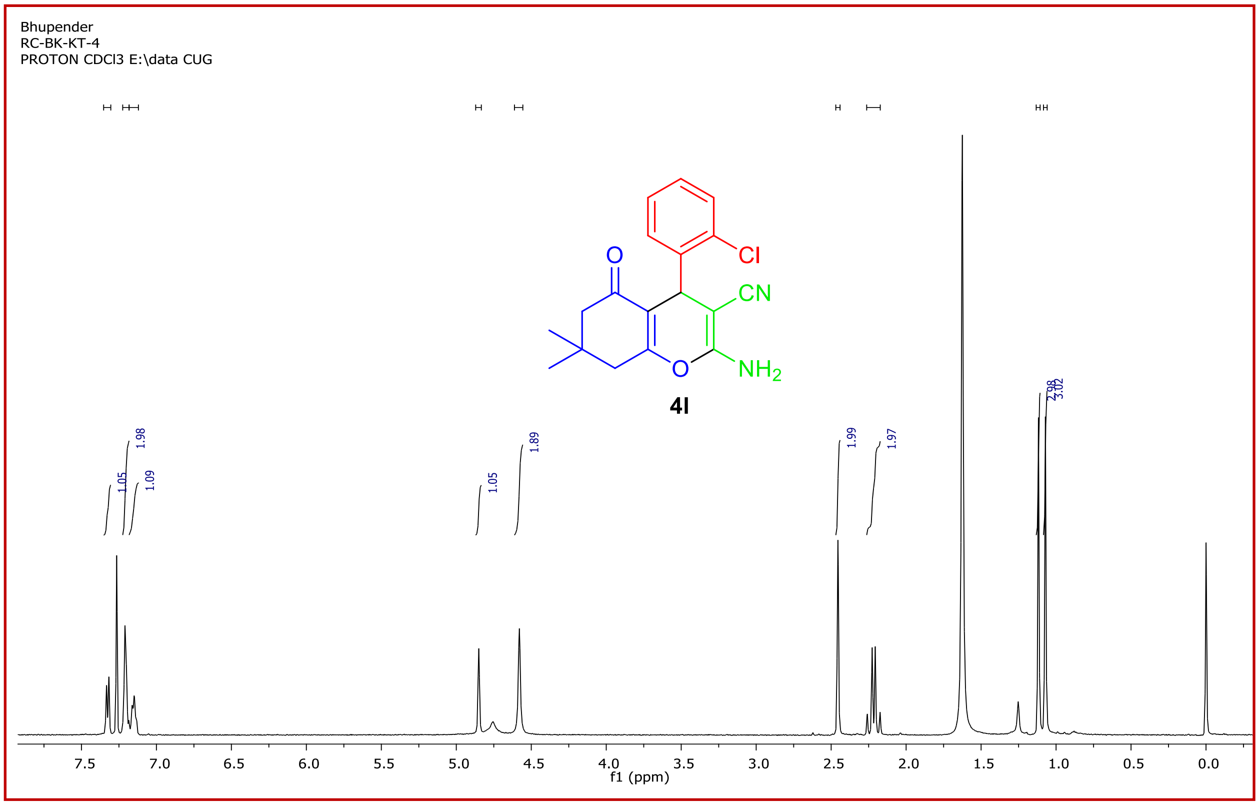


**Figure S27**: $^{1}$H NMR spectra of 2-amino-4-(2-chlorophenyl)-7,7-dimethyl-5-oxo-5,6,7,8-tetrahydro-4*H*-chromene-3-carbonitrile **4l**

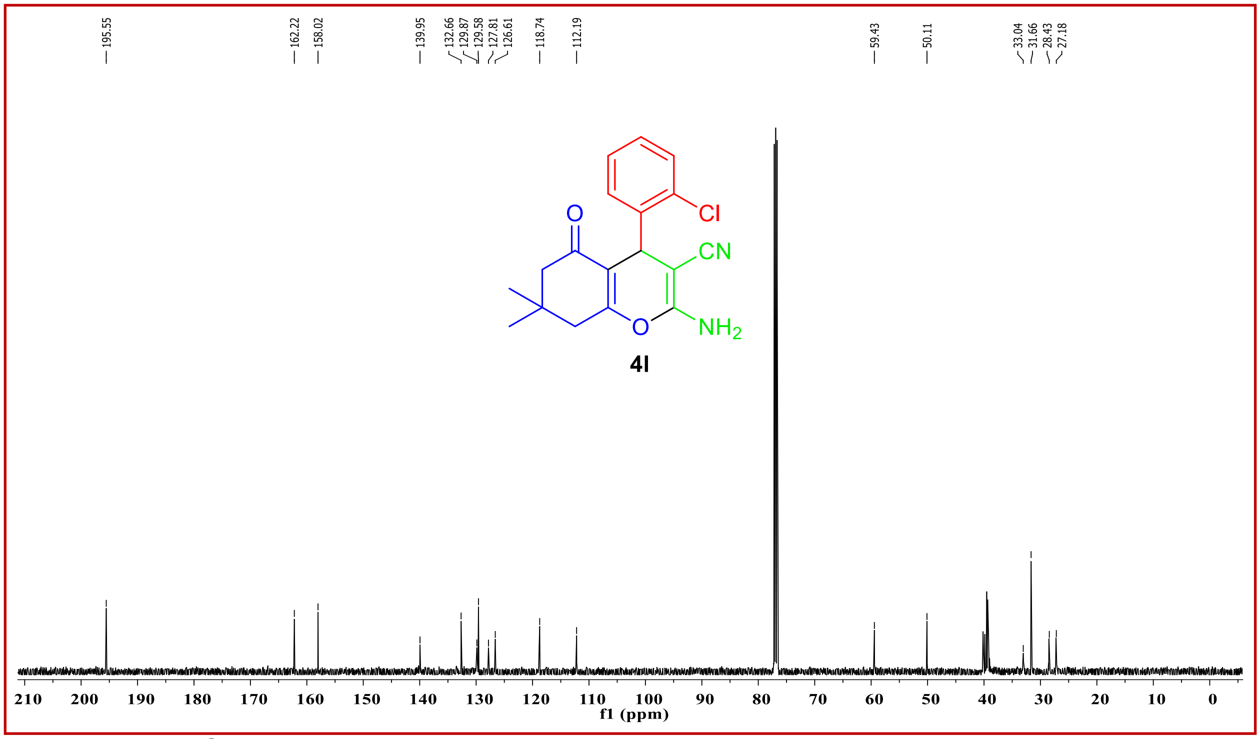


**Figure S28**: $^{13}$C NMR spectra of 2-amino-4-(2-chlorophenyl)-7,7-dimethyl-5-oxo-5,6,7,8-tetrahydro-4*H*-chromene-3-carbonitrile **4l**

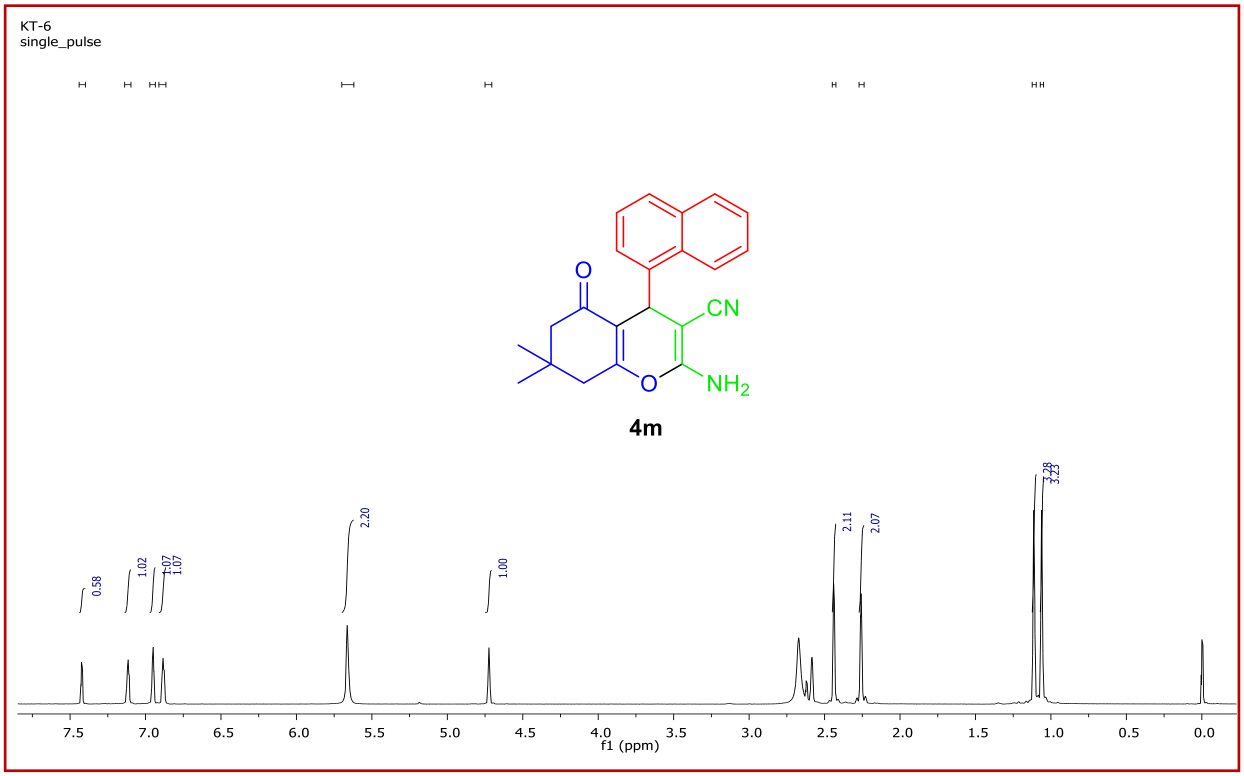


**Figure S29**: $^{1}$H NMR spectra of 2-amino-7,7-dimethyl-4-(naphthalen-2-yl)-5-oxo-5,6,7,8-tetrahydro-4*H*-chromene-3-carbonitrile **4m**

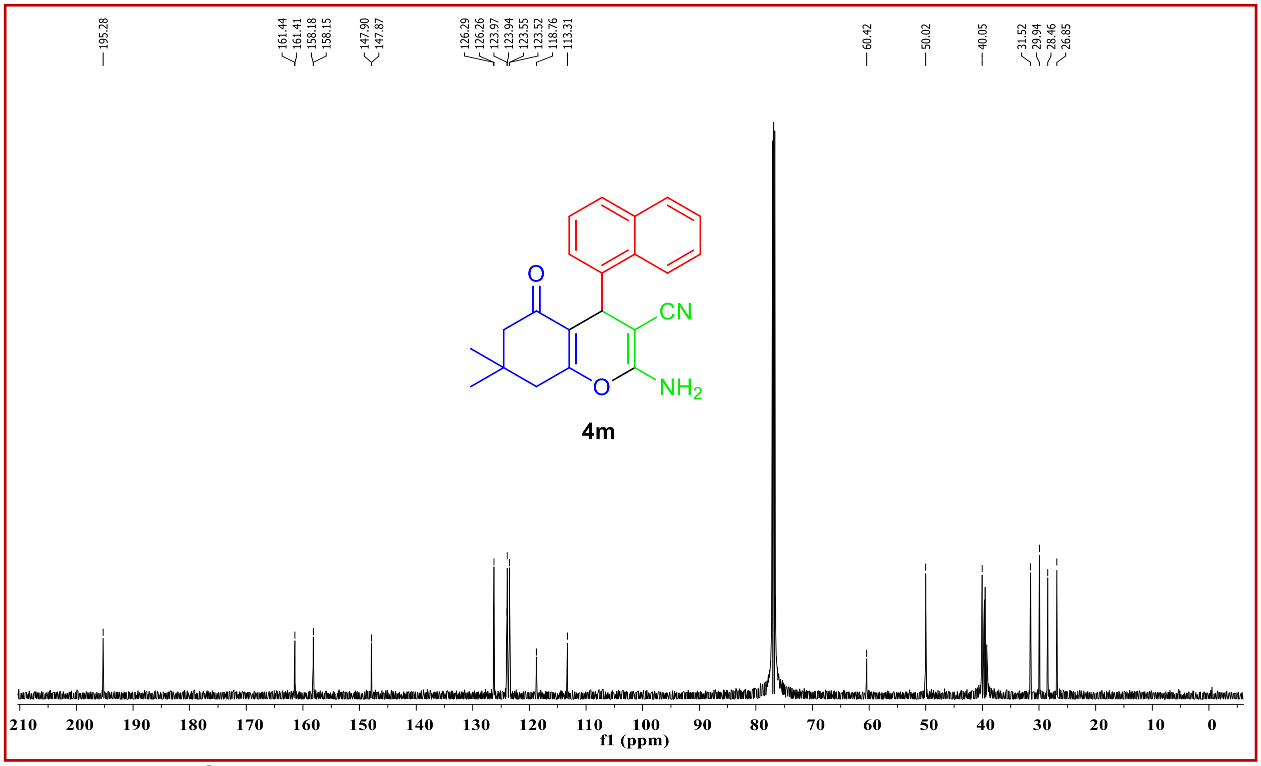


**Figure S30**: $^{13}$C NMR spectra of 2-amino-7,7-dimethyl-4-(naphthalen-2-yl)-5-oxo-5,6,7,8-tetrahydro-4*H*-chromene-3-carbonitrile **4m**

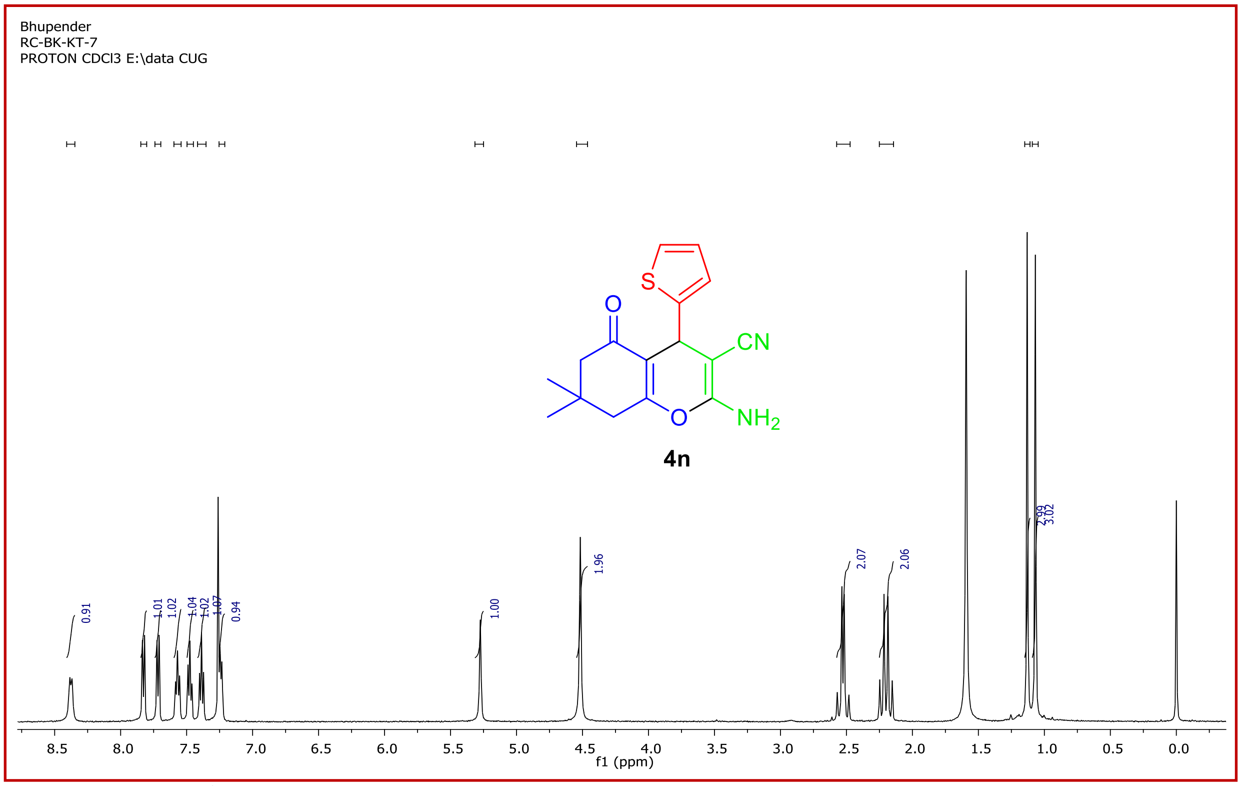


**Figure S31**: $^{1}$H NMR spectra of 2-amino-7,7-dimethyl-5-oxo-4-(thiophen-2-yl)-5,6,7,8-tetrahydro-4*H*-chromene-3-carbonitrile **4n**

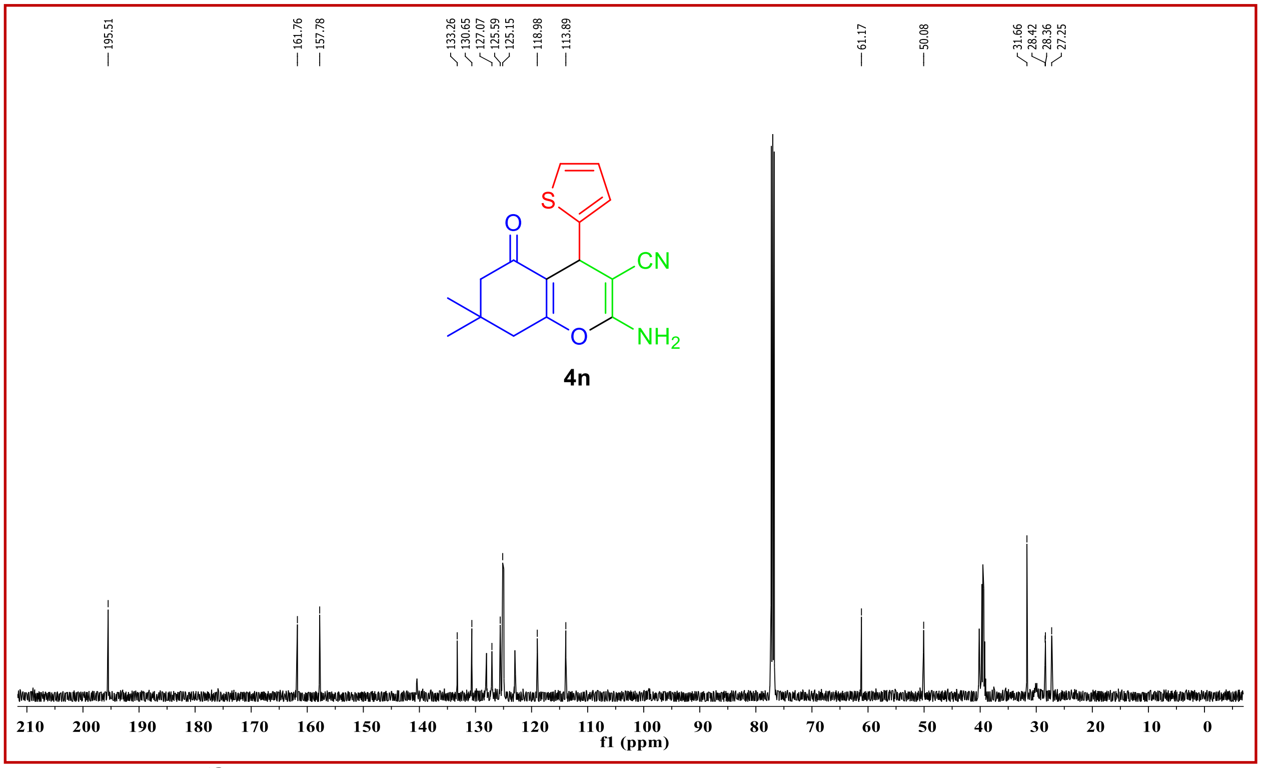


**Figure S32**: $^{13}$C NMR spectra of 2-amino-7,7-dimethyl-5-oxo-4-(thiophen-2-yl)-5,6,7,8-tetrahydro-4*H*-chromene-3-carbonitrile **4n**

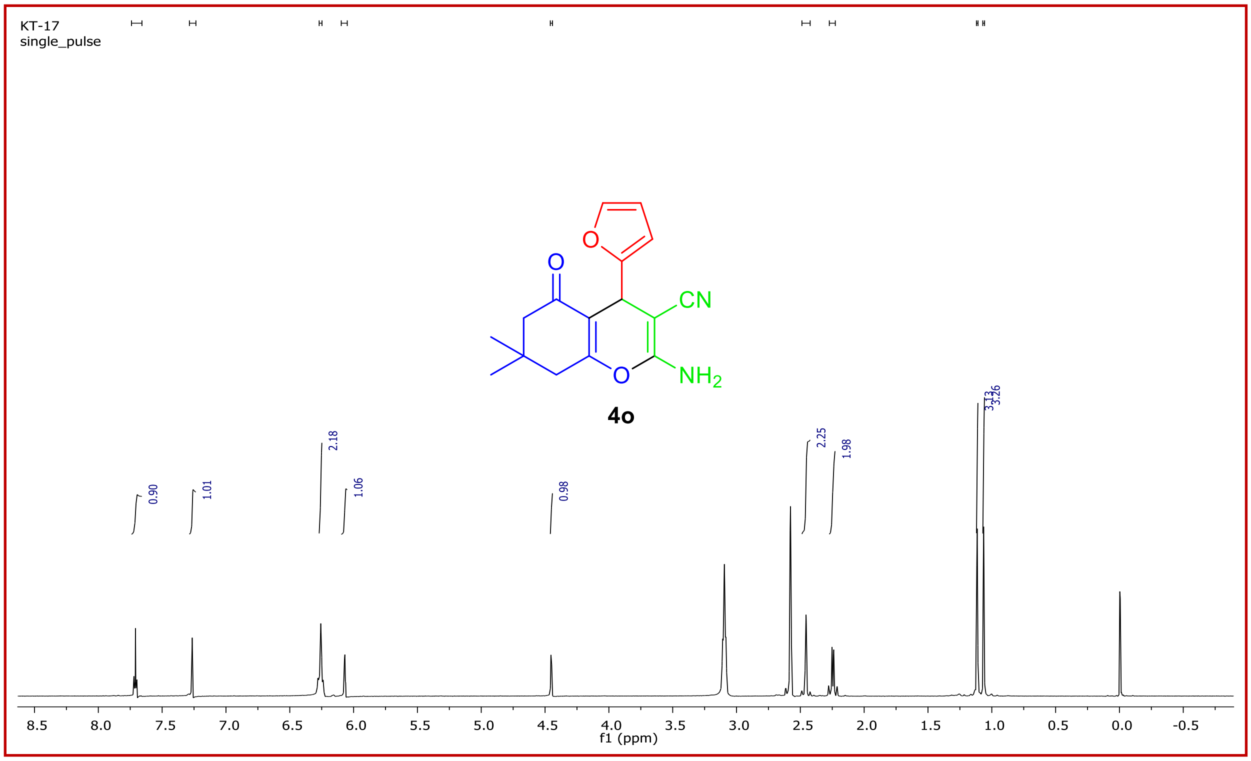


**Figure S33**: $^1$H NMR spectra of 2-amino-4-(furan-2-yl)-7,7-dimethyl-5-oxo-5,6,7,8-tetrahydro-4*H*-chromene-3-carbonitrile **4o**

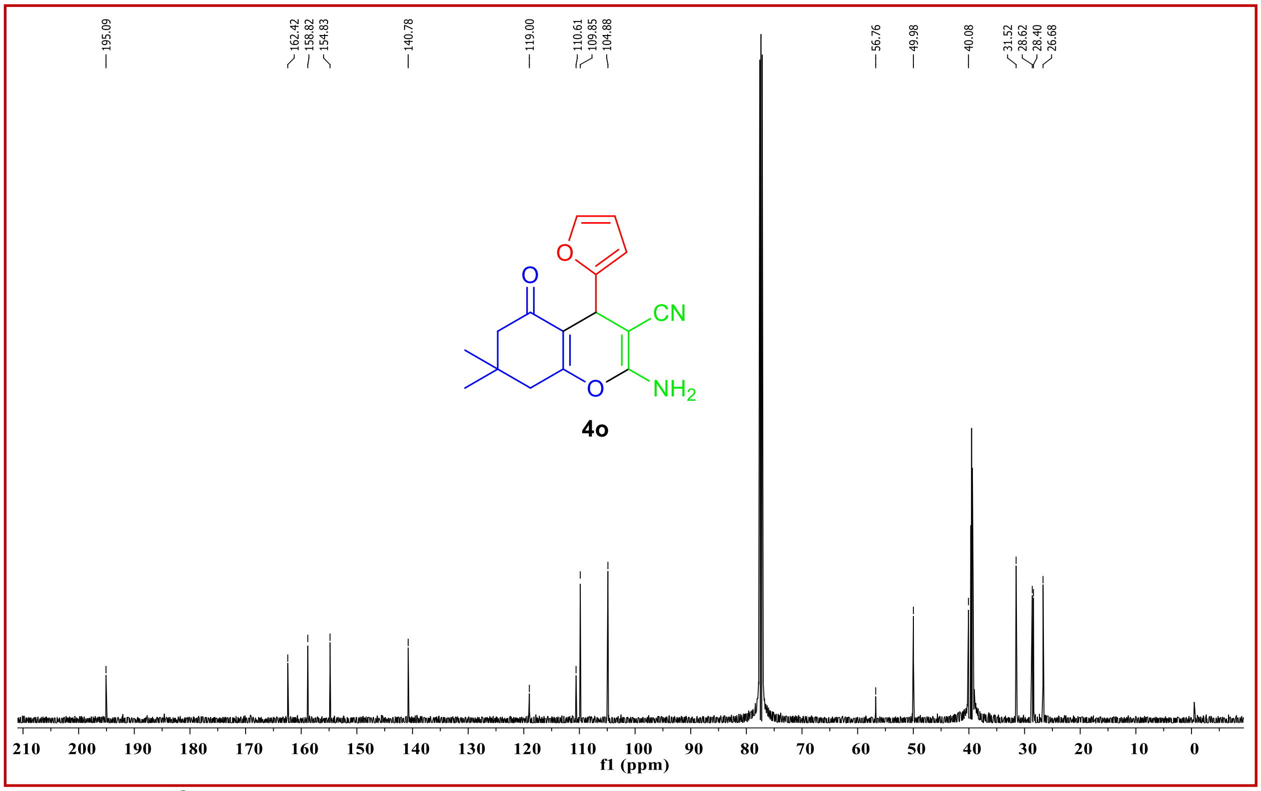


**Figure S34**: $^{13}$C NMR spectra of 2-amino-4-(furan-2-yl)-7,7-dimethyl-5-oxo-5,6,7,8-tetrahydro-4*H*-chromene-3-carbonitrile **4o**

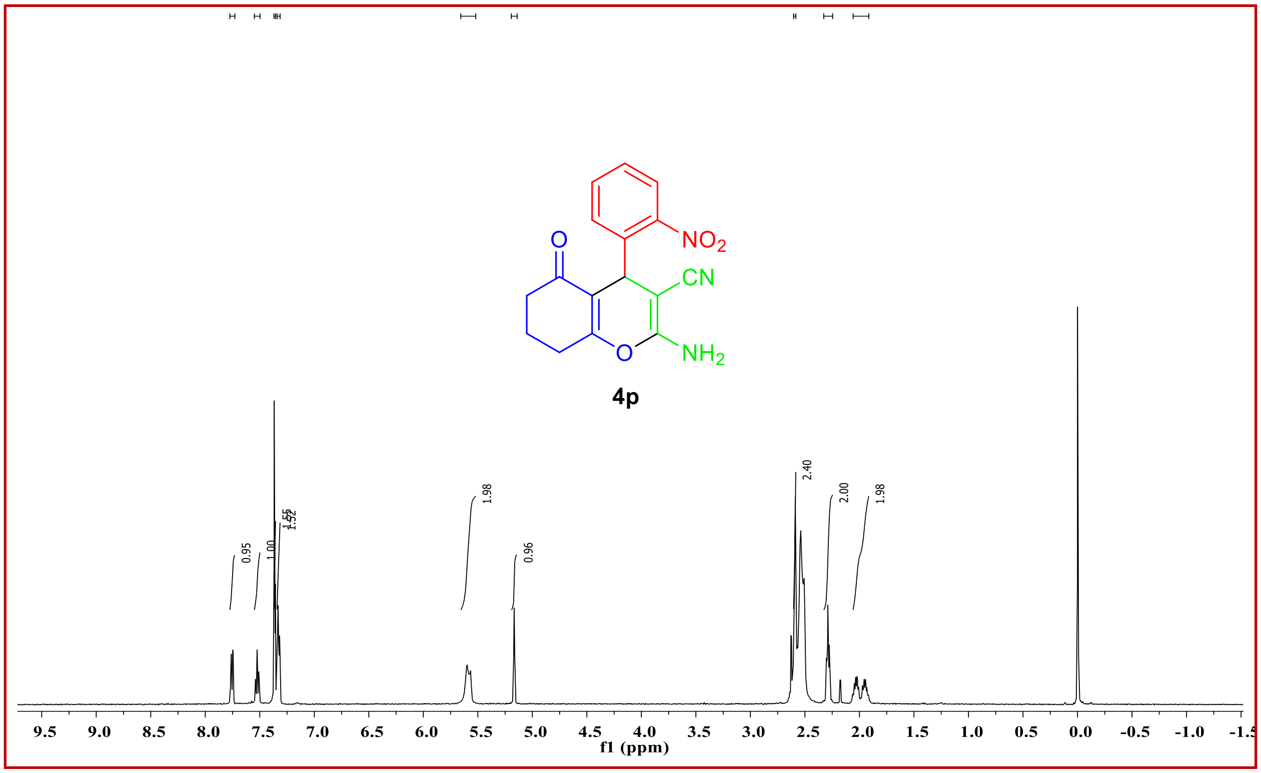


**Figure S35**: $^{1}$H NMR spectra of 2-amino-4-(2-nitrophenyl)-5-oxo-5,6,7,8-tetrahydro-4*H*-chromene-3-carbonitrile **4p**

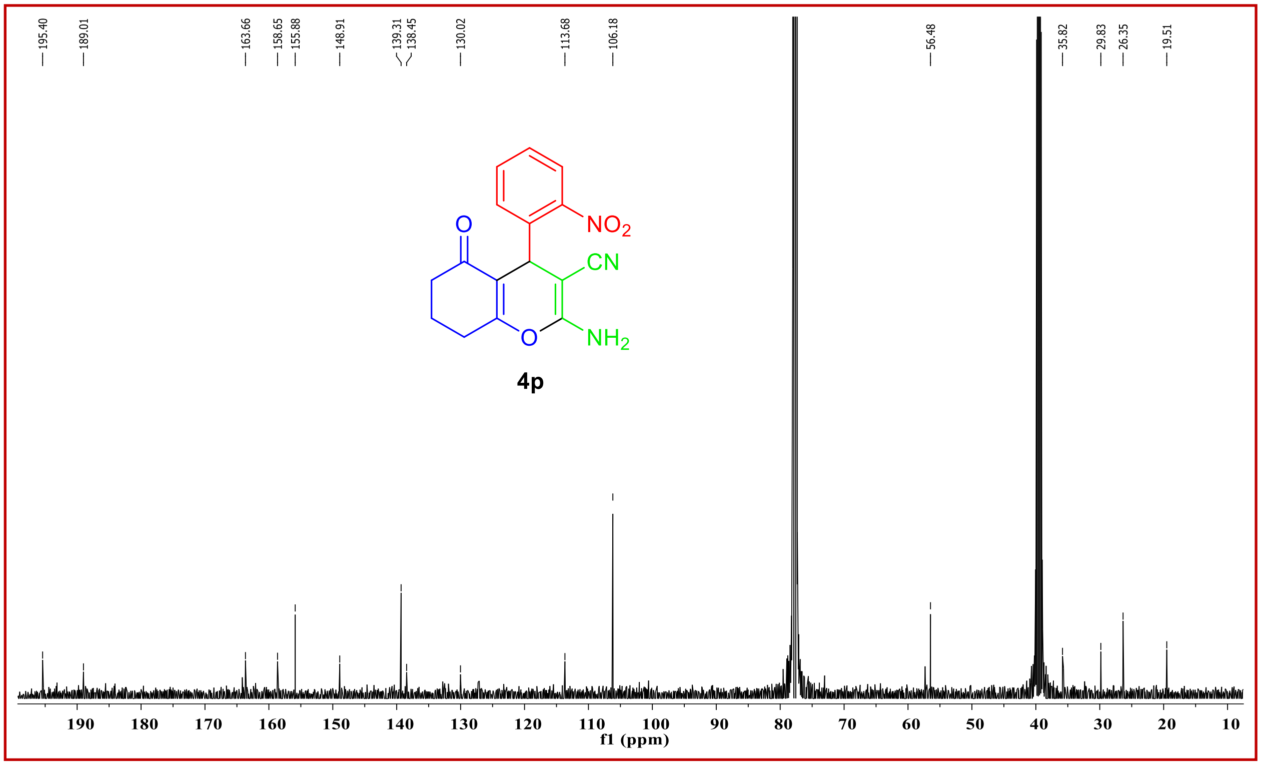


**Figure S36**: $^{13}$C NMR spectra of 2-amino-4-(2-nitrophenyl)-5-oxo-5,6,7,8-tetrahydro-4*H*-chromene-3-carbonitrile **4p**

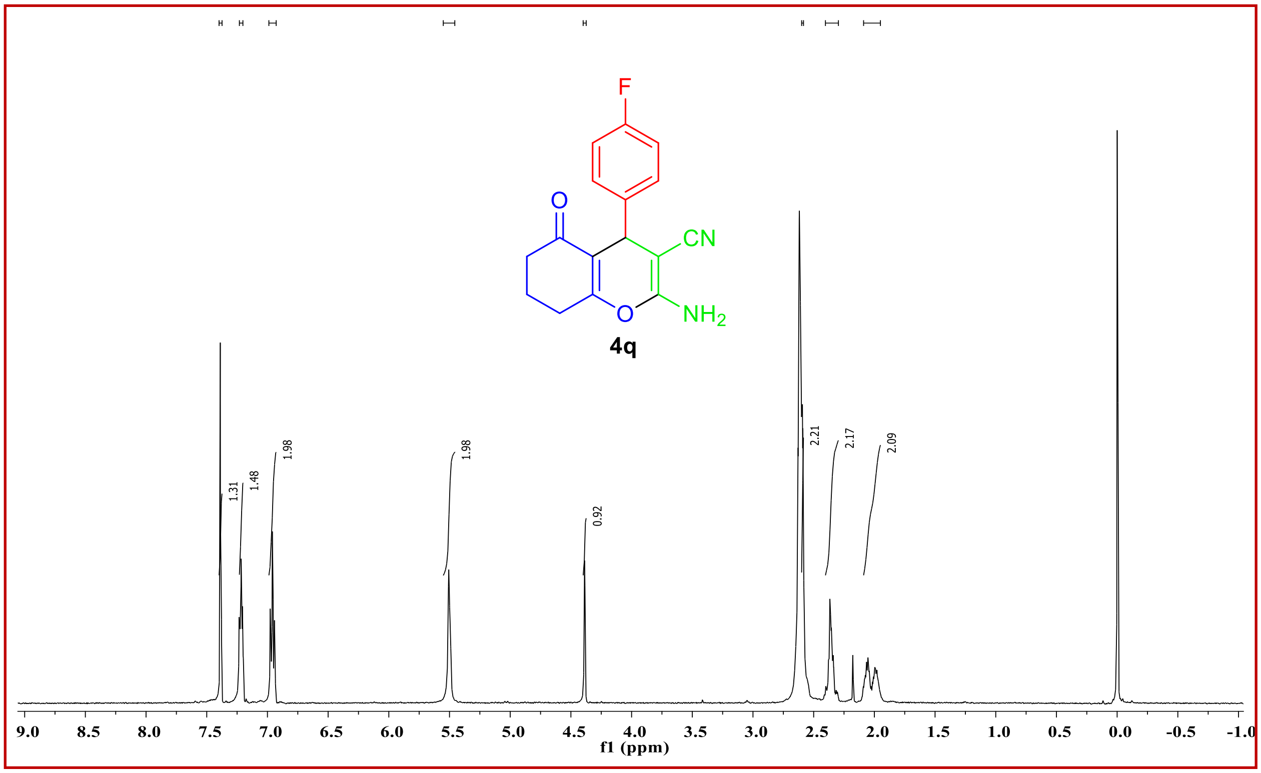


**Figure S37**: $^{1}$H NMR spectra of 2-amino-4-(4-fluorophenyl)-5-oxo-5,6,7,8-tetrahydro-4*H*-chromene-3-carbonitrile **4q**

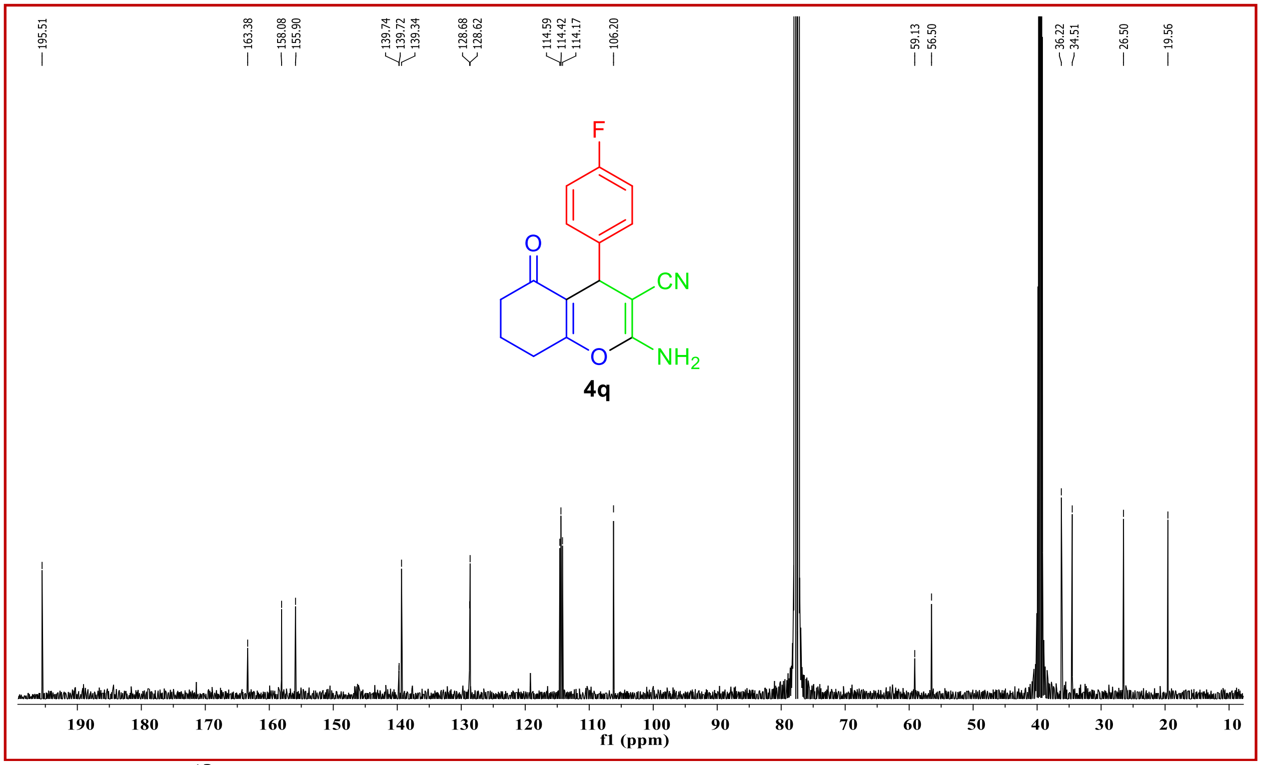


**Figure S38**: $^{13}$C NMR spectra of 2-amino-4-(4-fluorophenyl)-5-oxo-5,6,7,8-tetrahydro-4*H*-chromene-3-carbonitrile **4q**

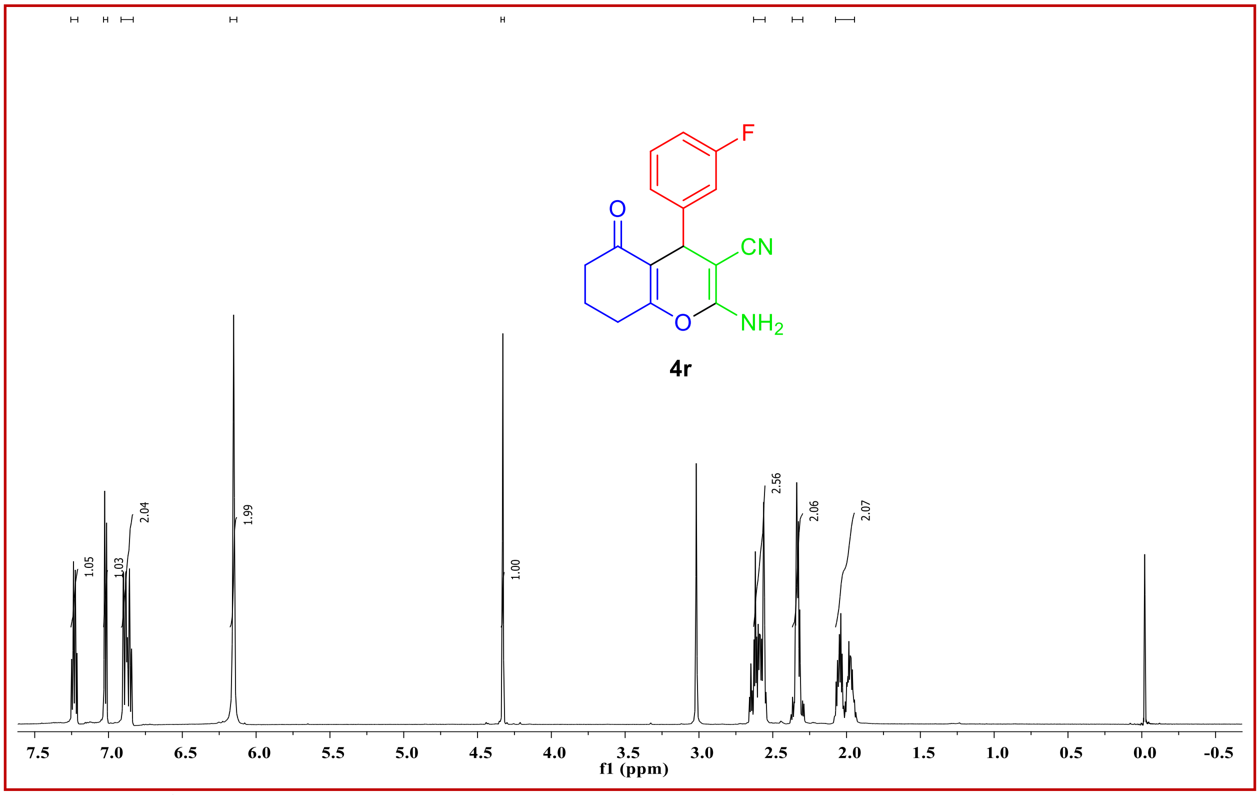

**Figure S39**: $^{1}$H NMR spectra of 2-amino-4-(3-fluorophenyl)-5-oxo-5,6,7,8-tetrahydro-4H-chromene-3-carbonitrile **4r**

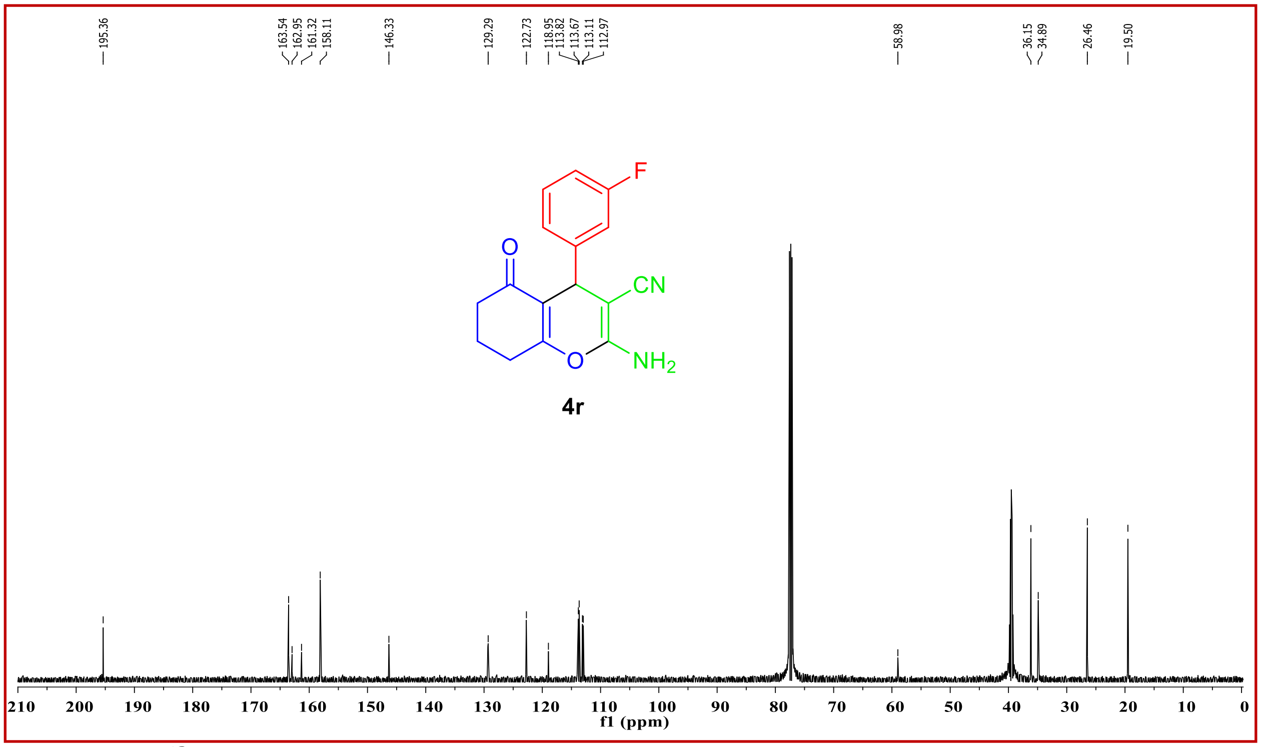

**Figure S40**: $^{13}$C NMR spectra of 2-amino-4-(3-fluorophenyl)-5-oxo-5,6,7,8-tetrahydro-4H-chromene-3-carbonitrile **4r**

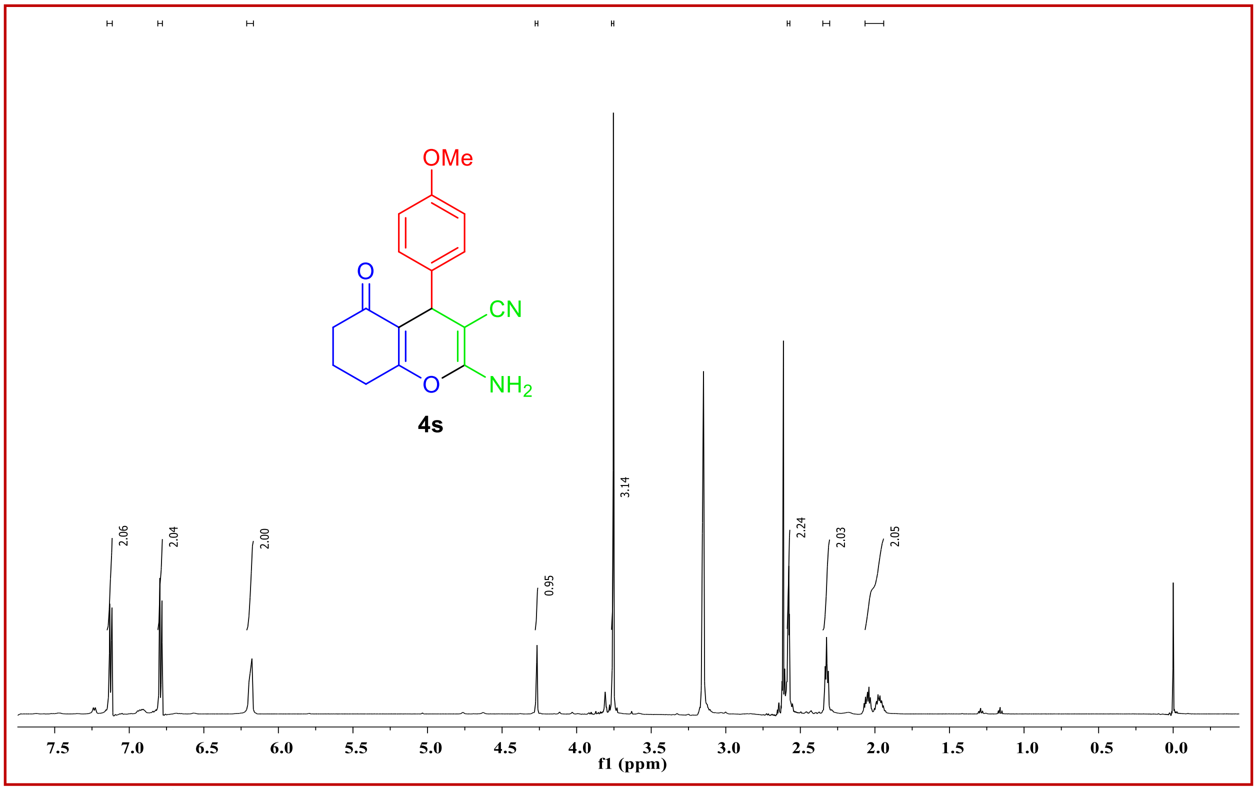


**Figure S41**: $^{1}$H NMR spectra of 2-amino-4-(4-methoxyphenyl)-5-oxo-5,6,7,8-tetrahydro-4*H*-chromene-3-carbonitrile **4s**

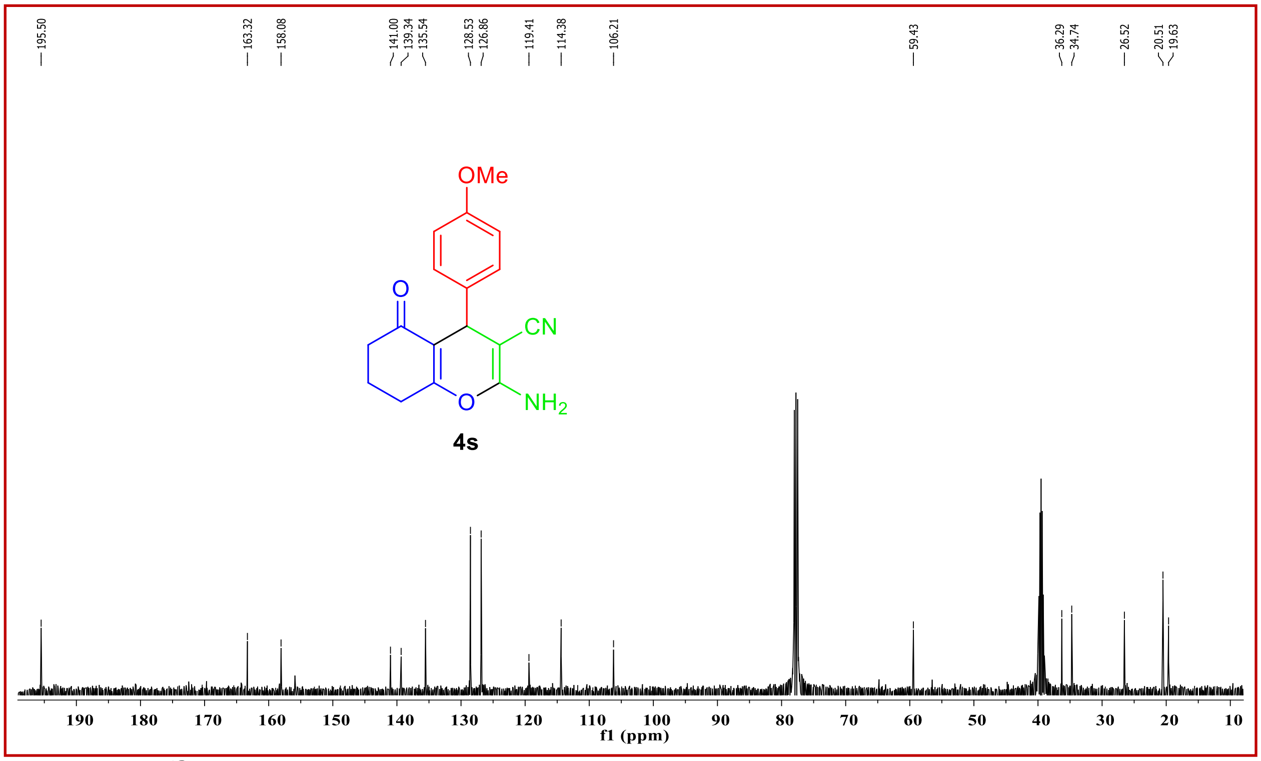


**Figure S42**: $^{13}$C NMR spectra of 2-amino-4-(4-methoxyphenyl)-5-oxo-5,6,7,8-tetrahydro-4*H*-chromene-3-carbonitrile **4s**

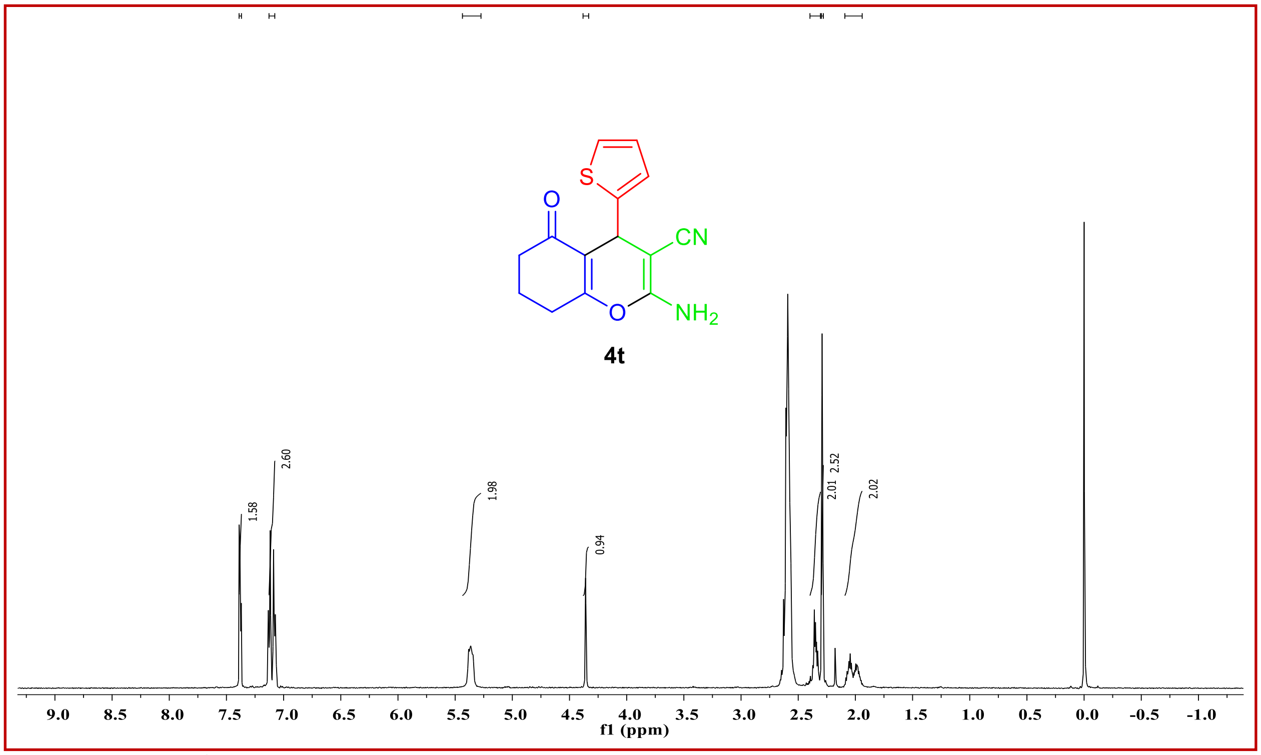


**Figure S43**: $^1$H NMR spectra of 2-amino-5-oxo-4-(thiophen-2-yl)-5,6,7,8-tetrahydro-4H-chromene-3-carbonitrile **4t**

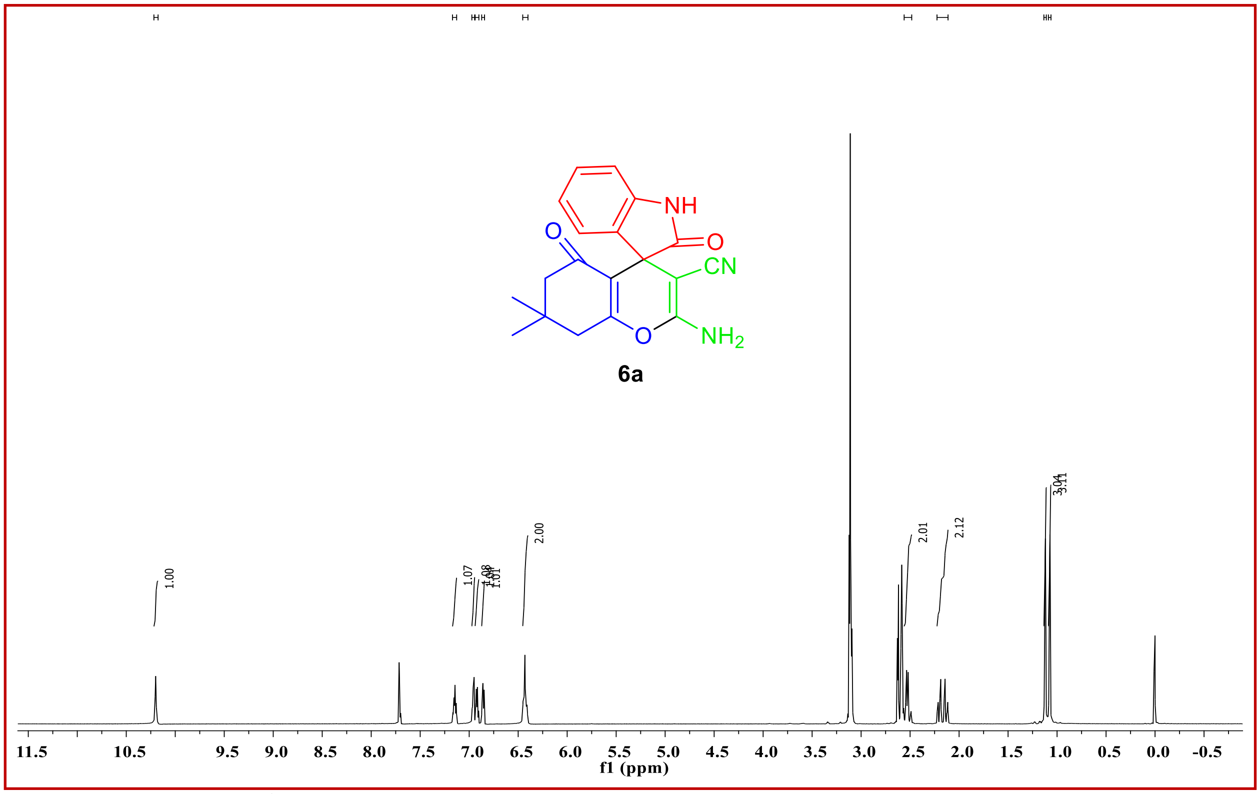


**Figure S44**: $^1$H NMR spectra of 2-amino-7,7-dimethyl-2',5-dioxo-5,6,7,8-tetrahydrospiro[chromene-4,3'-indoline]-3-carbonitrile **6a**

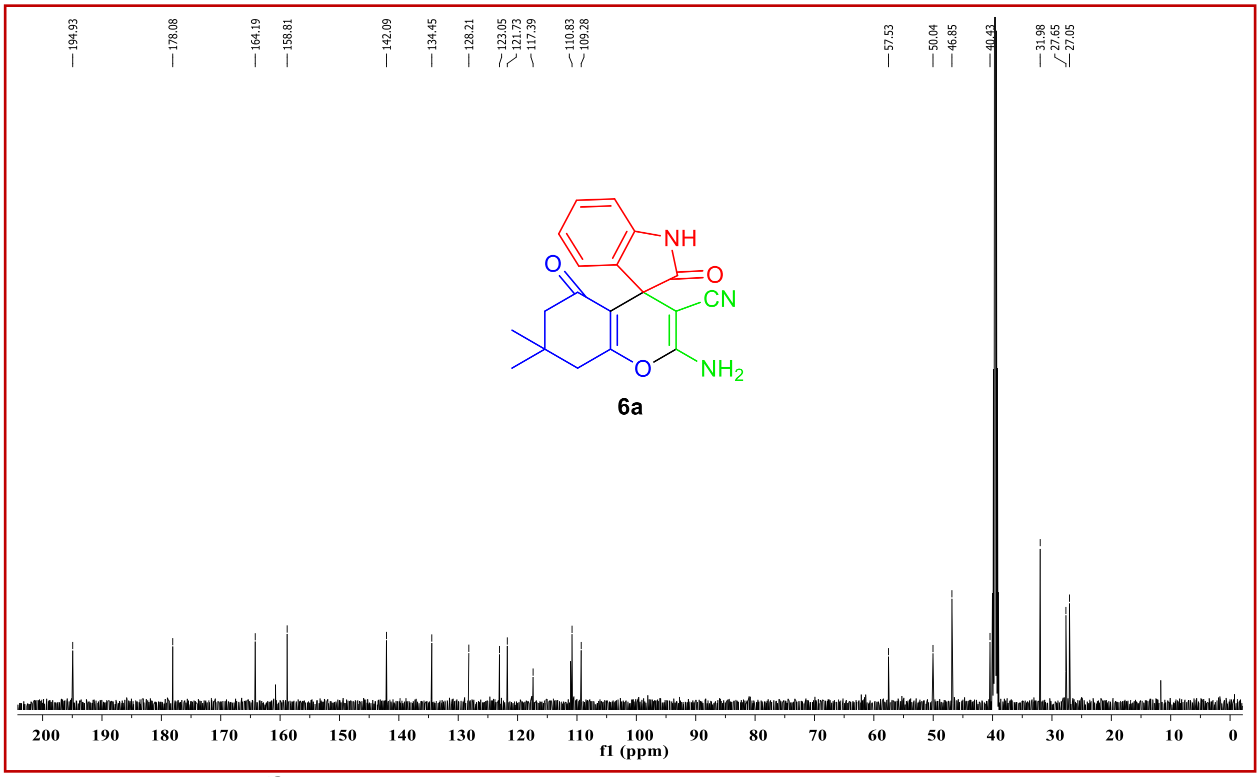


**Figure S45**: $^{13}$C NMR spectra of 2-amino-7,7-dimethyl-2',5-dioxo-5,6,7,8-tetrahydrospiro[chromene-4,3'-indoline]-3-carbonitrile **6a**

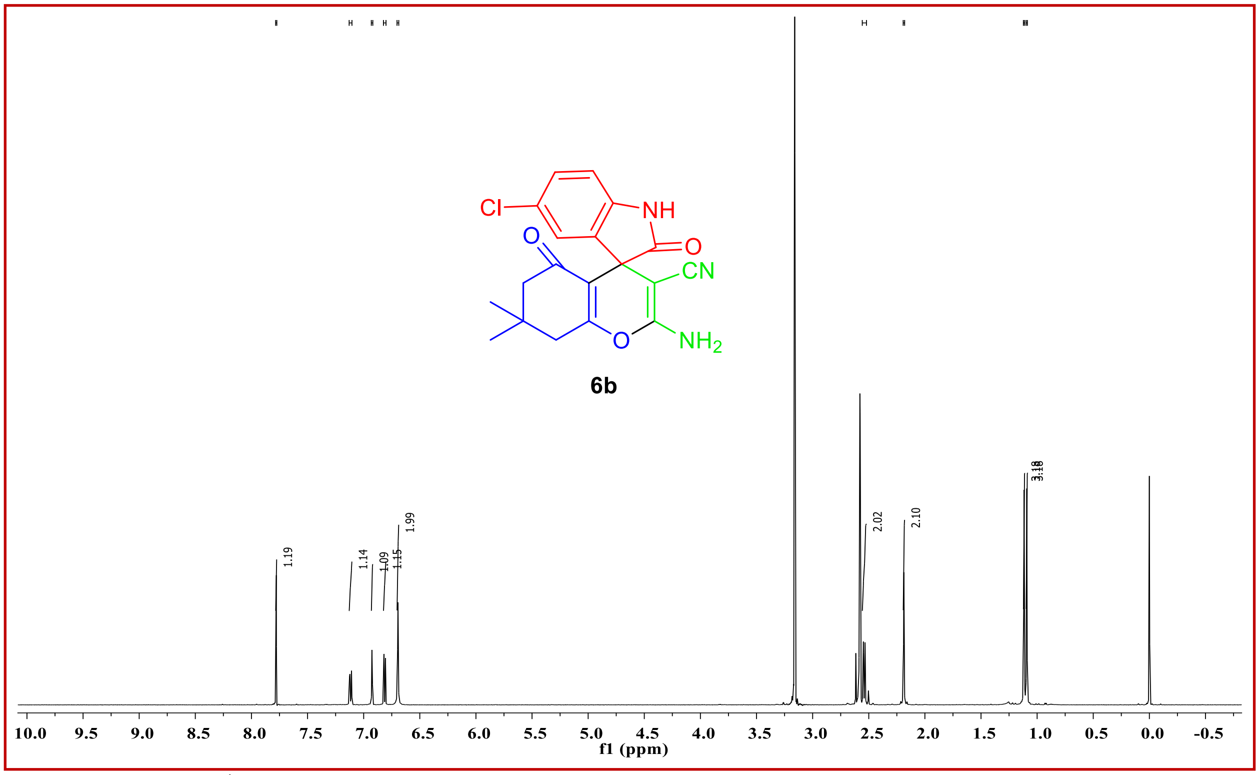


**Figure S46**: $^{1}$H NMR spectra of 2-amino-5'-chloro-7,7-dimethyl-2',5-dioxo-5,6,7,8-tetrahydrospiro[chromene-4,3'-indoline]-3-carbonitrile **6b**

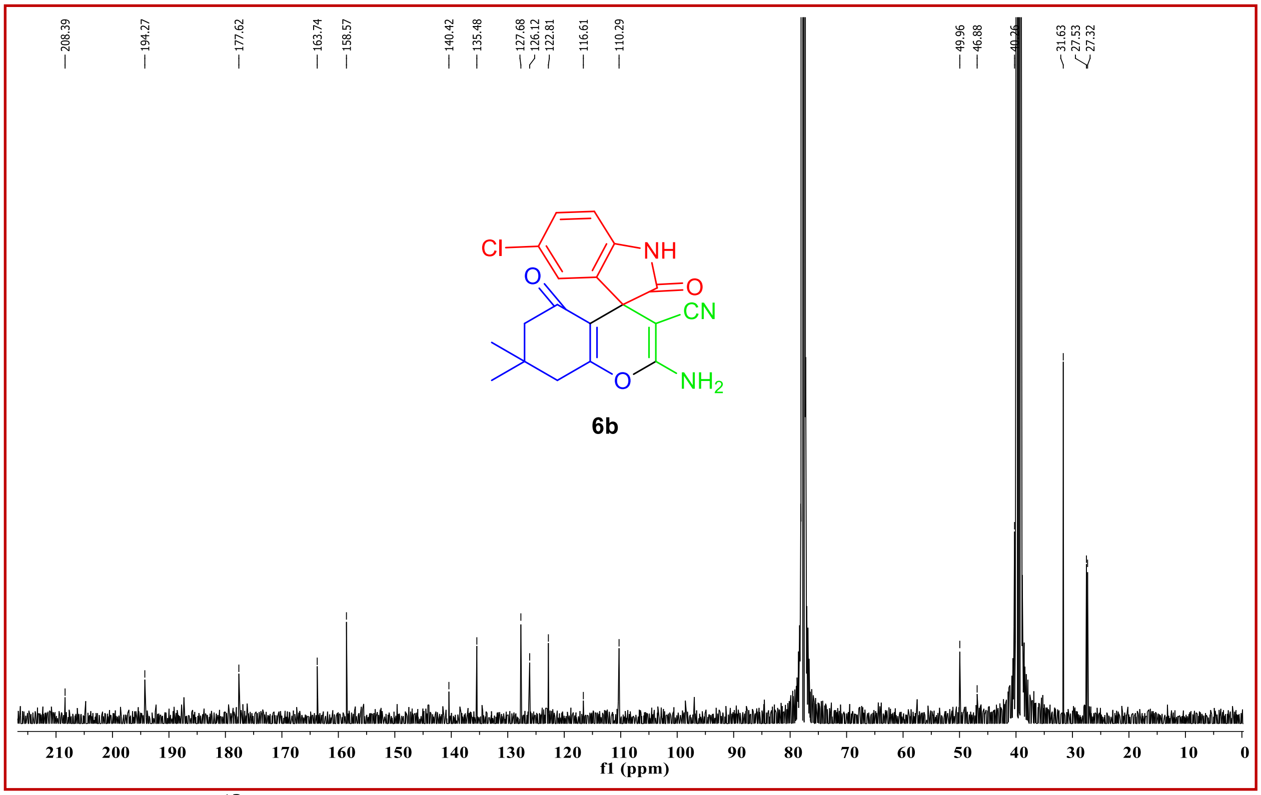

**Figure S47**: $^{13}C$ NMR spectra of 2-amino-5'-chloro-7,7-dimethyl-2',5-dioxo-5,6,7,8-tetrahydrospiro[chromene-4,3'-indoline]-3-carbonitrile **6b**

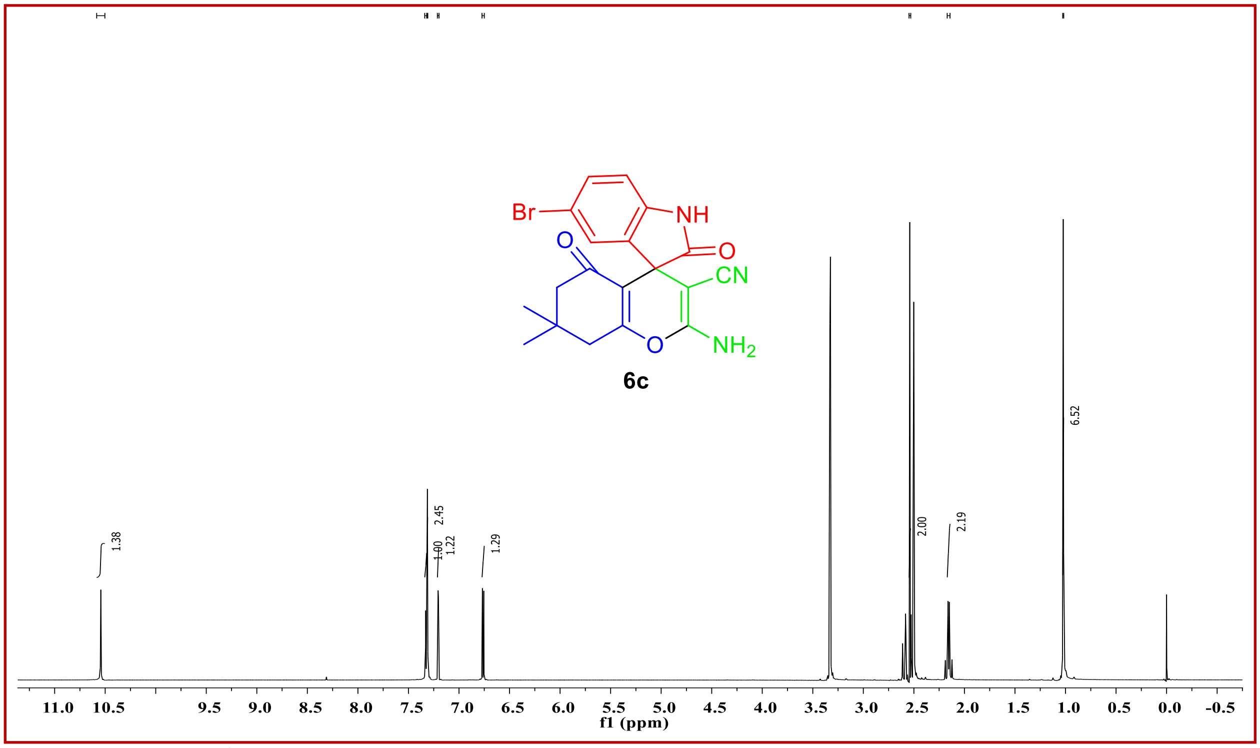

**Figure S48**: $^{1}H$ NMR spectra of 2-amino-5'-bromo-7,7-dimethyl-2',5-dioxo-5,6,7,8-tetrahydrospiro[chromene-4,3'-indoline]-3-carbonitrile **6c**

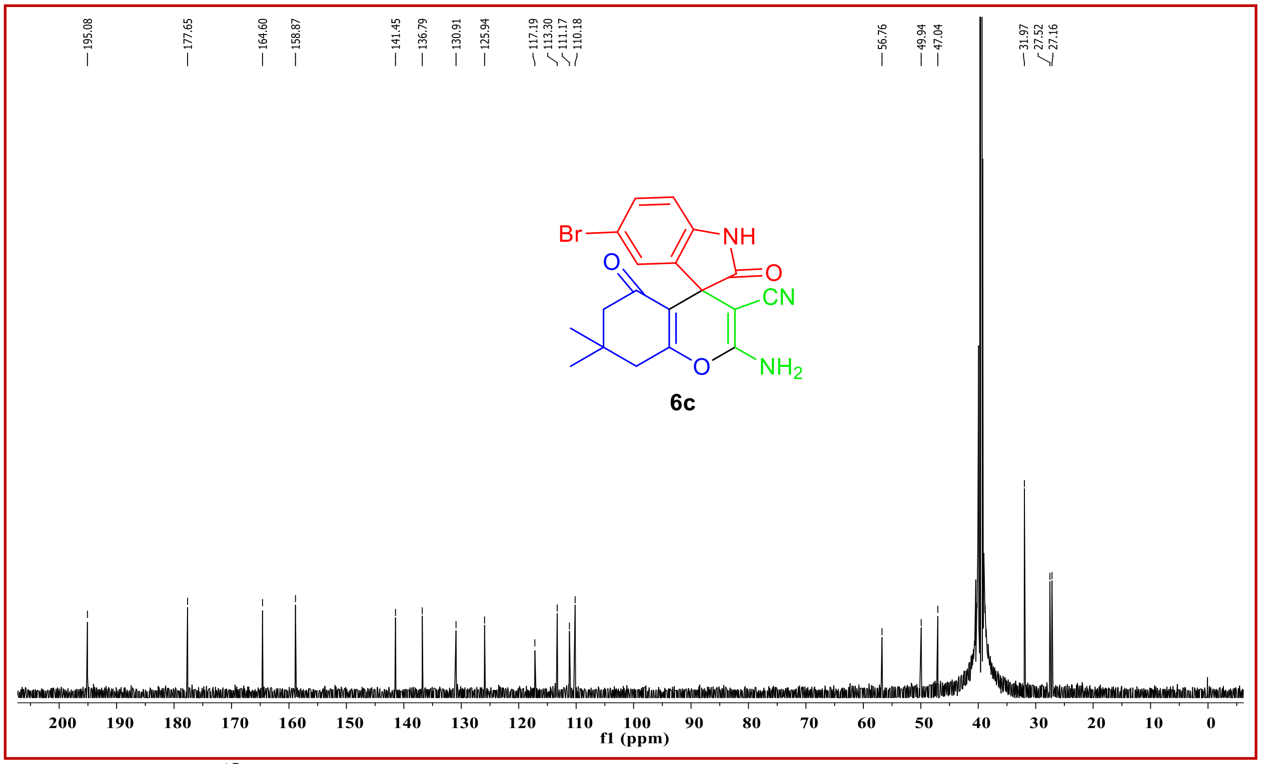


**Figure S49**: $^{13}C$ NMR spectra of 2-amino-5'-bromo-7,7-dimethyl-2',5-dioxo-5,6,7,8-tetrahydrospiro[chromene-4,3'-indoline]-3-carbonitrile **6c**

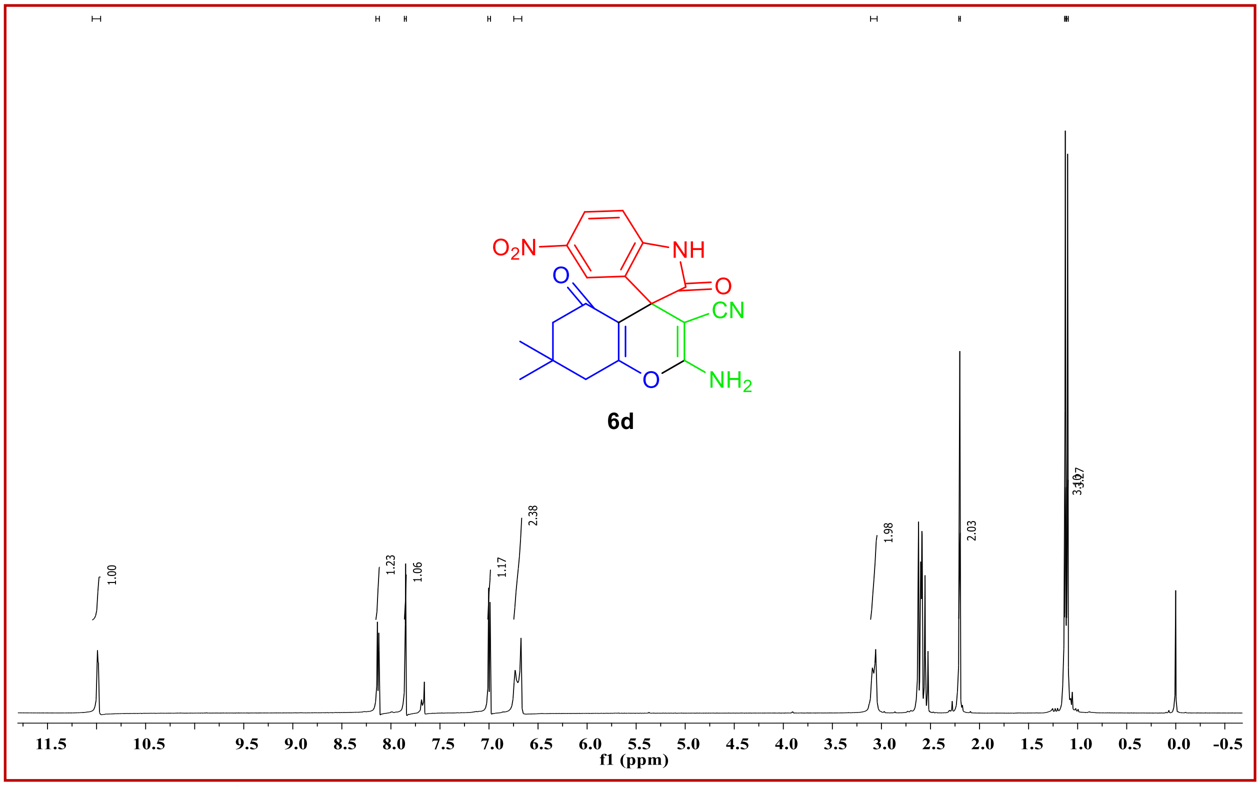


**Figure S50**: $^{1}H$ NMR spectra of 2-amino-7,7-dimethyl-5'-nitro-2',5-dioxo-5,6,7,8-tetrahydrospiro[chromene-4,3'-indoline]-3-carbonitrile **6d**

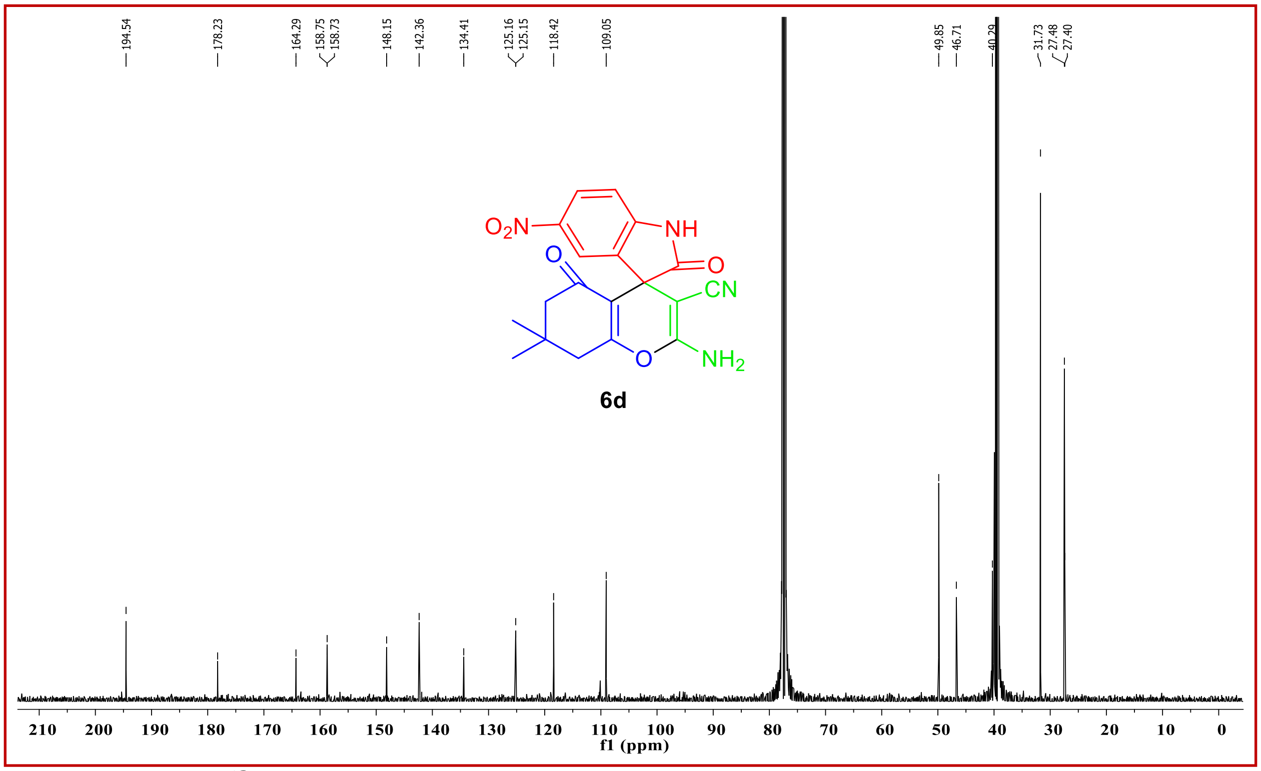

**Figure S51**: $^{13}C$ NMR spectra of 2-amino-7,7-dimethyl-5'-nitro-2',5-dioxo-5,6,7,8-tetrahydrospiro[chromene-4,3'-indoline]-3-carbonitrile **6d**

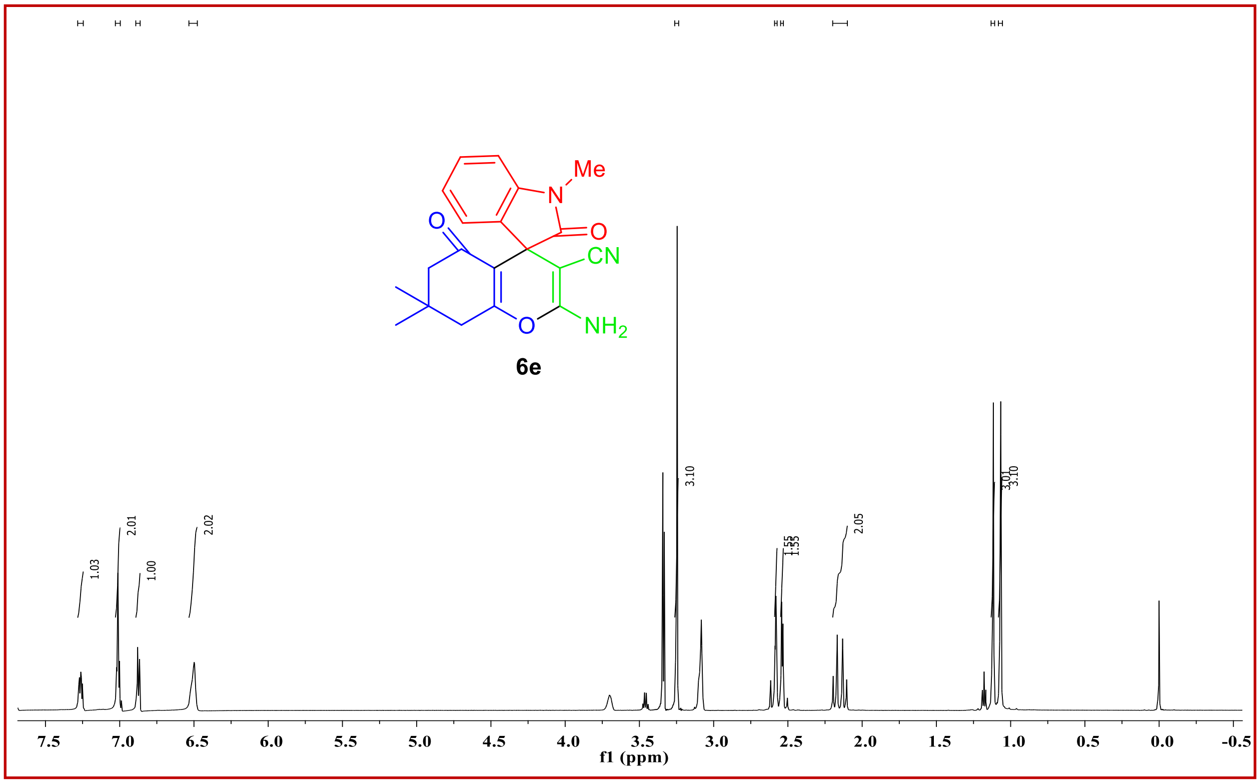

**Figure S52**: $^{1}H$ NMR spectra of 2-amino-1',7,7-trimethyl-2',5-dioxo-5,6,7,8-tetrahydrospiro[chromene-4,3'-indoline]-3-carbonitrile **6e**

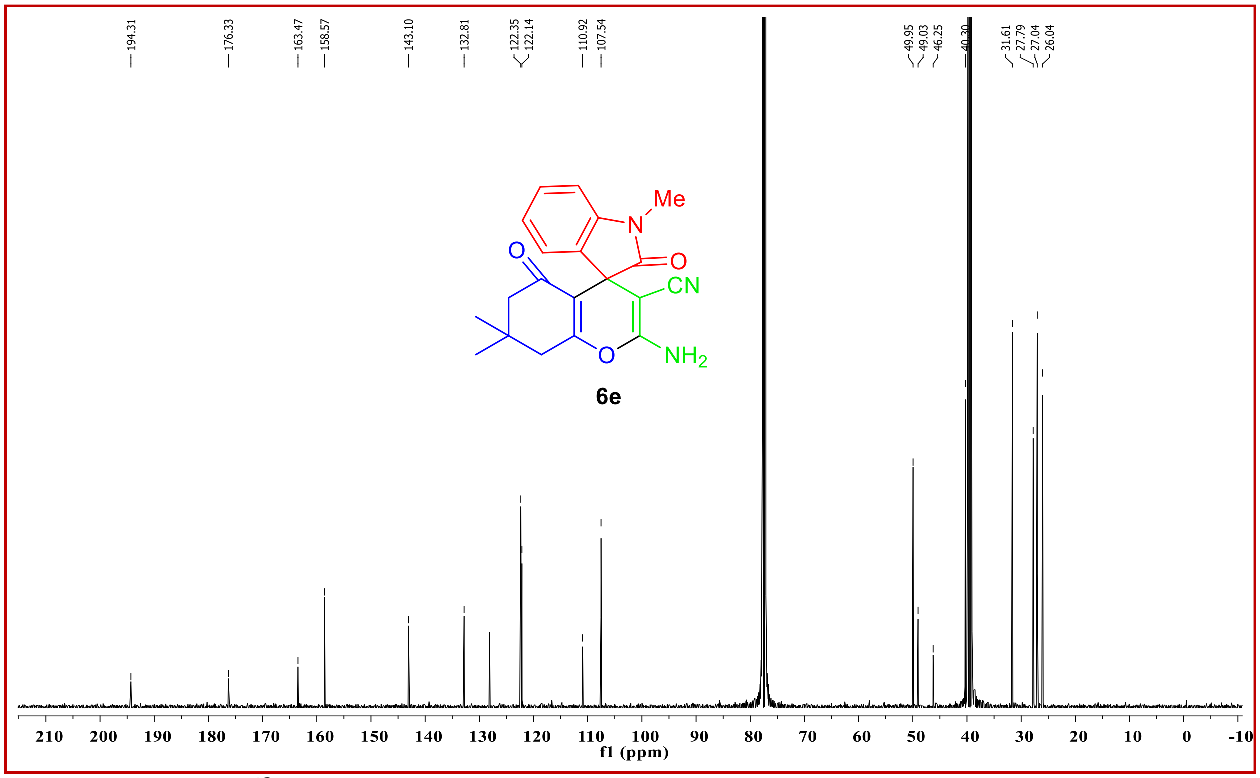

**Figure S53**: $^{13}$C NMR spectra of 2-amino-1',7,7-trimethyl-2',5-dioxo-5,6,7,8-tetrahydrospiro[chromene-4,3'-indoline]-3-carbonitrile **6e**

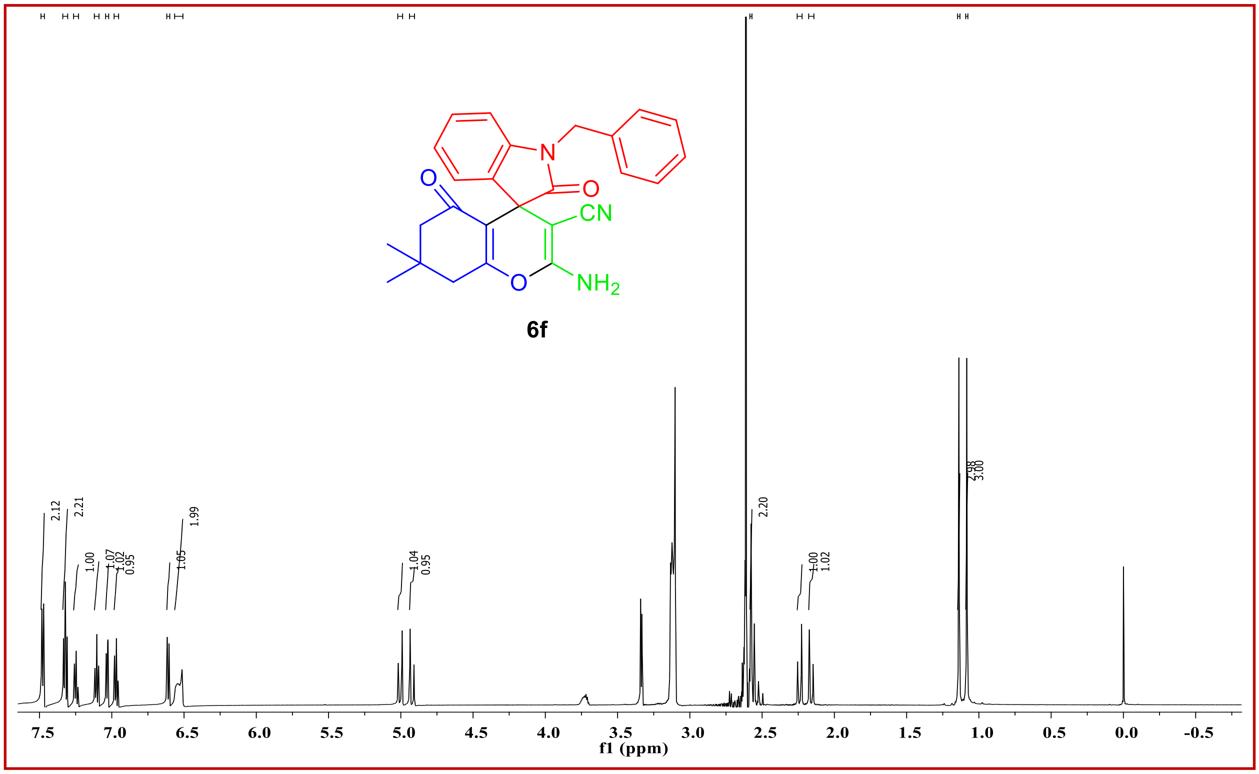

**Figure S54**: $^{1}$H NMR spectra of 2-amino-1'-benzyl-7,7-dimethyl-2',5-dioxo-5,6,7,8-tetrahydrospiro[chromene-4,3'-indoline]-3-carbonitrile **6f**

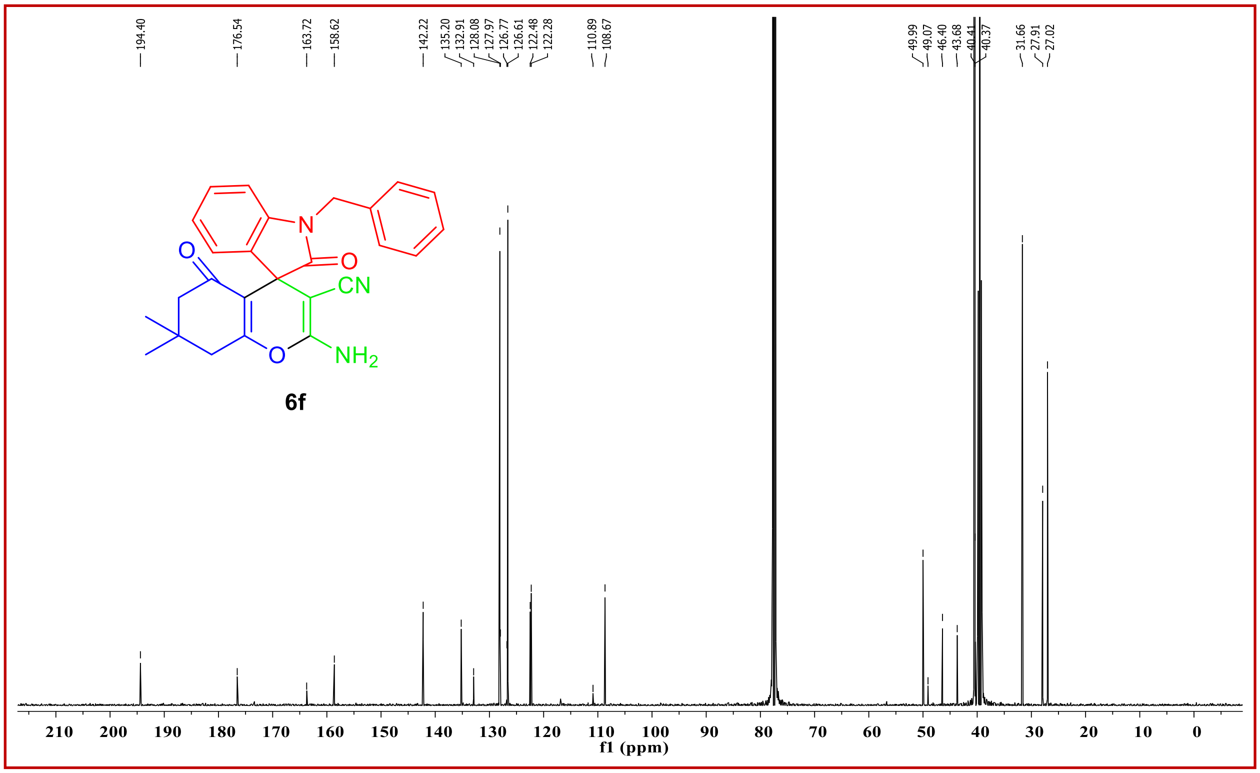

**Figure S55**: $^{13}C$ NMR spectra of 2-amino-1'-benzyl-7,7-dimethyl-2',5-dioxo-5,6,7,8-tetrahydrospiro[chromene-4,3'-indoline]-3-carbonitrile **6f**

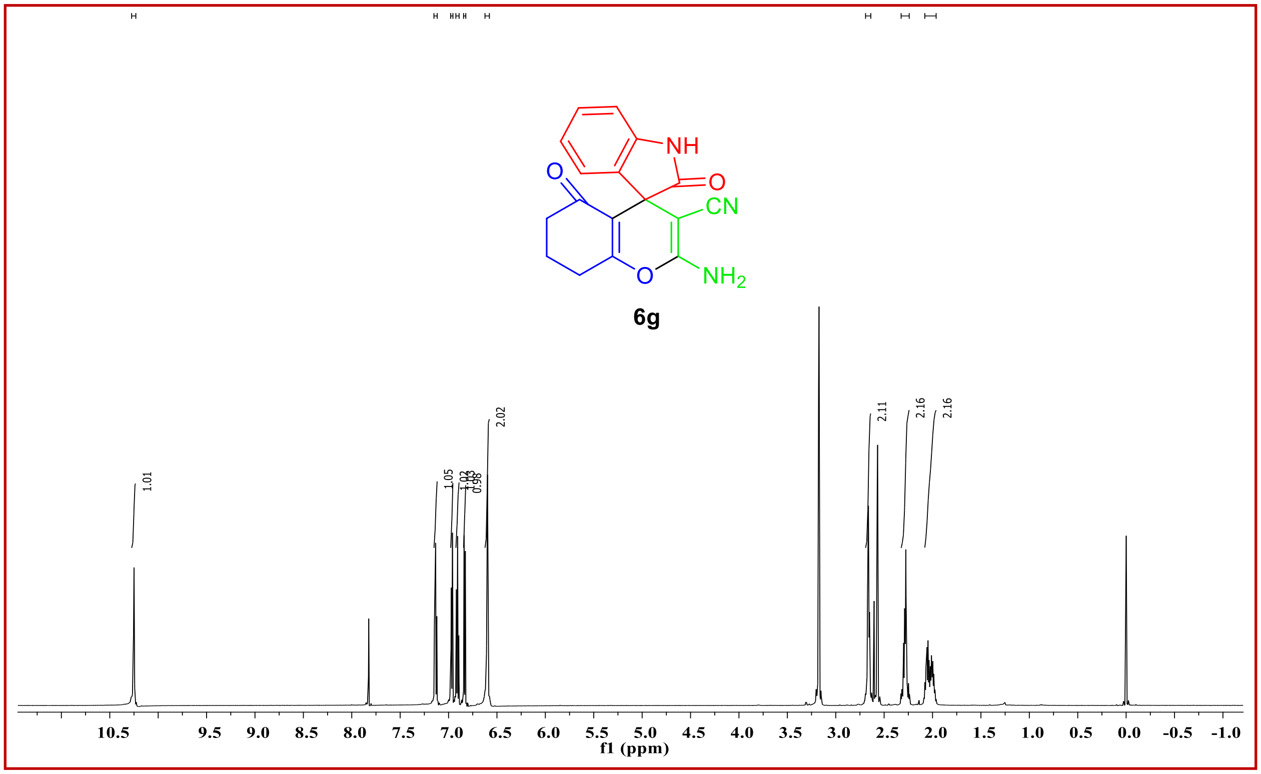

**Figure S56**: $^{1}H$ NMR spectra of 2-amino-2',5-dioxo-5,6,7,8-tetrahydrospiro[chromene-4,3'-indoline]-3-carbonitrile **6g**

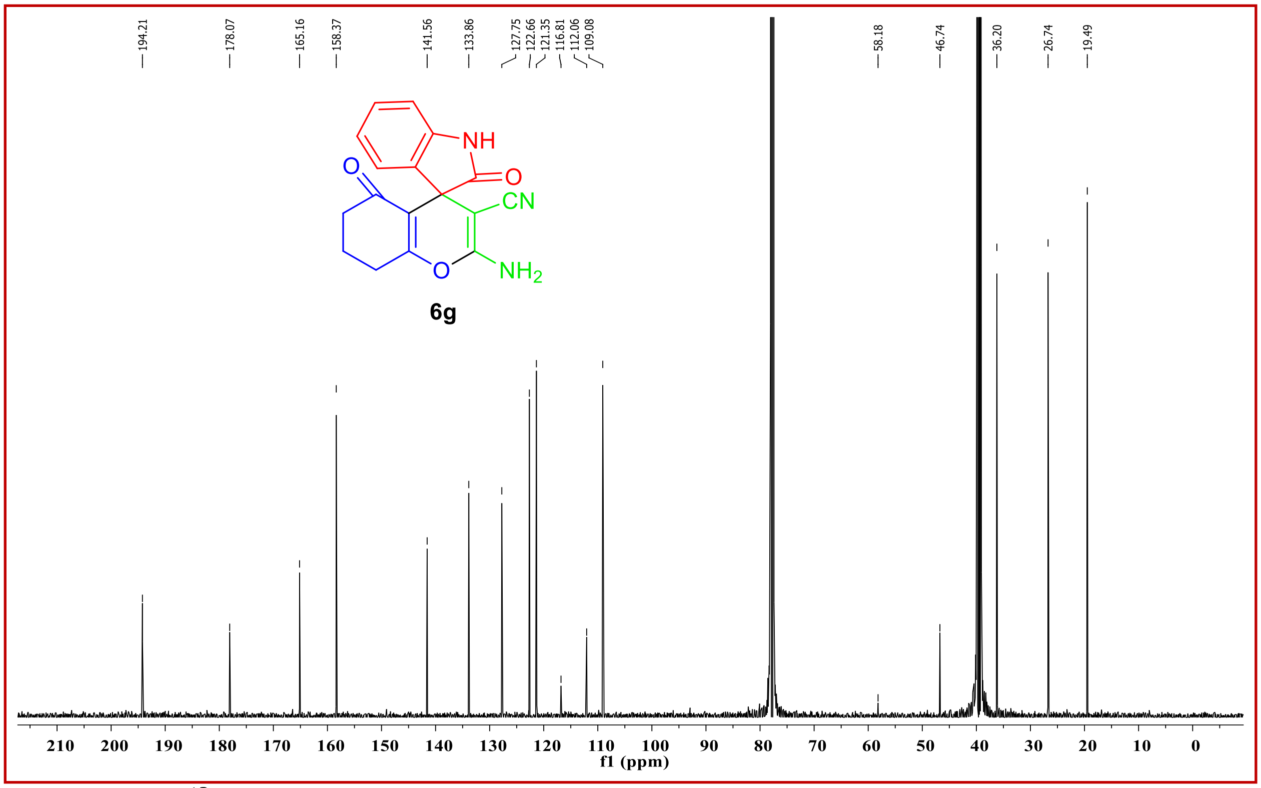


**Figure S57**: $^{13}$C NMR spectra of 2-amino-2',5-dioxo-5,6,7,8-tetrahydrospiro[chromene-4,3'-indoline]-3-carbonitrile **6g**

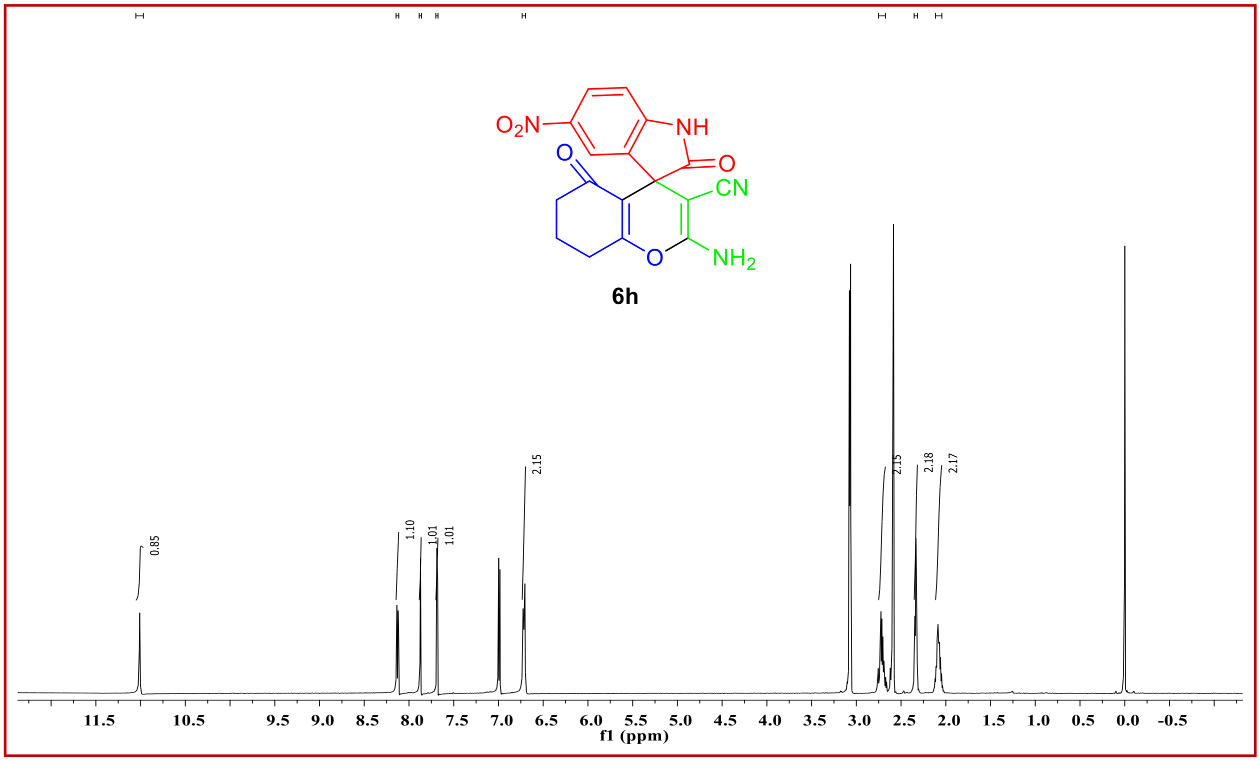


**Figure S58**: $^{1}$H NMR spectra of 2-amino-5'-nitro-2',5-dioxo-5,6,7,8-tetrahydrospiro[chromene-4,3'-indoline]-3-carbonitrile **6h**

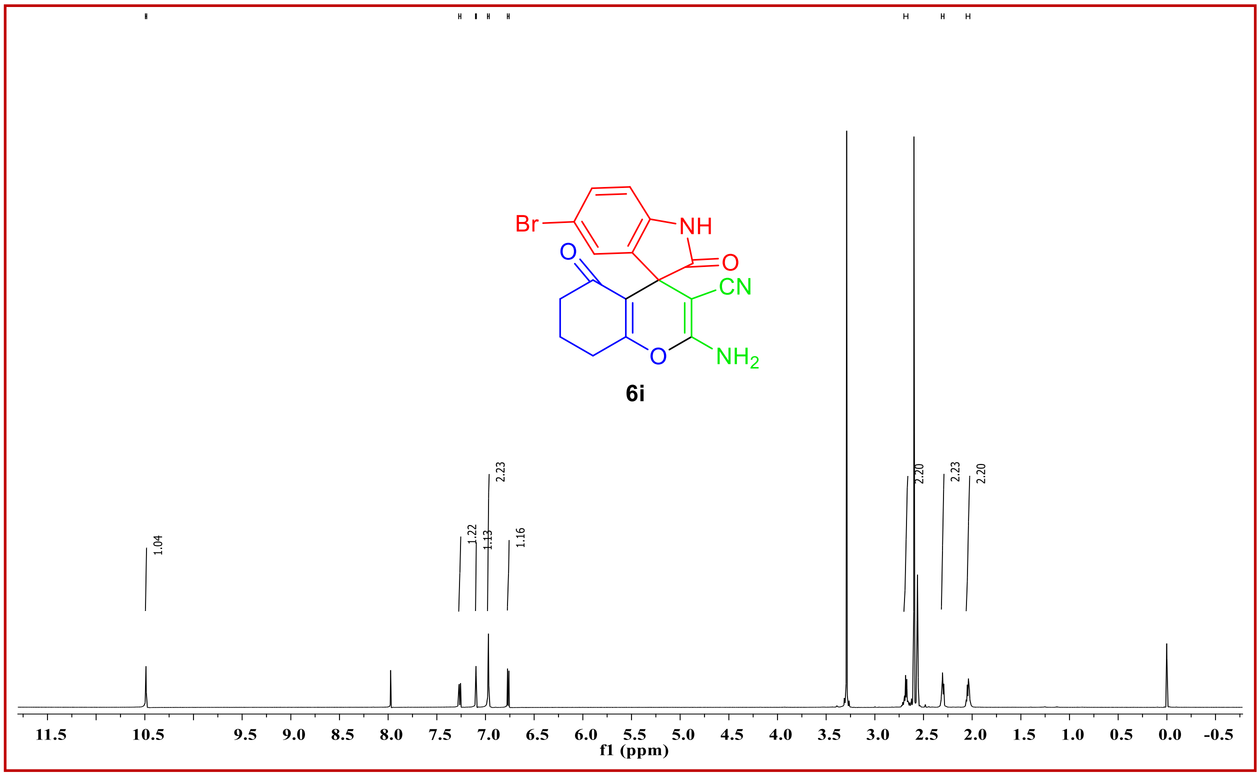

**Figure S59**: $^{1}$H NMR spectra of 2-amino-5'-bromo-2',5-dioxo-5,6,7,8-tetrahydrospiro[chromene-4,3'-indoline]-3-carbonitrile **6i**

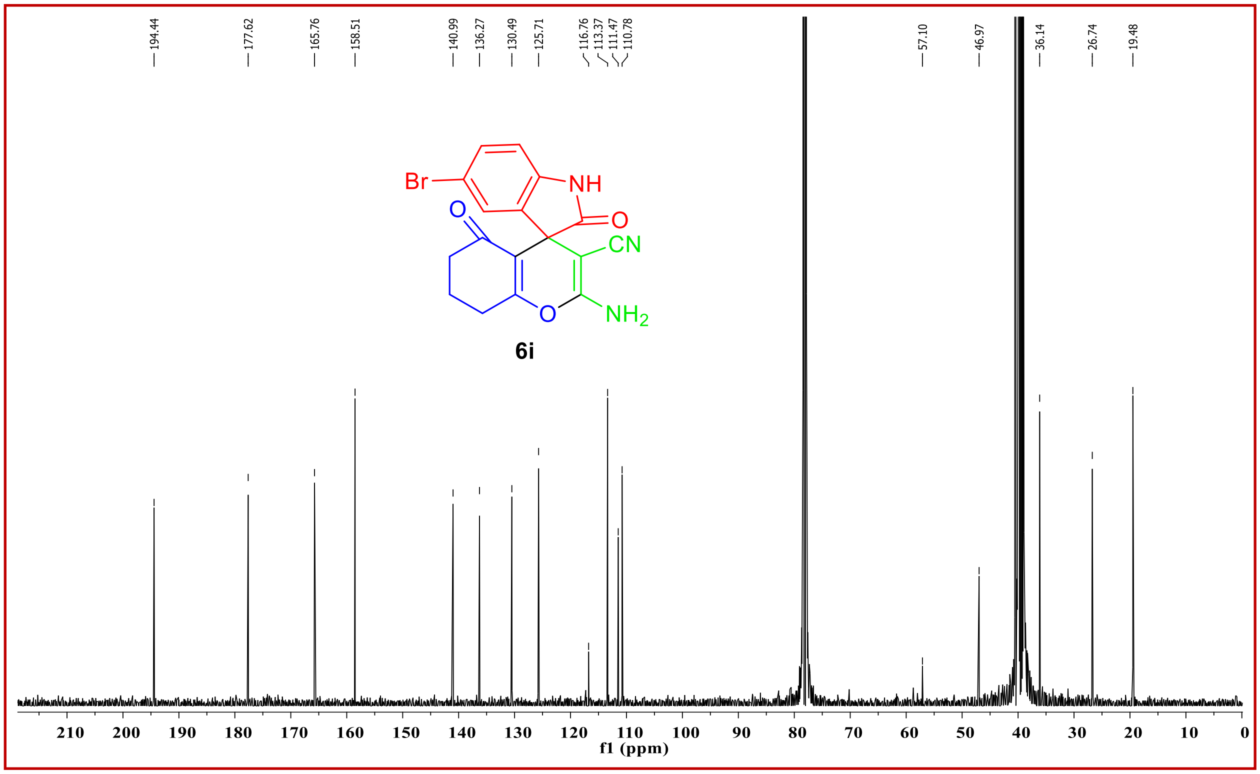

**Figure S60**: $^{13}$C NMR spectra of 2-amino-5'-bromo-2',5-dioxo-5,6,7,8-tetrahydrospiro[chromene-4,3'-indoline]-3-carbonitrile **6i**

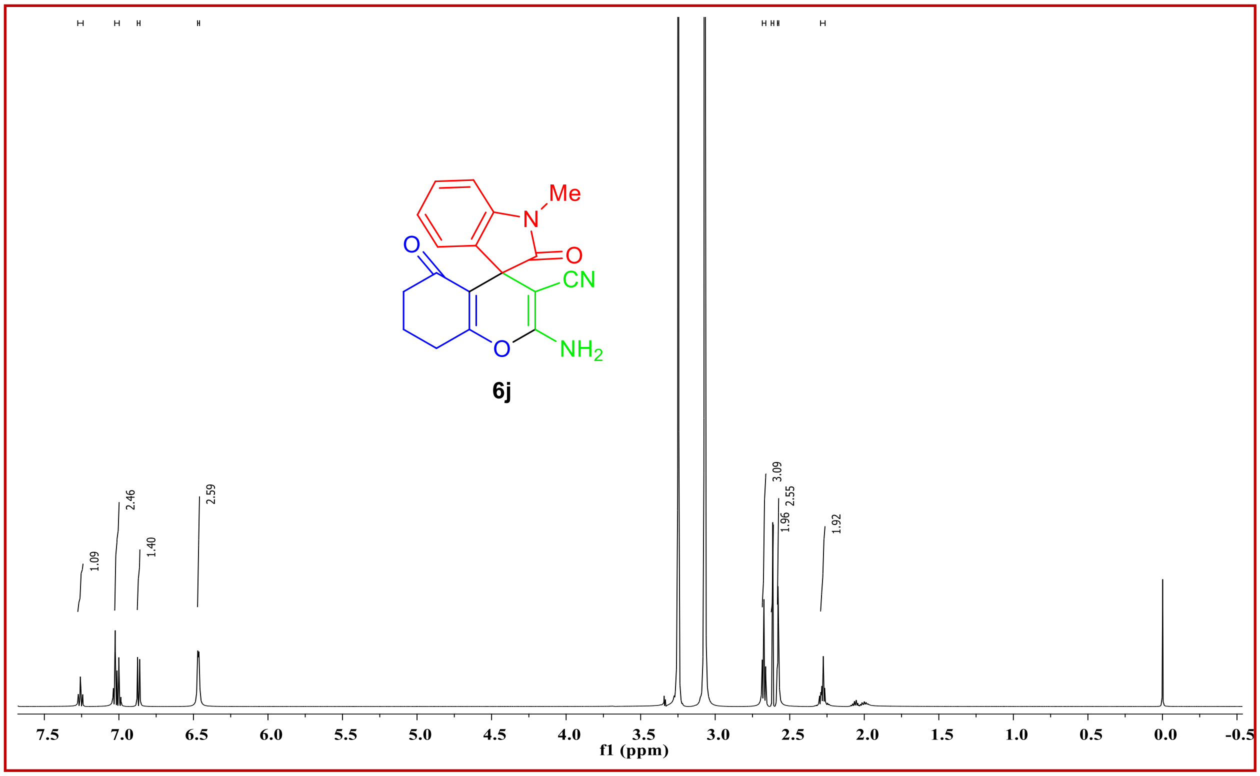


**Figure S61**: $^{1}$H NMR spectra of 2-amino-1'-methyl-2',5-dioxo-5,6,7,8-tetrahydrospiro[chromene-4,3'-indoline]-3-carbonitrile **6j**

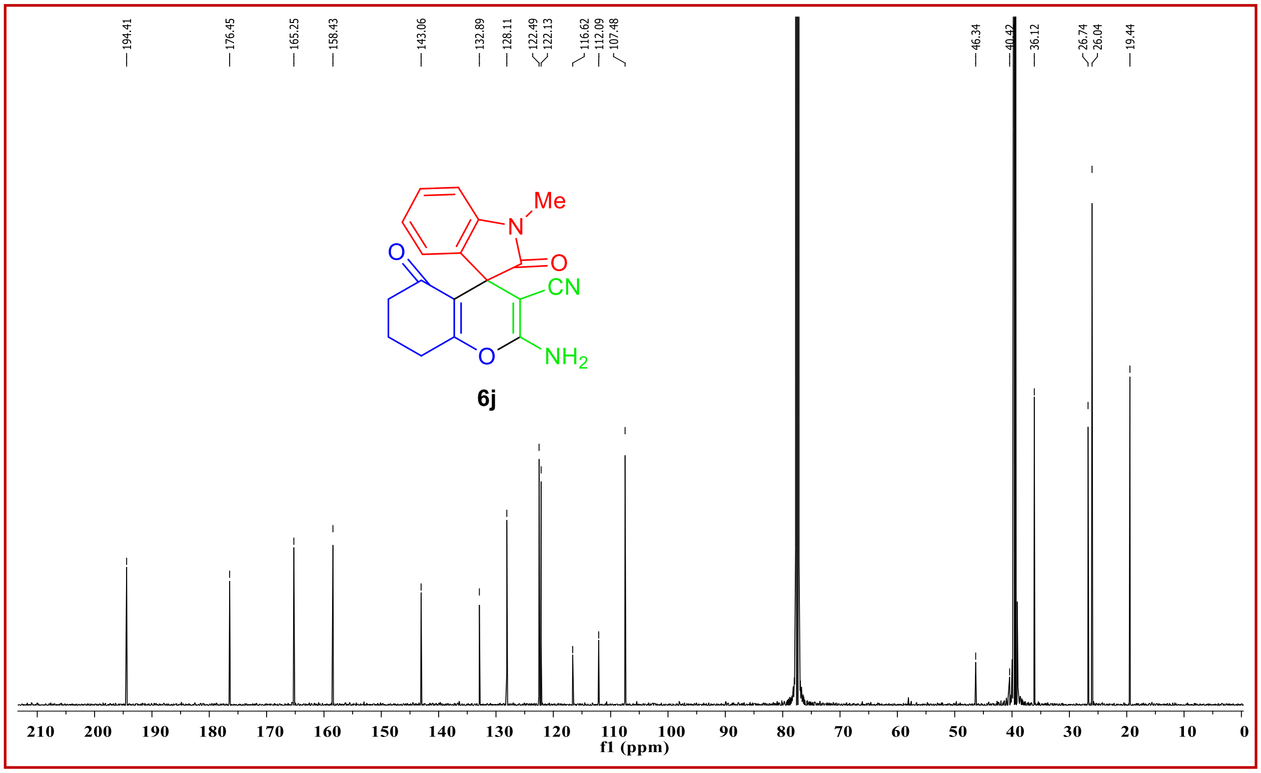


**Figure S62**: $^{13}$C NMR spectra of 2-amino-1'-methyl-2',5-dioxo-5,6,7,8-tetrahydrospiro[chromene-4,3'-indoline]-3-carbonitrile **6j**

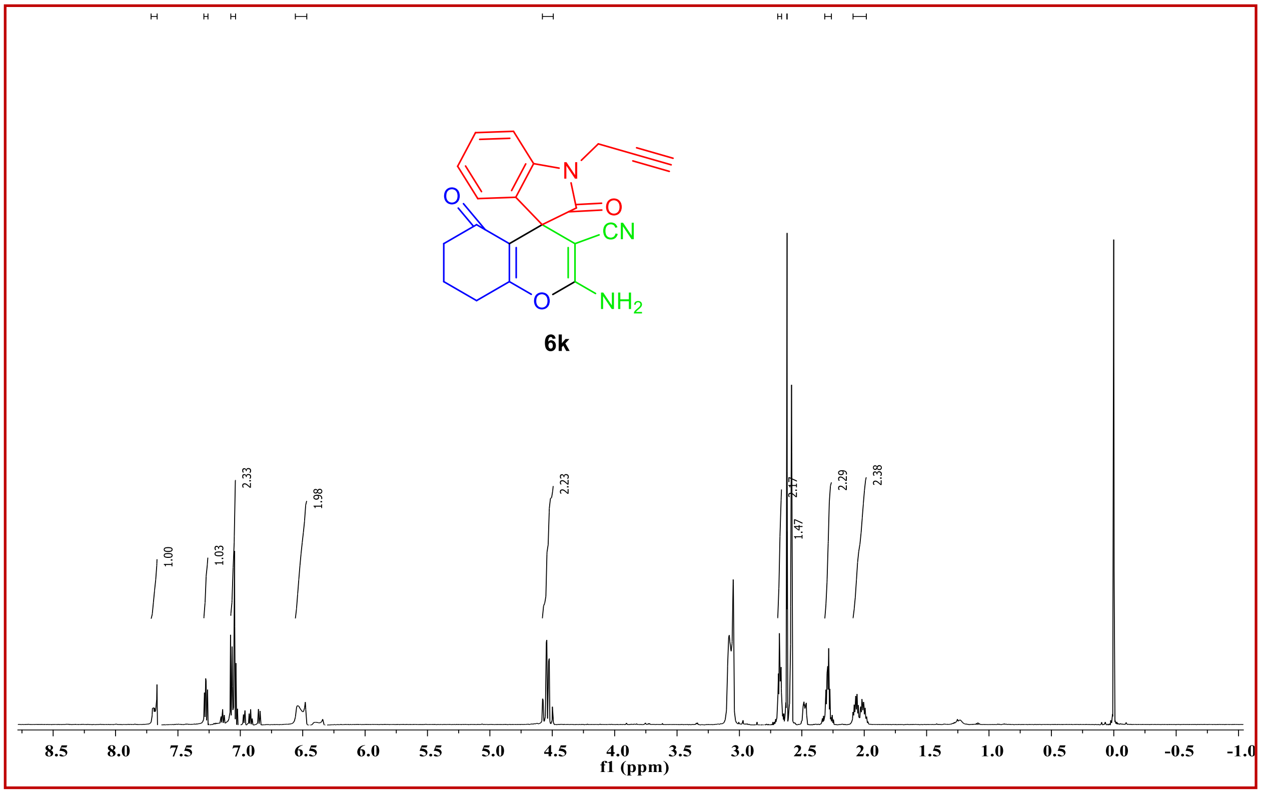


**Figure S63**: $^{1}$H NMR spectra of 2-amino-2',5-dioxo-1'-(prop-2-yn-1-yl)-5,6,7,8-tetrahydrospiro[chromene-4,3'-indoline]-3-carbonitrile **6k**

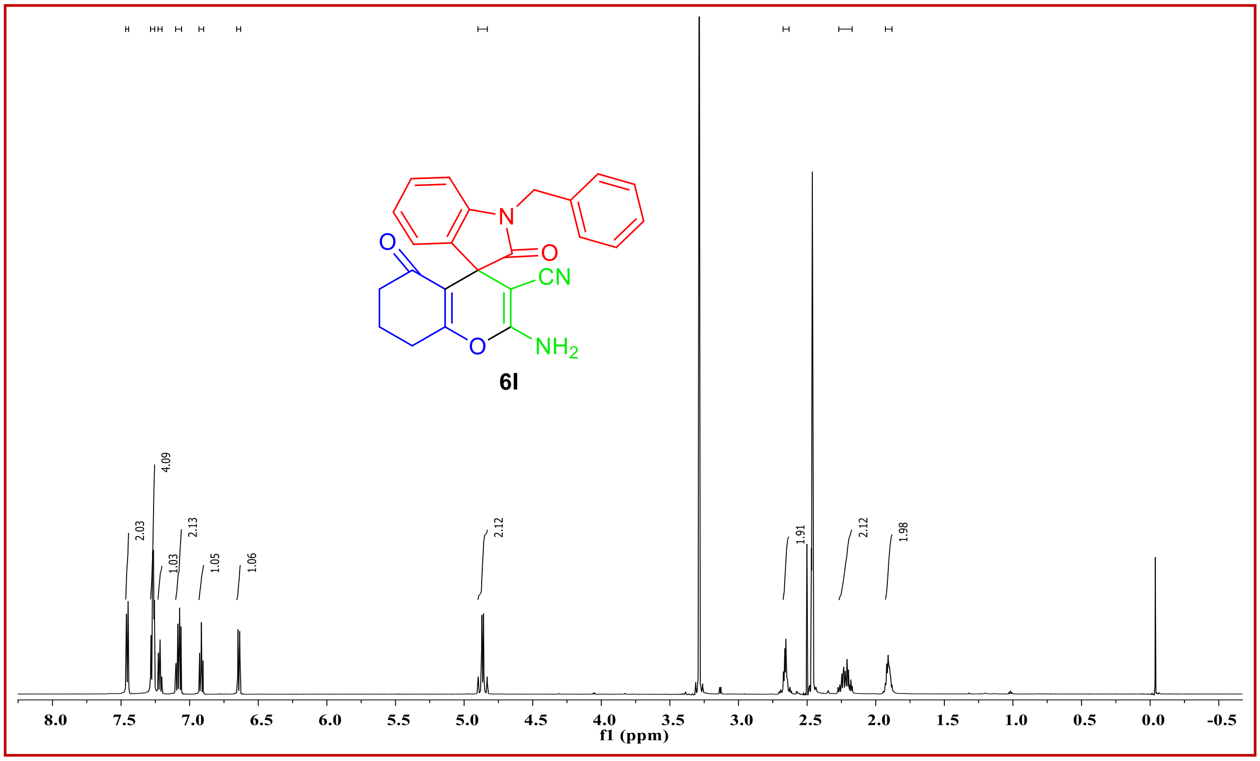


**Figure S64**: $^{1}$H NMR spectra of 2-amino-1'-benzyl-2',5-dioxo-5,6,7,8-tetrahydrospiro[chromene-4,3'-indoline]-3-carbonitrile **6l**

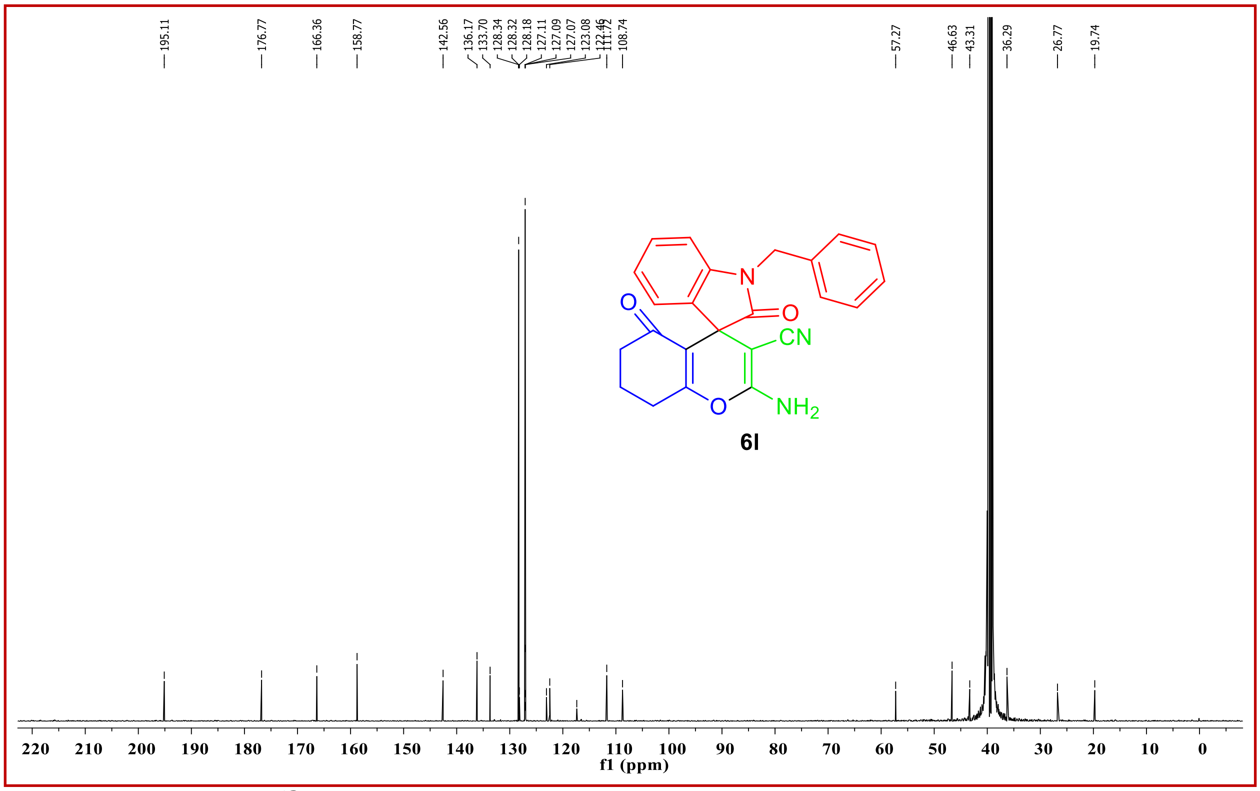


**Figure S65**: $^{13}$C NMR spectra of 2-amino-1'-benzyl-2',5-dioxo-5,6,7,8-tetrahydrospiro[chromene-4,3'-indoline]-3-carbonitrile **6l**

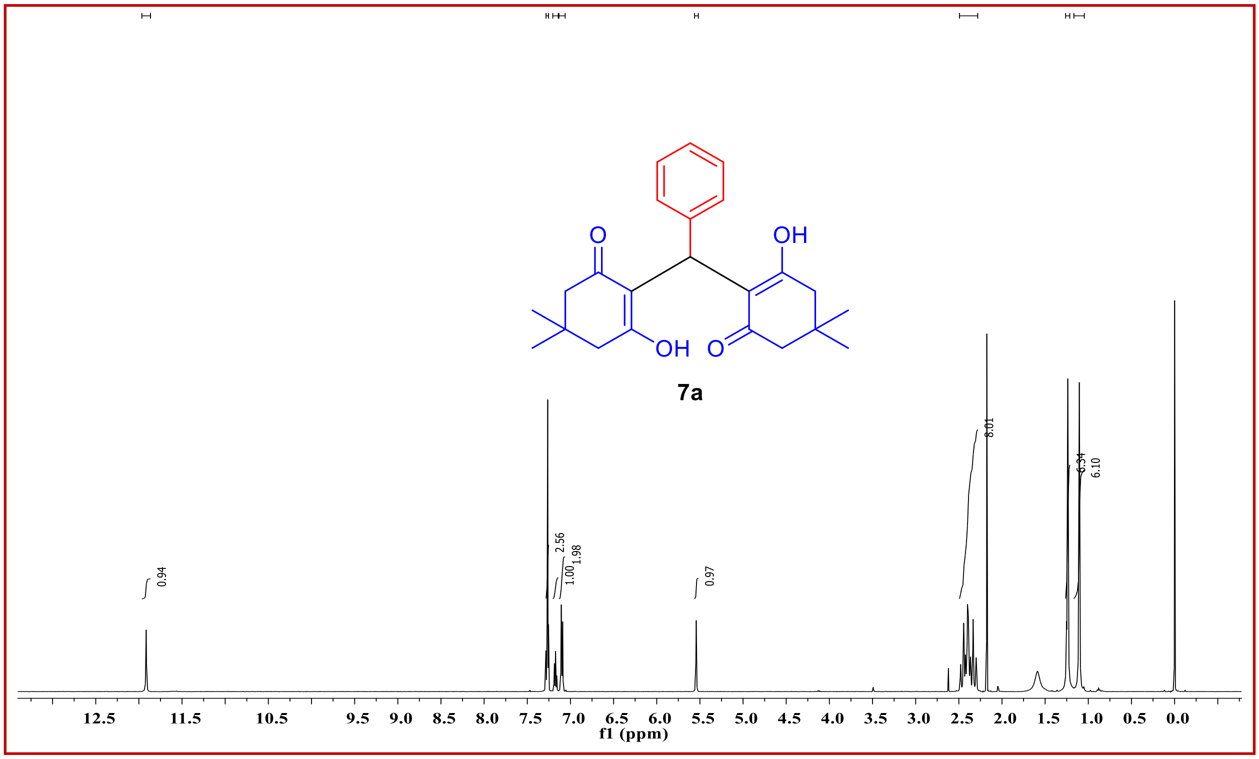


**Figure S66**: $^{1}$H NMR spectra of 2,2'-(phenylmethylene)bis(3-hydroxy-5,5-dimethylcyclohex-2-enone) **7a**

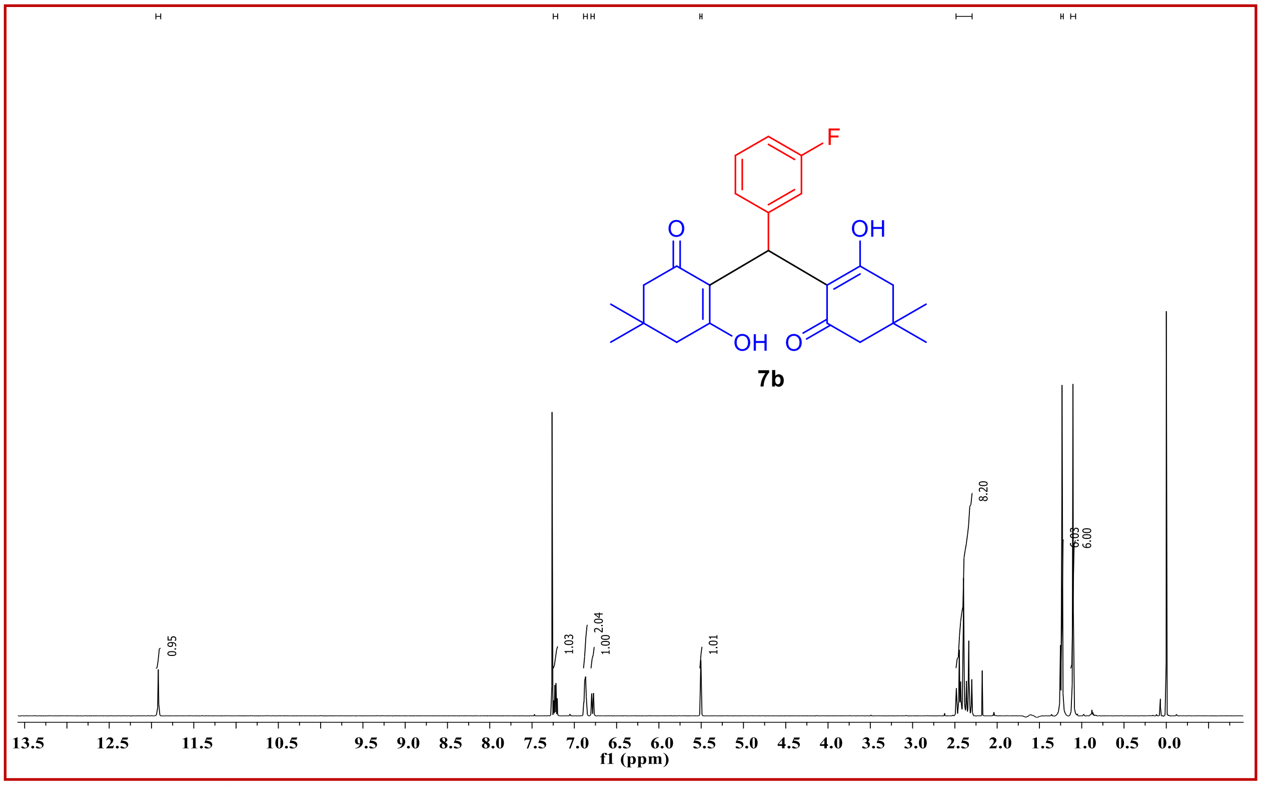

**Figure S67**: $^{1}$H NMR spectra of 2,2'-((3-fluorophenyl)methylene)bis(3-hydroxy-5,5-dimethylcyclohex-2-enone) **7b**

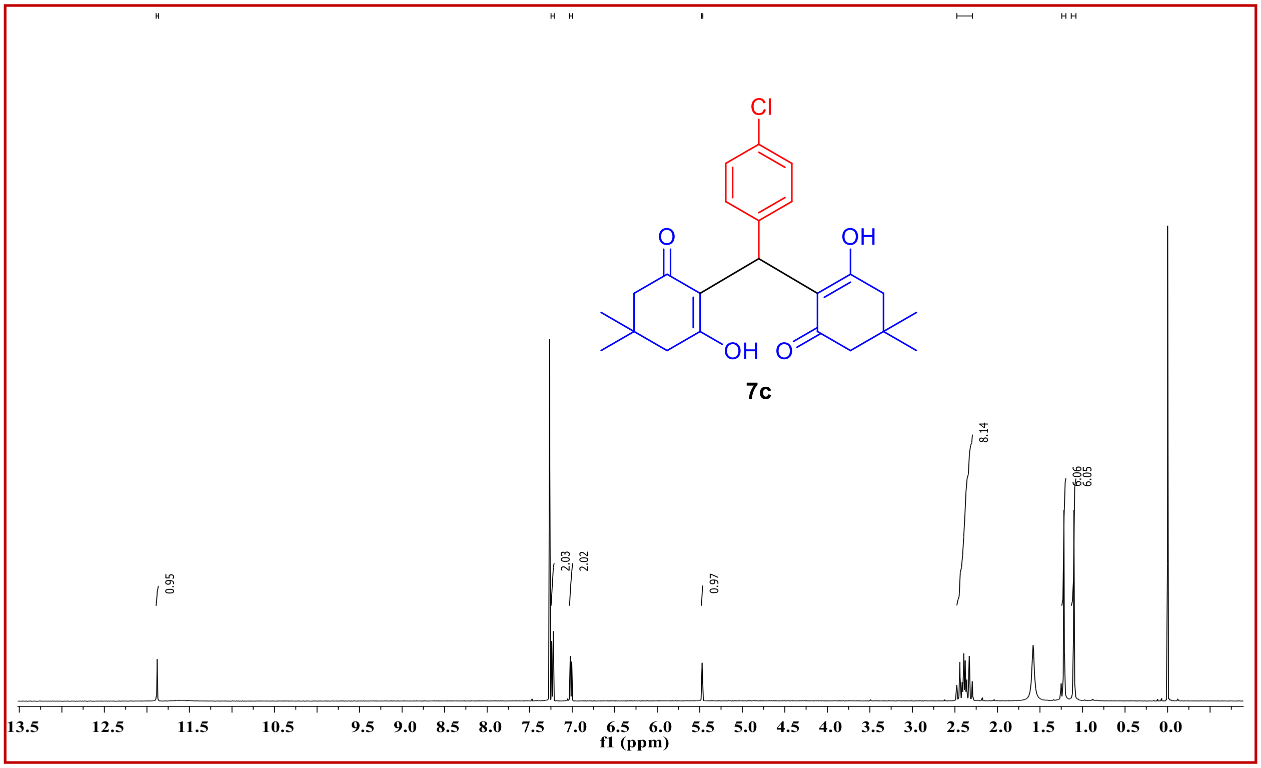

**Figure S68**: $^{1}$H NMR spectra of 2,2'-((4-chlorophenyl)methylene)bis(3-hydroxy-5,5-dimethylcyclohex-2-enone) **7c**

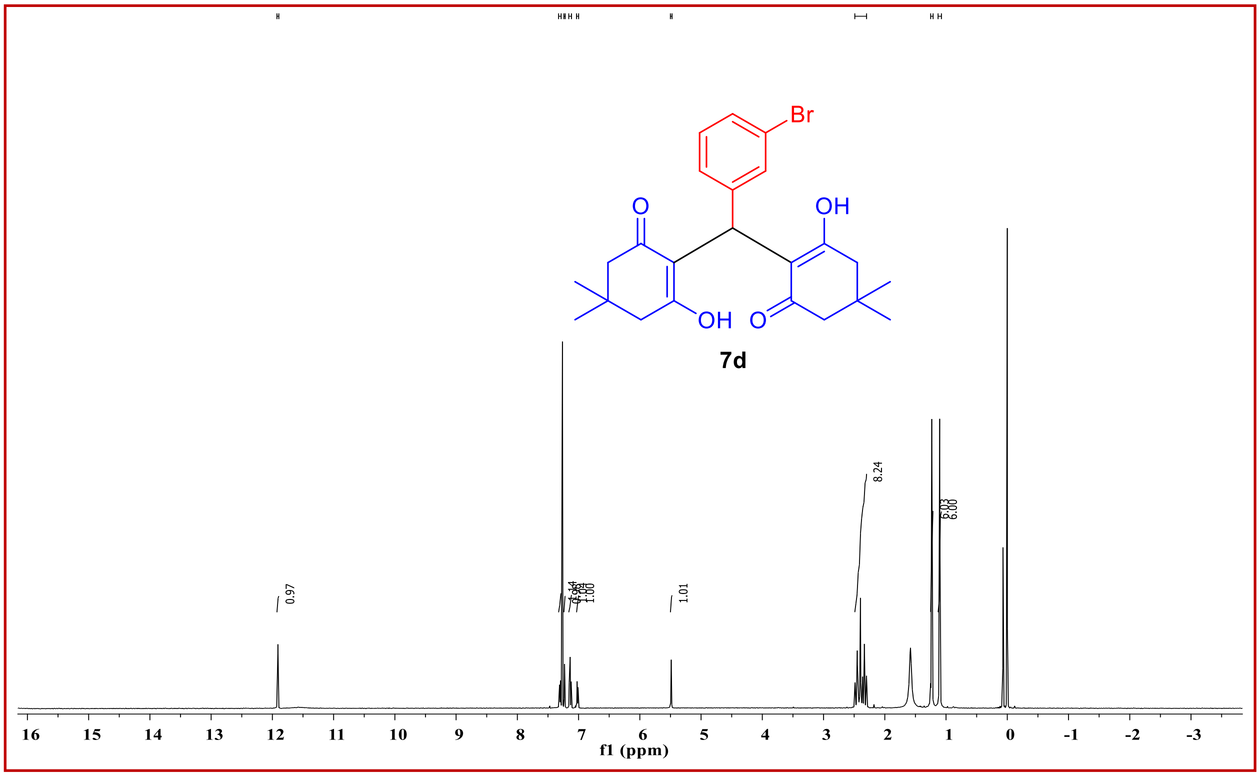


**Figure S69**: $^{1}$H NMR spectra of 2,2'-((3-bromophenyl)methylene)bis(3-hydroxy-5,5-dimethylcyclohex-2-enone) **7d**

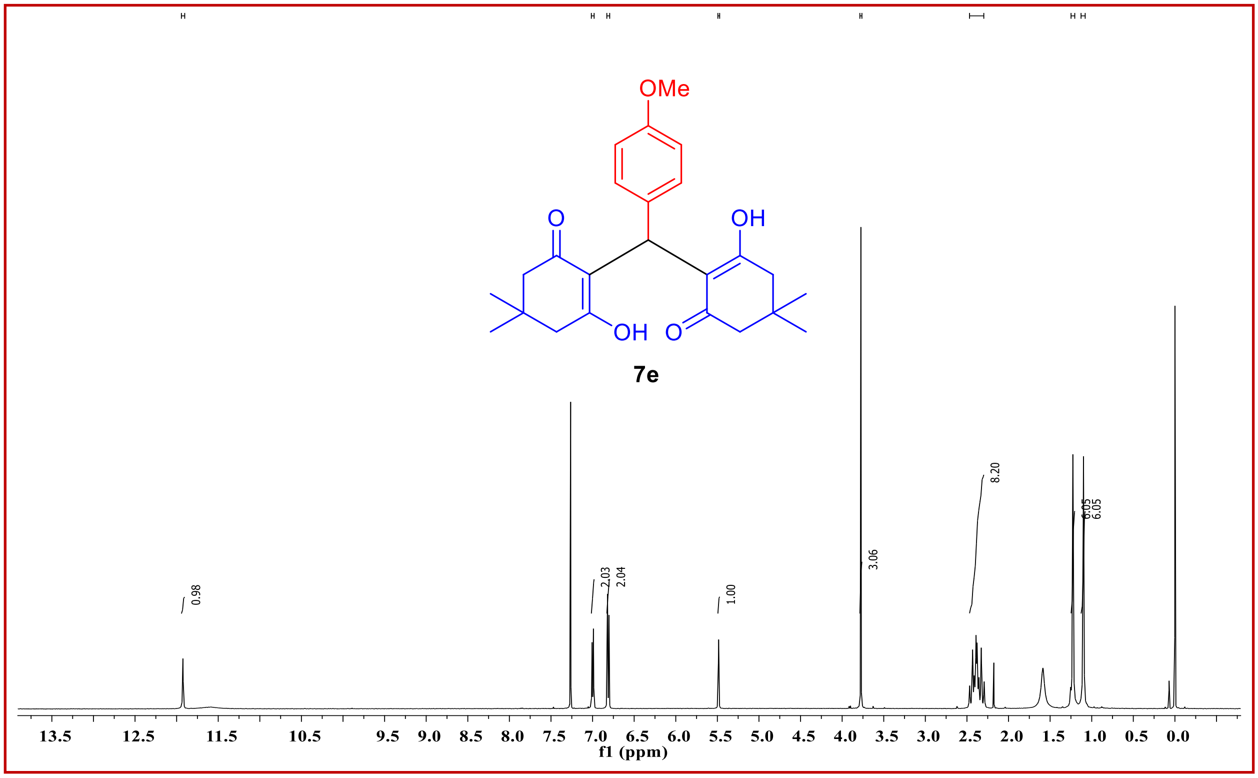


**Figure S70**: $^{1}$H NMR spectra of 2,2'-((4-methoxyphenyl)methylene)bis(3-hydroxy-5,5-dimethylcyclohex-2-enone) **7e**

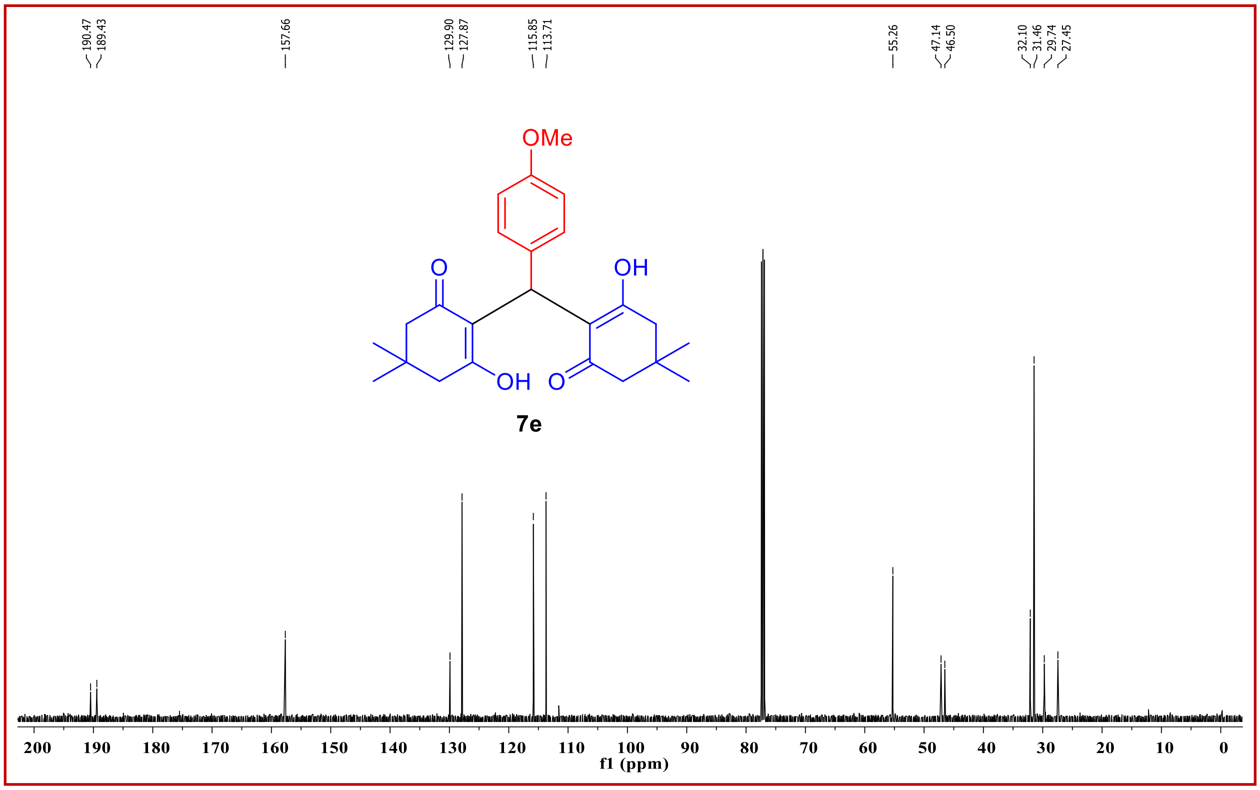

**Figure S71**: $^{13}C$ NMR spectra of 2,2'-((4-methoxyphenyl)methylene)bis(3-hydroxy-5,5-dimethylcyclohex-2-enone) **7e**

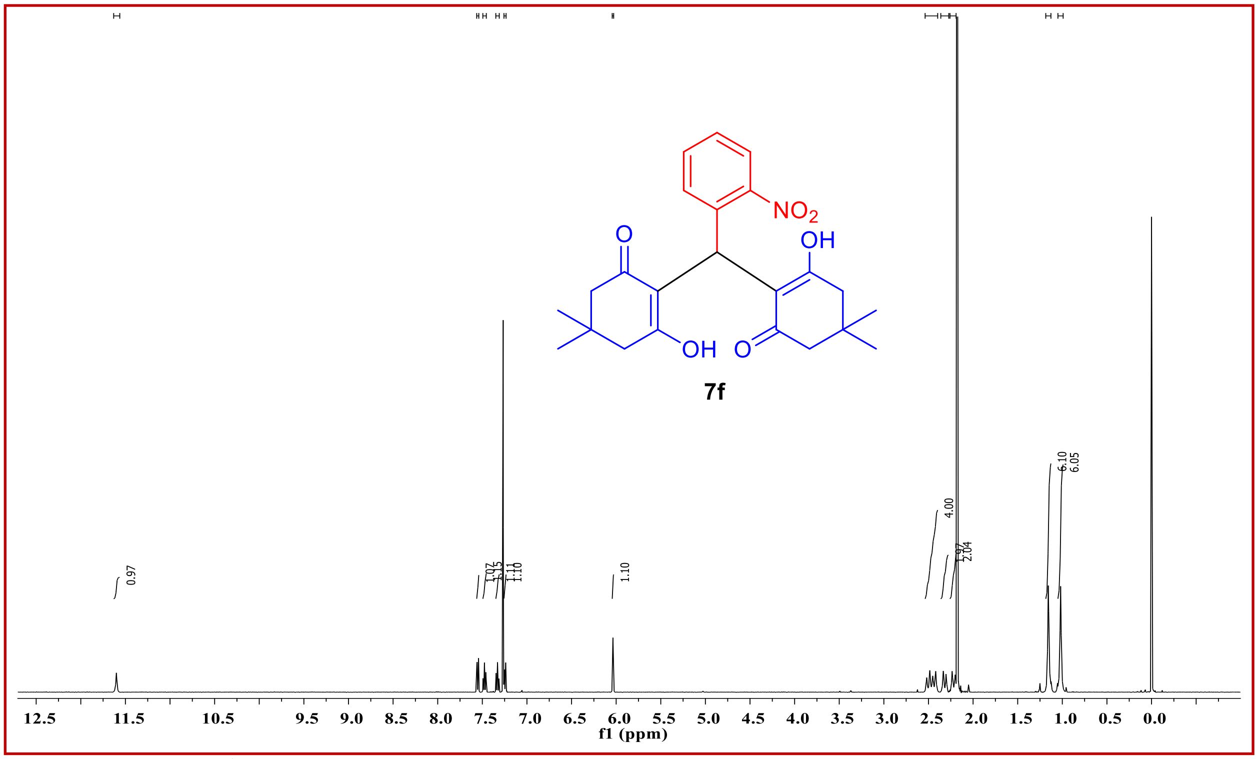

**Figure S72**: $^{1}H$ NMR spectra of 2,2'-((2-nitrophenyl)methylene)bis(3-hydroxy-5,5-dimethylcyclohex-2-enone) **7f**

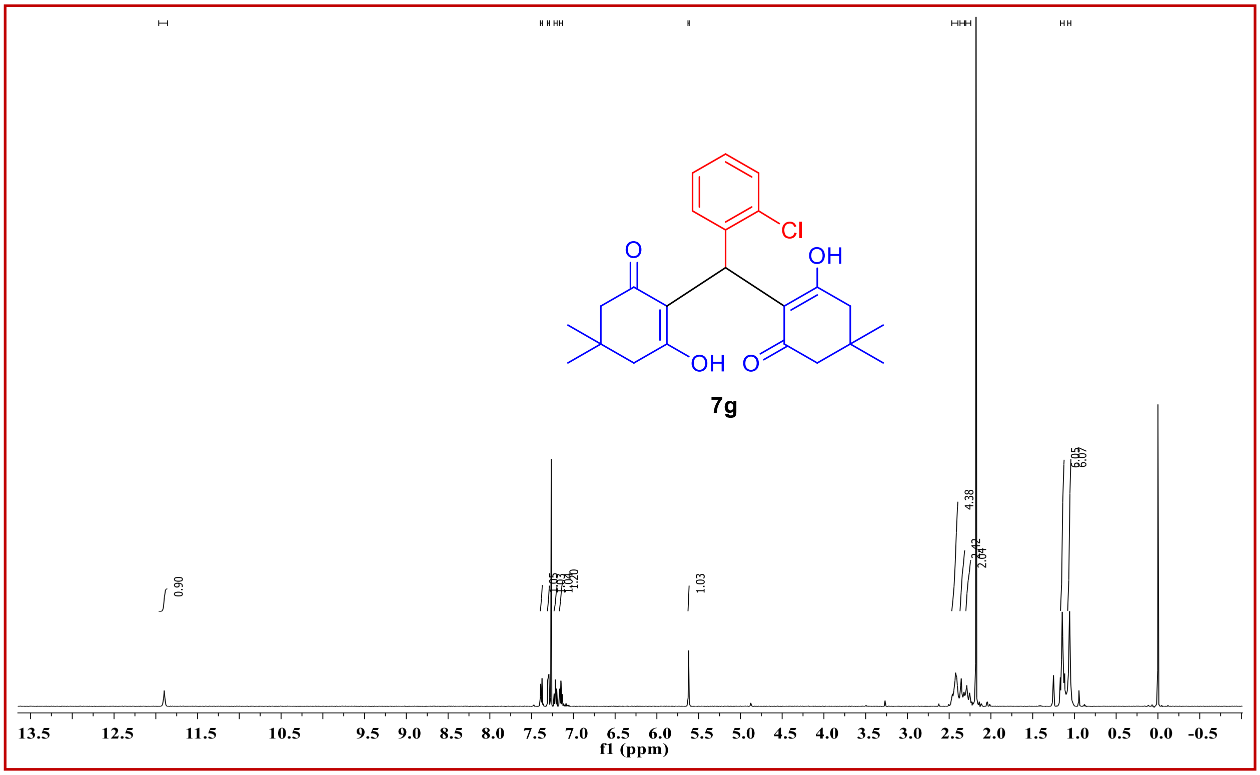


**Figure S73**: $^{1}$H NMR spectra of 2,2'-((2-chlorophenyl)methylene)bis(3-hydroxy-5,5-dimethylcyclohex-2-enone) **7g**

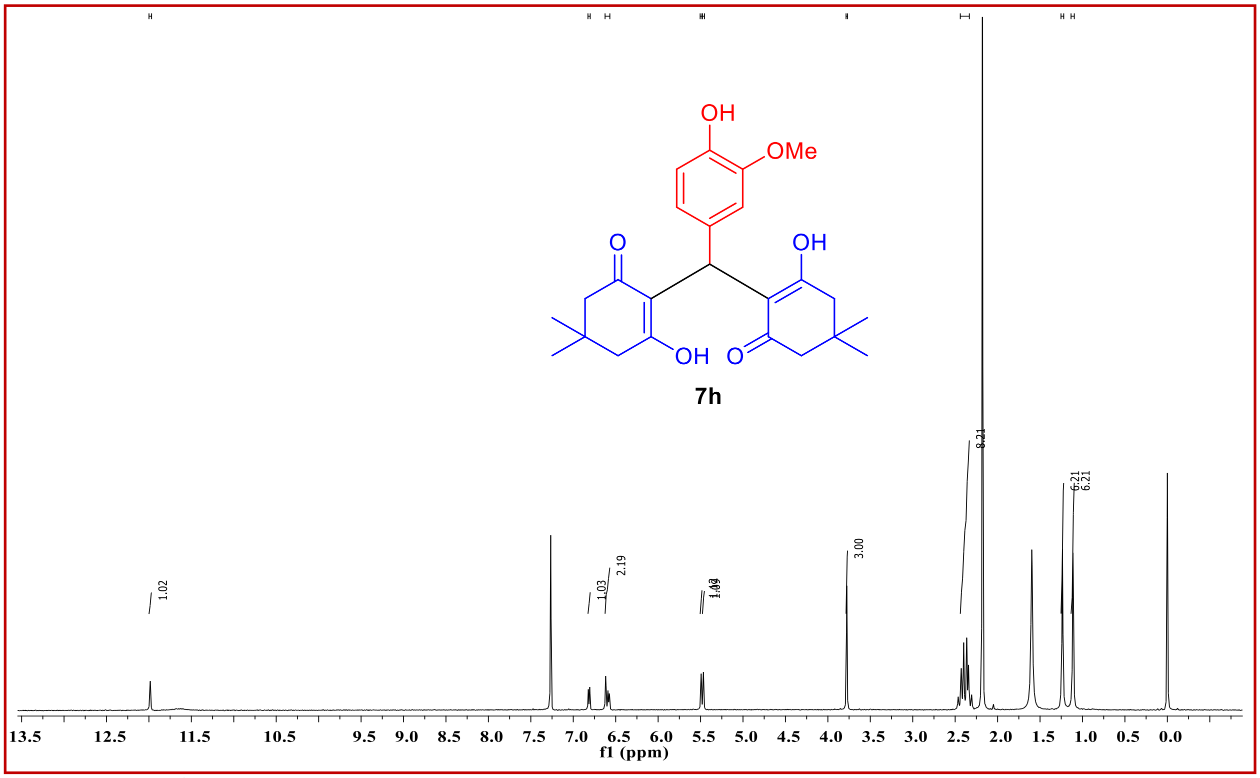


**Figure S74**: $^{1}$H NMR spectra of 2,2'-((4-hydroxy-3-methoxyphenyl)methylene)bis(3-hydroxy-5,5-dimethylcyclohex-2-enone) **7h**

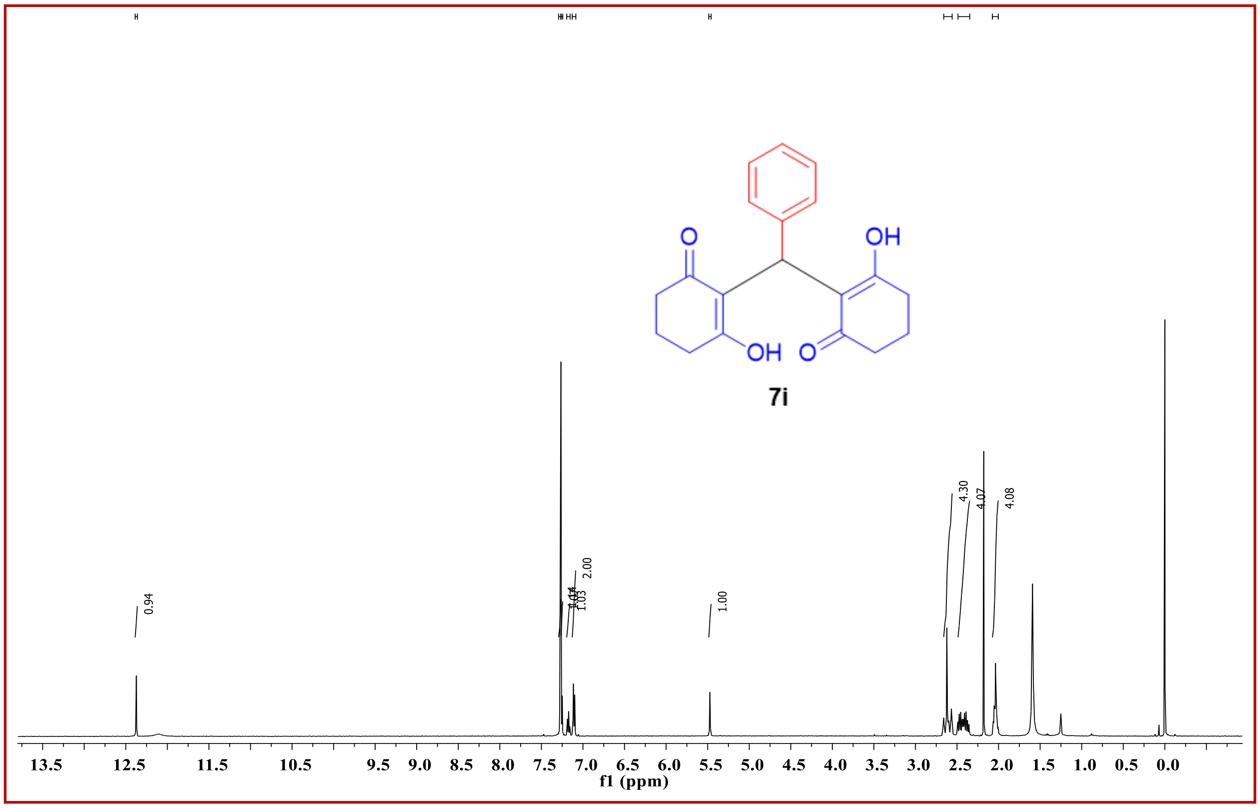


**Figure S75**: $^{1}$H NMR spectra of 2,2'-( phenylmethylene)bis(3-hydroxycyclohex-2-enone) **7i**

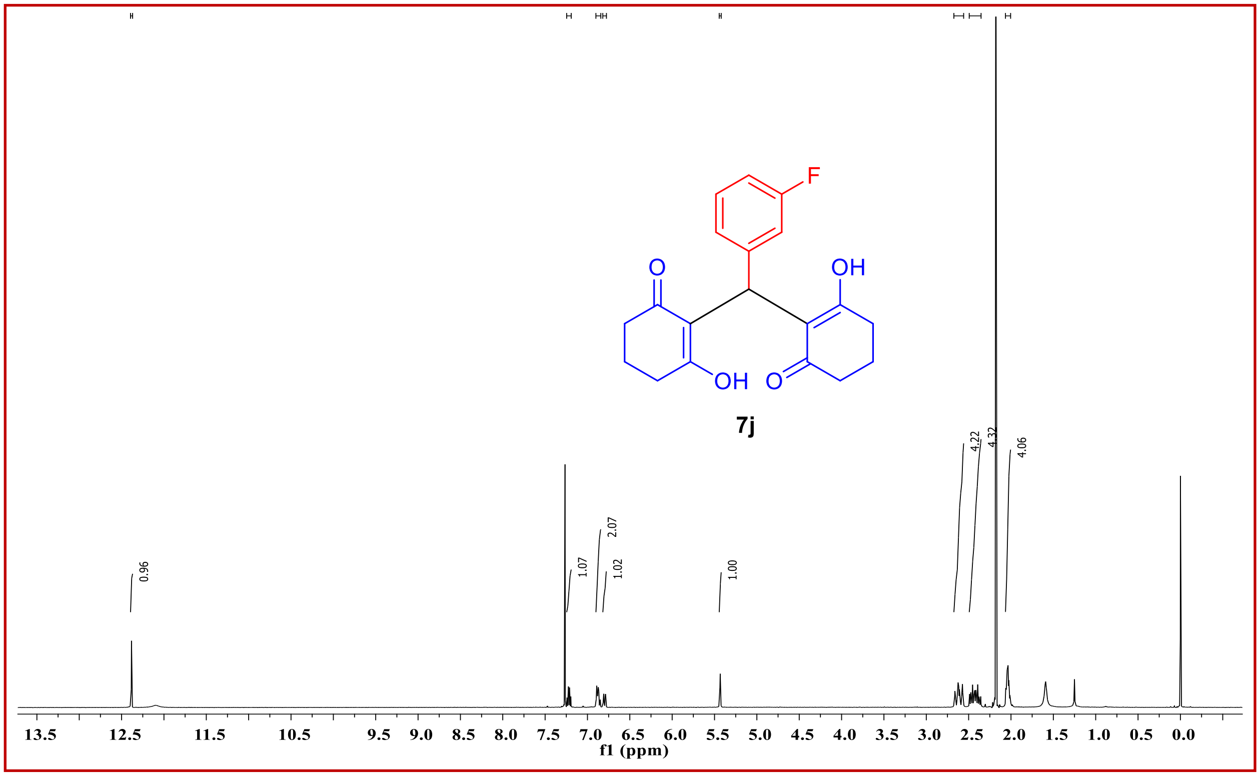


**Figure S76**: $^{1}$H NMR spectra of 2,2'-((3-fluorophenyl)methylene)bis(3-hydroxycyclohex-2-enone) **7j**

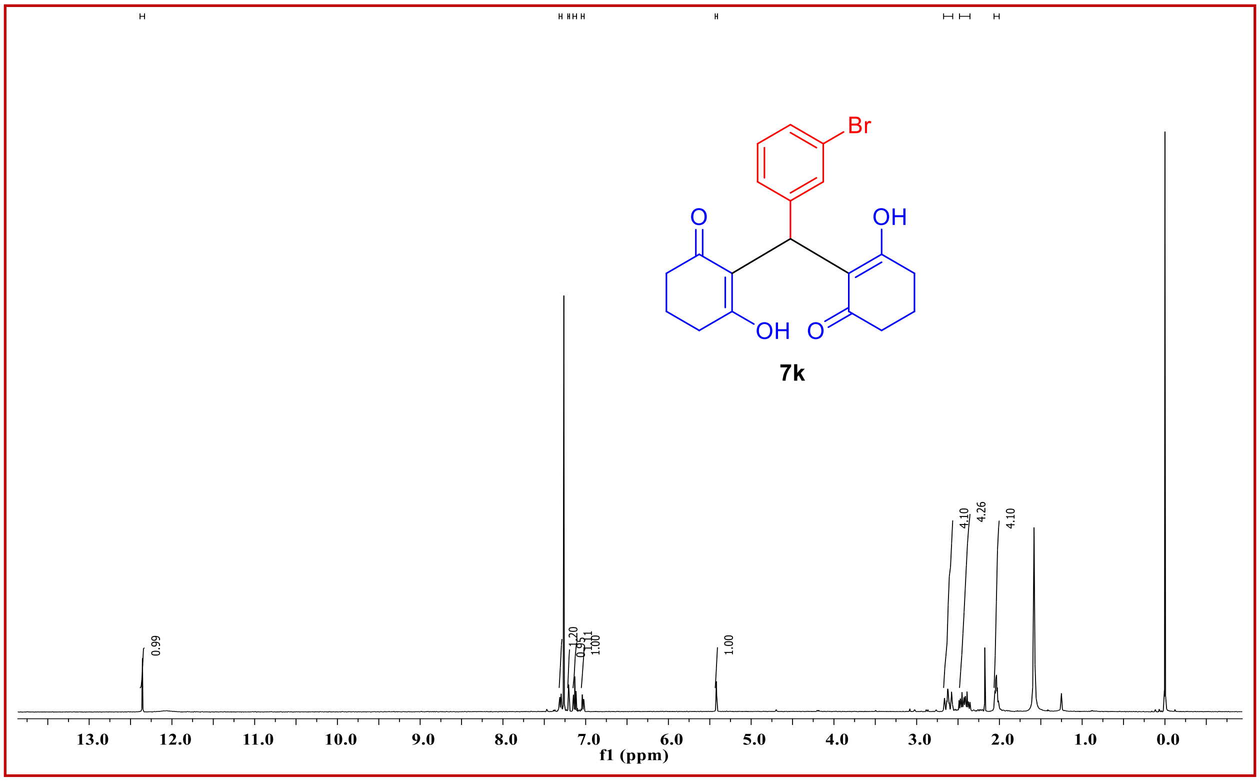


**Figure S77**: $^{1}$H NMR spectra of 2,2'-((3-bromophenyl)methylene)bis(3-hydroxycyclohex-2-enone) **7k**

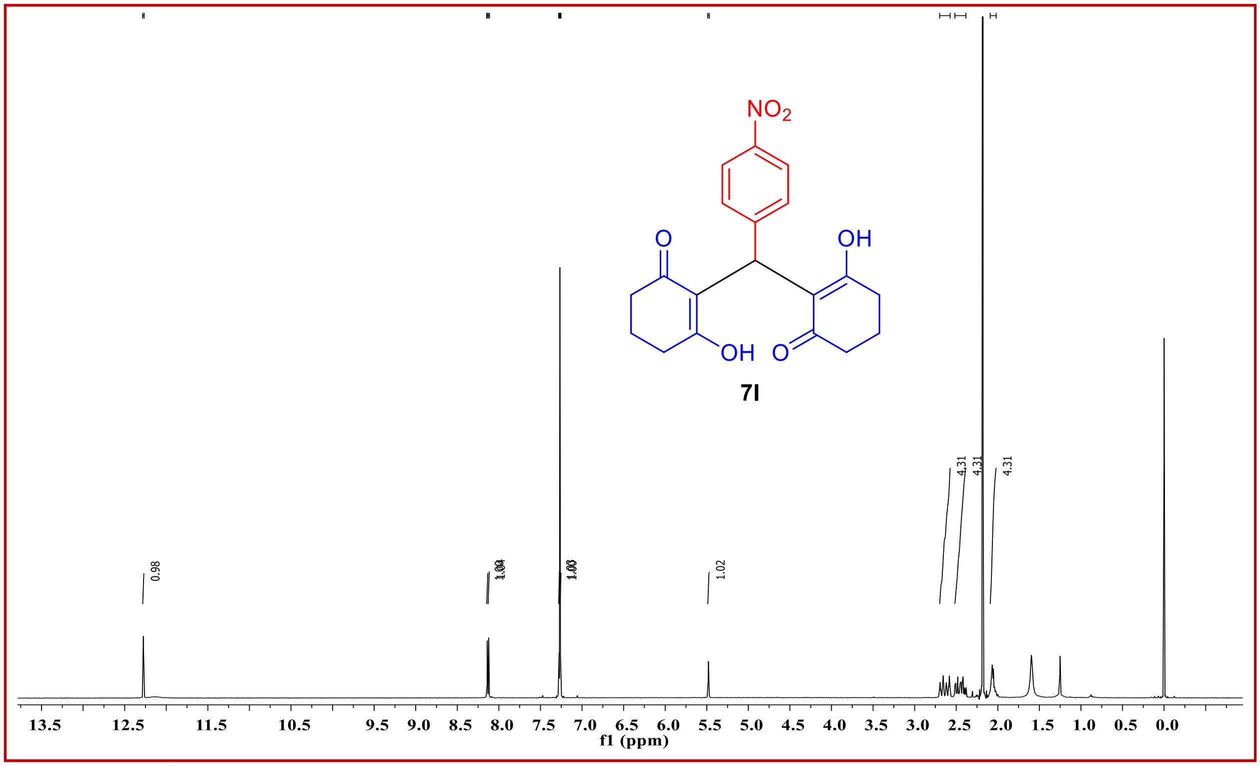


**Figure S78**: $^{1}$H NMR spectra of 2,2'-((4-nitrophenyl)methylene)bis(3-hydroxycyclohex-2-enone) **7l**

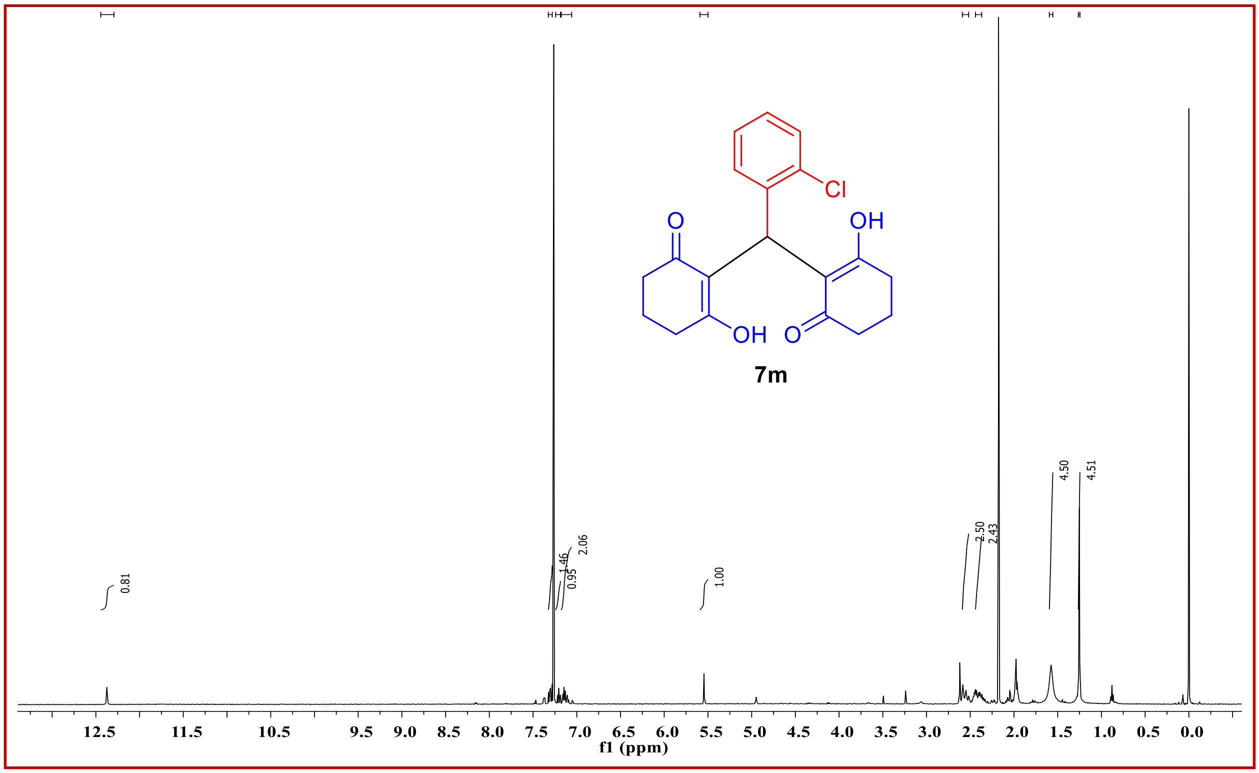


**Figure S79**: $^{1}$H NMR spectra of 2,2'-((2-chlorophenyl)methylene)bis(3-hydroxycyclohex-2-enone) **7m**

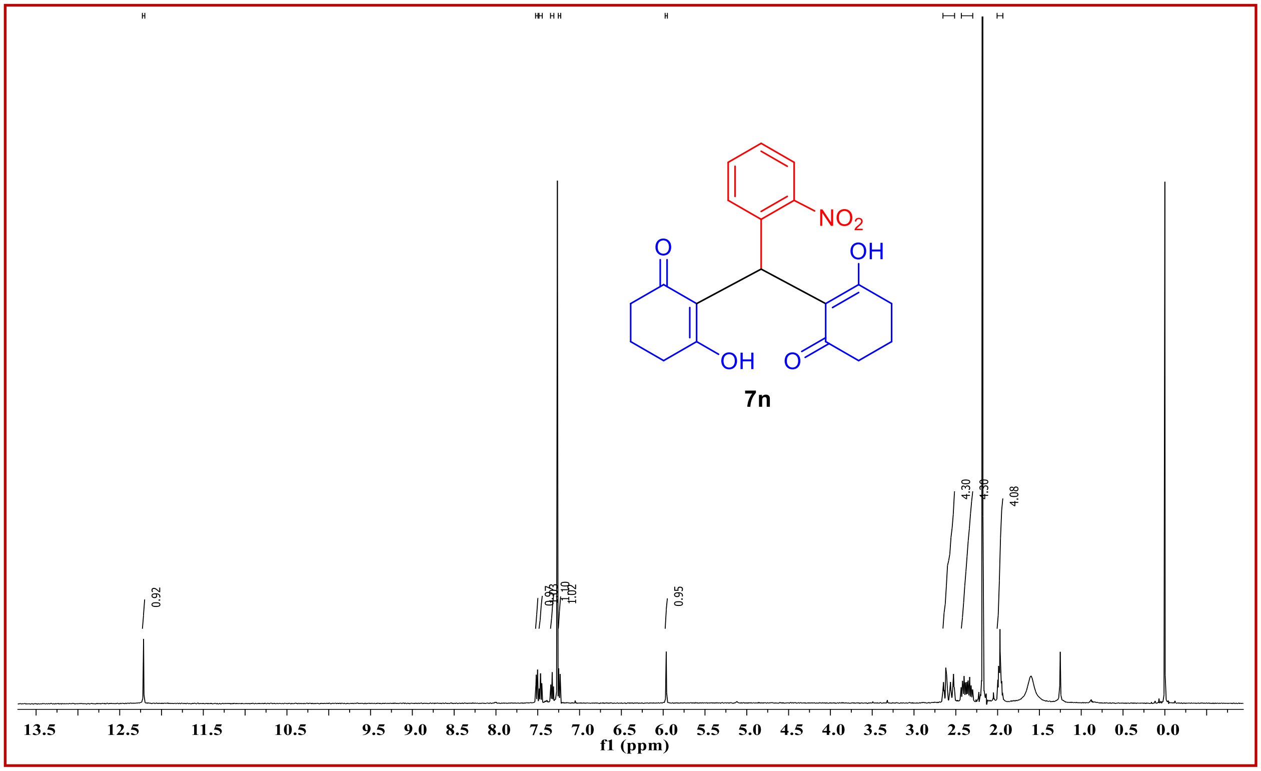


**Figure S80**: $^{1}$H NMR spectra of 2,2'-((2-nitrophenyl)methylene)bis(3-hydroxycyclohex-2-enone) **7n**

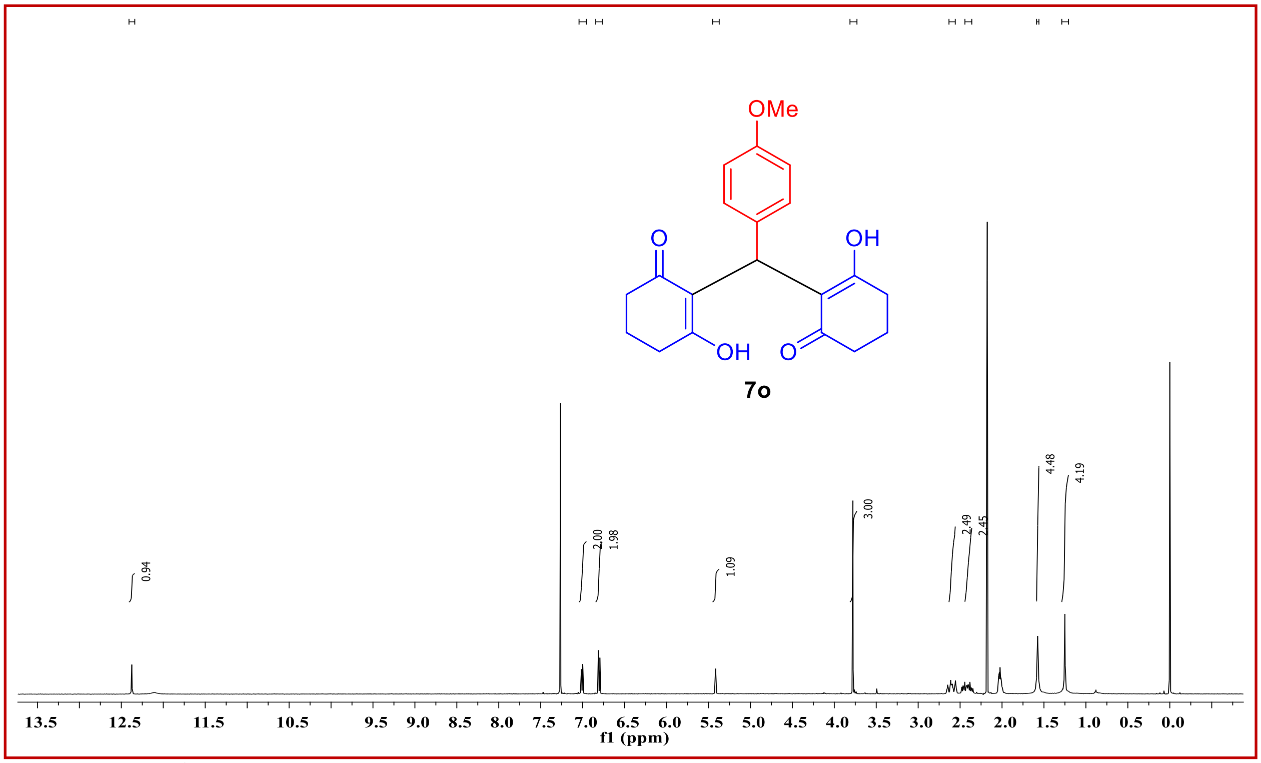


**Figure S81**: $^{1}$H NMR spectra of 2,2'-((4-methoxyphenyl)methylene)bis(3-hydroxycyclohex-2-enone) **7o**

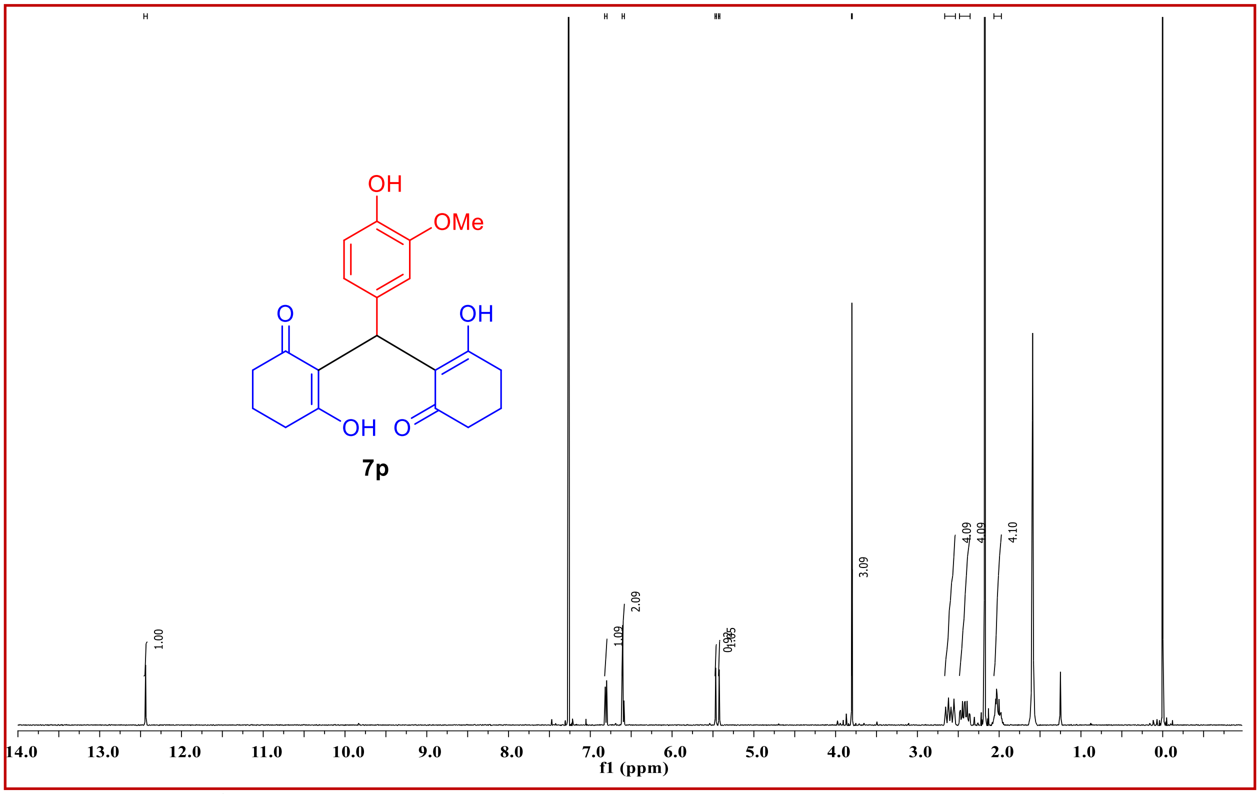


**Figure S82**: $^{1}$H NMR spectra of 2,2'-((4-hydroxy-3-methoxyphenyl)methylene)bis(3-hydroxycyclohex-2-enone) **7p**

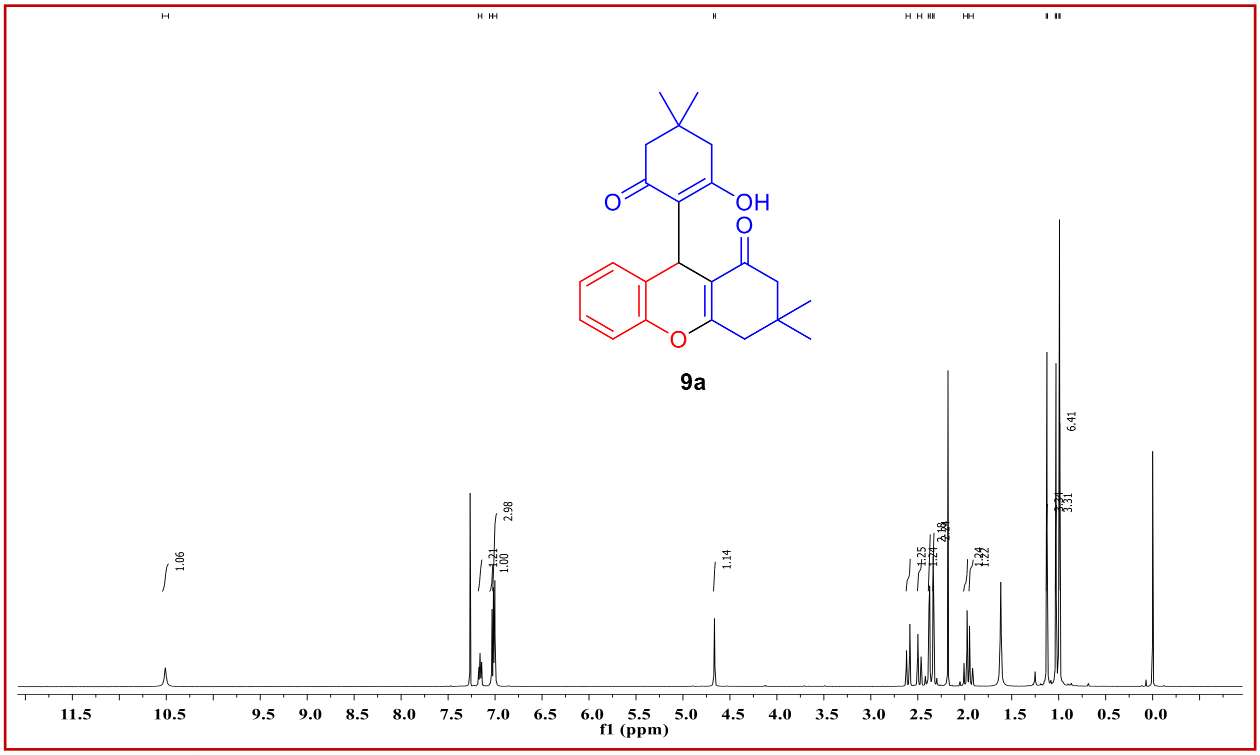

**Figure S83**: $^{1}$H NMR spectra of 9-(2-hydroxy-4,4-dimethyl-6-oxocyclohex-1-en-1-yl)-3,3-dimethyl-2,3,4,9-tetrahydro-1*H*-xanthen-1-one **9a**

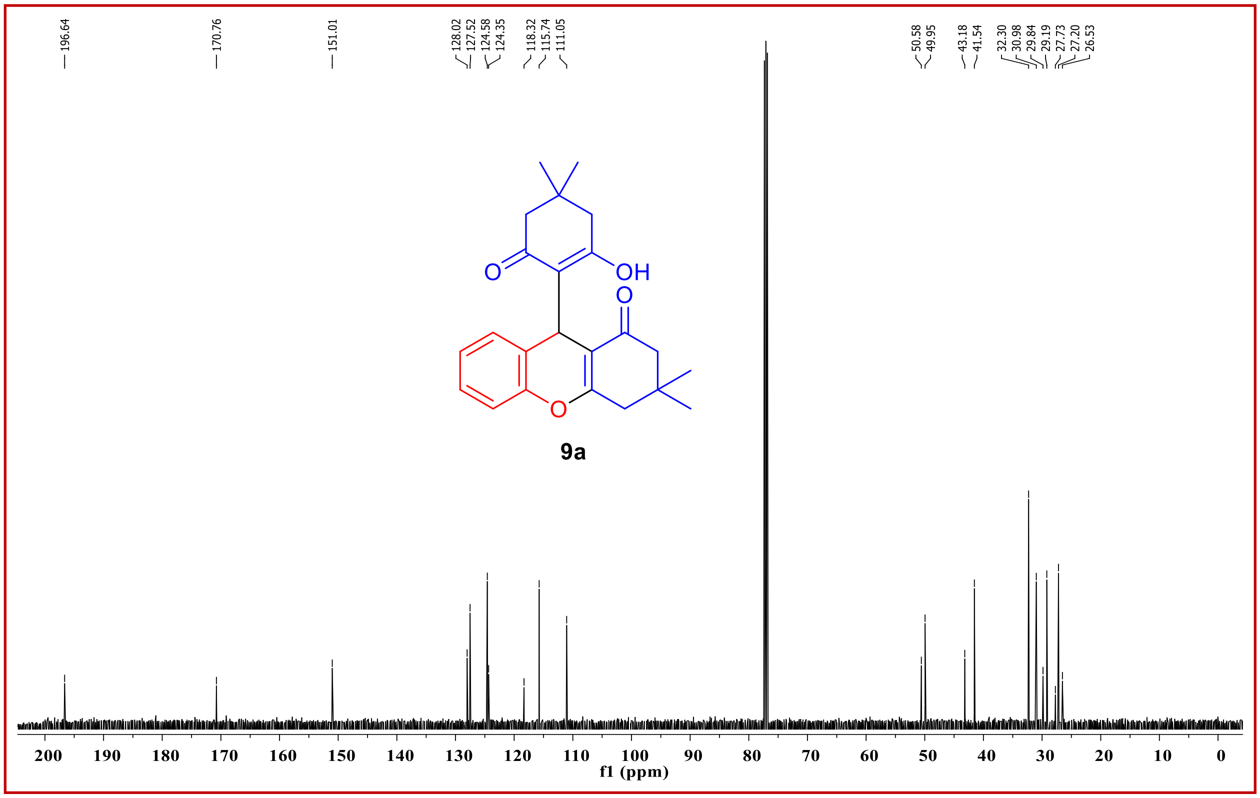

**Figure S84**: $^{13}$C NMR spectra of 9-(2-hydroxy-4,4-dimethyl-6-oxocyclohex-1-en-1-yl)-3,3-dimethyl-2,3,4,9-tetrahydro-1*H*-xanthen-1-one **9a**

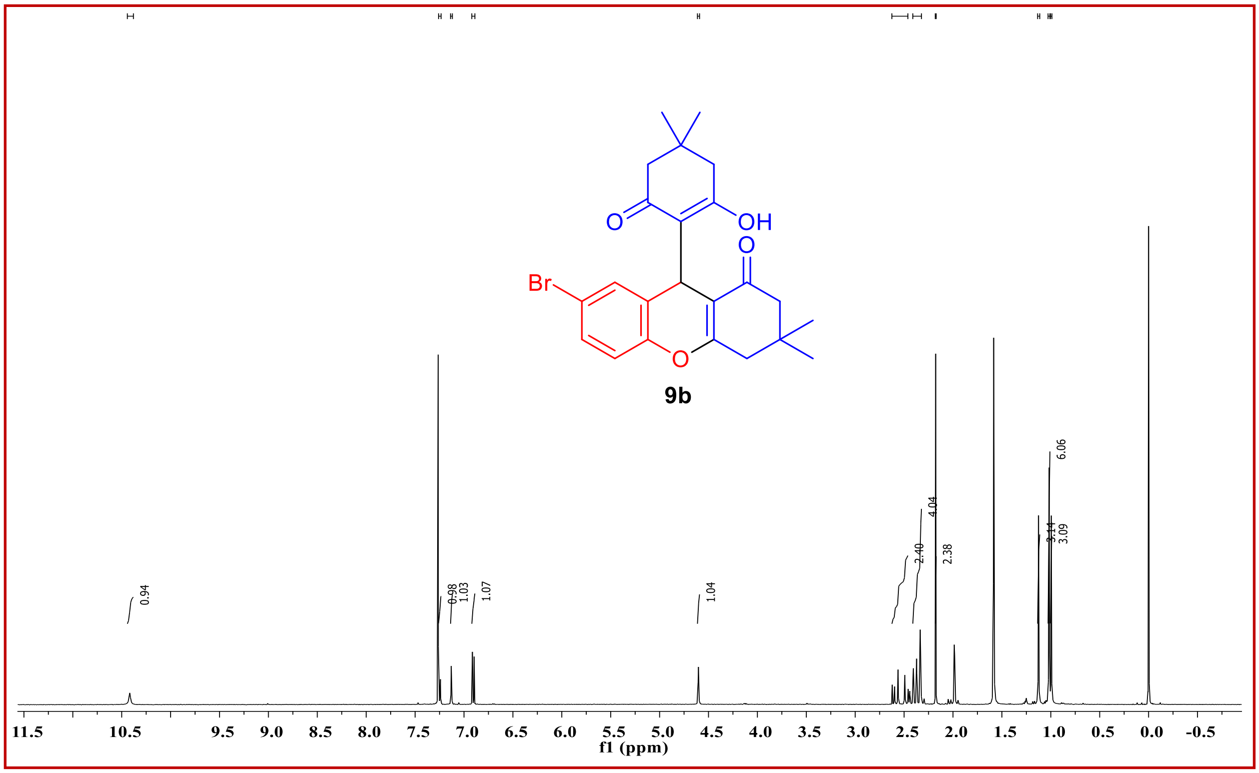


**Figure S85**: $^{1}H$ NMR spectra of 7-bromo-9-(2-hydroxy-4,4-dimethyl-6-oxocyclohex-1-en-1-yl)-3,3-dimethyl-2,3,4,9-tetrahydro-1*H*-xanthen-1-one **9b**

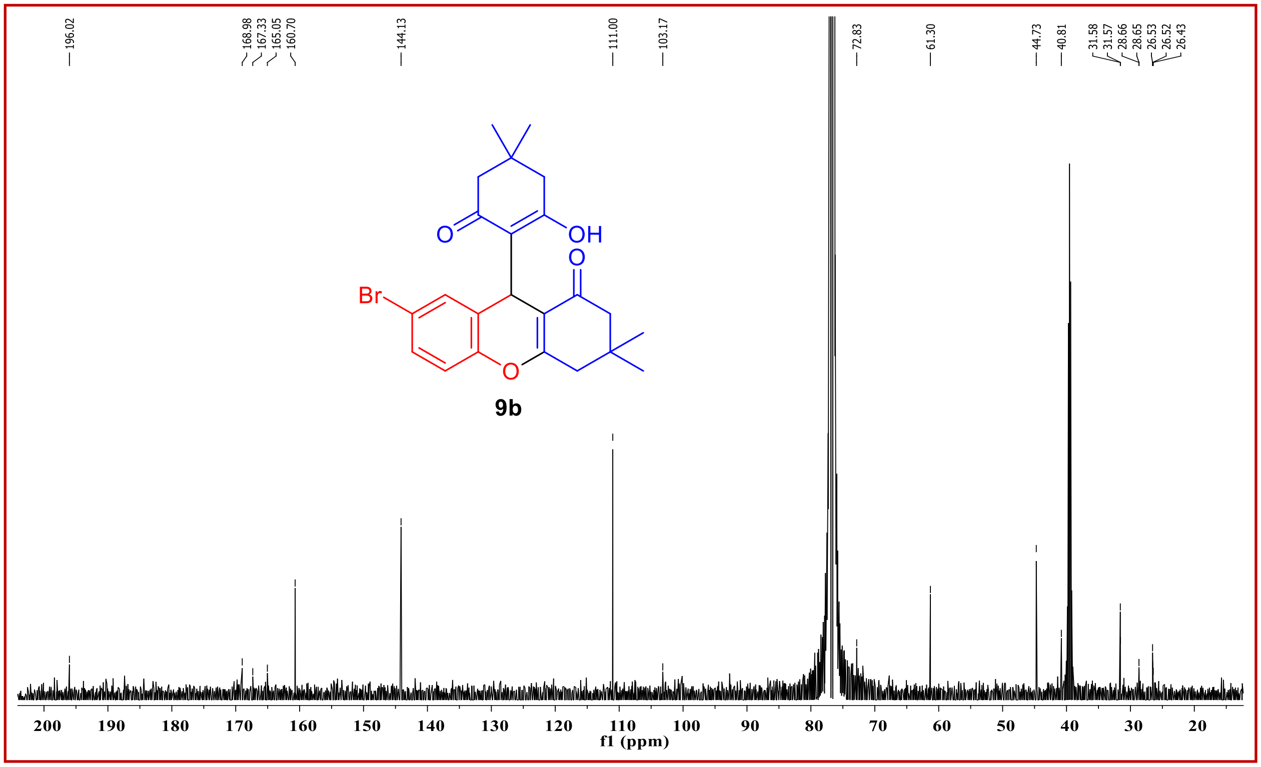


**Figure S86**: $^{13}C$ NMR spectra of 7-bromo-9-(2-hydroxy-4,4-dimethyl-6-oxocyclohex-1-en-1-yl)-3,3-dimethyl-2,3,4,9-tetrahydro-1*H*-xanthen-1-one **9b**

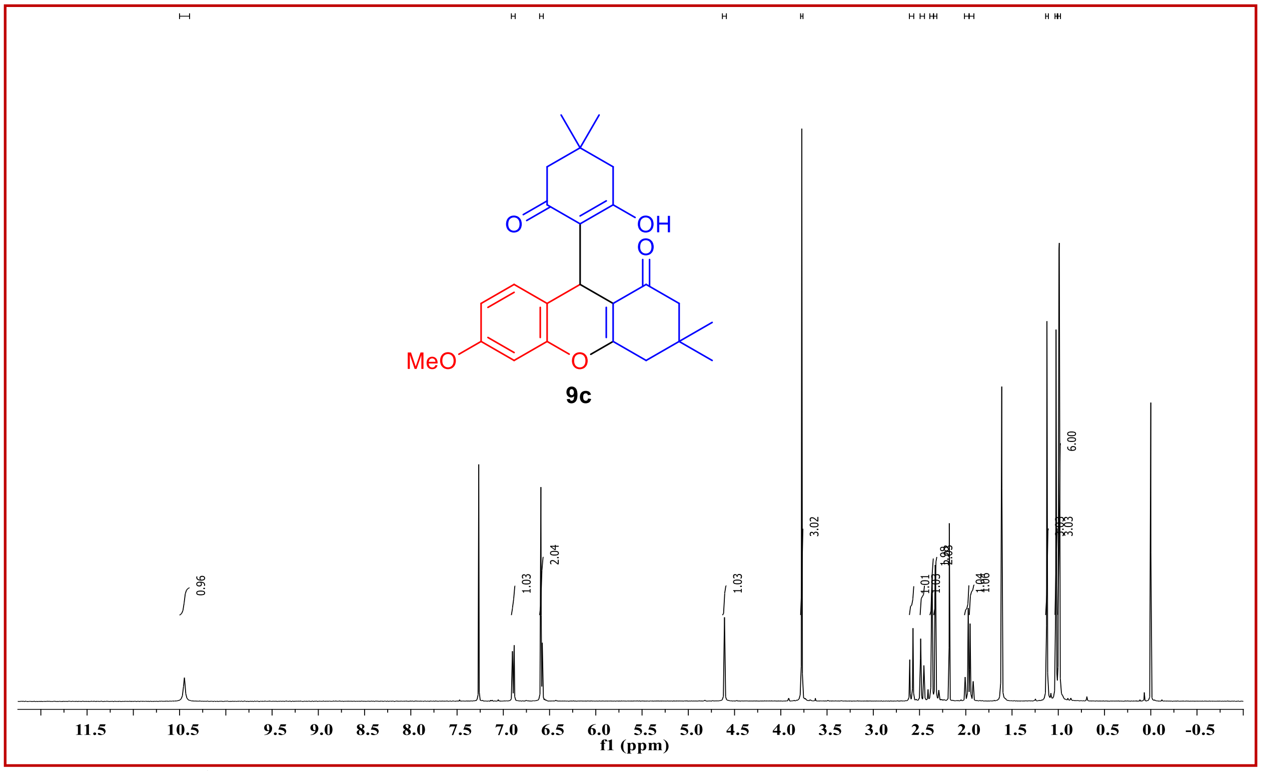


**Figure S87**: $^{1}$H NMR spectra of 9-(2-hydroxy-4,4-dimethyl-6-oxocyclohex-1-en-1-yl)-6-methoxy-3,3-dimethyl-2,3,4,9-tetrahydro-1*H*-xanthen-1-one **9c**

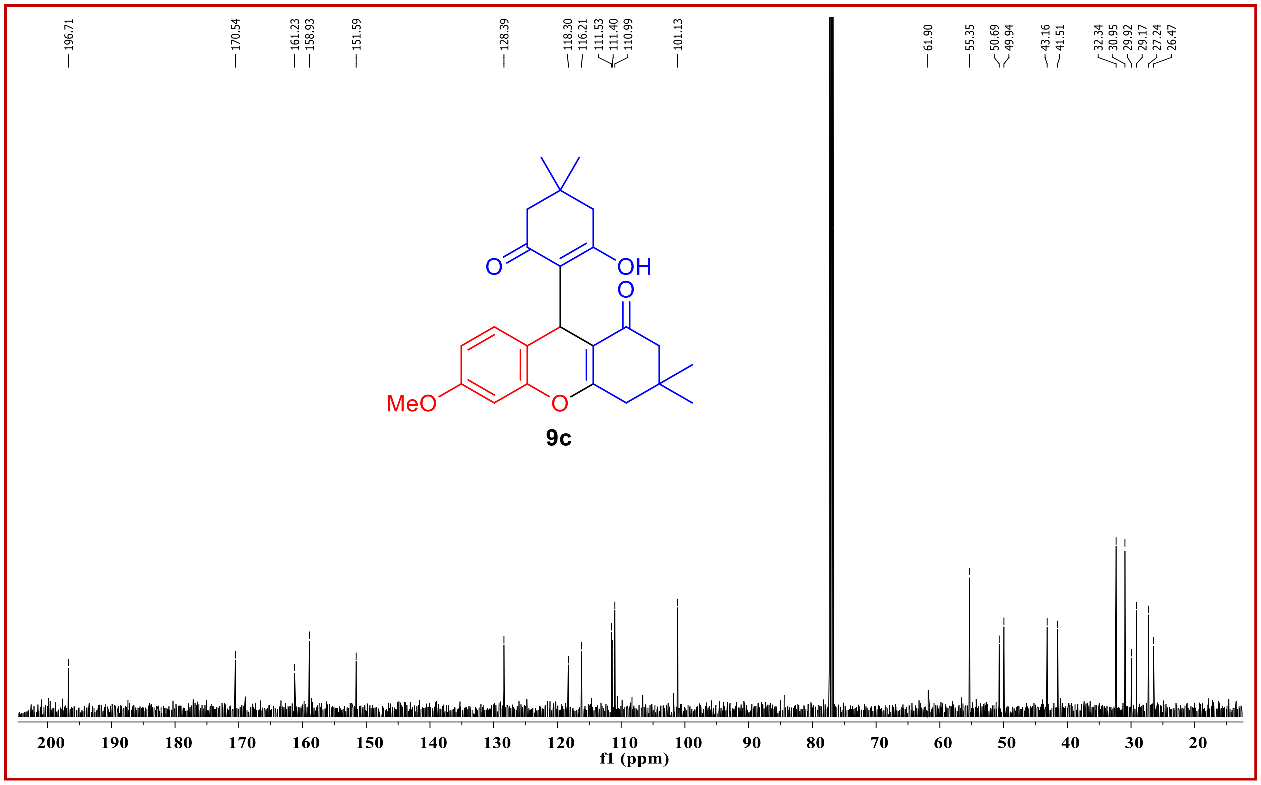


**Figure S88**: $^{13}$C NMR spectra of 9-(2-hydroxy-4,4-dimethyl-6-oxocyclohex-1-en-1-yl)-6-methoxy-3,3-dimethyl-2,3,4,9-tetrahydro-1*H*-xanthen-1-one **9c**

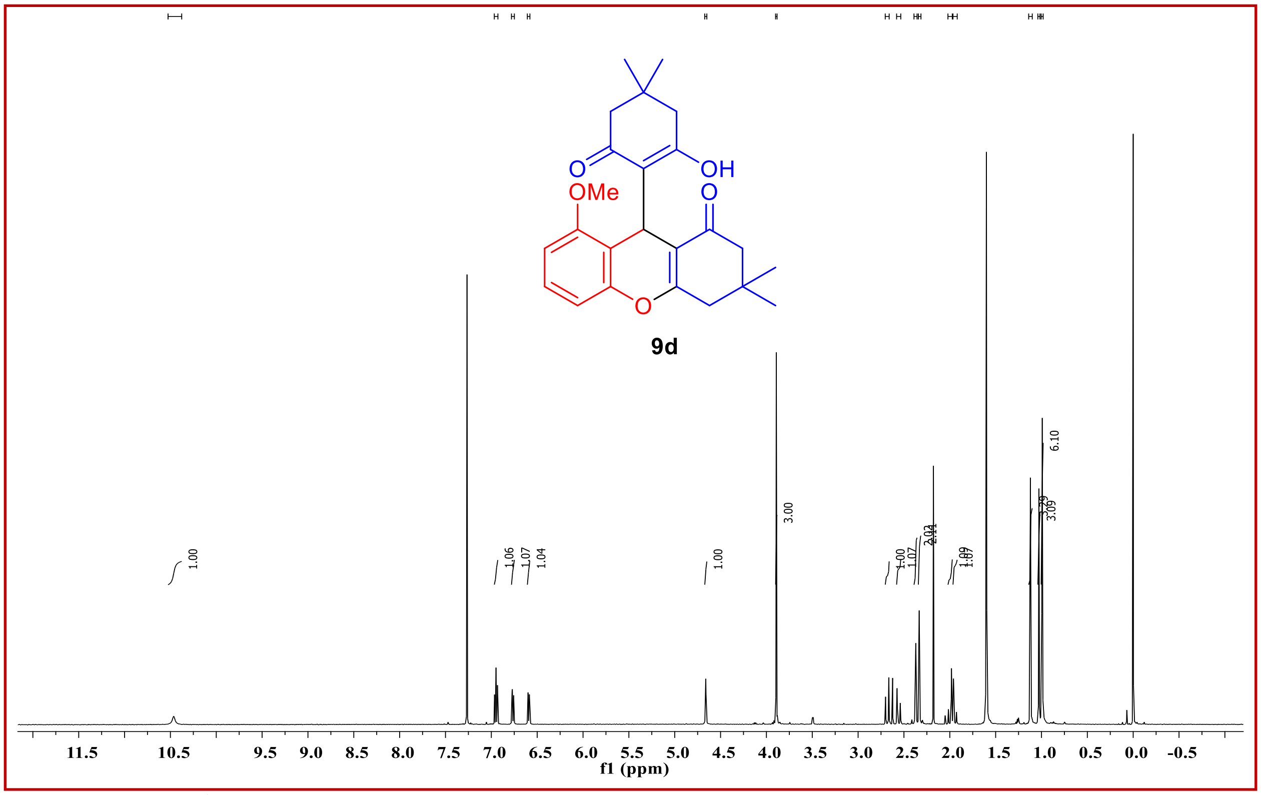


**Figure S89**: $^{1}$H NMR spectra of 9-(2-hydroxy-4,4-dimethyl-6-oxocyclohex-1-en-1-yl)-8-methoxy-3,3-dimethyl-2,3,4,9-tetrahydro-1*H*-xanthen-1-one **9d**

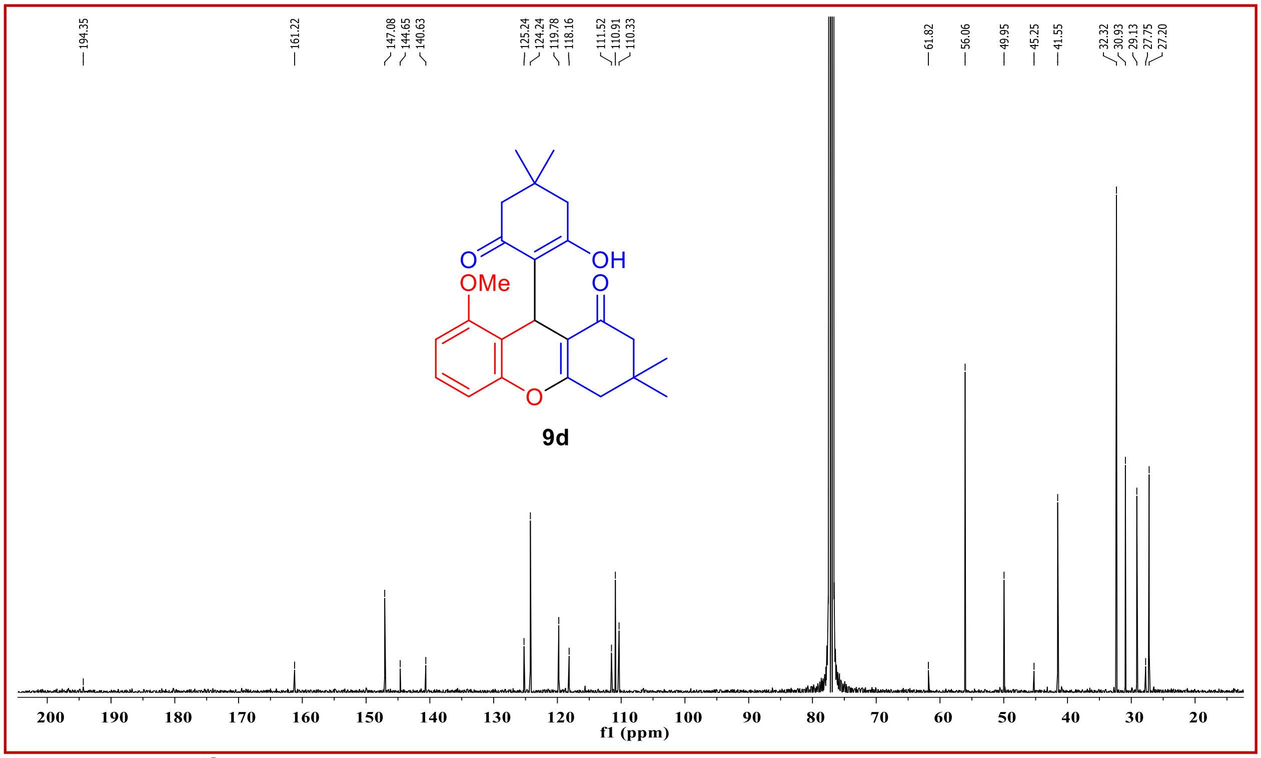


**Figure S90**: $^{13}$C NMR spectra of 9-(2-hydroxy-4,4-dimethyl-6-oxocyclohex-1-en-1-yl)-8-methoxy-3,3-dimethyl-2,3,4,9-tetrahydro-1*H*-xanthen-1-one **9d**

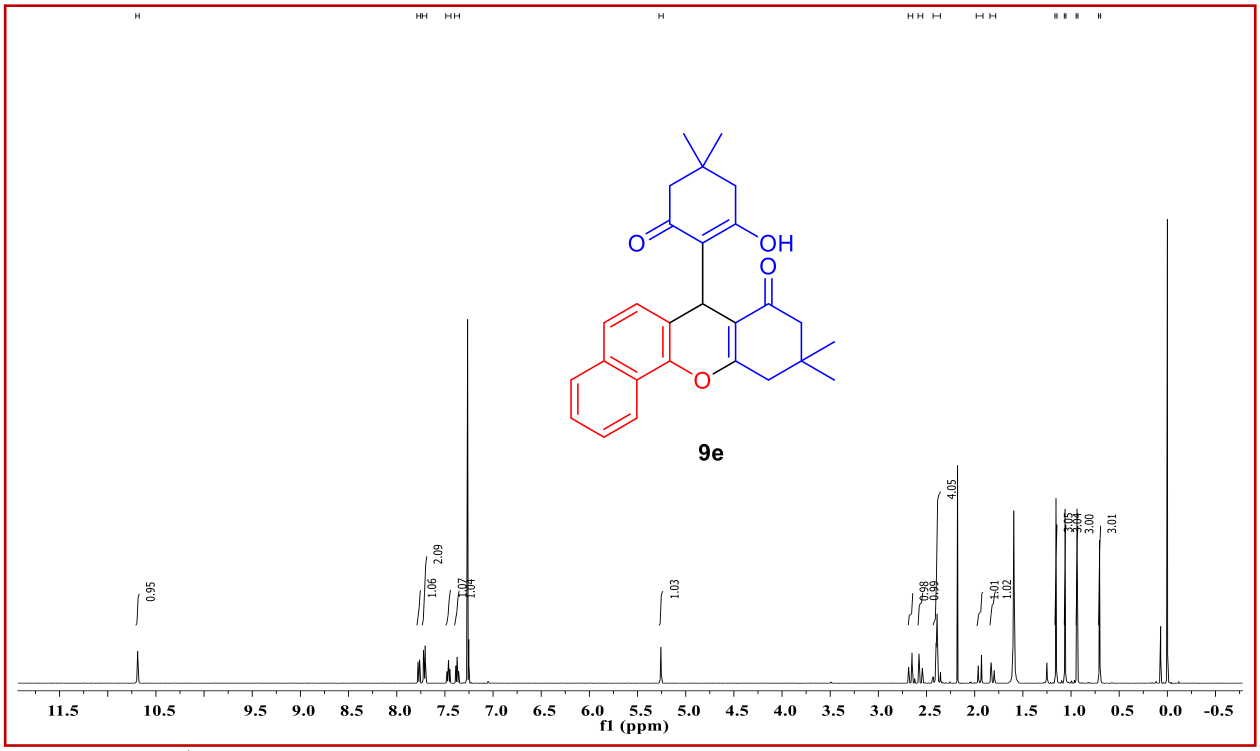

**Figure S91**: $^{1}$H NMR spectra of 7-(2-hydroxy-4,4-dimethyl-6-oxocyclohex-1-en-1-yl)-10,10-dimethyl-10,11-dihydro-7*H*-benzo[c]xanthen-8(9*H*)-one **9e**

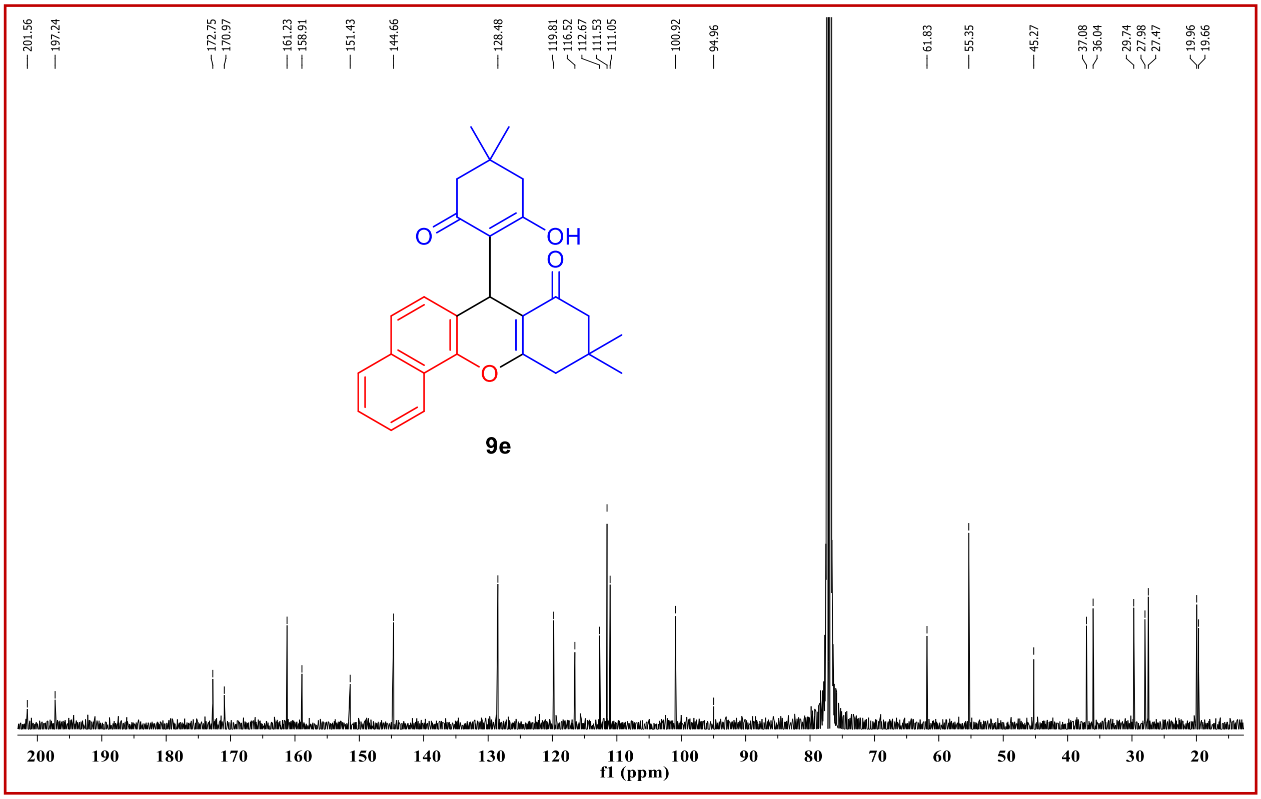

**Figure S92**: $^{13}$C NMR spectra of 7-(2-hydroxy-4,4-dimethyl-6-oxocyclohex-1-en-1-yl)-10,10-dimethyl-10,11-dihydro-7*H*-benzo[c]xanthen-8(9*H*)-one **9e**

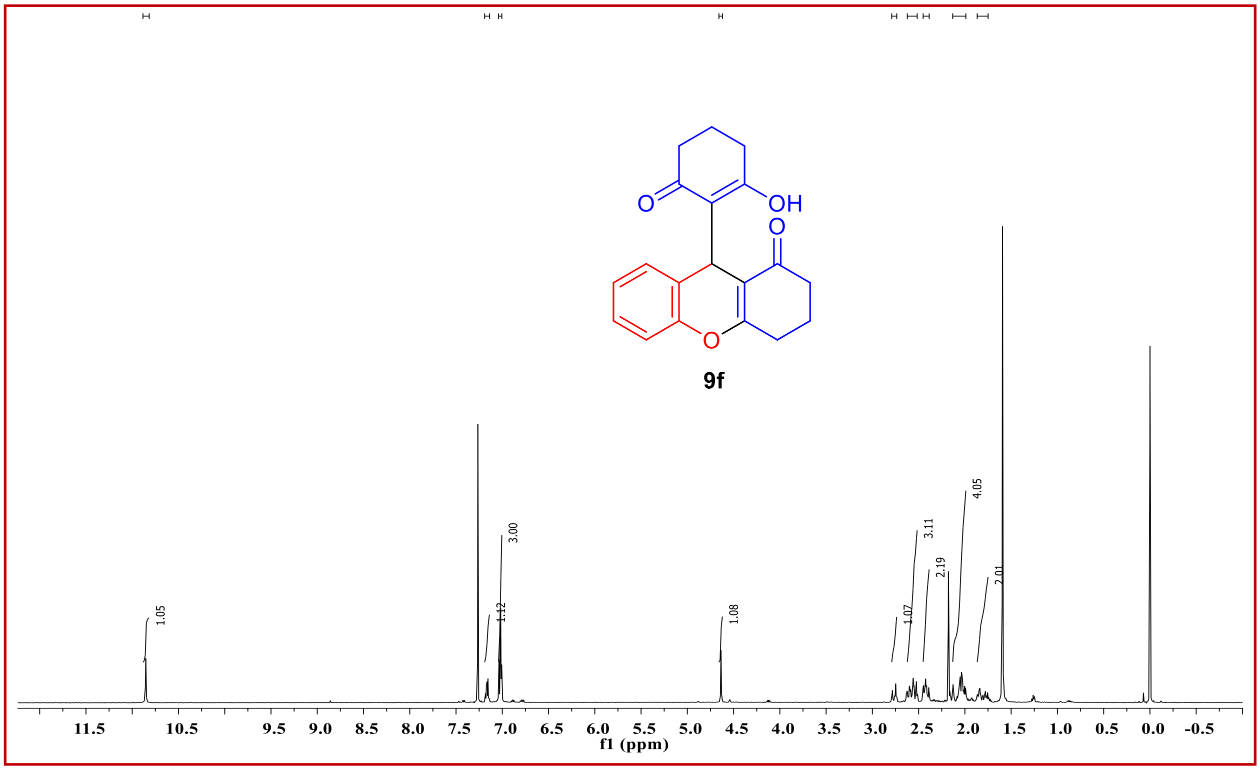


**Figure S93**: $^{1}$H NMR spectra of 9-(2-hydroxy-6-oxocyclohex-1-en-1-yl)-2,3,4,9-tetrahydro-1*H*-xanthen-1-one **9f**

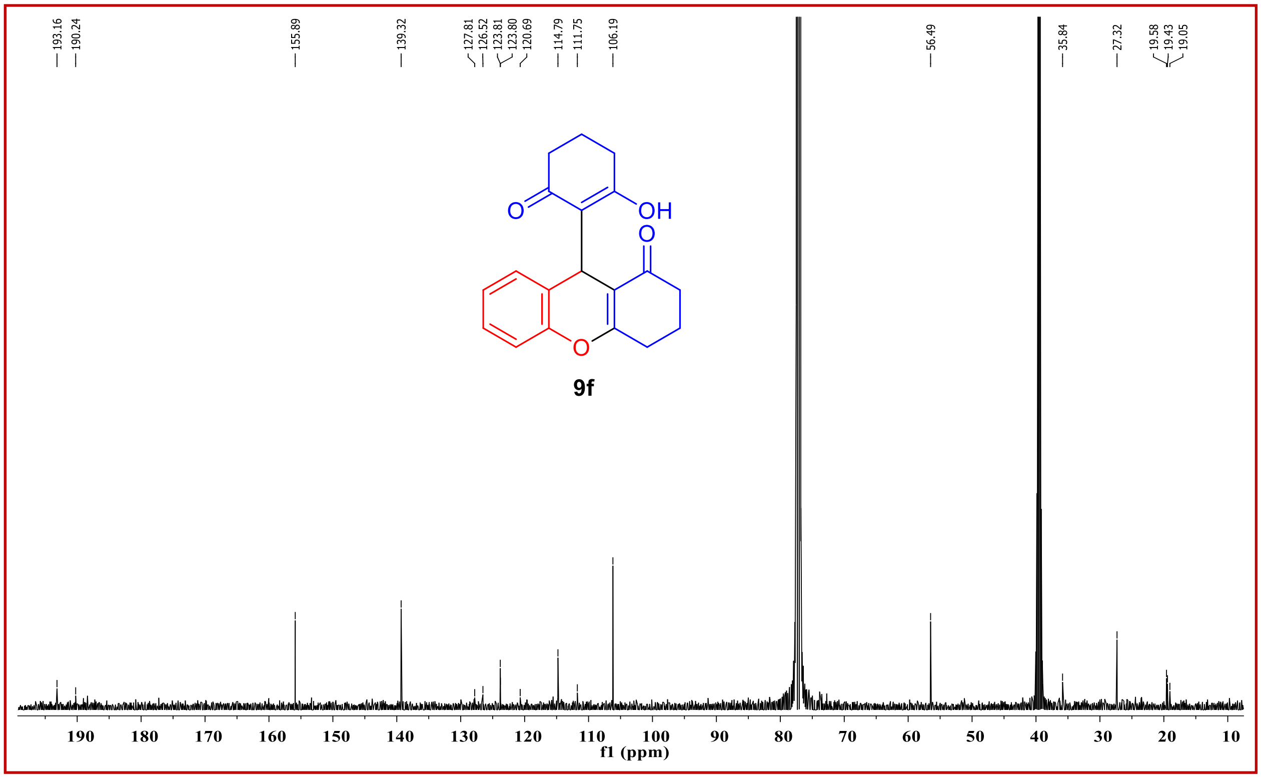


**Figure S94**: $^{13}$C NMR spectra of 9-(2-hydroxy-6-oxocyclohex-1-en-1-yl)-2,3,4,9-tetrahydro-1*H*-xanthen-1-one **9f**

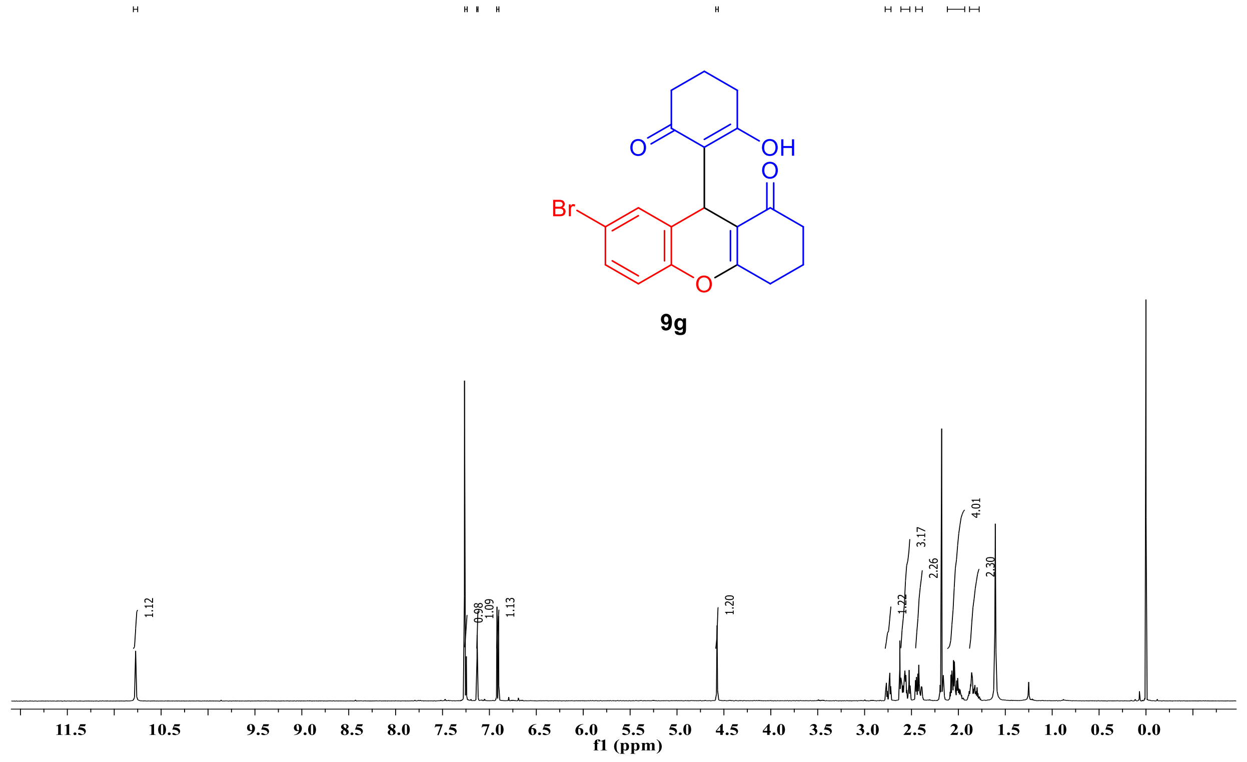


**Figure S95**: $^{1}$H NMR spectra of 7-bromo-9-(2-hydroxy-6-oxocyclohex-1-en-1-yl)-2,3,4,9-tetrahydro-1*H*-xanthen-1-one **9g**

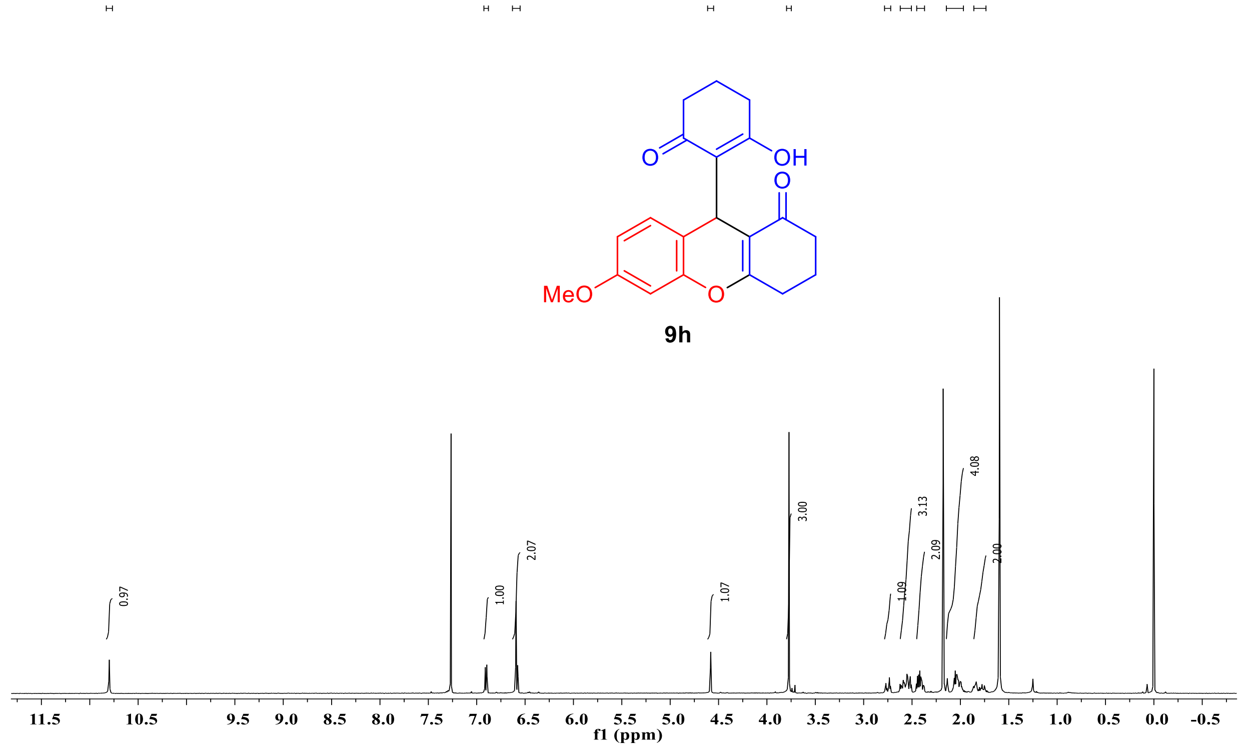


**Figure S96**: $^{1}$H NMR spectra of 9-(2-hydroxy-6-oxocyclohex-1-en-1-yl)-6-methoxy-2,3,4,9-tetrahydro-1*H*-xanthen-1-one **9h**

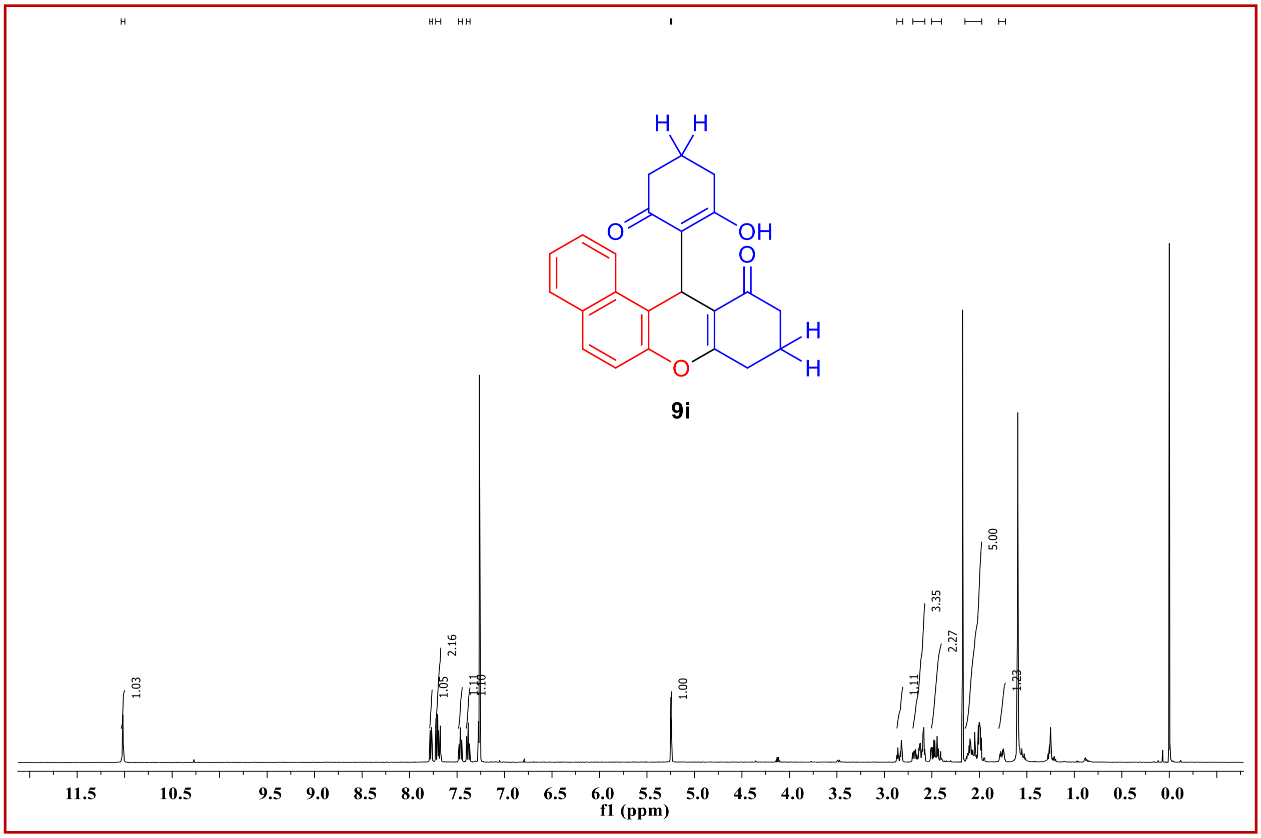


**Figure S97**: $^{1}$H NMR spectra of 7-(2-hydroxy-6-oxocyclohex-1-en-1-yl)-10,11-dihydro-7*H*-benzo[c]xanthen-8(9*H*)-one **9i**

We have studied the solvent effect theoretically by DFT calculations (B3LYP DEF2-SVP CPCMC(WATER)). The theoretical results are in complete agreement with the experimental results (**Table 4**, Manuscript).[1,2] The solvation effect was calculated for Water, Ethanol, Acetonitrile, and Hexane on intermediates **Int-3**, **Int-4**, **Int-5** and **6a**. From the theoretical calculation on optimized geometry of **Int-3-5** and **6a** in the above solvents, Water has the lowest single point energy as compared to other solvents (Water < CAN < EtOH < Hexane). The single point energy graphs are plotted for **Int-2-5** and **6a** as shown in **Figure S98**. All these intermediates has the lowest single point energy in water as solvent as compared to ACN, EtOH, and hexane. Therefore, we assume the reaction proceeds via the lowest energy pathway in water as the solvent and the same results we obtained experimentally, **Figure S98**.

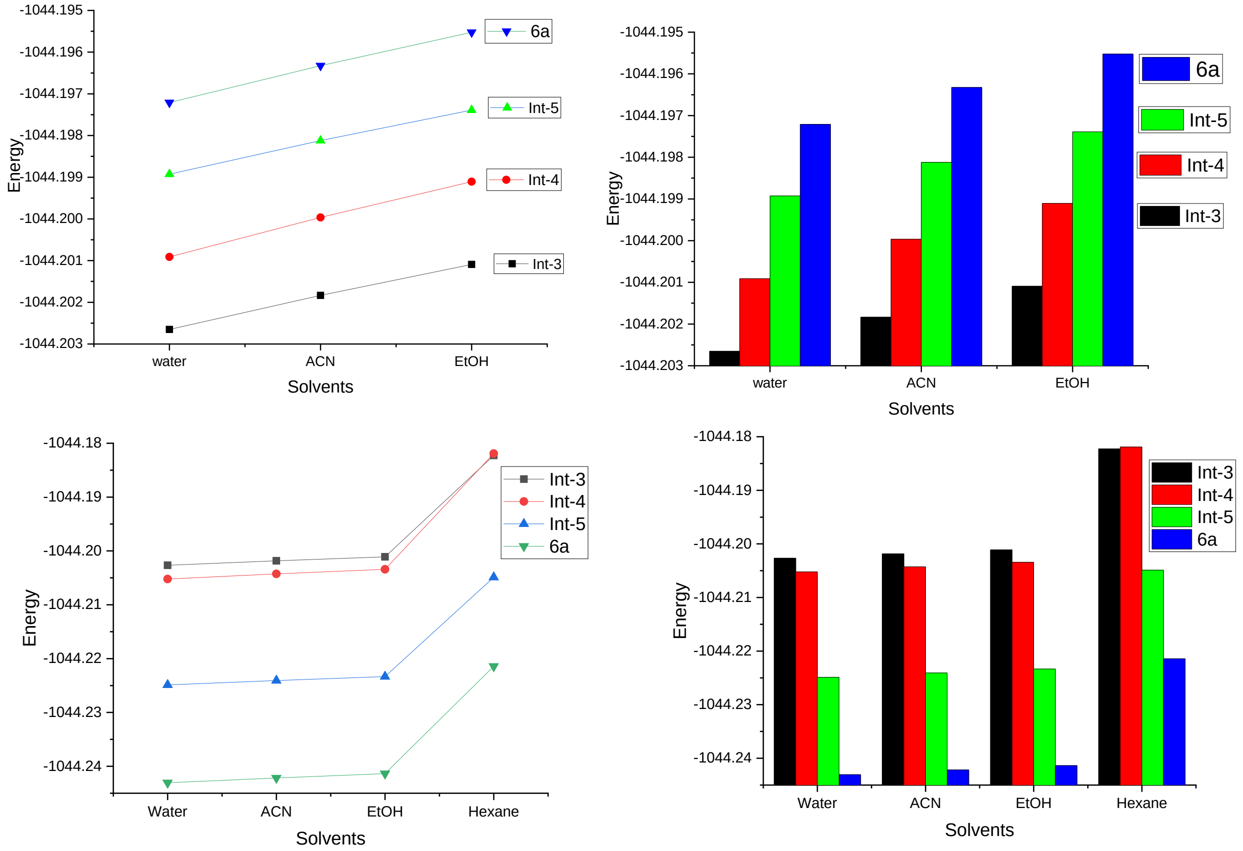


**Figure S98**: Single point energy calculation of **Int-3-5** and **6a** in various solvents.

Ref: (1) F. Weigend, *Phys. Chem. Chem. Phys.* **8**, 1057 (2006).

(2) Neese, F. Software update: the ORCA program system, version 4.0, *Wiley Interdiscip. Rev.: Comput. Mol. Sci.*, **8**, e1327 (2017).
